# Host genetic effects upon the early gut microbiota in a bovine model with graduated spectrum of genetic variation

**DOI:** 10.1038/s41396-019-0529-2

**Published:** 2019-10-17

**Authors:** Peixin Fan, Beilei Bian, Lin Teng, Corwin D. Nelson, J. Driver, Mauricio A. Elzo, Kwangcheol C. Jeong

**Affiliations:** 1 Emerging Pathogens Institute, University of Florida, Gainesville, FL 32611 USA; 2 Department of Animal Sciences, Institute of Food and Agricultural Sciences, University of Florida, Gainesville, FL 32611 USA

**Keywords:** Agricultural genetics, Metagenomics

## Abstract

Multiple synergistic factors affect the development and composition of mammalian gut microbiota, but effects of host genetics remain unclear. To illuminate the role of host genetics on gut microbiota, we employed animals with a graduated spectrum of genetic variation with minimal environmental influences. We bred 228 calves with linearly varying breed composition from 100% Angus (*Bos taurus*) to 100% Brahman (*Bos indicus*), as a proxy for genetic variation, and then raised the offspring in the same environment with identical diets. We hypothesized each breed would harbor distinct gut microbiota due to genetic influence. We found that the gut microbiota of preweaning calves at 3 months old is significantly affected by host genetics, profoundly by paternal genome. We also demonstrate that single nucleotide polymorphisms in host mucin-encoding genes, critical for gut mucosal health, are significantly correlated with both breed composition and mucin-degrading gut bacteria. We further demonstrate host genetics indirectly changes gut microbiota composition via microbe–microbe interactions. These findings indicate a strong contribution by host genetics in shaping the gut microbiota during early life stages, shedding light on impact of animal breeding on gut microbiota, which is associated with animal growth and health.

## Introduction

The diverse commensal bacterial communities in the gastrointestinal (GI) tract of humans and animals provide fundamental functions including regulating immune system development, increasing the host’s digestion capabilities, and preventing pathogen colonization [1–3]. Animal gut microbial communities are acquired and shaped dynamically after birth, and sometimes even before birth; they are complex systems that provide health-relevant functions [4–6]. Genetic coevolution between hosts and gut microbes has resulted in specific nutritional symbiosis such as short-chain fatty acid production and vitamin synthesis [7, 8], which indicates the existence of heritable microbiota. In other words, the nutritional benefits to hosts of some gut bacteria are so strong that some microbe taxa appear to be preferentially selected, either through direct contact or via genetic influence mediated by the parental genome. Due to the indispensable roles of heritable bacterial taxa, key modulators such as host genetics may determine host–microbe specificity to maintain microbial communities in the GI tract; bacterial colonization and filtering occur in response to continuous ecological interactions. Exploring how host genotypes modulate gut bacteria composition will deepen our understanding for establishing new targets to lower intestinal disorders and metabolic syndromes by maintaining homeostasis in the GI tract.

Although the degree to which host genetics shape the gut microbiota remains unclear, a growing number of studies across animal species (e.g., human, mouse, chicken, cattle, and swine) have demonstrated the role of host genetics in the gut microbiota composition [9–13]. With recent advanced technology, specifically genome-wide association study (GWAS), multiple associations have been identified between single nucleotide polymorphisms (SNPs) genotypes, primarily located in genes associated with host metabolic syndrome and immune disease, and abundance of commensal bacteria [14–16]. However, recently the statistical significance of associations between host SNPs and individual bacterial taxa has been challenged [17], and additional studies report that environmental factors dominate host genetics in shaping the gut microbiota [18, 19]. The discrepancies in interpreting the role of host genetics and environmental factors might be due to population variation, genetic distance, age, and environmental conditions. The interaction itself between environmental factors and host genetics could also mask host genetic effects in shaping the gut microbiota.

The GI tract is the largest digestive and immune organ. SNPs in the host genes that are associated with metabolic and immune functions are more frequently associated with the prevalence of commensal bacteria in the GI tract [20]. Hence, we hypothesized that animal populations with significantly different genetic backgrounds in metabolism and immune function would harbor readily apparent host genetic effects on gut microbiota compositions. For instance, Angus cattle (*Bos taurus*), the most common beef breed in the US, have a faster growth rate and better meat quality compared with the Brahman cattle breed (*Bos indicus*). Brahman cattle are more common in tropical regions due to their immunity traits of being able to resist pathogenic parasites and pathogenic bacterial colonization in their GI tract [21, 22]. They are also known for their superior heat tolerance and ability to utilize low-quality forage [23, 24]. Beef cattle breeding and genetic selection have been conducted using two breeds to generate livestock with desirable phenotypes such as high growth performance and low incidence of disease outbreaks [25–27].

To understand whether host genetics shapes the early gut microbiota and its effects on host phenotypes, we bred our study animals using a unique multibreed Angus–Brahman (MAB) herd; varying from 100% of one breed, through a gradual, mixed breed composition, to 100% of another breed. We selected 228 calves from the newborn herd. Selected calves were representative of the gradual change in genetic background, and had an even distribution of age and gender. We raised them in the same environmental conditions and fed them the same diets to minimize nongenetic influences. We then analyzed the calves’ gut microbiota composition when they were 3 months old because an animals’ early stage of life is critical due to the high incidence of disease outbreaks and rapid growth rate; the significance of the initial gut microbiota in early life stages has also been emphasized due to its role in the education of host immune systems and prolonged effects on the later stages of animal development [4, 28–30].

## Materials and methods

### Ethics statement

All operations to animals in this study followed the standard practices of animal care and use. The practices related to the animals in this study were approved by the University of Florida Institutional Animal Care and Use Committee (IACUC number 201408629 and 201803744).

### Animal genetic background and management

**Fig. 1:**
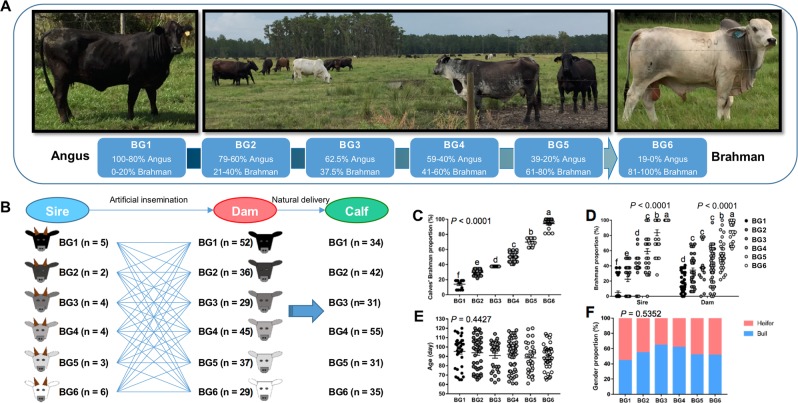
Animal breeding to generate calves with varying breed composition. **a** The unique multibreed Angus–Brahman (MAB) herd initiated in 1989 was maintained on the pasture in Florida. Cattle were divided into six breed groups (BGs) based on their breed composition. The breed composition of BG1 to BG6 ranged from 100% Angus to 100% Brahman. **b** Diallel design of mating in the MAB herd where sires from six BGs were mated to dams from six BGs. **c** Brahman proportion of 228 preweaning calves used in this study. **d** Brahman proportion of their sires and dams increased from BG1 to BG6. Age (**e**) and gender distribution (**f**) were not significantly different among six BGs. In each dotplot or barplot, values that do not have a common superscript are significantly different (*P* < 0.05) based on one-way ANOVA followed by Tukey’s HSD test for pairwise comparison of multiple means. **c**–**e** The bars represent mean ± SEM

Preweaning calves in this study were bred from the MAB herd of the University of Florida. The herd was established in 1989 to conduct long-term genetic studies in beef cattle [31]. Calves were assigned to six breed groups (BGs) according to the following breed composition ranges estimated from documented pedigree: BG1 = 100–80% Angus and 0–20% Brahman; BG2 = 79–60% Angus and 21–40% Brahman; BG3 = 62.5% Angus and 37.5% Brahman, BG4 = 59–40% Angus and 41–60% Brahman, BG5 = 39–20% Angus and 61–80% Brahman, and BG6 = 19–0% Angus, and 81–100% Brahman (Fig. 1a). Mating in the MAB herd followed a diallel design where sires from each of the six BGs were mated to dams from all six BGs [31]. The calves were naturally delivered on the pasture.

The preweaning calves were kept at the Beef Research Unit in Waldo, FL. and were raised with their dams on the same bahiagrass (*Paspalum notatum*) pastures. Animals in the herd received a complete mineral supplement (UF University Special Hi-Cu Mineral, University of Florida, Gainesville, Florida), and were provided with bermudagrass (*Cynodon dactylon*) hay and cotton-seed (*Gossypium* spp.) meal. Calf weights were taken immediately after birth and when fecal samples were collected.

### Sample collection and processing

Fecal and blood samples were collected from 228 preweaning calves (*n*_bull_ = 126, *n*_heifier_ = 102) ranging in age from 60 to 120 days in March and April 2016. Fecal samples were collected as previously described with minor modifications [32]. Briefly, fecal samples were collected from the rectal–anal junction using sterile cotton swabs. Swabs with fecal samples were placed in a 15 mL conical tube on ice and were transported on the same day to the laboratory for further processing. Each swab sample was resuspended in 2 mL of Luria-Bertani broth and 2 mL of 30% glycerol, split into four 2 mL tubes and frozen in an ultra-low freezer at −80 °C. Blood samples (10 mL per calf) were collected through the jugular by venipuncture. A portion (2 mL) of whole blood samples were stored at −20 °C for genotyping analysis. Plasma was separated from the remaining blood sample by centrifugation at 1000 × *g* for 20 min at 4 °C, and the supernatant was collected and stored at −20 °C for biochemical analysis.

### 16S rRNA gene sequencing

Genomic DNA was extracted from 500 µL of each fecal sample using the QIAamp PowerFecal DNA kit according to the manufacturer’s instructions (Qiagen, USA). The concentration and purity of the DNA were measured using a Nanodrop instrument (Spectrophotometer ND-1000, Thermo Fisher Scientific, USA). The DNA library was prepared and sequenced as described in the previous study [33]. Briefly, the V4 region of the 16S rRNA gene was amplified by polymerase chain reaction (PCR) with dual-index primers and Pfx AccuPrime master mix (Invitrogen, USA) [33]. The amplicons were purified and normalized in equimolar amounts using the SequalPrep plate normalization kit (Invitrogen, USA). The same amount of barcoded V4 amplicons from each sample were pooled to construct the DNA library. The fragment size and concentration of the DNA library were determined by tape station and Kapa quantitative PCR (qPCR) (Kapa Biosystems, USA). The final DNA library (600 μL 6 pmol/L library) was loaded into MiSeq v2, 2 × 250 cycle cartridge (Illumina, USA), and was sequenced using the Illumina MiSeq platform.

### Microbial community analysis

Raw sequencing reads were obtained from the Illumina BaseSpace website and analyzed with the Quantitative Insights into Microbial Ecology (QIIME) pipeline (version 1.9.0). Full details on 16S rRNA gene sequencing data analysis are available in Supplementary Information.

### Co-occurrence network analysis

To predict bacteria–bacteria interactions in the gut microbial community, co-occurrence patterns of core bacterial families, and genera that are present in at least 50% of samples were evaluated in the network interface using pairwise Spearman’s rank correlations (*r*_s_) based on the relative bacterial abundance according to the previous study [34]. The Spearman rank correlation was analyzed using Hmisc within RStudio (version 1.1456). A significant rank correlation between two taxa (*r*_s_ > 0.2 or *r*_s_ < −0.2, FDR-adjusted *P*-value *<* 0.05) was considered as a co-occurrence event. The network was visualized using the Force Atlas algorithm in the interactive platform Gephi (http://gephi.org). In the network, nodes represented different taxa, and edges indicated correlations among nodes. The size of the nodes represented the degree of connection, and the thickness of edges indicated the strength of the correlation.

### Functional prediction of gut microbiome

Functional capacity of the gut microbial community was predicted using PICRUSt (phylogenetic investigation of communities by reconstruction of unobserved states) online Galaxy version (http://galaxy.morganlangille.com/). The closed reference OTU table was generated by picking OTU against the 13 August 2013 Greengenes database using QIIME (version 1.9.0). Normalization of copy numbers, metagenome prediction, and function categorization based on Kyoto Encyclopedia of Genes and Genomes pathways were conducted using the online Galaxy version on Huttenhower Lab (v1.0.0) servers according to a standard analysis process [35].

### Quantitative real-time PCR analysis

The qPCR was conducted to confirm the differences in the relative abundance of *Faecalibacterium prausnitzii* and *Clostridium perfringens* between BG1 and BG6. Full details on the qPCR analysis are available in Supplementary Information.

### Animal genotyping

Animal genotyping was conducted as previously [36]. Briefly, DNA was extracted from calf blood samples using the QIAamp DNA mini kit according to the manufacturer’s instructions (Qiagen, USA). DNA samples were genotyped with GeneSeek Genomic Profiler F-250 at Neogen Corporation (GGP F-250, Neogen Genomics, USA). Quality control (QC) was conducted using the software PLINK1.9. QC filters included genotype completion rate (<90%), minor allele frequency (<1%), genotype call rate (<90%), and Hardy–Weinberg equilibrium deviation (chi-square *P*-value < 10^−8^). After removing samples with low genotype completion rate (<90%), 220 out of 225 samples were available for subsequent analysis. From an initial set of 221,049 SNPs, 77,948 autosomal SNPs passed QC filters and were used for principal component analysis (PCA). The PCA was conducted with the input of SNP genotyping matrix (0: reference homozygous, 1: heterozygous variant, 2: homozygous variant, 5: missing) using the prcomp function of RStudio (version 1.1456). The correlation between the Brahman proportion estimated by pedigree and top PC (PC1) was evaluated by Pearson’s correlation coefficient.

### Detection of blood parameters

The plasma glucose and nonesterified fatty acids (NEFA) concentrations were determined using glucose kit and NEFA kit (Randox Laboratories Ltd, UK), respectively. An automated RX series Clinical Chemistry Analysers (Randox Laboratories Ltd, UK) was used for all measurements. The plasma IgG1 concentrations were detected using a bovine IgG1 ELISA Quantitation set (Bethyl Laboratories, USA) according to the manufacturer’s protocol. Plasma was diluted in Tris-buffered saline (TBS)-Tween to a final dilution factor of 4 × 10^4^. All dilutions were duplicated. Absorbance was read using a BioTek Synergy plate reader (BioTek Instruments, Inc., USA) at a wavelength of 450 nm.

### Statistical analysis

All statistical analyses were conducted using RStudio (version 1.1456). The normal distribution of variables was assessed using the Shapiro–Wilk’s test with the shapiro.test function. The nonnormal values were log-transformed before downstream analysis. For the relative abundance of specific bacterial taxa that were not present in all the samples, a small numeric constant (half of the detection limit: 0.00003663) was added to all values before logarithm transformation.

Fold differences in the copy number of *F. prausnitzii* and *C. perfringens* between BG1 and BG6 were analyzed by Student *t*-tests by using the *t*.test function. Differences in age, breed composition, gender among BGs, as well as differences in the relative abundance of mucin-degrading bacteria among calves with different SNP genotypes were analyzed by using the one-way analysis of variance (ANOVA) test followed by Tukey’s honestly significant difference (HSD) test for pairwise comparison of multiple means. The aov and TukeyHSD functions were used, respectively. A *P*-value < 0.05 was considered to be statistically significant, and 0.05 < *P*-value < 0.1 was considered as tendency towards significance.

A multiple linear regression model was applied to analyze the fixed effects of breed composition, age, and sex on response variable including Chao 1, Shannon index, the relative abundance of core bacteria, microbial function, weight gain, and blood parameters. Associations between SNP genotypes and the relative abundance of bacteria were accessed using a multiple linear regression model with the fixed effects of Brahman proportion, SNP genotype, age and sex, and the dependent variable of relative abundance of mucin-degrading bacteria. The potential contribution of bacteria on animal phenotypes was assessed using a multiple linear regression model with the fixed effects of the relative abundance of bacteria, Brahman proportion, age and sex, and the dependent variable of weight gain and blood parameters. The glm function was used to fit the generalized linear models. The Akaike information criterion was used to choose the best model.

Correlations between average of Brahman proportion in each BG and prevalence of OTU in each BG were assessed by Pearson correlation coefficients. Correlations between Brahman proportion and genotype of SNPs in or near mucin-encoding genes were assessed by Spearman rank correlation coefficients. The correlations were analyzed by using the cor.test function. The *P-*values were adjusted using the false discovery rate (FDR) method for multiple comparison with the p.adjust function. For Pearson correlation, an FDR-adjusted *P-*value < 0.05 was considered statistically significant. For the Spearman rank correlation, the significant correlation was considered with coefficient > 0.2 or < −0.2, as well as an FDR-adjusted *P-*value < 0.05.

## Results

### Animal breeding for generation of a herd with varying breed composition

To understand the influence of host genetics on early development of gut microbiota composition, we bred calves with different breed composition using a unique MAB herd (Fig. 1a). We selected 228 preweaning calves from a total of 278 newborn calves, based on breed composition, age, and gender. Twenty-four sires from six BGs were mated to 228 dams from the same six BGs, resulting in 228 calves that were naturally delivered (Fig. 1b). All calves were raised in the same environmental conditions on pasture with their dams. The calves were also assigned into six BG based on their breed composition, ranging from 100% Angus to 100% Brahman (Fig. 1c). The information of preweaning calves including age, sex, and breed composition, as well as breed composition of their sires and dams is presented in Supplementary Table S1. The Brahman proportion of calves (Fig. 1c), as well as that of their sires and dams (Fig. 1d) gradually increased from BG1 to BG6, with a similar distribution of age ranges (Fig. 1e) and gender (Fig. 1f) across the six BGs.

**Fig. 2:**
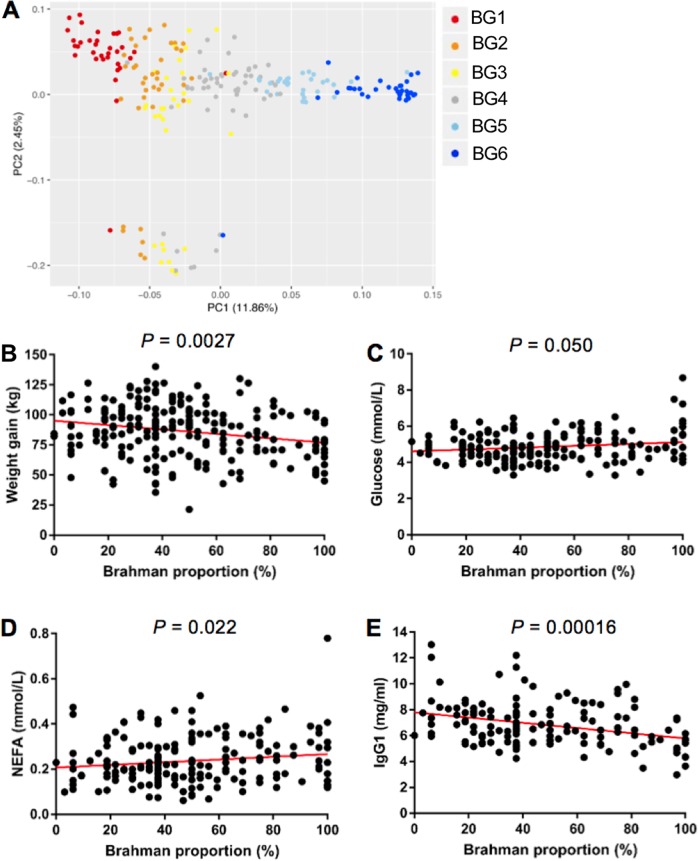
Genotype and phenotype of the generated calves. **a** The PCA plot based on SNP genotyping data shows the genetic distance of the calves across six BGs. The color of the dots represents the BG of calves estimated by pedigree. **b** Weight gain of the preweaning calves was negatively correlated with calves’ Brahman proportion. **c** Plasma glucose level tended to be positively correlated with calves’ Brahman proportion. **d** Plasma nonesterified fatty acid (NEFA) level was positively correlated with calves’ Brahman proportion. **e** Plasma IgG1 level was negative correlated with calves’ Brahman proportion. **b**–**e** The Brahman proportion was estimated by pedigree

The gradual change of genetic composition of the study herd was evaluated by measuring genetic distance and physiological parameters (Fig. 2). In the PCA plot, the first and second PCs (PC1 and PC2) explained 11.86% and 2.45% of the variation in the entire genetic data, respectively (Fig. 2a). The PC1 had a very strong correlation (*R* = 0.97, *P* = 2.2 × 10^−16^) with the Brahman proportion estimated by pedigree (Supplementary Fig. 1), indicating the strong agreement on population structure between estimation from pedigree and evaluation by SNP genotyping.

For physiological analysis, a multiple linear regression model was applied including Brahman proportion, age in days, and gender as three independent variables, and phenotypes including weight gain, glucose, NEFA, and IgG1 concentrations as dependent variables. All the phenotypes considered in this study were significantly associated with breed composition, or tended to be (Fig. 2b–e). Calves with higher Brahman proportion gained less weight (Fig. 2b, *P* = 0.0027), and had higher plasma glucose (Fig. 2c, *P* = 0.050) and NEFA levels (Fig. 2d, *P* = 0.022). These data are consistent with previous studies which show slower growth rate and higher energy expenditure of Brahman calves compared with Angus calves [37, 38]. In addition, we observed less plasma IgG1 (Fig. 2e, *P* = 0.0002) level in calves with higher Brahman proportion, indicating variation in systemic immune function among the calves with different breed composition. This may support the previous observation that the *Bos indicus* breed is more resistant to parasites compared with the *Bos taurus* breed partly due to the distinct immune signature in the skin [39]. Taken together, these data indicate that we generated calves belonging to six BGs with varied breed composition that was consistent with the measured phenotypes.

### The gut microbiota composition differs with breed composition of calves

To characterize the early gut microbiota of MAB calves, the 16S rRNA gene sequencing was conducted. An average of 114,771 ± 2917 (mean ± SEM) raw paired-end raw reads were generated per fecal sample, clustering into 40850 ± 756 (mean ± SEM) OTUs, ranged from 13,656 to 81,888 (Supplementary Table S2). The sequencing depth was normalized to 13,650 per sample for downstream analysis.

**Fig. 3:**
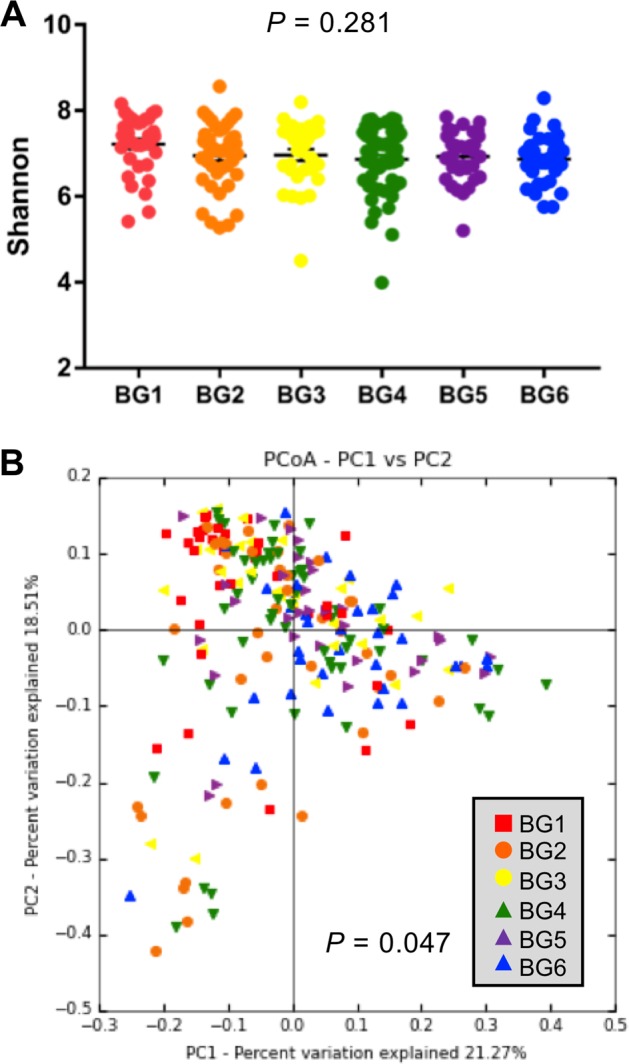
Alpha and beta diversity of gut microbiota of preweaning calves across six BGs. **a** Shannon index among six BGs. The difference in Shannon index across six BGs was analyzed using one-way ANOVA followed by Tukey’s HSD test for pairwise comparison of multiple means. The bars represent mean ± SEM. **b** PCoA plot of weighted UniFrac community distance comparing gut microbiota structure among six BGs. The difference in weighted UniFrac distance among BGs was analyzed by analysis of similarities (ANOSIM)

Although the alpha diversity measured by Shannon index was similar among six BGs (Fig. 3a, *P* > 0.05), the beta diversity measured by weighted UniFrac distances accounting for dissimilarity in both presence and abundance of bacteria in the GI tract was significantly different among the six BGs (Fig. 3b, *P* *=* 0.047). As shown in the PCoA plot, PC1 and PC2 explained a 40% variation in the gut microbiota composition among 228 calves (Fig. 3b). BG6 that has the farthest genetic distance showed clear separation with BG1 in the PCoA plot (Supplementary Fig. 2E). However, closer genetic BGs, BG2, BG3, BG4, and BG5, showed less separation with BG1 compared with BG6 (Supplementary Fig. 2A–D), suggesting different gut microbiota structure affected by a gradual change in breed composition.

**Fig. 4:**
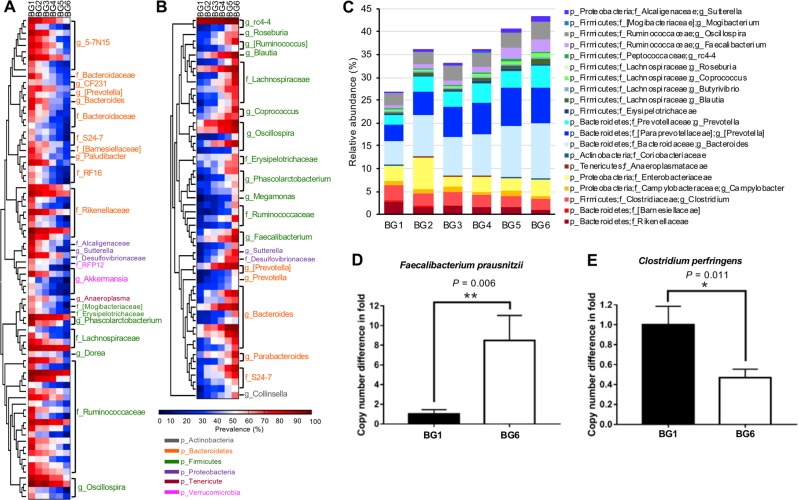
Gut microbiota composition of preweaning calves across six BGs. **a**, **b** Heatmaps represent the prevalence of OTUs across six BGs. Only OTUs that were at least classified at the family level and with their prevalence showing significant positive or negative correlations with Brahman proportion were included in the heatmaps (*P*_adjust_ < 0.05). **c** The relative abundances of core bacteria (identified in at least 80% of the 228 calves) across six BGs. Only bacteria that were linearly influenced by breed composition, analyzed by a multiple linear regression model, were included in the bar graph. **d** Fold difference in the copy number of *Faecalibacterium prausnitzii* between BG1 and BG6. **e** Fold difference in the copy number of *Clostridium perfringens* between BG1 and BG6. **d**, **e** Data are presented as mean ± SEM, with statistical differences. *P < 0.05; **P < 0.01

To identify specific bacteria affected by breed composition, correlations between breed composition and the prevalence as well as the relative abundance of bacteria were analyzed. Among the 734 OTUs that were present in more than 50% of samples in at least one BG, prevalence of 20.8% (153 OTUs) showed significant correlation with breed composition after correction of multiple comparison (Supplementary Table S3, *P*_adjust_ < 0.05). There were 78 OTUs classified at least at the family level having higher prevalence in calves with more Angus breed proportion (Fig. 4a), and the other 56 having higher prevalence in calves with more Brahman breed proportion (Fig. 4b). These breed-associated OTUs primarily were belonging to Ruminococcaceae and Bacteroidaceae. To explore the influence of breed composition on the bacterial abundance in the GI tract, a multiple linear regression model was applied with three independent variables: Brahman proportion, age of calf in days, and gender; and one dependent variable was applied with the relative abundance of core bacteria present in at least 80% of fecal samples. As a result, the relative abundance of classified core bacterial family or genus that constituted about 30% of the microbial community was identified to be significantly linearly associated with breed composition (Fig. 4c and Supplementary Table S4).

Interestingly, bacteria that were enriched in preweaning calves with more Brahman breed proportion included *Coprococcus, Faecalibacterium*, *Blautia*, and *Butyrivibrio* that are fiber-digesting and beneficial butyrate-producing bacteria [40–43]. Consistently, microbial genes involved in carbohydrate metabolism were enriched in preweaning calves with higher Brahman proportion (*P* = 0.0012) predicted by PICRUSt, especially for genes participating in the metabolism of fructose, mannose, galactose, starch, and sucrose (Supplementary Fig. 3). In contrast, bacteria enriched in calves with higher Angus proportion were predicted to have a stronger ability to metabolize lipids and amino acids (Supplementary Fig. 3). *Campylobacter* and Enterobacteriaceae, which contain species of pathogenic bacteria that commonly trigger calf diarrhea [44, 45], and mucin-degrading bacteria such as *Akkermansia*, Rikenellaceae, and *Clostridium* were more abundant in BGs with a higher proportion of Angus breed. Mucin is a crucial component of the gut mucosal barrier [46]. Elevation of mucin-degrading bacteria that use mucin as a source of both carbon and nitrogen has been reported to result in an increased susceptibility to GI pathogens due to a reduction in the intestinal barrier [47].

To validate the differences in the bacterial abundance measured by the 16S rRNA gene sequencing, two bacterial species, *F. prausnitzii* and *C. perfringens* were selected for qPCR confirmation in BG1 and BG6. These two bacteria are representative of butyrate-producing bacteria and opportunistic pathogenic bacteria, respectively. Consistent with the 16S rRNA gene sequencing data, the copy number of *F. prausnitzii* in BG6 was eight times higher than BG1 (Fig. 4d, *P* = 0.006); whereas, the pathogenic bacteria *C. perfringens* in BG6 was about 40% of BG1 (Fig. 4e, *P* = 0.011).

### Sire breed composition primarily explains the differences in gut microbiota structure among breed groups of preweaning calves

**Fig. 5:**
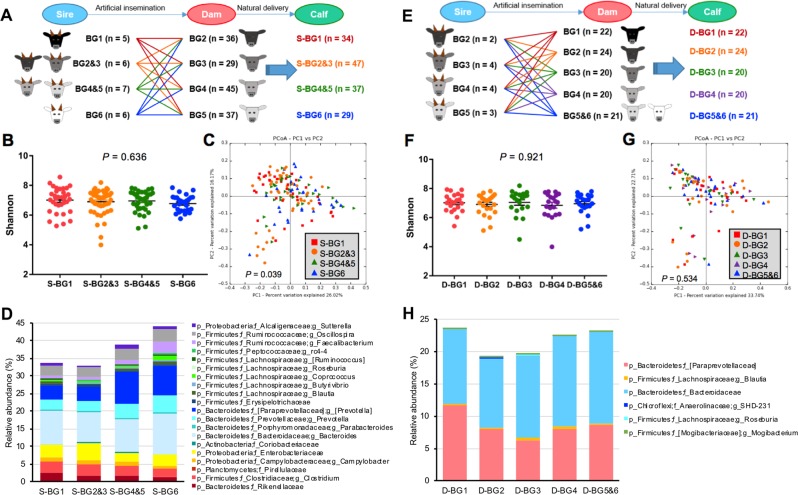
Sire breed composition mainly explains the linear change of the gut microbiota of preweaning calves among six BGs. **a** Preweaning calves were regrouped into four sire-BGs based on their sire breed composition. **b** Shannon index among four sire-BGs. **c** PCoA plot of weighted UniFrac community distance comparing gut microbiota composition among four sire-BGs (S-BGs). **d** The relative abundances of core bacteria that are linearly associated with sire breed composition. **e** Preweaning calves were regrouped into five dam-BGs (D-BGs) based on their dam breed composition. **f** Shannon index among five D-BGs. **g** PCoA plot of weighted UniFrac community distance comparing gut microbiota composition among five D-BGs. **h** The relative abundances of core bacteria that are linearly associated with dam breed composition. **b**, **f** The bars represent mean ± SEM

The calf herd was maintained on the same pasture throughout the length of this study. Although preweaning calves had access to an identical diet, including supplementary feed, the nutrients in milk provided by dams may have differed due to variations in dam breed composition. Therefore, to explore the effects of sire breed composition, which reflect genetic impact only, we regrouped calves based on their sire breed composition to remove any possible effects of milk nutrient variation caused by dam breed composition. Calves were reassigned into four sire breed groups (S-BGs) based on their sire breed composition (Fig. 5a): S-BG1 (calves bred by sires belonging to BG1), S-BG2&3 (calves bred by sires belonging to BG2 and BG3), S-BG4&5 (calves bred by sires belonging to BG4 and BG5), and S-BG6 (calves bred by sires belonging to BG6). To minimize genetic variation caused by dams, calves bred from dams belonging to BG1 or BG6 were excluded from the analysis that resulted in balanced dam breed composition among sire progeny. Therefore, the variation in breed composition among preweaning calves from S-BGs would be primarily due to the differences in sire breed composition (Supplementary Fig. 4A–B), while breed composition of dams among S-BGs were similar (Supplementary Fig. 4C). The alpha diversity showed no significant difference among four S-BGs (Fig. 5b), with similar age range (Supplementary Fig. 4D) and gender (Supplementary Fig. 4E). However, the PCoA plot (Fig. 5c) showed that the gut microbiota structure was significantly different among four S-BGs (*P* = 0.039). A multiple linear regression model including the independent variables of calf age, gender, proportion of Brahman sire, and proportion of Brahman dam, and the dependent variable of relative abundances of core bacteria, indicated that ~30–45% of classified core bacteria were linearly associated with the Brahman proportion of the sire (Fig. 5d, Supplementary Table S5). In addition, the relative abundance of most bacteria that were linearly associated with the Brahman proportion in six calf BGs were also associated with the Brahman proportion in calves of four S-BGs. These bacteria include fiber-digesting bacteria (*Coprococcus, Blautia, Faecalibacterium*, and *Butyrivibrio*), and pathogenic bacteria (*Clostridium*, *Campylobacter*, and Enterobacteriaceae), as well as mucin-degrading Verrucomicrobia and Rikenellaceae. We also evaluated the effects of sire breed composition on animal phenotypes including weight gain and glucose, NFEA, and IgG1 levels, in four S-BGs but only IgG1 level was tended to show negative association with sire Brahman proportion (Supplementary Table S5, Supplementary Fig. 5A–D).

We then explored the effects of dam breed composition on the gut microbiota of the early stage of calves by regrouping calves based on the breed composition of dams (Fig. 5e). To balance sire breed composition of dam progeny, calves bred from sires belonging to BG1 and BG6 were removed, and calves belonging to BG5 and BG6 were combined into one group. Therefore, the variation in breed composition of preweaning calves among five dam breed groups (D-BGs) would be primarily due to the differences in dam breed composition (Supplementary Fig. 6A–C). Surprisingly, unlike dramatic variation in gut microbiota among four S-BGs, the bacterial diversity (Fig. 5f) and microbiota structure (Fig. 5g) among five D-BGs were not significantly different. Only five bacterial families/genera were identified to be significantly linearly associated with dam breed composition based on a multiple linear regression model (Fig. 5h, Supplementary Table S6). However, dam breed composition was significantly associated with animal growth (Supplementary Table S6, *P* = 0.0002), with calves bred from dams in BG1 having greater weight gain compared with those bred from dams in BG6 (Supplementary Fig. 5E). But no significant association was observed between dams’ breed composition with plasma glucose, NEFA, and IgG1 concentrations (Supplementary Table S6, Supplementary Fig. 5F–H, *P* > 0.10). Taken together, the breed composition of sires profoundly affected the structure of the early gut microbiota in their progeny, whereas dams’ breed composition did not significantly affect the gut microbiota.

### SNP genotypes in mucin-encoding genes are associated with breed composition and mucin-degrading bacteria

**Fig. 6:**
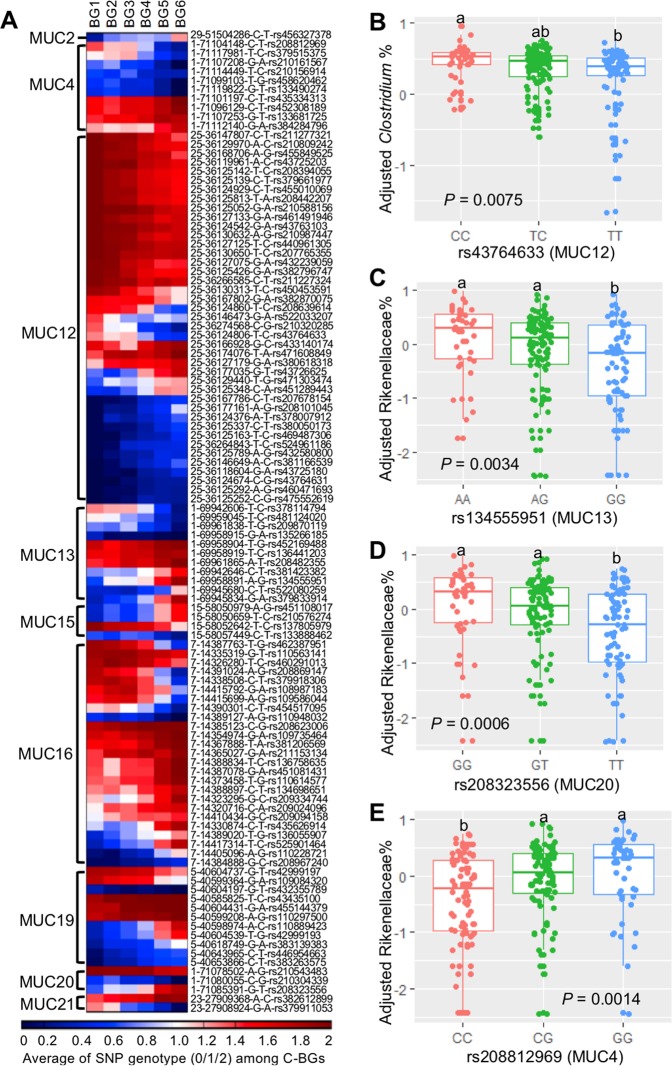
Genotypes of SNP located in or near mucin-encoding genes are associated with breed composition and abundance of mucin-degrading bacteria. **a** The heatmap represents the linear change of SNP genotypes located the in mucin-coding genes with breed composition. **b** The log_10_ transformed relative abundance of *Clostridium* in preweaning calves who have different genotypes at rs43764633 (MUC12). **c**–**e** The log_10_ transformed relative abundance of Rikenellaceae in preweaning calves who have different genotypes at rs134555951 (MUC13), rs208323556 (MUC20), and rs208812969 (MUC4). In each dotplot, values that do not have a common superscript are significantly different (*P* < 0.05) based on one-way ANOVA followed by Tukey’s HSD test for pairwise comparison of multiple means

As several mucin-degrading bacteria were linearly associated with breed composition, we hypothesized that variations in the genotype of mucin-encoding genes among the calves might contribute to the differences in the relative abundance of mucin-degrading bacteria. Among the 327 SNP markers located in or near mucin-encoding genes identified by genotyping, 173 had known rs ID, call rate higher than 90% and minor allele frequency higher than 1%, and were used for downstream analysis. Spearman rank correlation coefficients indicated that genotypes of 108 SNP markers located in or near the mucin-encoding genes were significantly correlated with breed composition (Supplementary Table S7, Fig. 6a, *r*_s_ > 0.20 or *r*_s_ < −0.20, *P*_adjust_ < 0.005).

To further explore the connection between SNP genotypes in or near mucin-encoding genes and mucin-degrading bacteria, we used a multiple linear regression model including the genotype of 152 breed-associated SNPs, Brahman proportion, age in days and gender as four independent variables, and the relative abundance of three mucin-degrading bacteria that are enriched in calves with more Angus proportion (*Clostridium*, Rikenellaceae, and *Akkermansi*a) as a dependent variable. We identified 34 significant linear associations between SNP genotype in mucin-encoding genes and the relative abundance of mucin-degrading bacteria (Supplementary Table S8). In Fig. 6b–e, we show difference in relative abundance of mucin-degrading bacteria among the genotypes of four SNPs that had strongest association with bacteria. The SNPs were located in MUC12 (rs43764633), MUC13 (rs134555951), MUC20 (rs208323556), and MUC4 (rs208812969) that are expressed in the bovine GI tract [48].

### Influence of host genetics on microbe–microbe interactions

**Fig. 7:**
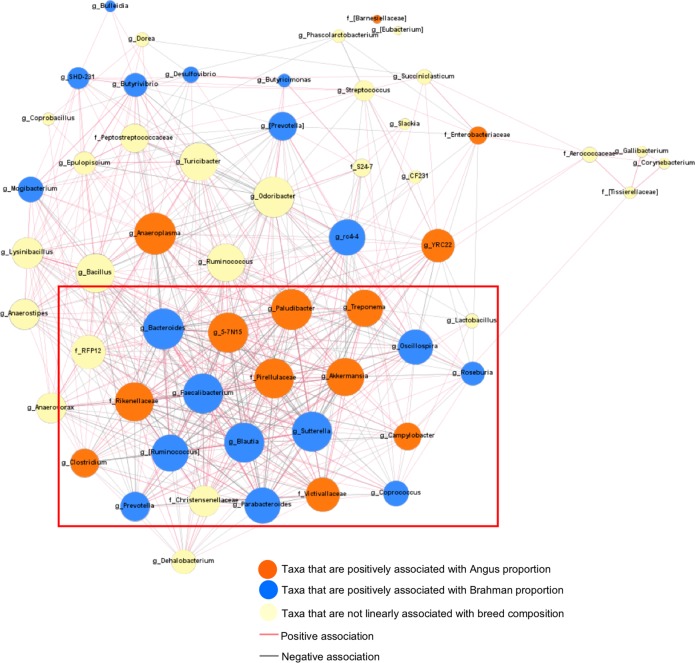
Co-occurrence bacterial network shows 550 significant connections (*P*_adjust_ < 0.05, *r*_s_ > 0.2, or *r*_s_ < −0.2) among 57 bacteria families or genera (identified in at least 50% of 228 calves) in preweaning calves. Connections were detected based on Spearman’s rank correlation coefficient. Dot size represents the number of connections with other taxa. Thickness of lines represent the strength of the relatedness. Genera associated with breed composition are presented in green box

To expand our analysis beyond the selected host–microbe interaction, we analyzed bacteria–bacteria interactions using a co-occurrence network analysis. This analysis was conducted on 57 classified core bacterial families or genera that were present in over 50% of the fecal samples. A total of 550 connections had significant Spearman rank correlations (*P*_adjust_ < 0.05, *r*_s_ > 0.2 or *r*_s_ < −0.2) among microbes (Fig. 7). The strongest positive correlation was detected between *Bacillus* and *Lysinibacillus* (*r*_s_ = 0.8043), while the strongest negative correlation was between 5*-7N15* and *Bacteroides* (*r*_s_ = −0.802). Especially, 21, 31, and 32 correlations were detected between mucin-degrading bacteria *Clostridium*, *Akkermansia*, and Rikenellaceae with other bacteria, respectively, including negative correlations with butyrate-producing bacteria *Blautia*, *Faecalibacterium*, and *Coprococcus,* as well as positive correlations with opportunistic pathogen *Campylobacter*.

### Potential contributions of gut microbiota on calves

**Fig. 8:**
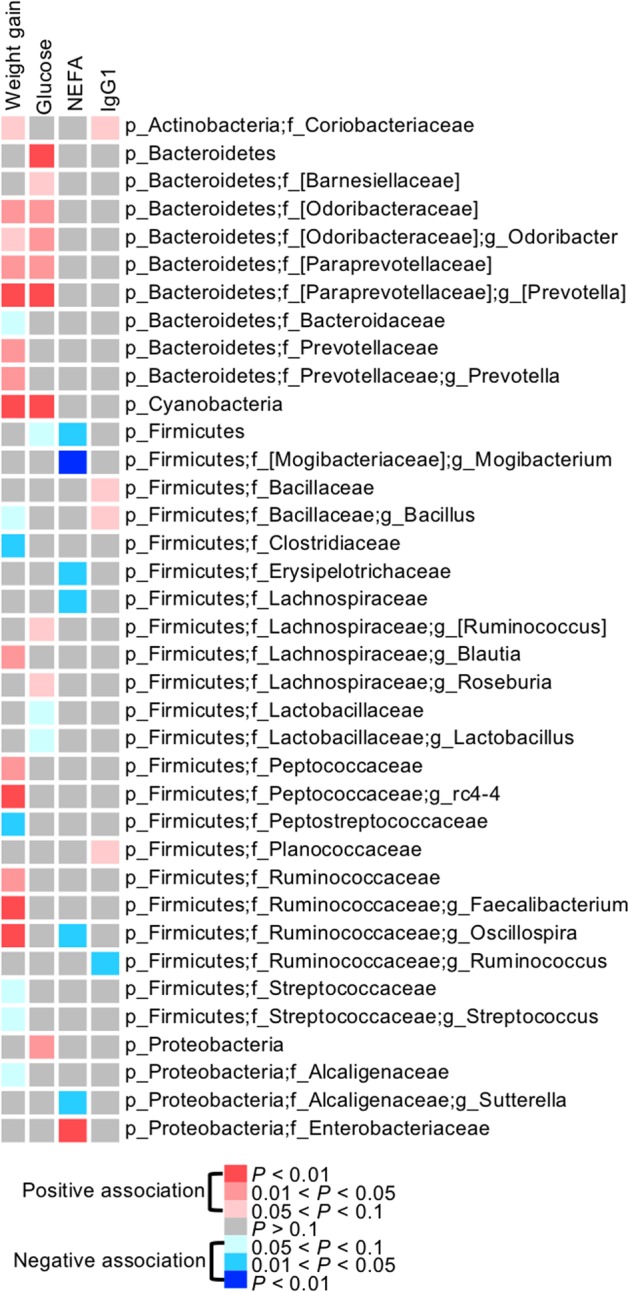
A heatmap shows association between the relative abundance of core bacteria and weight gain, plasma glucose, NEFA, and IgG1 concentrations analyzed by a multiple linear regression model

To further explore the potential impact of gut microbiota on the MAB herd, we analyzed the associations between the relative abundance of core bacteria and animal growth, metabolic parameters, or immune parameters using the multiple linear regression model. The relative abundance of bacteria served as an explanatory variable along with age, Brahman proportion, and gender. Numerous bacteria, including butyrate-producing bacteria *Faecalibacterium*, *Oscillospira*, *Blautia* that were more abundant in calves with more Brahman proportion were positively associated with weight gain (Fig. 8). Negative associations were detected between weight gain and the relative abundance of Bacteroidaceae, Peptostreptococcaceae, Clostridiaceae, *Bacillus*, and *Streptococcus*, likely containing opportunistic pathogenic bacteria (Fig. 8). In addition, we identified several positive associations between Firmicutes bacteria and plasma glucose level, and negative associations between Bacteroidetes bacteria and plasma NEFA level. However, fewer and weaker associations were detected between the relative abundance of bacteria and plasma IgG1 level (Fig. 8).

## Discussion

To understand the impact of host genetics on the gut microbiota structure, we bred a very unique animal model with a linear change of breed composition and raised them in the same environmental conditions with the same diets to minimize nongenetic influences. By using this animal model, we observed effects of host genetics on shaping the gut microbiota structure.

The influence of host genetics on gut microbiota was proposed in the early 1980s by observing more similar fecal microbiota shared between five pairs of monozygotic twins than between dizygotic twins using a culture method [49]. Later, with the development of next-generation sequencing and larger sample sizes of twin studies, the role of host genetics in the gut microbiota structure was confirmed, followed by detection of associations between host SNPs and bacterial abundance [9, 14, 16]. However, the level of strength of host genetic impact on the gut microbiota has not reached consensus yet, primarily due to the different populations used in the studies, with variations in genetic distance, as well as other factors such as age, diet, and living style [17, 18, 50–52]. These factors might have masked the significance of the host genetic role in the gut microbiota in previous studies, leading to a failure in the identification of repeatable associations between host SNPs and bacterial abundance.

In this study, we found core microbial genera among MAB calves that were linearly influenced by breed composition and consistent with measured phenotypes. Calves with more Brahman proportion harbored less mucin-degrading bacteria and opportunistic pathogenic bacteria, but more butyrate-producing bacteria, which are commonly considered as beneficial commensal bacteria primarily due to their antiinflammatory property. Surprisingly, when we regrouped calves based on sires’ breed composition to minimize the maternal effects (e.g., milk composition, companionship), we found that sire genetics had significant impacts in the early gut microbiota structure. However, this same effect was not observed when the calves were regrouped based on their D-BGs. Therefore, consistent associations between the relative abundances of many bacteria in calf BGs and S-BGs indicate that host genetics, influenced heavily by their sires, affect the formation of early gut microbiota structure at the age of 3 months. Previous studies have revealed the effect of sire breed on the rumen microbial populations of beef cattle in feedlot, which is associated with rumen fermentation and methane emissions [12, 53]. This study reports the strong sire breed effect on hindgut microbiota and growth performance of beef cattle during early stage of life. Further research is needed to determine how long this strong effect continues into later developmental stages, as well as how low abundant bacterial taxa, neglected in this study but could be biologically important, may be influenced by host genetics.

The lesser effect of the dam breed composition on the calves’ GI tract microbiota development was quite surprising. Although the relative abundance of several bacteria showed a linear association with dam breed composition, the number was significantly lower compared with that affected by sire breed composition. However, it is noteworthy that dam breed effects may be much more complex. Although we regrouped calves to minimize the maternal effects driven by host genetics, other maternal effects such as milk composition and companionship might have masked potentially undetected genetic effects. Furthermore, genetic relationships among calves in D-BGs could be more complicated compared with that of calves in S-BGs because more dams were used for the mating that might have resulted in higher genetic variability among calves within D-BGs.

One of the primary functions of the gut microbiota is protecting the gut lining from pathogen colonization. Interestingly, among the bacteria that were linearly associated with breed composition, pathogenic bacteria *C. perfringens* and *Campylobacter* were less abundant in calves with more Brahman proportion. *C. perfringens* and *Campylobacter* are both identified as causes of enteric diseases in young calves due to toxin production [54–56]. Moreover, we also observed that the relative abundance of several mucin-degrading bacteria reduced as Brahman proportion decreased. An increase in mucin-degrading bacteria was reported to be positively associated with pathogen colonization due to the consumption of mucin, which is a crucial component of the intestinal epithelial barrier [47, 57–59]. As expected, positive associations between mucin-degrading bacteria and pathogenic bacteria were detected in our study. We further observed a linear association between SNP genotypes located in or near mucin-encoding genes and breed composition, as well as strong correlations between mucin-degrading bacteria (*Clostridium*, Rikenellaceae, and *Akkermansi*a) and SNPs markers located in mucin-coding genes (MUC4, MUC12, MUC13, and MUC20), which shed light on variation of mucin-degrading bacteria in the MAB herd. Transcription of the four mucin genes has been detected in the GI tract of cattle, including the large intestine [48]. In human studies, membrane-bound MUC4 protects colonic epithelium [60]. MUC12 and MUC20 are involved in the epithelial cell protection [61], and the significant downregulation of MUC12 and MUC20 in the colon and ileum has been detected in patients with Crohn’s disease [62]. MUC13 has been reported to be highly expressed on the human intestinal mucosal surface, and polymorphism in MUC13 is related to inflammatory bowel disease [63]. Therefore, the polymorphisms in cattle mucin genes probably contribute to variation in host defense systems among the calves with different breed compositions and result in the distinction of the gut microbiota according to their genetic background.

In this study, we primarily focused on understanding the impact of the gut microbiota in the early preweaning stage in which the rumen is not fully developed yet [64], and consequently, the hindgut is critically vital for energy harvest [65]. In addition, at this stage, animals grow faster and immunity starts to develop, and the GI tract microbiota is more diverse than late growth stage [66]. Based on this evidence, we hypothesized that host genetics probably exert a stronger impact on early gut microbiota that further contributes to animal development. Compared with Angus cattle, Brahman cattle have a greater ability to utilize low-quality feeds that contain low concentrations of protein and soluble carbohydrates [23]. Conversely, Angus cattle grow more quickly, and their meat contains more marbling than Brahman [67]. We found that fiber-digesting bacteria *F. prausnitzii*, *Blautia producta, Oscillospira*, and *Coprococcus*, considered as beneficial commensal gut bacteria in calf and humans due to their production of butyrate [68–71], increased linearly as Brahman proportion increased. Notably, butyrate-producing bacteria more abundant in calves with more Brahman proportion showed positive associations with weight gain, indicating their contribution on energy harvest during the early stage of calves. Our results also revealed variations in metabolic status among MAB calves reflected in weight gain, plasma glucose, and NEFA concentrations. As predicted by PICRUSt, which has high agreement with metagenomic sequencing data [35], the relative abundances of microbial genes involved in carbohydrate metabolic pathways linearly increased as Brahman proportion increased, while those participating in amino acid and lipid metabolic pathways increased as Angus proportion increased, suggesting that breed composition influenced nutrient environment provided to the gut commensal bacteria.

We also found that the level of plasma IgG1, which is the primary antibody in the circulation system, decreased linearly as calves’ and sires’ Brahman proportion increased, indicating a linked effect of host genetics on systemic immunity. IgG1 is induced when the infectious disease is manifest to protect the host by binding itself to pathogens [72]. Therefore, calves with greater Brahman proportion potentially had lower IgG1 because they harbored less pathogenic bacteria in the GI tract. Although *Bos indicus* is known as a disease resistant breed and widely used in animal breeding in the tropical area, most studies focus on parasite resistance due to their thick skin with few explorations in the distinction of their immune function and its relationship with other infectious diseases [22, 72, 73]. The discovery of variation in genotype of the mucin genes, expression of plasma IgG1, as well as the abundance of pathogenic bacteria in calf BGs suggests that the *Bos indicus* breed may have different innate and adaptive immunity compared with the *Bos taurus* breed that enables them to better resist infectious diseases.

Besides breed composition, we also assessed the effect of age and gender on gut microbiota structure of preweaning calves by using a multiple linear regression model. Consistent with the previous findings that Brahman cattle reach puberty later than Angus cattle [38, 74], we observed that most bacteria that exhibited linear correlations with Brahman proportion had opposite correlations with calf age (Supplementary Table S4), indicating that the gut microbiota of calves with higher Brahman proportion also develops more slowly than that of calves with higher Angus proportion. Sex of the calf did not significantly influence the gut microbiota structure in this study (Supplementary Fig. 7, *P* = 0.830), but was associated with the relative abundance of several bacteria (Supplementary Table S4). The relative abundance of Bacteroidetes and [Paraprevotellaceae], which was associated with plasma glucose level, was higher in heifers, suggesting that gender could partly affect calf growth and metabolic status through changes in microbiota. This is consistent with the previous findings that bacteria associated with sex were involved in numerous carbohydrate and lipid metabolic pathways [75, 76].

Furthermore, we found that breed-associated bacteria, such as *Anaeroplasma*, *Paludibacter*, *Bacteroides*, *5-7N15*, Pirellulaceae, interacted with many other bacteria, suggesting that the relative abundance of these bacteria were highly dependent on other bacteria in the GI tract. Besides, various negative connections were detected among Angus-associated bacteria and Brahman-associated bacteria, especially among butyrate-producing bacteria (*Faecalibacterium*, *Blautia*, *Oscillospira*) and mucin-degrading bacteria (*Clostridium*, Rikenellaceae, and *Akkermansia*). These data are consistent with the previous study reporting the competition between fiber-digesting bacteria and mucin-degrading bacteria [47]. Therefore, the host genotype may directly affect on colonization of certain bacteria and indirectly shape the gut microbiota structure through the bacteria–bacteria interactions.

In summary, we found that host genetics, especially from the sire, significantly contribute to the structure of a calf’s gut microbiota at age 3 months old. Furthermore, we identified host SNPs that were associated with specific bacterial genera involved in the gut health and nutritional acquisition. Further studies to understand additional factors beyond host genetics, age, and gender are needed to distinguish the factors that affect development of the bovine microbiota structure. Ultimately, understanding mechanisms to develop and maintain the gut microbiota homeostasis will be necessary for a sustainable agricultural production system. These results also have implications for studying the complex suite of factors that lead to human metabolic syndromes and intestinal disorders.

## Supplementary information

Supplementary Information

Supplementary Table S1. Animal information of breed composition, age, and gender

Supplementary Table S2. Sequencing information

Supplementary Table S3. Correlation between Brahman proportion and OTU prevalence

Supplementary Table S4. Influences of age, Brahman proportion and gender on growth, plasma parameters, and gut microbiota of MAB1 preweaning calves reflected from the multiple linear regression model

Supplementary Table S5. Influences of age, sires' and dams' Brahman proportion, and gender on growth, plasma parameters, and gut microbiota of regrouped MAB1 preweaning calves based on sires' breed co

Supplementary Table S6. Influences of age, sires' and dams' Brahman proportion, and gender on growth, plasma parameters, and gut microbiota of regrouped MAB1 preweaning calves based on dams' breed com

Supplementary Table S7. Correlation between Brahman proportion and genotypes of SNPs that are located in or near mucin-encoding genes

Supplementary Table S8. Associations between the genotypes of SNPs located in mucin-encoding genes of MAB1 preweaning calves and log10 transformed relative abundance of mucin-degrading bacteria reveal

## Data Availability

The V4 region of 16 S rRNA gene sequencing data generated and analyzed during the current study are available in the NCBI primary data archive (PDA) with the accession number SRP115548.
