# Supplementary material for: Host genetic effects upon the early gut microbiota in a bovine model with graduated spectrum of genetic variation
Source: ISME J. 2019 Oct 17;14(1):302–17. doi: 10.1038/s41396-019-0529-2 (PMC6908690; doi:10.1038/s41396-019-0529-2)
Supplement: Supplementary file 1 — Supplementary Information [file 41396_2019_529_MOESM1_ESM.docx]

Supplementary information for

**Host genetic effects upon the early gut microbiota in a bovine model**

**with graduated spectrum of genetic variation**

Peixin Fan^1, 2^, Beilei Bian^2^, Lin Teng^1, 2^, Corwin D. Nelson^2^, J. Danny Driver^2^,

Mauricio A. Elzo^2^, and Kwangcheol C. Jeong^1, 2*^

^1^Emerging Pathogens Institute, University of Florida, Gainesville, FL 32611, USA

^2^Department of Animal Sciences, Institute of Food and Agricultural Sciences, University of Florida, Gainesville, FL 32611, USA

Running title: Host genetics on gut microbiota

Keywords: Microbiota, Host genetics, Single nucleotide polymorphisms

*****Corresponding author

K.C. Jeong, PhD

Associate Professor of Microbiology

Address: 2055 Mowry Rd, Gainesville, FL USA 32611

Email:kcjeong@ufl.edu

Phone: 1-352-294-5376

**Supplementary Methods**

*Microbial community analysis*

Raw sequencing reads were obtained from the Illumina BaseSpace website and analyzed with the Quantitative Insights into Microbial Ecology (QIIME) pipeline (version 1.9.0). Paired-end reads were joined with the scripts multiple_join_paired_ends.py. The unjoined reads were discarded for further analysis. Demultiplexing and quality filtering (at Phred >= Q20) was performed with the script multiple_split_libraries_fastq.py. Chimeric sequences were identified using usearch61 and removed from subsequent analysis with the script identify_chimeric_seqs.py. The open-reference OTU picking was conducted via UCLUST to cluster similar sequences sharing ≥ 99% similarity and taxonomy was assigned based on Silva 132 database (https://www.arb-silva.de/documentation/release-132/) with the script pick_open_reference_otus.py. The OTU table was generated with the script make_otu_table.py. The sequencing data were normalized to 13650 (13656 was the lowest sequencing depth among samples) with the script single_rarefaction.py. Chao and Shannon index produced as alpha diversity was analyzed with the script: alpha_diversity.py. Weighted UniFrac distance produced as beta-diversity measures and then subjected to principal coordinates analysis (PCoA) with the script: beta_diversity_through_plots.py. Analysis of similarities (ANOSIM) was used to detect the statistical difference of UniFrac distance metric with the script compare_categories.py. The relative abundance of bacterial taxa at six-level taxonomic classification (phylum, class, order, family, genus and species) was obtained with the script: summarize_taxa_through_plots.py.

*Quantitative real-time PCR (qPCR) analysis*

The qPCR was conducted to confirm the differences in the relative abundance of *Faecalibacterium prausnitzii* and *Clostridium perfringens* between BG1 and BG6 using the primer sets as follows. Total bacteria: KCP918, 5’- GTGSTGCAYGGYTGTCGTCA-3’ and KCP919, 5’-ACGTCRTCCMCACCTTCCTC-3’; *F. prausnitzii*: KCP906, 5’-CCATGAATTGCCTTCAAAACTGTT-3’ and KCP 907, 5’-GAGCCTCAGCGTCAGTTGGT-3’; *C. perfringens*: KCP916, 5’-ATGCAAGTCGAGCGAKG-3’ and KCP917, 5’-TATGCGGTATTAATCTYCCTTT-3’. The quantification of DNA for each bacterial species in fecal samples was performed with CFX96 Real-Time PCR System (Biorad, USA) using the SsoAdvanced Universal SYBR Green Supermix (Biorad, USA). Each diluted standard sample was assayed in triplicate in a 10 µl reaction containing 1 µl 10 ng genomic DNA template, 0.5 µl 10 pmol/µl each primer, and 5 µl SYBR Green Supermix. Amplification involved one cycle at 98°C for 3 min for polymerase activation and DNA denaturation, and then 40 cycles of 98°C for 15 s followed by annealing/extension at 60°C for 15 s. A melting curve of PCR products was monitored by slow heating with an increment of 0.5°C for 5 s from 65 to 95°C, with fluorescence collection at 0.5°C intervals. The standard curve was generated according to Li et al.’s [1] method with a minor modification. First, universal bacterial primer set, 63F-1387R (KCP812:forward-5’-CAGGCCTAACACATGCAAGTC-3’ and KCP813: reverse-5’-GGGCGG WGTGTACAAGGC-3’) was used to amplify the 16S rRNA gene using DNA template from one calf fecal sample. The 16S rRNA gene PCR product was purified using Wizard SV Gel and PCR Clean-Up System (Promega, USA). Ten-fold serial dilution of purified 16S rRNA gene PCR product was used as DNA template to conduct standard curve for quantification of total bacteria, *F. prausnitzii* and *C. perfringens* to confirm the efficiency of the primers. The differences in copy numbers of *F. prausnitzii* and *C. perfringens* between BG1 and BG6 were compared.

**References**

1. Li M, Penner GB, Hernandez-Sanabria E, Oba M, Guan LL. Effects of sampling location and time, and host animal on assessment of bacterial diversity and fermentation parameters in the bovine rumen. J Appl Microbiol 2009; 107: 1924-1934.

**Supplementary figures**

**
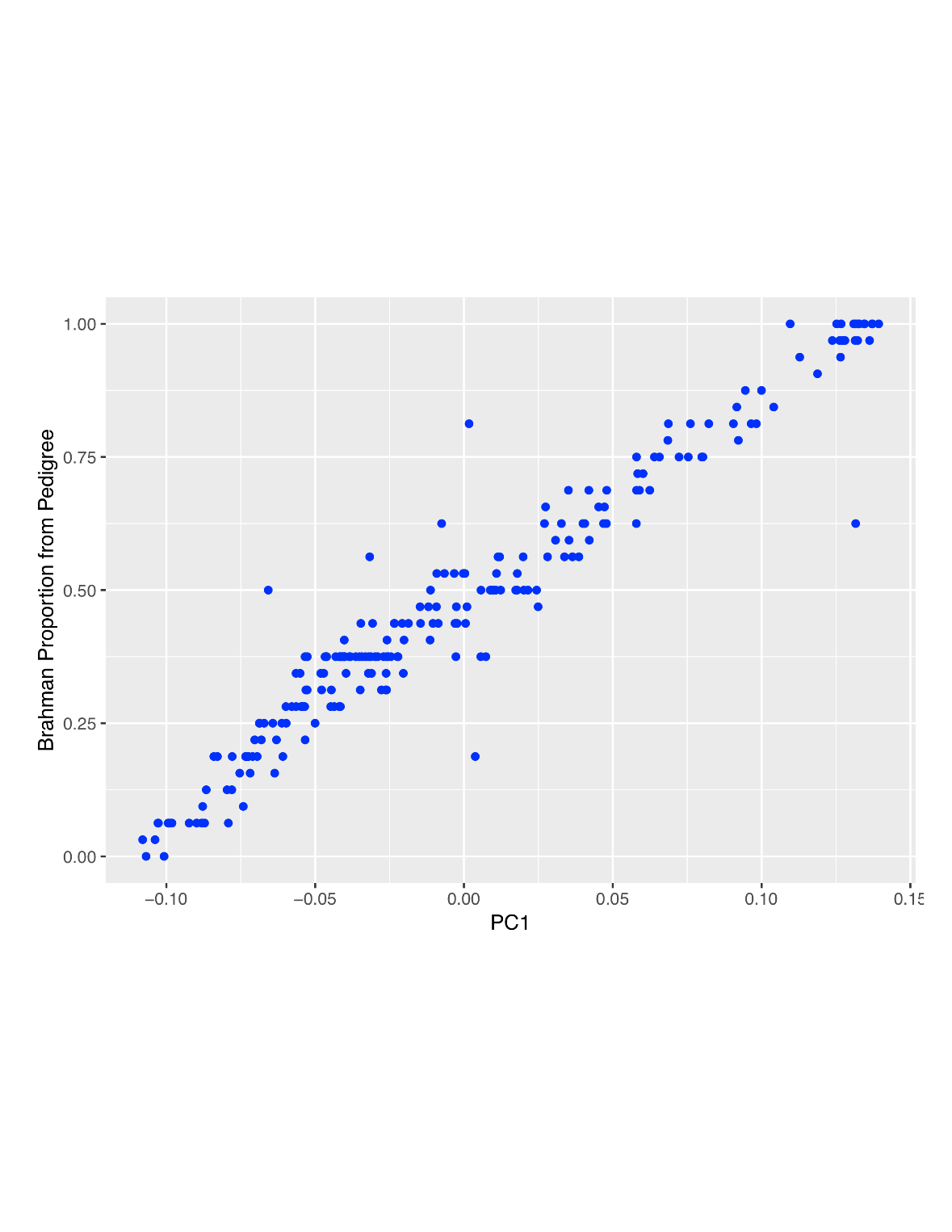
**

**Supplementary Figure 1. Relationship between the Brahman proportion estimated by pedigree and first principal component (PC1) generated from principal component analysis (PCA) using the SNP genotyping data (R = 0.97, P = 2.2×10^-16^).**

**
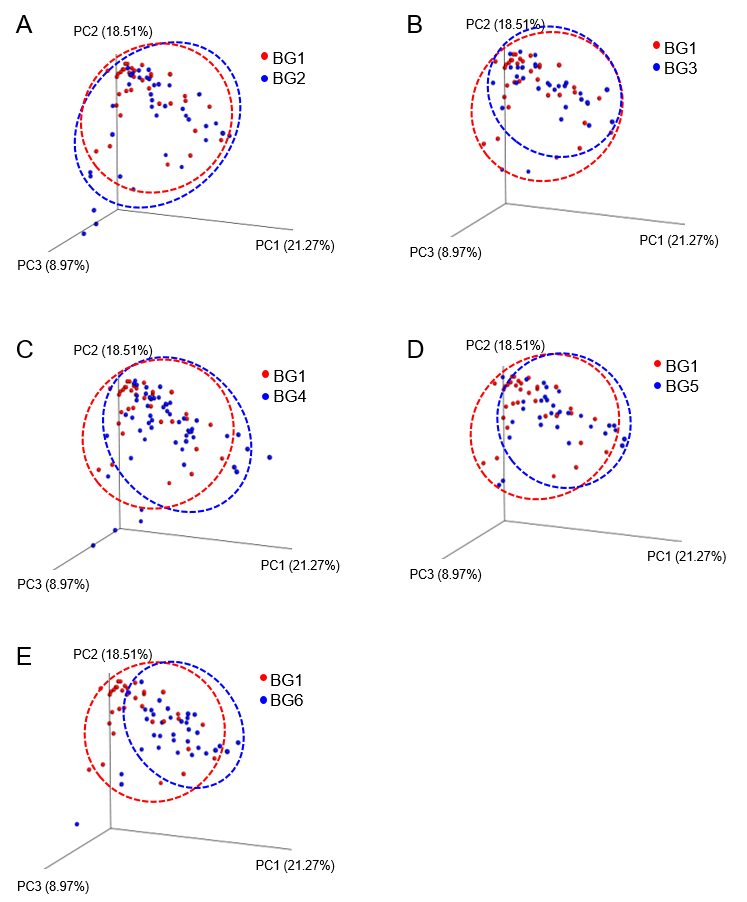
**

**Supplementary Figure 2. PCoA plot of weighted UniFrac community distance comparing gut microbiota structure between BG1 with other BGs.** (A) PCoA plot of BG1 and BG2. (B) PCoA plot of BG1 and BG3. (C) PCoA plot of BG1 and BG4. (D) PCoA plot of BG1 and BG5. (E) PCoA plot of BG1 and BG6.


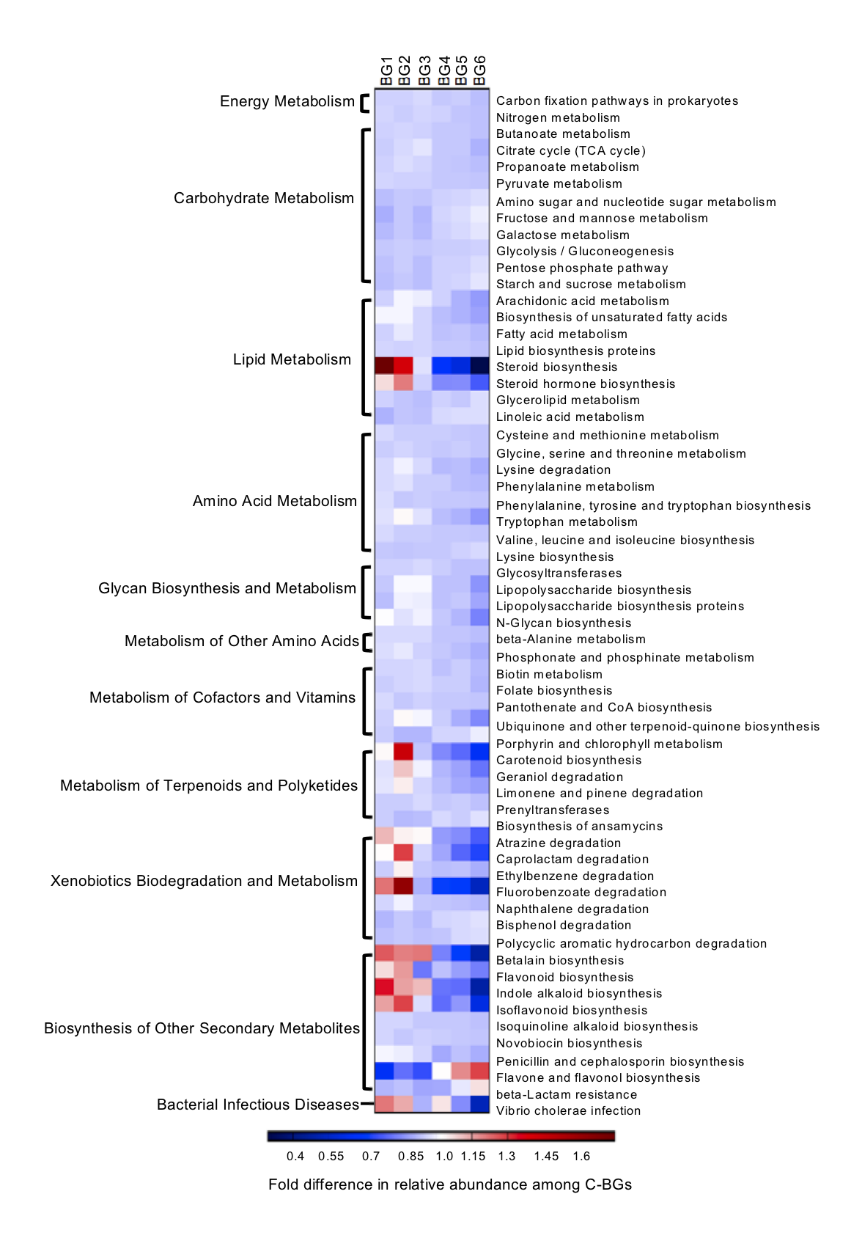


**Supplementary Figure 3. A heatmap shows the fold differences in the relative abundances of the predicted functional genes involved in KEGG pathways among 6 BGs.** Only KEGG pathways with their relative abundance significantly correlated with breed composition were involved in the heatmap.


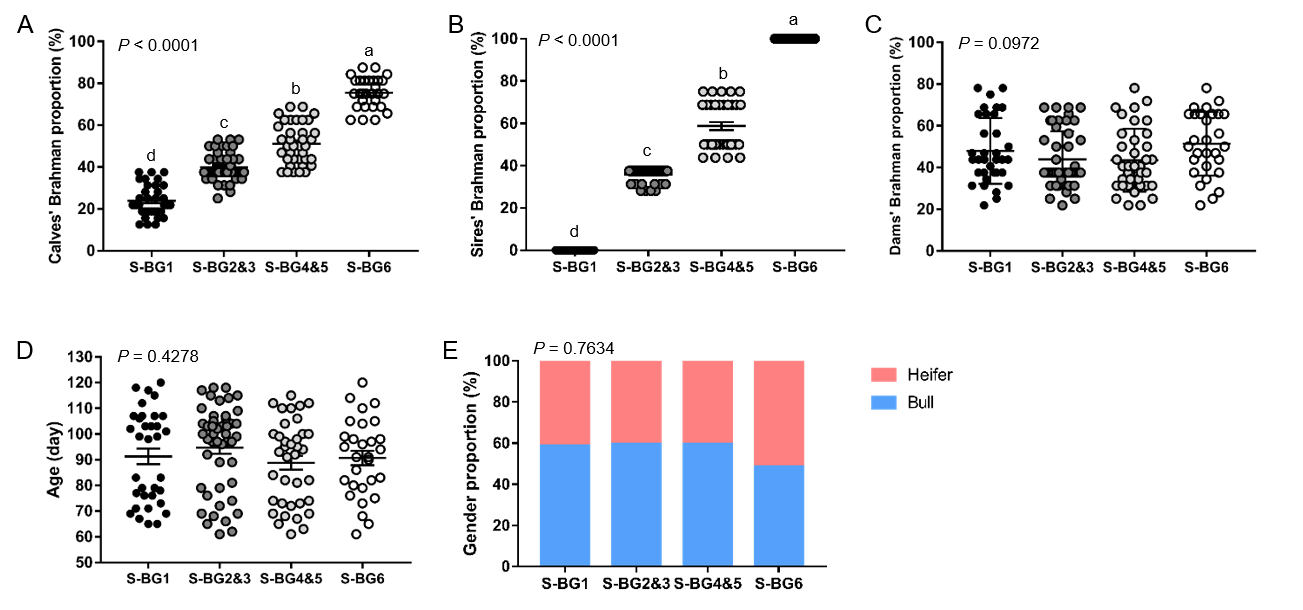


**Supplementary Figure 4.** **Variation in breed composition, age and gender across sire breed groups (S-BGs).** Brahman proportion of preweaning calves (A), as well as Brahman proportion of their sires (B) increase from sire breed group 1 (S-BG1) to S-BG6, while Brahman proportion of their dams (C), age (D) and gender distribution (E) were almost identical among S-BGs. In each dotplot or barplot, values that do not have a common superscript are significantly different (*P* < 0.05) based on one-way ANOVA followed by Tukey’s HSD test for pairwise comparison of multiple means. In (A-D), the bars represent mean ± SEM.


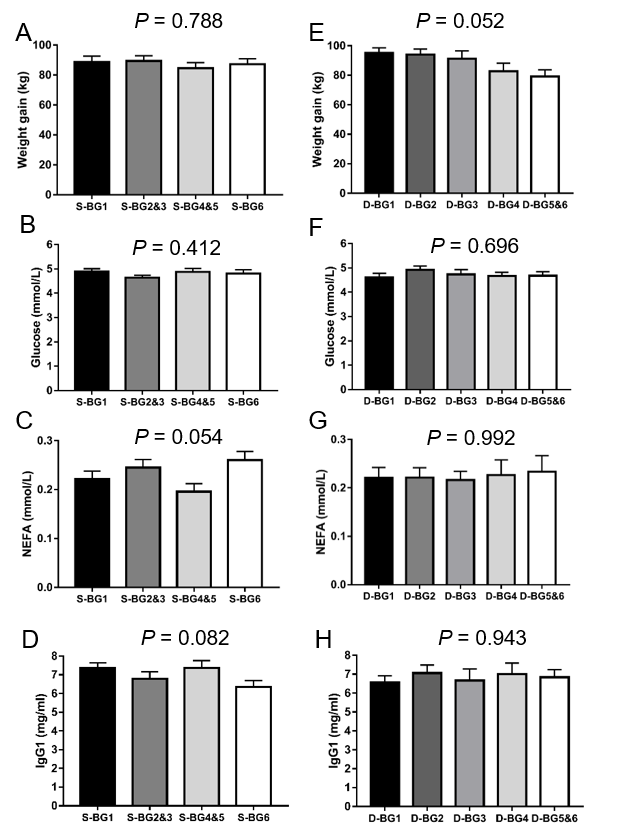


**Supplementary Figure 5. Animal growth, metabolic and immune status of preweaning calves among different S-BGs and D-BGs.** (A-D) Weight gain, plasma glucose, NEFA and IgG1 levels across 4 sire-BGs. (E-H) Weight gain, plasma glucose, NEFA and IgG1 levels across 5 dam-BGs. Data are shown as mean ± SEM. Differences among breed groups were analyzed with one-way ANOVA.


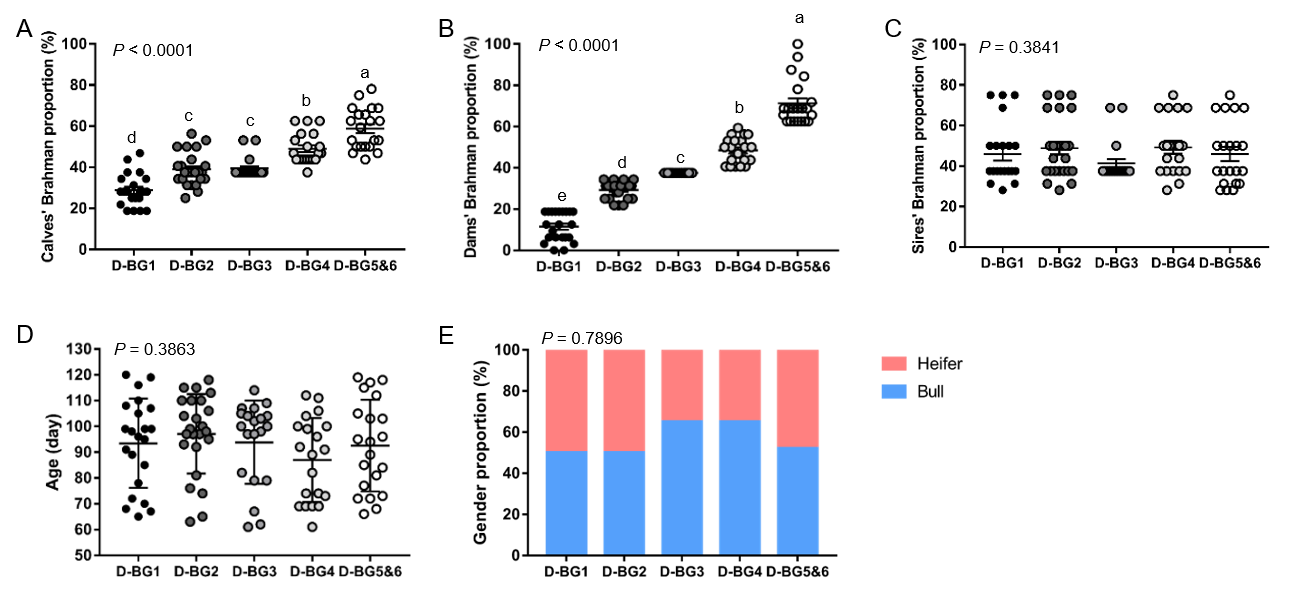


**Supplementary Figure 6. Variation in breed composition, age and gender across dam breed groups (D-BGs).** Brahman proportion of preweaning calves (A), as well as Brahman proportion of their dams (B) increase from dam breed group 1 (D-BG1) to D-BG5&6, while Brahman proportion of their sires (C), age (D) and gender distribution (E) are almost identical among D-BGs. In each dotplot or barplot, values that do not have a common superscript are significantly different (*P* < 0.05) based on one-way ANOVA followed by Tukey’s HSD test for pairwise comparison of multiple means. In (A-D), the bars represent mean ± SEM.


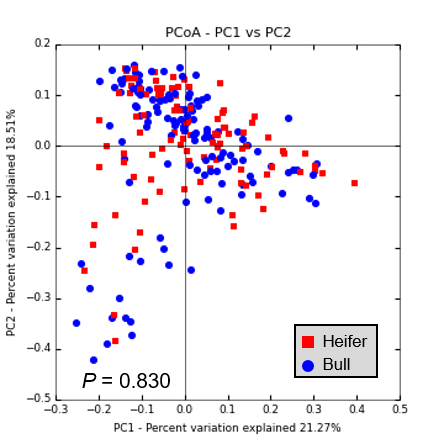


**Supplementary Figure 7. PCoA plot of weighted UniFrac community distance comparing gut microbiota structure between heifers and bulls.**
