# Supplementary material for: Host genetic effects upon the early gut microbiota in a bovine model with graduated spectrum of genetic variation
Source: ISME J. 2019 Oct 17;14(1):302–17. doi: 10.1038/s41396-019-0529-2 (PMC6908690; doi:10.1038/s41396-019-0529-2)
Supplement: Supplementary file 2 — Supplementary Table S1. Animal information of breed composition, age, and gender [file 41396_2019_529_MOESM2_ESM.pdf]

Supplementary Table S1. Animal information of breed composition, age, and gender.

Calf ID	Sample ID	Sequencing ID	Age (day)	Sex (1, bull; 2, heifer)	Calf BG[1]	Calf Brahman proportion[2]	Sire ID	Sire BG	Sire Brahman proportion	Dam ID	Dam BG	Dam Brahman proportion
3160093	KCS6124	KM581	99	1	2	31.3%	3999293	3	37.5%	3100160	2	25.0%
3160108	KCS6125	KM582	98	1	2	31.3%	1109074	1	0.0%	3050170	5	65.6%
3160134	KCS6126	KM583	94	1	5	75.0%	3999307	6	100.0%	3100057	4	46.9%
3160008	KCS6127	KM584	120	1	5	71.9%	3999307	6	100.0%	3130234	4	43.8%
3160049	KCS6128	KM585	107	1	2	21.9%	1109074	1	0.0%	3110093	4	43.8%
3160076	KCS6129	KM586	103	1	1	12.5%	3999270	1	0.0%	3080054	2	25.0%
3160082	KCS6130	KM587	102	2	2	21.9%	3999270	1	0.0%	3110028	4	43.8%
3160077	KCS6131	KM588	103	1	1	9.4%	3999301	1	0.0%	3120063	1	18.8%
3160074	KCS6132	KM589	103	1	2	34.4%	3999293	3	37.5%	3100175	2	31.3%
3160137	KCS6133	KM590	92	2	6	96.9%	2120125	6	100.0%	3120148	6	93.8%
3160073	KCS6134	KM591	103	1	1	6.3%	3999270	1	0.0%	3110066	1	12.5%
3160048	KCS6135	KM592	107	2	1	18.8%	1109074	1	0.0%	3050203	3	37.5%
3160121	KCS6136	KM593	96	1	4	56.3%	3100189	5	68.8%	3120175	4	40.6%
3160127	KCS6137	KM594	95	1	3	37.5%	3070006	4	43.8%	3130169	2	34.4%
3160153	KCS6138	KM595	88	2	6	100.0%	3999306	6	100.0%	3010138	6	100.0%
3160191	KCS6139	KM596	68	1	5	65.6%	3100189	5	68.8%	3120163	5	62.5%
3160129	KCS6140	KM597	95	1	6	100.0%	3999306	6	100.0%	3100117	6	100.0%
3160114	KCS6141	KM598	97	2	6	84.4%	3999307	6	100.0%	3090085	5	68.8%
3160104	KCS6142	KM599	98	1	6	87.5%	3999307	6	100.0%	3110030	5	78.1%
3160128	KCS6143	KM600	95	1	6	96.9%	3999307	6	100.0%	3100107	6	93.8%
3160140	KCS6144	KM601	91	1	6	100.0%	3999296	6	100.0%	3070067	6	96.9%
3160118	KCS6145	KM602	97	1	6	96.9%	3999307	6	100.0%	3120022	6	93.8%
3160106	KCS6146	KM603	98	1	6	100.0%	3999296	6	100.0%	3120143	6	100.0%
3160090	KCS6147	KM604	100	1	4	40.6%	3080063	4	50.0%	3120095	2	31.3%
3160062	KCS6148	KM605	105	1	5	62.5%	3080011	2	28.1%	3120211	6	93.8%
3160072	KCS6149	KM606	103	2	2	25.0%	3999288	1	0.0%	3090016	4	46.9%
3160148	KCS6150	KM607	89	1	4	50.0%	3090067	2	31.3%	3100126	5	65.6%
3160087	KCS6151	KM608	100	1	5	62.5%	3100189	5	68.8%	3090042	4	56.3%
3160084	KCS6152	KM609	101	1	4	59.4%	3999306	6	100.0%	3080149	1	18.8%
3160040	KCS6153	KM610	110	1	2	34.4%	1119033	3	37.5%	3090069	2	31.3%
3160080	KCS6154	KM611	102	1	3	37.5%	3999305	3	37.5%	3050183	3	37.5%
3160113	KCS6155	KM612	97	1	3	37.5%	3070006	4	43.8%	3070019	2	31.3%
3160054	KCS6156	KM613	106	1	1	12.5%	3999287	1	0.0%	3120031	2	28.1%
3160085	KCS6157	KM614	101	1	1	18.8%	3999301	1	0.0%	3110040	3	37.5%
3160050	KCS6158	KM615	107	2	2	25.0%	1119033	3	37.5%	3090038	1	12.5%
3160107	KCS6160	KM617	98	2	3	37.5%	1119033	3	37.5%	3110015	3	37.5%
3160051	KCS6161	KM618	107	1	3	37.5%	3999293	3	37.5%	3110029	3	37.5%
3160067	KCS6162	KM619	104	1	3	37.5%	3999294	3	37.5%	3090074	3	37.5%
3160055	KCS6163	KM620	106	1	4	43.8%	3070006	4	43.8%	3090021	4	40.6%
3160146	KCS6164	KM621	90	1	5	78.1%	3999284	6	100.0%	3060073	4	56.3%
3160025	KCS6165	KM622	115	1	1	6.3%	3999301	1	0.0%	3110194	1	9.4%
3160145	KCS6166	KM623	91	1	2	34.4%	3080077	4	50.0%	3080153	1	18.8%
3160094	KCS6167	KM624	99	2	5	68.8%	3999307	6	100.0%	3050182	3	37.5%
3160135	KCS6168	KM625	93	2	4	53.1%	3999274	5	75.0%	3100072	2	31.3%
3160075	KCS6169	KM626	103	1	4	53.1%	3999293	3	37.5%	3120056	5	68.8%
3160024	KCS6170	KM627	115	2	1	3.1%	3999287	1	0.0%	3110002	1	6.3%
3160122	KCS6171	KM628	96	2	4	53.1%	3999294	3	37.5%	3120122	5	68.8%
3160110	KCS6172	KM629	98	2	4	50.0%	3999296	6	100.0%	3050081	1	0.0%
3160060	KCS6173	KM630	105	1	6	81.3%	3999284	6	100.0%	3120222	5	62.5%
3160143	KCS6174	KM631	91	2	5	75.0%	3999306	6	100.0%	3080185	4	50.0%
3160092	KCS6175	KM632	99	1	1	18.8%	3999287	1	0.0%	3040097	3	37.5%
3160116	KCS6176	KM633	97	2	1	6.3%	3999270	1	0.0%	3120037	1	12.5%
3160052	KCS6177	KM634	107	2	1	18.8%	3999288	1	0.0%	3120101	2	34.4%
3160079	KCS6178	KM635	103	2	4	50.0%	3999305	3	37.5%	3120159	5	62.5%
3160034	KCS6179	KM636	112	1	1	12.5%	3999301	1	0.0%	3110076	2	21.9%
3160099	KCS6180	KM637	99	2	2	31.3%	3080077	4	50.0%	3100218	1	9.4%
3160059	KCS6181	KM638	105	1	3	37.5%	3999293	3	37.5%	3060040	3	37.5%
3160086	KCS6182	KM639	100	2	4	43.8%	3070006	4	43.8%	3120116	4	40.6%
3160097	KCS6183	KM640	99	2	1	0.0%	3999270	1	0.0%	3110086	1	0.0%
3160130	KCS6184	KM641	95	1	4	56.3%	3999307	6	100.0%	3070114	1	12.5%
3160179	KCS6185	KM642	74	1	4	53.1%	3110021	4	50.0%	3120044	4	56.3%
3160100	KCS6186	KM643	99	1	3	37.5%	3080214	5	75.0%	3090210	1	0.0%
3160111	KCS6187	KM644	98	1	6	81.3%	3999306	6	100.0%	3110116	5	65.6%
3160071	KCS6188	KM645	103	2	3	37.5%	3999301	1	0.0%	3120023	5	78.1%
3160131	KCS6189	KM646	96	1	5	68.8%	3999296	6	100.0%	3060064	2	34.4%
3160078	KCS6190	KM647	103	1	3	37.5%	3999294	3	37.5%	3120120	3	37.5%
3160101	KCS6191	KM648	99	1	4	46.9%	3080214	5	75.0%	3090138	1	18.8%
3160112	KCS6192	KM649	97	2	2	25.0%	3090067	2	31.3%	3050032	2	21.9%
3160117	KCS6193	KM650	97	2	2	31.3%	3090067	2	31.3%	3120086	2	31.3%
3160098	KCS6194	KM651	99	2	4	56.3%	2120125	6	100.0%	3100235	1	12.5%
3160105	KCS6195	KM652	98	2	4	43.8%	3100189	5	68.8%	3080048	2	21.9%
3160088	KCS6196	KM653	100	1	3	37.5%	1119033	3	37.5%	3070005	3	37.5%
3160120	KCS6197	KM654	96	1	1	18.8%	3999294	3	37.5%	3100227	1	3.1%
3160109	KCS6198	KM655	98	2	2	25.0%	3090067	2	31.3%	3120017	1	18.8%
3160068	KCS6199	KM656	104	1	4	43.8%	1119033	3	37.5%	3110031	4	53.1%
3160144	KCS6200	KM657	91	2	5	62.5%	3999274	5	75.0%	3110141	4	50.0%
3160053	KCS6201	KM658	106	2	2	31.3%	3999294	3	37.5%	3090104	2	25.0%
3160141	KCS6202	KM659	91	2	5	68.8%	3999307	6	100.0%	3120021	4	40.6%
3160096	KCS6203	KM660	99	1	4	43.8%	3999274	5	75.0%	3090177	1	9.4%
3160133	KCS6204	KM702	94	1	4	59.4%	3080077	4	50.0%	3110049	5	68.8%
3160061	KCS6205	KM703	105	2	2	21.9%	3999294	3	37.5%	3060039	1	6.3%
3160136	KCS6206	KM704	92	1	4	56.3%	3080214	5	75.0%	3090052	2	34.4%
3160115	KCS6207	KM705	97	1	3	37.5%	3999294	3	37.5%	3100185	3	37.5%
3160147	KCS6208	KM706	90	1	4	56.3%	2120125	6	100.0%	3100058	1	12.5%
3160159	KCS6209	KM707	83	1	5	62.5%	3999284	6	100.0%	3050015	2	21.9%

3160066	KCS6210	KM708	104	2	1	6.3%	3999287	1	0.0%	3080010	1	12.5%
3160081	KCS6211	KM709	102	2	1	0.0%	3999270	1	0.0%	3070146	1	3.1%
3160089	KCS6212	KM710	100	2	3	37.5%	3999293	3	37.5%	3120137	3	37.5%
3160083	KCS6213	KM711	101	1	4	50.0%	3999284	6	100.0%	3120028	1	3.1%
3160041	KCS6214	KM712	110	1	2	28.1%	3080063	4	50.0%	3120033	1	6.3%
3160158	KCS6215	KM713	84	1	6	96.9%	3999306	6	100.0%	3120142	6	93.8%
3160157	KCS6216	KM714	84	1	5	68.8%	3999274	5	75.0%	3110118	5	65.6%
3160095	KCS6218	KM715	99	2	2	21.9%	3999287	1	0.0%	3030057	4	43.8%
3160070	KCS6221	KM716	103	1	6	100.0%	2120125	6	100.0%	3130028	6	100.0%
3160171	KCS6222	KM717	79	2	6	81.3%	3999306	6	100.0%	3130198	5	65.6%
3160203	KCS6223	KM718	65	1	1	15.6%	3999301	1	0.0%	3120114	2	31.3%
3160006	KCS6224	KM719	120	2	1	18.8%	3080011	2	28.1%	3130043	1	6.3%
3160011	KCS6226	KM720	118	1	2	28.1%	3080011	2	28.1%	3130235	2	28.1%
3160139	KCS6227	KM721	92	1	1	3.1%	1109074	1	0.0%	3110065	1	6.3%
3160164	KCS6228	KM722	81	1	4	50.0%	1119033	3	37.5%	3110082	5	62.5%
3160064	KCS6229	KM723	104	1	5	68.8%	3999306	6	100.0%	3130113	3	37.5%
3160020	KCS6230	KM724	115	1	4	40.6%	3080063	4	50.0%	3130009	2	31.3%
3160119	KCS6231	KM725	97	1	3	37.5%	3999305	3	37.5%	3130260	3	37.5%
3160037	KCS6232	KM726	110	1	4	50.0%	3999274	5	75.0%	3130184	2	25.0%
3160123	KCS6233	KM727	95	1	5	62.5%	3080077	4	50.0%	3130080	5	71.9%
3160178	KCS6234	KM728	74	2	4	50.0%	1109074	1	0.0%	3130185	6	96.9%
3160125	KCS6235	KM729	95	1	1	6.3%	3999301	1	0.0%	3130109	1	9.4%
3160138	KCS6236	KM730	92	1	4	43.8%	3080011	2	28.1%	3130167	4	56.3%
3160056	KCS6237	KM731	105	2	1	9.4%	3999288	1	0.0%	3130052	1	18.8%
3160200	KCS6238	KM732	67	1	6	90.6%	3129930	6	100.0%	3110127	6	81.3%
3160132	KCS6240	KM734	94	1	1	6.3%	3999270	1	0.0%	3130191	1	15.6%
3160172	KCS6241	KM735	78	1	2	28.1%	3110021	4	50.0%	3110041	1	6.3%
3160154	KCS6243	KM737	86	2	6	81.3%	3129930	6	100.0%	3130273	5	65.6%
3160017	KCS6244	KM738	117	2	4	46.9%	3090067	2	31.3%	3110232	5	62.5%
3160019	KCS6245	KM739	115	2	2	34.4%	1119033	3	37.5%	3130125	2	31.3%
3160031	KCS6246	KM740	112	2	5	62.5%	3110021	4	50.0%	3130066	5	78.1%
3160030	KCS6247	KM741	112	2	5	75.0%	3999306	6	100.0%	3130007	4	46.9%
3160046	KCS6248	KM742	107	2	3	37.5%	3999294	3	37.5%	3130127	3	37.5%
3160181	KCS6249	KM743	74	2	3	37.5%	3110021	4	50.0%	3110019	2	28.1%
3160151	KCS6250	KM744	88	1	6	100.0%	3999307	6	100.0%	3130275	6	96.9%
3160038	KCS6251	KM745	110	1	6	84.4%	3999307	6	100.0%	3130034	5	68.8%
3160013	KCS6252	KM746	118	1	2	31.3%	3999270	1	0.0%	3130089	5	65.6%
3160176	KCS6253	KM747	76	1	5	65.6%	2120125	6	100.0%	3110063	2	31.3%
3160149	KCS6254	KM748	89	1	4	50.0%	3999305	3	37.5%	3130121	4	59.4%
3160009	KCS6255	KM749	119	2	5	75.0%	3080063	4	50.0%	3130133	6	100.0%
3160173	KCS6256	KM750	78	1	2	21.9%	1109074	1	0.0%	3110089	4	43.8%
3160124	KCS6259	KM751	95	2	2	28.1%	3999294	3	37.5%	3130176	1	18.8%
3160065	KCS6260	KM752	104	2	3	37.5%	3999293	3	37.5%	3130220	3	37.5%
3160126	KCS6261	KM753	95	2	6	81.3%	2120125	6	100.0%	3130172	5	65.6%
3160205	KCS6263	KM754	65	1	3	37.5%	1119033	3	37.5%	3060223	3	37.5%
3160170	KCS6264	KM755	79	2	3	37.5%	1119033	3	37.5%	3120100	3	37.5%
3160015	KCS6265	KM756	117	2	1	18.8%	3999287	1	0.0%	3130267	4	40.6%
3160010	KCS6266	KM757	119	2	2	28.1%	1119033	3	37.5%	3130056	1	18.8%
3160160	KCS6267	KM758	83	1	2	21.9%	1109074	1	0.0%	3130119	4	43.8%
3160184	KCS6268	KM759	73	2	3	37.5%	3999301	1	0.0%	3070061	5	75.0%
3160021	KCS6269	KM760	115	1	2	28.1%	3999301	1	0.0%	3130075	4	56.3%
3160063	KCS6270	KM761	104	2	4	40.6%	3110021	4	50.0%	3130200	2	31.3%
3160193	KCS6272	KM762	68	2	1	6.3%	1109074	1	0.0%	3110230	1	9.4%
3160018	KCS6273	KM763	116	2	1	18.8%	3090067	2	31.3%	3130019	1	6.3%
3160043	KCS6275	KM764	109	1	3	37.5%	1119033	3	37.5%	3130202	3	37.5%
3160028	KCS6277	KM766	113	2	2	34.4%	3999293	3	37.5%	3130123	2	31.3%
3160032	KCS6279	KM767	112	2	6	93.8%	3999307	6	100.0%	3130140	6	84.4%
3160187	KCS6280	KM768	70	1	2	28.1%	1119033	3	37.5%	3120145	1	18.8%
3160150	KCS6281	KM769	89	1	1	18.8%	1119033	3	37.5%	3060006	1	0.0%
3160204	KCS6283	KM771	65	1	2	31.3%	3080063	4	50.0%	3090007	1	12.5%
3160188	KCS6284	KM772	69	2	5	62.5%	3100189	5	68.8%	3130022	4	53.1%
3160199	KCS6285	KM773	67	1	4	53.1%	3100189	5	68.8%	3100033	3	37.5%
3160162	KCS6286	KM774	82	2	5	75.0%	3999296	6	100.0%	3130064	4	50.0%
3160163	KCS6287	KM775	82	2	6	87.5%	3999296	6	100.0%	3130205	5	71.9%
3160044	KCS6288	KM776	109	2	4	59.4%	3999307	6	100.0%	3130090	1	18.8%
3160185	KCS6289	KM777	72	2	1	6.3%	3999301	1	0.0%	3090224	1	12.5%
3160026	KCS6292	KM779	114	2	6	81.3%	3999306	6	100.0%	3100140	5	65.6%
3160174	KCS6293	KM780	76	1	2	31.3%	1109074	1	0.0%	3100039	5	65.6%
3160182	KCS6294	KM781	73	2	6	93.8%	3129930	6	100.0%	3100017	6	84.4%
3160168	KCS6296	KM782	79	1	2	28.1%	3999270	1	0.0%	3130179	4	56.3%
3160161	KCS6297	KM783	83	1	2	21.9%	1109074	1	0.0%	3130119	4	43.8%
3160103	KCS6299	KM784	99	1	6	96.9%	3999306	6	100.0%	3110155	6	93.8%
3160039	KCS6301	KM786	110	1	4	50.0%	2120125	6	100.0%	3130153	1	0.0%
3160186	KCS6302	KM787	72	1	3	37.5%	3100189	5	68.8%	3090048	1	3.1%
3160175	KCS6303	KM788	76	1	3	37.5%	1119033	3	37.5%	3090171	2	34.4%
3160192	KCS6305	KM789	68	2	6	100.0%	3129930	6	100.0%	3130055	6	96.9%
3160045	KCS6306	KM790	108	1	2	28.1%	3999293	3	37.5%	3130122	1	18.8%
3160036	KCS6307	KM791	110	2	4	50.0%	3100189	5	68.8%	3130008	2	31.3%
3160167	KCS6308	KM792	79	1	3	37.5%	1119033	3	37.5%	3050050	3	37.5%
3160197	KCS6309	KM793	67	2	6	96.9%	2120125	6	100.0%	3130186	6	93.8%
3160047	KCS6311	KM795	107	2	1	18.8%	3999287	1	0.0%	3130093	3	37.5%
3160091	KCS6313	KM796	100	2	3	37.5%	3999293	3	37.5%	3110007	3	37.5%
3160012	KCS6315	KM797	118	2	4	50.0%	3080011	2	28.1%	3130155	5	68.8%
3160166	KCS6316	KM798	80	1	5	75.0%	3999284	6	100.0%	3130063	4	53.1%
3160169	KCS6319	KM799	79	2	6	100.0%	3999306	6	100.0%	3130126	6	96.9%
3160027	KCS6321	KM800	114	1	3	37.5%	3999294	3	37.5%	3080057	3	37.5%
3160007	KCS6322	KM801	120	2	2	34.4%	1109074	1	0.0%	3130036	5	68.8%
3160202	KCS6323	KM802	66	2	4	43.8%	3080011	2	28.1%	3050104	5	62.5%

3160029	KCS6324	KM803	113	2	6	96.9%	3999296	6	100.0%	3130242	6	93.8%
3160183	KCS6326	KM804	73	2	5	65.6%	3100189	5	68.8%	3060126	5	62.5%
3160196	KCS6328	KM806	68	2	2	34.4%	3110021	4	50.0%	3080189	1	18.8%
3160058	KCS6329	KM807	105	2	5	71.9%	2120125	6	100.0%	3130046	4	43.8%
3160033	KCS6330	KM808	112	1	4	43.8%	3080077	4	50.0%	3130039	4	40.6%
3160177	KCS6333	KM809	76	2	4	56.3%	3129930	6	100.0%	3120043	1	12.5%
3160102	KCS6334	KM810	99	1	4	46.9%	3080077	4	50.0%	3120040	4	43.8%
3160022	KCS6335	KM811	115	1	4	53.1%	1119033	3	37.5%	3130174	5	68.8%
3160035	KCS6336	KM812	111	1	4	46.9%	3080063	4	50.0%	3130258	4	43.8%
3160156	KCS6337	KM813	86	1	6	96.9%	3999284	6	100.0%	3130013	6	93.8%
3160023	KCS6338	KM814	115	2	4	43.8%	3100189	5	68.8%	3130026	1	18.8%
3160057	KCS6339	KM815	105	1	4	50.0%	3999296	6	100.0%	3130049	1	3.1%
3160195	KCS6340	KM816	68	2	1	18.8%	3090067	2	31.3%	3080118	1	9.4%
3160201	KCS6341	KM817	67	2	2	25.0%	1119033	3	37.5%	3110009	1	12.5%
3160189	KCS6342	KM818	69	1	2	34.4%	3999270	1	0.0%	3060170	5	68.8%
3160208	KCS6351	KM820	85	2	2	28.1%	3999305	3	37.5%	3110137	1	18.8%
3160212	KCS6352	KM821	82	1	6	100.0%	3129930	6	100.0%	3120074	6	96.9%
3160240	KCS6353	KM822	68	2	5	62.5%	3129930	6	100.0%	3130278	2	28.1%
3160251	KCS6355	KM824	61	2	4	43.8%	1119033	3	37.5%	3070133	4	53.1%
3160245	KCS6357	KM826	65	2	1	18.8%	1109074	1	0.0%	3130177	3	37.5%
3160222	KCS6358	KM827	76	2	1	15.6%	1109074	1	0.0%	3100194	2	31.3%
3160246	KCS6359	KM828	65	1	3	37.5%	3110021	4	50.0%	3080091	2	21.9%
3160241	KCS6361	KM829	67	2	1	15.6%	1109074	1	0.0%	3090150	2	31.3%
3160233	KCS6363	KM831	71	2	6	100.0%	2120125	6	100.0%	3110104	6	100.0%
3160243	KCS6365	KM833	65	2	6	96.9%	2120125	6	100.0%	3110123	6	93.8%
3160229	KCS6366	KM834	72	1	4	46.9%	3090067	2	31.3%	3120227	5	62.5%
3160214	KCS6367	KM835	81	1	4	56.3%	3999306	6	100.0%	3100221	1	12.5%
3160207	KCS6370	KM837	85	1	6	100.0%	2120125	6	100.0%	3130035	6	96.9%
3160230	KCS6372	KM838	72	2	5	68.8%	3100189	5	68.8%	3110018	5	68.8%
3160248	KCS6375	KM840	62	1	3	37.5%	1119033	3	37.5%	3070124	3	37.5%
3160211	KCS6376	KM841	82	1	4	43.8%	3080077	4	50.0%	3110160	3	37.5%
3160239	KCS6378	KM843	68	1	4	40.6%	1119033	3	37.5%	3090163	4	43.8%
3160234	KCS6379	KM844	71	2	2	34.4%	1109074	1	0.0%	3120168	5	68.8%
3160235	KCS6380	KM845	69	2	3	37.5%	3090067	2	31.3%	3120007	4	40.6%
3160221	KCS6381	KM846	77	2	1	6.3%	1109074	1	0.0%	3110003	1	9.4%
3160250	KCS6382	KM847	61	2	4	53.1%	3100189	5	68.8%	3050059	3	37.5%
3160231	KCS6383	KM848	71	1	2	25.0%	1109074	1	0.0%	3130132	4	50.0%
3160224	KCS6385	KM849	75	1	5	62.5%	3999307	6	100.0%	3050052	2	25.0%
3160215	KCS6386	KM850	81	2	3	37.5%	3110021	4	50.0%	3110142	2	25.0%
3160225	KCS6387	KM851	74	2	4	43.8%	1119033	3	37.5%	3120117	4	50.0%
3160220	KCS6388	KM852	77	1	2	25.0%	1109074	1	0.0%	3110162	4	46.9%
3160244	KCS6389	KM853	65	1	5	75.0%	3129930	6	100.0%	3120176	4	46.9%
3160213	KCS6390	KM854	82	1	4	46.9%	3110021	4	50.0%	3110129	4	43.8%
3160249	KCS6391	KM855	61	2	5	75.0%	3129930	6	100.0%	3080080	4	53.1%
3160217	KCS6392	KM856	79	1	3	37.5%	1109074	1	0.0%	3120058	5	78.1%
3160219	KCS6393	KM857	77	2	5	68.8%	3110021	4	50.0%	3120235	6	84.4%
3160216	KCS6394	KM858	79	1	1	6.3%	1109074	1	0.0%	3120035	1	12.5%
3160206	KCS6395	KM859	85	1	5	78.1%	3100189	5	68.8%	3090144	6	87.5%
3160226	KCS6397	KM861	73	2	6	81.3%	3129930	6	100.0%	3120018	5	65.6%
3160227	KCS6399	KM862	73	1	4	50.0%	3110021	4	50.0%	3120146	4	50.0%
3160209	KCS6401	KM863	84	1	6	100.0%	3999284	6	100.0%	3120066	6	100.0%
3160247	KCS6402	KM864	63	2	4	50.0%	3100189	5	68.8%	3090053	2	34.4%
3160237	KCS6405	KM865	69	2	4	56.3%	3100189	5	68.8%	3040141	4	46.9%
3160238	KCS6406	KM866	69	1	2	25.0%	1109074	1	0.0%	3030016	4	46.9%
3160236	KCS6407	KM867	69	1	4	43.8%	1119033	3	37.5%	3120131	4	50.0%

Notes:

[1]BG: breed group

[2]The Brahman proportion is estimated by pedigree
