# Supplementary material for: Host genetic effects upon the early gut microbiota in a bovine model with graduated spectrum of genetic variation
Source: ISME J. 2019 Oct 17;14(1):302–17. doi: 10.1038/s41396-019-0529-2 (PMC6908690; doi:10.1038/s41396-019-0529-2)
Supplement: Supplementary file 3 — Supplementary Table S2. Sequencing information [file 41396_2019_529_MOESM3_ESM.pdf]

Supplementary Table S2. Sequencing information.

Calf ID	Sequencing ID	Raw paired end reads	Reads after joining, demultiplexing and quality filtering	Reads after removal of chimeric sequences (Total OTUs)
3160006	KM719	105782	43466	42809
3160007	KM801	94304	41829	41322
3160008	KM584	207072	42464	41258
3160009	KM749	72060	31211	30710
3160010	KM757	63342	27402	27190
3160011	KM720	153356	69239	68445
3160012	KM797	74582	31511	30317
3160013	KM746	81514	35523	35311
3160015	KM756	122012	53256	52383
3160017	KM738	72022	28680	28129
3160018	KM763	152638	66969	65133
3160019	KM739	73146	29825	29373
3160020	KM724	61348	27279	26865
3160021	KM760	117848	50663	49477
3160022	KM811	97252	43052	42237
3160023	KM814	98872	43405	43130
3160024	KM627	77022	33255	32870
3160025	KM622	112154	49201	48753
3160026	KM779	129860	56778	55040
3160027	KM800	91790	39190	37980
3160028	KM766	54434	24138	23705
3160029	KM803	83392	36865	35797
3160030	KM741	107340	41836	41162
3160031	KM740	120500	47823	47449
3160032	KM767	64342	28603	28298
3160033	KM808	117424	52365	50577
3160034	KM636	110750	50511	49658
3160035	KM812	91834	39741	38683
3160036	KM791	37308	16308	15919
3160037	KM726	86362	37865	37279
3160038	KM745	50728	22047	21820
3160039	KM786	124516	55542	54529
3160040	KM610	193662	39258	38752
3160041	KM712	65956	29098	30069
3160043	KM764	97144	40369	40076
3160044	KM776	65490	29258	29002
3160045	KM790	92270	40913	39864
3160046	KM742	103278	44697	44052
3160047	KM795	102402	43879	43358
3160048	KM592	221252	44295	43463
3160049	KM585	156872	27347	26728
3160050	KM615	181188	29905	29445
3160051	KM618	195162	29404	29054
3160052	KM634	113348	51629	50988
3160053	KM658	71216	30178	29611
3160054	KM613	257418	51728	50819
3160055	KM620	172198	31461	31067
3160056	KM731	81988	33128	32486
3160057	KM815	100208	44072	43460
3160058	KM807	106636	46301	45381
3160059	KM638	96588	41288	40633
3160060	KM630	95736	43263	42571

3160061	KM703	91142	40055	39444
3160062	KM605	196036	37460	36818
3160063	KM761	103878	44767	43879
3160064	KM723	97976	43138	42508
3160065	KM752	95388	40447	39947
3160066	KM708	106664	47226	46156
3160067	KM619	234844	49497	48758
3160068	KM656	95302	41249	40289
3160070	KM716	80326	35587	35175
3160071	KM645	101150	45122	44232
3160072	KM606	201652	27347	26672
3160073	KM591	171438	27738	27250
3160074	KM589	213532	32025	31489
3160075	KM626	81382	36338	35953
3160076	KM586	213620	42098	41392
3160077	KM588	174474	29948	29317
3160078	KM647	95276	42307	41465
3160079	KM635	67824	29775	29402
3160080	KM611	81838	35586	35152
3160081	KM709	37024	16304	16022
3160082	KM587	186728	36702	35962
3160083	KM711	97876	41421	40804
3160084	KM609	198664	23163	22665
3160085	KM614	280730	55272	54517
3160086	KM639	95324	42326	41794
3160087	KM608	77346	34000	33402
3160088	KM653	88650	37677	36874
3160089	KM710	96308	41971	41202
3160090	KM604	199356	32437	31986
3160091	KM796	82514	36247	35242
3160092	KM632	83268	37605	36954
3160093	KM581	181080	78059	75866
3160094	KM624	84370	36813	36079
3160095	KM715	84078	36562	35528
3160096	KM660	69330	30253	29717
3160097	KM640	110620	46562	45834
3160098	KM651	151892	67984	66849
3160099	KM637	72860	32040	31523
3160100	KM643	113450	49171	47633
3160101	KM648	114446	49637	48248
3160102	KM810	110402	48082	47250
3160103	KM784	99558	44549	43797
3160104	KM599	184368	32060	31250
3160105	KM652	116224	48664	47171
3160106	KM603	143978	24205	23631
3160107	KM617	192158	36886	36374
3160108	KM582	193452	22833	22389
3160109	KM655	102126	45257	44441
3160110	KM629	94390	41929	41359
3160111	KM644	83084	35604	34782
3160112	KM649	97668	42054	40881
3160113	KM612	187514	38578	37715
3160114	KM598	203066	37656	36875
3160115	KM705	97774	42282	41802
3160116	KM633	130906	54588	53680

3160117	KM650	116630	51104	49878
3160118	KM602	218308	27920	27501
3160119	KM725	75816	33971	33459
3160120	KM654	87414	38665	37885
3160121	KM593	156346	29515	28850
3160122	KM628	126778	52260	51547
3160123	KM727	71814	31572	31279
3160124	KM751	72390	31360	30827
3160125	KM729	151364	65736	64838
3160126	KM753	99150	41359	40773
3160127	KM594	193190	28532	27992
3160128	KM600	198538	32442	31661
3160129	KM597	77316	33453	33114
3160130	KM641	91754	38745	37460
3160131	KM646	159470	69940	68682
3160132	KM734	79352	28571	27905
3160133	KM702	81982	34463	33766
3160134	KM583	174746	20963	20534
3160135	KM625	63380	27726	27419
3160136	KM704	103166	44138	43477
3160137	KM590	198912	39746	38988
3160138	KM730	96702	38922	38175
3160139	KM721	139942	61491	60381
3160140	KM601	187794	24445	23574
3160141	KM659	64300	27894	27520
3160143	KM631	117616	52903	51791
3160144	KM657	132126	57720	57099
3160145	KM623	61080	26886	26619
3160146	KM621	88704	37883	36663
3160147	KM706	83890	37354	36484
3160148	KM607	209412	27611	27211
3160149	KM748	92022	40518	39946
3160150	KM769	58412	26055	25888
3160151	KM744	105482	45427	44398
3160153	KM595	205508	29252	28390
3160154	KM737	92000	37410	36607
3160156	KM813	139404	60614	58917
3160157	KM714	72554	31751	31165
3160158	KM713	97294	43592	42646
3160159	KM707	83456	35725	35076
3160160	KM758	75066	31550	31412
3160161	KM783	83984	36927	36164
3160162	KM774	107384	47974	46511
3160163	KM775	106652	48392	47062
3160164	KM722	65396	28849	28733
3160166	KM798	69816	30479	29398
3160167	KM792	97022	42405	42089
3160168	KM782	102840	45403	44294
3160169	KM799	92964	39722	39028
3160170	KM755	100898	44056	42776
3160171	KM717	79576	34902	34263
3160172	KM735	85022	33288	32734
3160173	KM750	100714	43429	43223
3160174	KM780	116568	50946	49397
3160175	KM788	151332	65364	63336

3160176	KM747	91212	38445	37939
3160177	KM809	125970	54733	53657
3160178	KM728	118108	50966	50330
3160179	KM642	113756	49330	48411
3160181	KM743	77000	33122	32344
3160182	KM781	78274	33631	32026
3160183	KM804	99728	43476	42155
3160184	KM759	88300	37664	36506
3160185	KM777	66618	29780	29471
3160186	KM787	112362	49186	48283
3160187	KM768	63560	27593	27236
3160188	KM772	110238	49945	49561
3160189	KM818	94702	41386	40732
3160191	KM596	175780	29913	29398
3160192	KM789	177292	76394	73530
3160193	KM762	164892	73086	71807
3160195	KM816	128922	55432	53735
3160196	KM806	93390	38848	37560
3160197	KM793	89998	39073	37767
3160199	KM773	93992	41025	40086
3160200	KM732	72074	28769	27753
3160201	KM817	73264	31719	31088
3160202	KM802	87550	38761	37898
3160203	KM718	103804	44867	44010
3160204	KM771	69810	30613	29817
3160205	KM754	92820	40519	39578
3160206	KM859	100130	45773	45176
3160207	KM837	116430	46721	46368
3160208	KM820	105280	45928	44641
3160209	KM863	100060	43319	42287
3160211	KM841	120896	52197	51197
3160212	KM821	102968	43609	42689
3160213	KM854	144238	63968	62878
3160214	KM835	96402	38480	37987
3160215	KM850	94972	41759	40719
3160216	KM858	102898	44706	43456
3160217	KM856	30710	13832	13656
3160219	KM857	81274	36306	35795
3160220	KM852	100418	45429	44327
3160221	KM846	109176	47174	46244
3160222	KM827	117416	47998	47253
3160224	KM849	197136	84366	81888
3160225	KM851	94476	41566	40612
3160226	KM861	127694	56106	54687
3160227	KM862	114212	49809	48720
3160229	KM834	120958	44080	43467
3160230	KM838	186182	78518	76619
3160231	KM848	152918	65836	65411
3160233	KM831	84656	33577	32773
3160234	KM844	113330	49560	48318
3160235	KM845	134734	57077	56314
3160236	KM867	88354	39952	39734
3160237	KM865	106758	46929	45963
3160238	KM866	134540	58661	57293
3160239	KM843	103612	43133	42454

3160240	KM822	106968	46350	45205
3160241	KM829	133744	52476	52031
3160243	KM833	109918	42774	41648
3160244	KM853	127920	56011	54704
3160245	KM826	158122	63882	62622
3160246	KM828	148574	58497	57354
3160247	KM864	104878	46162	44865
3160248	KM840	115576	48919	47843
3160249	KM855	112632	50230	49124
3160250	KM847	117602	50868	49628
3160251	KM824	101774	43479	42506
