# Supplementary material for: Host genetic effects upon the early gut microbiota in a bovine model with graduated spectrum of genetic variation
Source: ISME J. 2019 Oct 17;14(1):302–17. doi: 10.1038/s41396-019-0529-2 (PMC6908690; doi:10.1038/s41396-019-0529-2)
Supplement: Supplementary file 4 — Supplementary Table S3. Correlation between Brahman proportion and OTU prevalence [file 41396_2019_529_MOESM4_ESM.pdf]

Supplementary Table S3. Correlation between Brahman proportion and OTU prevalence.

Silva OTU ID	Assigned taxa	OTU prevalence						Pearson correlation coefficient	P_{raw} value	$P_{adjusted}$ value
		BG1	BG2	BG3	BG4	BG5	BG6			
CP002106.159965.161467	p_Actinobacteria; c_Coriobacteriia; o_Coriobacteriales; f_Coriobacteriaceae; g_; s_	38.24%	38.10%	51.61%	43.64%	29.03%	28.57%	-0.591	0.217	0.356
AB269993.1.1510	p_Actinobacteria; c_Coriobacteriia; o_Coriobacteriales; f_Coriobacteriaceae; g_; s_	32.35%	30.95%	35.48%	49.09%	58.06%	34.29%	0.395	0.438	0.582
New.CleanUp.ReferenceOTU22410	p_Actinobacteria; c_Coriobacteriia; o_Coriobacteriales; f_Coriobacteriaceae; g_; s_	47.06%	35.71%	38.71%	45.45%	61.29%	42.86%	0.320	0.537	0.666
AB270132.1.1511	p_Actinobacteria; c_Coriobacteriia; o_Coriobacteriales; f_Coriobacteriaceae; g_; s_	38.24%	47.62%	45.16%	60.00%	51.61%	60.00%	0.812	0.049	0.127
AB746605.1.1507	p_Actinobacteria; c_Coriobacteriia; o_Coriobacteriales; f_Coriobacteriaceae; g_; s_	67.65%	57.14%	61.29%	74.55%	77.42%	68.57%	0.470	0.347	0.488
AB559621.1.1499	p_Actinobacteria; c_Coriobacteriia; o_Coriobacteriales; f_Coriobacteriaceae; g_Collinsella; s_aerofaciens	14.71%	26.19%	35.48%	43.64%	48.39%	65.71%	0.987	0.000	0.010
New.ReferenceOTU151	p_Actinobacteria; c_Coriobacteriia; o_Coriobacteriales; f_Coriobacteriaceae; g_Slackia; s_	70.59%	57.14%	87.10%	70.91%	70.97%	71.43%	0.097	0.855	0.893
New.ReferenceOTU183	p_Bacteroidetes; c_Bacteroidia; o_Bacteroidales; f_; g_; s_	55.88%	40.48%	25.81%	23.64%	22.58%	8.57%	-0.927	0.008	0.042
New.CleanUp.ReferenceOTU12439	p_Bacteroidetes; c_Bacteroidia; o_Bacteroidales; f_; g_; s_	50.00%	30.95%	38.71%	27.27%	19.35%	11.43%	-0.946	0.004	0.032
FJ951848.1.1488	p_Bacteroidetes; c_Bacteroidia; o_Bacteroidales; f_; g_; s_	61.76%	40.48%	29.03%	25.45%	29.03%	17.14%	-0.856	0.030	0.088
FJ951840.1.1491	p_Bacteroidetes; c_Bacteroidia; o_Bacteroidales; f_; g_; s_	52.94%	47.62%	29.03%	30.91%	29.03%	17.14%	-0.909	0.012	0.054
EU794077.1.1453	p_Bacteroidetes; c_Bacteroidia; o_Bacteroidales; f_; g_; s_	47.06%	59.52%	38.71%	30.91%	19.35%	20.00%	-0.853	0.031	0.091
AY244882.1.1488	p_Bacteroidetes; c_Bacteroidia; o_Bacteroidales; f_; g_; s_	50.00%	28.57%	41.94%	32.73%	22.58%	20.00%	-0.846	0.034	0.098
EU794094.1.1451	p_Bacteroidetes; c_Bacteroidia; o_Bacteroidales; f_; g_; s_	58.82%	47.62%	58.06%	34.55%	29.03%	22.86%	-0.911	0.011	0.052
LC028574.1.1518	p_Bacteroidetes; c_Bacteroidia; o_Bacteroidales; f_; g_; s_	67.65%	52.38%	48.39%	36.36%	32.26%	22.86%	-0.969	0.001	0.019
LC028792.1.1522	p_Bacteroidetes; c_Bacteroidia; o_Bacteroidales; f_; g_; s_	70.59%	54.76%	54.84%	50.91%	45.16%	22.86%	-0.965	0.002	0.021
KR514411.1.1450	p_Bacteroidetes; c_Bacteroidia; o_Bacteroidales; f_; g_; s_	50.00%	42.86%	32.26%	29.09%	12.90%	25.71%	-0.813	0.049	0.127
JX095613.1.1399	p_Bacteroidetes; c_Bacteroidia; o_Bacteroidales; f_; g_; s_	64.71%	47.62%	58.06%	49.09%	32.26%	42.86%	-0.760	0.080	0.173
EU794163.1.1454	p_Bacteroidetes; c_Bacteroidia; o_Bacteroidales; f_; g_; s_	85.29%	69.05%	74.19%	54.55%	45.16%	45.71%	-0.920	0.009	0.046
LC028780.1.1522	p_Bacteroidetes; c_Bacteroidia; o_Bacteroidales; f_; g_; s_	82.35%	78.57%	67.74%	58.18%	48.39%	45.71%	-0.959	0.003	0.024
LC028693.1.1520	p_Bacteroidetes; c_Bacteroidia; o_Bacteroidales; f_; g_; s_	58.82%	59.52%	77.42%	63.64%	54.84%	45.71%	-0.573	0.235	0.375
LC028553.1.1520	p_Bacteroidetes; c_Bacteroidia; o_Bacteroidales; f_; g_; s_	73.53%	66.67%	74.19%	56.36%	41.94%	48.57%	-0.851	0.032	0.093
EU777151.1.1352	p_Bacteroidetes; c_Bacteroidia; o_Bacteroidales; f_; g_; s_	73.53%	71.43%	80.65%	67.27%	58.06%	51.43%	-0.867	0.025	0.082
LC028634.1.1522	p_Bacteroidetes; c_Bacteroidia; o_Bacteroidales; f_; g_; s_	85.29%	78.57%	80.65%	65.45%	70.97%	57.14%	-0.911	0.011	0.052
LC028867.1.1518	p_Bacteroidetes; c_Bacteroidia; o_Bacteroidales; f_; g_; s_	88.24%	76.19%	77.42%	65.45%	61.29%	60.00%	-0.929	0.007	0.042
LC028766.1.1522	p_Bacteroidetes; c_Bacteroidia; o_Bacteroidales; f_; g_; s_	88.24%	80.95%	74.19%	69.09%	58.06%	62.86%	-0.907	0.013	0.055
LC028691.1.1522	p_Bacteroidetes; c_Bacteroidia; o_Bacteroidales; f_; g_; s_	91.18%	83.33%	83.87%	69.09%	70.97%	62.86%	-0.936	0.006	0.036
LC028565.1.1522	p_Bacteroidetes; c_Bacteroidia; o_Bacteroidales; f_; g_; s_	85.29%	83.33%	80.65%	74.55%	67.74%	65.71%	-0.972	0.001	0.018
LC028589.1.1522	p_Bacteroidetes; c_Bacteroidia; o_Bacteroidales; f_; g_; s_	97.06%	92.86%	96.77%	80.00%	77.42%	77.14%	-0.872	0.023	0.078
EU794219.1.1459	p_Bacteroidetes; c_Bacteroidia; o_Bacteroidales; f_[Barnesiellaceae]; g_; s_	70.59%	59.52%	54.84%	45.45%	35.48%	25.71%	-0.994	0.000	0.008
AWWG01000020.296.1831	p_Bacteroidetes; c_Bacteroidia; o_Bacteroidales; f_[Barnesiellaceae]; g_; s_	26.47%	35.71%	35.48%	41.82%	70.97%	60.00%	0.877	0.022	0.077
FBYB01000006.1397107.1398611	p_Bacteroidetes; c_Bacteroidia; o_Bacteroidales; f_[Odoribacteraceae]; g_Butyricimonas; s_	38.24%	45.24%	48.39%	40.00%	45.16%	54.29%	0.731	0.099	0.200
AB874571.1.1447	p_Bacteroidetes; c_Bacteroidia; o_Bacteroidales; f_[Odoribacteraceae]; g_Butyricimonas; s_	55.88%	61.90%	70.97%	65.45%	77.42%	68.57%	0.686	0.132	0.251
CDTY01027388.3437.4940	p_Bacteroidetes; c_Bacteroidia; o_Bacteroidales; f_[Odoribacteraceae]; g_Odoribacter; s_	35.29%	42.86%	48.39%	54.55%	54.84%	57.14%	0.906	0.013	0.055
GQ448681.1.1394	p_Bacteroidetes; c_Bacteroidia; o_Bacteroidales; f_[Odoribacteraceae]; g_Odoribacter; s_	88.24%	85.71%	83.87%	92.73%	93.55%	94.29%	0.750	0.086	0.183
JN559646.1.1500	p_Bacteroidetes; c_Bacteroidia; o_Bacteroidales; f_[Paraprevotellaceae]; g_; s_	5.88%	19.05%	25.81%	29.09%	22.58%	51.43%	0.889	0.018	0.068
New.ReferenceOTU2	p_Bacteroidetes; c_Bacteroidia; o_Bacteroidales; f_[Paraprevotellaceae]; g_[Prevotella]; s_	67.65%	52.38%	51.61%	45.45%	38.71%	34.29%	-0.956	0.003	0.026
JX095635.1.1399	p_Bacteroidetes; c_Bacteroidia; o_Bacteroidales; f_[Paraprevotellaceae]; g_[Prevotella]; s_	64.71%	71.43%	61.29%	49.09%	41.94%	40.00%	-0.898	0.015	0.061
New.ReferenceOTU125	p_Bacteroidetes; c_Bacteroidia; o_Bacteroidales; f_[Paraprevotellaceae]; g_[Prevotella]; s_	8.82%	11.90%	25.81%	30.91%	35.48%	54.29%	0.976	0.001	0.017
FJ678923.1.1446	p_Bacteroidetes; c_Bacteroidia; o_Bacteroidales; f_[Paraprevotellaceae]; g_[Prevotella]; s_	50.00%	61.90%	51.61%	72.73%	77.42%	65.71%	0.650	0.163	0.293
FJ682054.1.1473	p_Bacteroidetes; c_Bacteroidia; o_Bacteroidales; f_[Paraprevotellaceae]; g_[Prevotella]; s_	44.12%	54.76%	70.97%	80.00%	87.10%	88.57%	0.920	0.009	0.046
EU474827.1.1245	p_Bacteroidetes; c_Bacteroidia; o_Bacteroidales; f_[Paraprevotellaceae]; g_CF231; s_	55.88%	57.14%	45.16%	43.64%	35.48%	31.43%	-0.950	0.004	0.029
EU794267.1.1453	p_Bacteroidetes; c_Bacteroidia; o_Bacteroidales; f_[Paraprevotellaceae]; g_CF231; s_	67.65%	69.05%	61.29%	61.82%	41.94%	40.00%	-0.932	0.007	0.039
HM124005.1.1488	p_Bacteroidetes; c_Bacteroidia; o_Bacteroidales; f_[Paraprevotellaceae]; g_CF231; s_	73.53%	80.95%	80.65%	83.64%	90.32%	85.71%	0.831	0.041	0.111
EU794144.1.1446	p_Bacteroidetes; c_Bacteroidia; o_Bacteroidales; f_Bacteroidaceae; g_; s_	61.76%	47.62%	25.81%	16.36%	19.35%	5.71%	-0.903	0.014	0.057
EU468705.1.1287	p_Bacteroidetes; c_Bacteroidia; o_Bacteroidales; f_Bacteroidaceae; g_; s_	50.00%	42.86%	38.71%	34.55%	32.26%	14.29%	-0.974	0.001	0.018
FJ848390.1.1496	p_Bacteroidetes; c_Bacteroidia; o_Bacteroidales; f_Bacteroidaceae; g_; s_	61.76%	54.76%	45.16%	27.27%	29.03%	17.14%	-0.944	0.005	0.032
JX096097.1.1397	p_Bacteroidetes; c_Bacteroidia; o_Bacteroidales; f_Bacteroidaceae; g_; s_	50.00%	19.05%	35.48%	25.45%	22.58%	20.00%	-0.674	0.142	0.267
EU465631.1.1395	p_Bacteroidetes; c_Bacteroidia; o_Bacteroidales; f_Bacteroidaceae; g_; s_	76.47%	52.38%	61.29%	40.00%	38.71%	22.86%	-0.939	0.005	0.035
EU794226.1.1442	p_Bacteroidetes; c_Bacteroidia; o_Bacteroidales; f_Bacteroidaceae; g_; s_	64.71%	52.38%	54.84%	38.18%	22.58%	25.71%	-0.930	0.007	0.040
LC028829.1.1517	p_Bacteroidetes; c_Bacteroidia; o_Bacteroidales; f_Bacteroidaceae; g_; s_	76.47%	64.29%	51.61%	43.64%	35.48%	28.57%	-0.963	0.002	0.022
JX095438.1.1393	p_Bacteroidetes; c_Bacteroidia; o_Bacteroidales; f_Bacteroidaceae; g_5-7N15; s_	55.88%	45.24%	25.81%	10.91%	16.13%	11.43%	-0.847	0.033	0.097
New.ReferenceOTU337	p_Bacteroidetes; c_Bacteroidia; o_Bacteroidales; f_Bacteroidaceae; g_5-7N15; s_	55.88%	28.57%	29.03%	27.27%	9.68%	14.29%	-0.863	0.027	0.085
FJ951907.1.1487	p_Bacteroidetes; c_Bacteroidia; o_Bacteroidales; f_Bacteroidaceae; g_5-7N15; s_	55.88%	30.95%	25.81%	21.82%	22.58%	14.29%	-0.845	0.034	0.099
New.CleanUp.ReferenceOTU12984	p_Bacteroidetes; c_Bacteroidia; o_Bacteroidales; f_Bacteroidaceae; g_5-7N15; s_	70.59%	45.24%	45.16%	34.55%	29.03%	14.29%	-0.959	0.003	0.024
JX095553.1.1395	p_Bacteroidetes; c_Bacteroidia; o_Bacteroidales; f_Bacteroidaceae; g_5-7N15; s_	64.71%	52.38%	45.16%	32.73%	22.58%	17.14%	-0.973	0.001	0.018
KC991126.1.1446	p_Bacteroidetes; c_Bacteroidia; o_Bacteroidales; f_Bacteroidaceae; g_5-7N15; s_	61.76%	52.38%	48.39%	38.18%	29.03%	25.71%	-0.973	0.001	0.018
New.ReferenceOTU298	p_Bacteroidetes; c_Bacteroidia; o_Bacteroidales; f_Bacteroidaceae; g_5-7N15; s_	73.53%	66.67%	58.06%	47.27%	32.26%	25.71%	-0.983	0.000	0.011
FJ848410.1.1494	p_Bacteroidetes; c_Bacteroidia; o_Bacteroidales; f_Bacteroidaceae; g_5-7N15; s_	79.41%	73.81%	64.52%	60.00%	48.39%	31.43%	-0.995	0.000	0.008
New.ReferenceOTU225	p_Bacteroidetes; c_Bacteroidia; o_Bacteroidales; f_Bacteroidaceae; g_5-7N15; s_	70.59%	66.67%	61.29%	47.27%	41.94%	34.29%	-0.971	0.001	0.018
EU794240.1.1447	p_Bacteroidetes; c_Bacteroidia; o_Bacteroidales; f_Bacteroidaceae; g_5-7N15; s_	88.24%	76.19%	70.97%	54.55%	48.39%	42.86%	-0.960	0.002	0.024
LC028711.1.1516	p_Bacteroidetes; c_Bacteroidia; o_Bacteroidales; f_Bacteroidaceae; g_5-7N15; s_	85.29%	76.19%	74.19%	61.82%	64.52%	42.86%	-0.959	0.003	0.024
KJ571117.1.1485	p_Bacteroidetes; c_Bacteroidia; o_Bacteroidales; f_Bacteroidaceae; g_5-7N15; s_	82.35%	78.57%	67.74%	67.27%	58.06%	48.57%	-0.984	0.000	0.011
EU778731.1.1402	p_Bacteroidetes; c_Bacteroidia; o_Bacteroidales; f_Bacteroidaceae; g_Bacteroides; s_	67.65%	42.86%	41.94%	27.27%	29.03%	17.14%	-0.914	0.011	0.051
HM124113.1.1489	p_Bacteroidetes; c_Bacteroidia; o_Bacteroidales; f_Bacteroidaceae; g_Bacteroides; s_	23.53%	42.86%	45.16%	54.55%	38.71%	28.57%	-0.021	0.968	0.982
JN167608.1.1489	p_Bacteroidetes; c_Bacteroidia; o_Bacteroidales; f_Bacteroidaceae; g_Bacteroides; s_	76.47%	66.67%	61.29%	41.82%	41.94%	28.57%	-0.960	0.002	0.023
HQ757038.1.1425	p_Bacteroidetes; c_Bacteroidia; o_Bacteroidales; f_Bacteroidaceae; g_Bacteroides; s_	17.65%	19.05%	29.03%	40.00%	51.61%	48.57%	0.919	0.009	0.046
FQXX01000006.451362.452869	p_Bacteroidetes; c_Bacteroidia; o_Bacteroidales; f_Bacteroidaceae; g_Bacteroides; s_	17.65%	26.19%	38.71%	40.00%	35.48%	51.43%	0.886	0.019	0.071
CP009057.4969468.4971009	p_Bacteroidetes; c_Bacteroidia; o_Bacteroidales; f_Bacteroidaceae; g_Bacteroides; s_	23.53%	28.57%	29.03%	34.55%	41.94%	51.43%	0.992	0.000	0.008
GQ871708.1.1489	p_Bacteroidetes; c_Bacteroidia; o_Bacteroidales; f_Bacteroidaceae; g_Bacteroides; s_	32.35%	47.62%	32.26%	40.00%	54.84%	60.00%	0.834	0.039	0.108
New.ReferenceOTU336	p_Bacteroidetes; c_Bacteroidia; o_Bacteroidales; f_Bacteroidaceae; g_Bacteroides; s_	32.35%	52.38%	32.26%	36.36%	48.39%	62.86%	0.720	0.106	0.211
FJ506946.1.1387	p_Bacteroidetes; c_Bacteroidia; o_Bacteroidales; f_Bacteroidaceae; g_Bacteroides; s_	17.65%	16.67%	29.03%	27.27%	41.94%	65.71%	0.953	0.003	0.027
CDTY01006121.13.1519	p_Bacteroidetes; c_Bacteroidia; o_Bacteroidales; f_Bacteroidaceae; g_Bacteroides; s_	23.53%	33.33%	32.26%	36.36%	48.39%	68.57%	0.971	0.001	0.018
CDYK01040934.16.1524	p_Bacteroidetes; c_Bacteroidia; o_Bacteroidales; f_Bacteroidaceae; g_Bacteroides; s_	20.59%	45.24%	45.16%	41.82%	64.52%	68.57%	0.925	0.008	0.043
BABD01000001.4971.6478	p_Bacteroidetes; c_Bacteroidia; o_Bacteroidales; f_Bacteroidaceae; g_Bacteroides; s_	29.41%	42.86%	29.03%	41.82%	51.61%	74.29%	0.906	0.013	0.055
CAEG01000022.14.1521	p_Bacteroidetes; c_Bacteroidia; o_Bacteroidales; f_Bacteroidaceae; g_Bacteroides; s_	23.53%	35.71%	51.61%	52.73%	61.29%	74.29%	0.968	0.002	0.019
EF403496.1.1491	p_Bacteroidetes; c_Bacteroidia; o_Bacteroidales; f_Bacteroidaceae; g_Bacteroides; s_	64.71%	78.57%	77.42%	69.09%	70.97%	85.71%	0.602	0.206	0.342
EF403478.1.1500	p_Bacteroidetes; c_Bacteroidia; o_Bacteroidales; f_Bacteroidaceae; g_Bacteroides; s_	50.00%	66.67%	64.52%	72.73%	96.77%	97.14%	0.955	0.003	0.026
AQHY01000028.339.1856	p_Bacteroidetes; c_Bacteroidia; o_Bacteroidales; f_Bacteroidaceae; g_Bacteroides; s_	61.76%	85.71%	77.42%	90.91%	100.00%	97.14%	0.857	0.029	0.088
DQ905947.1.1583	p_Bacteroidetes; c_Bacteroidia; o_Bacteroidales; f_Bacteroidaceae; g_Bacteroides; s_	64.71%	85.71%	87.10%	94.55%	100.00%	100.00%	0.877	0.022	0.077
LN998056.1.1491	p_Bacteroidetes; c_Bacteroidia; o_Bacteroidales; f_Bacteroidaceae; g_Bacteroides; s_barnesiae	26.47%	38.10%	51.61%	47.27%	54.84%	48.57%	0.724	0.104	0.208
CDZK01020573.3680.5190	p_Bacteroidetes; c_Bacteroidia; o_Bacteroidales; f_Bacteroidaceae; g_Bacteroides; s_coprophilus	64.71%	76.19%	74.19%	83.64%	96.77%	88.57%	0.860	0.028	0.086
ABVO01000045.3575.5089	p_Bacteroidetes; c_Bacteroidia; o_Bacteroidales; f_Bacteroidaceae; g_Bacteroides; s_eggerthii	26.47%	50.00%	41.94%	60.00%	77.42%	85.71%	0.964	0.002	0.021
DQ905926.1.1529	p_Bacteroidetes; c_Bacteroidia; o_Bacteroidales; f_Bacteroidaceae; g_Bacteroides; s_fragilis	61.76%	85.71%	83.87%	89.09%	93.55%	97.14%	0.861	0.028	0.086
AB200218.1.1492	p_Bacteroidetes; c_Bacteroidia; o_Bacteroidales; f_Bacteroidaceae; g_Bacteroides; s_plebeius	29.41%	45.24%	29.03%	45.45%	58.06%	60.00%	0.859	0.028	0.087
New.ReferenceOTU163	p_Bacteroidetes; c_Bacteroidia; o_Bacteroidales; f_Bacteroidaceae; g_Bacteroides; s_plebeius	50.00%	59.52%	54.84%	58.18%	80.65%	88.57%	0.942	0.005	0.033
BABD01000482.1904.3411	p_Bacteroidetes; c_Bacteroidia; o_Bacteroidales; f_Bacteroidaceae; g_Bacteroides; s_uniformis	50.00%	54.76%	67.74%	83.64%	80.65%	91.43%	0.925	0.008	0.043
JX096352.1.1389	p_Bacteroidetes; c_Bacteroidia; o_Bacteroidales; f_BS11; g_; s_	52.94%	38.10%	29.03%	23.64%	22.58%	20.00%	-0.872	0.023	0.078
EU470251.1.1404	p_Bacteroidetes; c_Bacteroidia; o_Bacteroidales; f_Porphyromonadaceae; g_Paludibacter; s_	64.71%	28.57%	35.48%	23.64%	19.35%	11.43%	-0.865	0.026	0.083
FJ848442.1.1500	p_Bacteroidetes; c_Bacteroidia; o_Bacteroidales; f_Porphyromonadaceae; g_Paludibacter; s_	67.65%	64.29%	48.39%	36.36%	38.71%	28.57%	-0.917	0.010	0.048
EU778625.1.1365	p_Bacteroidetes; c_Bacteroidia; o_Bacteroidales; f_Porphyromonadaceae; g_Parabacteroides; s_	26.47%	23.81%	32.26%	25.45%	32.26%	51.43%	0.826	0.043	0.115
CDYE01001899.59024.60534	p_Bacteroidetes; c_Bacteroidia; o_Bacteroidales; f_Porphyromonadaceae; g_Parabacteroides; s_	23.53%	23.81%	29.03%	43.64%	51.61%	68.57%	0.975	0.001	0.018
FJ683073.1.1401	p_Bacteroidetes; c_Bacteroidia; o_Bacteroidales; f_Porphyromonadaceae; g_Parabacteroides; s_	38.24%	57.14%	51.61%	54.55%	61.29%	82.86%	0.927	0.008	0.042
KF843488.1.1412	p_Bacteroidetes; c_Bacteroidia; o_Bacteroidales; f_Prevotellaceae; g_Prevotella; s_stercorea	8.82%	21.43%	25.81%	32.73%	38.71%	51.43%	0.991	0.000	0.008
CDYZ01066022.19.1525	p_Bacteroidetes; c_Bacteroidia; o_Bacteroidales; f_Prevotellaceae; g_Prevotella; s_stercorea	67.65%	88.10%	93.55%	94.55%	90.32%	97.14%	0.731	0.099	0.200
JX095373.1.1393	p_Bacteroidetes; c_Bacteroidia; o_Bacteroidales; f_RF16; g_; s_	58.82%	50.00%	41.94%	32.73%	19.35%	8.57%	-0.995	0.000	0.008
FJ848404.1.1494	p_Bacteroidetes; c_Bacteroidia; o_Bacteroidales; f_RF16; g_; s_	61.76%	52.38%	45.16%	34.55%	22.58%	14.29%	-0.989	0.000	0.009
EU460981.1.1395	p_Bacteroidetes; c_Bacteroidia; o_Bacteroidales; f_RF16; g_; s_	55.88%	50.00%	41.94%	32.73%	19.35%	17.14%	-0.970	0.001	0.018
LC028840.1.1517	p_Bacteroidetes; c_Bacteroidia; o_Bacteroidales; f_RF16; g_; s_	79.41%	76.19%	67.74%	52.73%	32.26%	37.14%	-0.928	0.008	0.042
New.ReferenceOTU110	p_Bacteroidetes; c_Bacteroidia; o_Bacteroidales; f_Rikenellaceae; g_; s_	67.65%	52.38%	45.16%	41.82%	25.81%	8.57%	-0.996	0.000	0.008
KJ571141.1.1490	p_Bacteroidetes; c_Bacteroidia; o_Bacteroidales; f_Rikenellaceae; g_; s_	64.71%	45.24%	35.48%	30.91%	32.26%	11.43%	-0.935	0.006	0.036
New.ReferenceOTU251	p_Bacteroidetes; c_Bacteroidia; o_Bacteroidales; f_Rikenellaceae; g_; s_	61.76%	28.57%	38.71%	23.64%	25.81%	17.14%	-0.812	0.050	0.127

FJ951888.1.1489	p_Bacteroidetes; c_Bacteroidia; o_Bacteroidales; f_Rikenellaceae; g_; s_	70.59%	38.10%	45.16%	30.91%	38.71%	20.00%	-0.830	0.041	0.111
LC028769.1.1521	p_Bacteroidetes; c_Bacteroidia; o_Bacteroidales; f_Rikenellaceae; g_; s_	67.65%	50.00%	48.39%	41.82%	29.03%	22.86%	-0.976	0.001	0.017
EU773167.1.1399	p_Bacteroidetes; c_Bacteroidia; o_Bacteroidales; f_Rikenellaceae; g_; s_	61.76%	45.24%	48.39%	43.64%	32.26%	22.86%	-0.972	0.001	0.018
JX095568.1.1395	p_Bacteroidetes; c_Bacteroidia; o_Bacteroidales; f_Rikenellaceae; g_; s_	55.88%	47.62%	54.84%	60.00%	58.06%	34.29%	-0.533	0.276	0.416
KR514409.1.1448	p_Bacteroidetes; c_Bacteroidia; o_Bacteroidales; f_Rikenellaceae; g_; s_	85.29%	69.05%	74.19%	54.55%	48.39%	37.14%	-0.966	0.002	0.021
EU474723.1.1405	p_Bacteroidetes; c_Bacteroidia; o_Bacteroidales; f_Rikenellaceae; g_; s_	79.41%	73.81%	70.97%	65.45%	61.29%	48.57%	-0.992	0.000	0.008
LC028600.1.1521	p_Bacteroidetes; c_Bacteroidia; o_Bacteroidales; f_Rikenellaceae; g_; s_	85.29%	80.95%	74.19%	63.64%	54.84%	51.43%	-0.968	0.001	0.019
FJ680633.1.1448	p_Bacteroidetes; c_Bacteroidia; o_Bacteroidales; f_Rikenellaceae; g_; s_	85.29%	78.57%	70.97%	72.73%	64.52%	62.86%	-0.942	0.005	0.033
JX095333.1.1395	p_Bacteroidetes; c_Bacteroidia; o_Bacteroidales; f_Rikenellaceae; g_; s_	94.12%	80.95%	80.65%	70.91%	70.97%	65.71%	-0.922	0.009	0.045
KP745017.1.1454	p_Bacteroidetes; c_Bacteroidia; o_Bacteroidales; f_S24-7; g_; s_	55.88%	38.10%	45.16%	34.55%	25.81%	11.43%	-0.966	0.002	0.020
JX096233.1.1401	p_Bacteroidetes; c_Bacteroidia; o_Bacteroidales; f_S24-7; g_; s_	58.82%	45.24%	48.39%	40.00%	29.03%	14.29%	-0.985	0.000	0.011
New.ReferenceOTU309	p_Bacteroidetes; c_Bacteroidia; o_Bacteroidales; f_S24-7; g_; s_	64.71%	57.14%	61.29%	45.45%	45.16%	28.57%	-0.952	0.003	0.027
New.ReferenceOTU85	p_Bacteroidetes; c_Bacteroidia; o_Bacteroidales; f_S24-7; g_; s_	41.18%	50.00%	41.94%	25.45%	25.81%	34.29%	-0.581	0.226	0.365
FJ880190.1.1497	p_Bacteroidetes; c_Bacteroidia; o_Bacteroidales; f_S24-7; g_; s_	14.71%	45.24%	41.94%	58.18%	54.84%	48.57%	0.666	0.149	0.274
FJ881192.1.1499	p_Bacteroidetes; c_Bacteroidia; o_Bacteroidales; f_S24-7; g_; s_	29.41%	35.71%	45.16%	41.82%	41.94%	51.43%	0.872	0.024	0.078
FJ681930.1.1456	p_Bacteroidetes; c_Bacteroidia; o_Bacteroidales; f_S24-7; g_; s_	8.82%	28.57%	32.26%	29.09%	48.39%	51.43%	0.937	0.006	0.036
JX095660.1.1399	p_Bacteroidetes; c_Bacteroidia; o_Bacteroidales; f_S24-7; g_; s_	91.18%	76.19%	67.74%	65.45%	61.29%	57.14%	-0.909	0.012	0.054
JX198578.1.1507	p_Bacteroidetes; c_Bacteroidia; o_Bacteroidales; f_S24-7; g_; s_	29.41%	33.33%	45.16%	43.64%	51.61%	60.00%	0.969	0.001	0.019
FJ681829.1.1459	p_Bacteroidetes; c_Bacteroidia; o_Bacteroidales; f_S24-7; g_; s_	29.41%	38.10%	54.84%	50.91%	45.16%	68.57%	0.836	0.038	0.107
FJ685382.1.1405	p_Bacteroidetes; c_Bacteroidia; o_Bacteroidales; f_S24-7; g_; s_	23.53%	47.62%	48.39%	49.09%	67.74%	77.14%	0.961	0.002	0.023
EF400759.1.1489	p_Bacteroidetes; c_Bacteroidia; o_Bacteroidales; f_S24-7; g_; s_	29.41%	47.62%	54.84%	60.00%	74.19%	88.57%	0.991	0.000	0.008
EU381832.1.1457	p_Chloroflexi; c_Anaerolineae; o_Anaerolineales; f_Anaerolinaceae; g_SHD-231; s_	29.41%	35.71%	45.16%	49.09%	51.61%	51.43%	0.879	0.021	0.077
GQ327480.1.1307	p_Chloroflexi; c_Anaerolineae; o_Anaerolineales; f_Anaerolinaceae; g_SHD-231; s_	52.94%	57.14%	64.52%	52.73%	64.52%	54.29%	0.117	0.825	0.873
EU466334.1.1381	p_Cyanobacteria; c_4C0d-2; o_YS2; f_; g_; s_	70.59%	54.76%	45.16%	38.18%	29.03%	17.14%	-0.982	0.000	0.012
LC028660.1.1513	p_Cyanobacteria; c_4C0d-2; o_YS2; f_; g_; s_	70.59%	59.52%	41.94%	27.27%	25.81%	25.71%	-0.874	0.023	0.078
CBEW010000180.8693.10203	p_Cyanobacteria; c_4C0d-2; o_YS2; f_; g_; s_	47.06%	52.38%	32.26%	34.55%	45.16%	31.43%	-0.543	0.266	0.406
AB614740.1.1482	p_Cyanobacteria; c_4C0d-2; o_YS2; f_; g_; s_	61.76%	69.05%	58.06%	50.91%	58.06%	45.71%	-0.764	0.077	0.170
AB746570.1.1514	p_Cyanobacteria; c_4C0d-2; o_YS2; f_; g_; s_	50.00%	54.76%	54.84%	56.36%	61.29%	57.14%	0.774	0.071	0.162
AB506276.1.1515	p_Cyanobacteria; c_4C0d-2; o_YS2; f_; g_; s_	52.94%	66.67%	70.97%	72.73%	87.10%	82.86%	0.907	0.013	0.055
AB034017.1.1505	p_Elusimicrobia; c_Elusimicrobia; o_Elusimicrobiales; f_Elusimicrobiaceae; g_; s_	50.00%	45.24%	54.84%	50.91%	61.29%	48.57%	0.261	0.617	0.722
HQ323426.1.1541	p_Firmicutes; c_Bacilli; o_Bacillales; f_Bacillaceae; g_Bacillus	94.12%	88.10%	87.10%	89.09%	93.55%	97.14%	0.534	0.275	0.416
JF825494.1.1570	p_Firmicutes; c_Bacilli; o_Bacillales; f_Bacillaceae; g_Bacillus; s_	52.94%	59.52%	48.39%	63.64%	67.74%	42.86%	-0.155	0.770	0.844
KF625180.1.1799	p_Firmicutes; c_Bacilli; o_Bacillales; f_Bacillaceae; g_Bacillus; s_	41.18%	50.00%	35.48%	45.45%	41.94%	45.71%	0.153	0.772	0.844
EU465848.1.1412	p_Firmicutes; c_Bacilli; o_Bacillales; f_Bacillaceae; g_Bacillus; s_	67.65%	54.76%	54.84%	47.27%	67.74%	51.43%	-0.287	0.581	0.698
LC028864.1.1544	p_Firmicutes; c_Bacilli; o_Bacillales; f_Bacillaceae; g_Bacillus; s_	91.18%	76.19%	77.42%	87.27%	93.55%	82.86%	0.109	0.838	0.882
KJ600949.1.1573	p_Firmicutes; c_Bacilli; o_Bacillales; f_Bacillaceae; g_Bacillus; s_	88.24%	92.86%	96.77%	90.91%	100.00%	94.29%	0.550	0.258	0.399
New.ReferenceOTU48	p_Firmicutes; c_Bacilli; o_Bacillales; f_Planococcaceae	64.71%	50.00%	38.71%	41.82%	45.16%	28.57%	-0.840	0.036	0.104
EF517948.1.1687	p_Firmicutes; c_Bacilli; o_Bacillales; f_Planococcaceae; g_Lysinibacillus	97.06%	88.10%	96.77%	92.73%	96.77%	94.29%	0.087	0.869	0.904
AB818493.1.1469	p_Firmicutes; c_Bacilli; o_Lactobacillales; f_Aerococcaceae; g_Facklamia; s_	38.24%	45.24%	32.26%	50.91%	35.48%	45.71%	0.210	0.689	0.782
DI206451.3.1491	p_Firmicutes; c_Bacilli; o_Lactobacillales; f_Lactobacillaceae; g_Lactobacillus; s_	35.29%	52.38%	38.71%	38.18%	38.71%	54.29%	0.462	0.356	0.498
AF197125.1.1555	p_Firmicutes; c_Bacilli; o_Lactobacillales; f_Lactobacillaceae; g_Lactobacillus; s_	38.24%	30.95%	32.26%	36.36%	54.84%	54.29%	0.811	0.050	0.128
New.ReferenceOTU123	p_Firmicutes; c_Bacilli; o_Lactobacillales; f_Lactobacillaceae; g_Lactobacillus; s_	38.24%	45.24%	48.39%	58.18%	51.61%	57.14%	0.819	0.046	0.122
LG042641.312212.313771	p_Firmicutes; c_Bacilli; o_Lactobacillales; f_Lactobacillaceae; g_Lactobacillus; s_	100.00%	97.62%	93.55%	89.09%	93.55%	97.14%	-0.280	0.591	0.704
AB559728.1.1569	p_Firmicutes; c_Bacilli; o_Lactobacillales; f_Lactobacillaceae; g_Lactobacillus; s_	100.00%	100.00%	100.00%	98.18%	100.00%	97.14%	-0.712	0.112	0.221
AB559642.1.1565	p_Firmicutes; c_Bacilli; o_Lactobacillales; f_Lactobacillaceae; g_Lactobacillus; s_	100.00%	100.00%	100.00%	98.18%	100.00%	100.00%	-0.019	0.972	0.982
New.ReferenceOTU136	p_Firmicutes; c_Bacilli; o_Lactobacillales; f_Lactobacillaceae; g_Lactobacillus; s_mucosae	55.88%	50.00%	48.39%	40.00%	45.16%	51.43%	-0.307	0.554	0.681
New.ReferenceOTU323	p_Firmicutes; c_Bacilli; o_Lactobacillales; f_Lactobacillaceae; g_Lactobacillus; s_reuteri	38.24%	42.86%	45.16%	38.18%	29.03%	51.43%	0.212	0.686	0.780
FJ879139.1.1535	p_Firmicutes; c_Bacilli; o_Lactobacillales; f_Lactobacillaceae; g_Lactobacillus; s_reuteri	41.18%	47.62%	64.52%	60.00%	74.19%	65.71%	0.798	0.057	0.137
AY363377.1.1576	p_Firmicutes; c_Bacilli; o_Lactobacillales; f_Lactobacillaceae; g_Lactobacillus; s_reuteri	100.00%	95.24%	96.77%	92.73%	100.00%	100.00%	0.268	0.608	0.717
AZJT01000071.1165.2796	p_Firmicutes; c_Bacilli; o_Lactobacillales; f_Streptococcaceae; g_Streptococcus; s_	91.18%	97.62%	80.65%	90.91%	90.32%	94.29%	0.128	0.809	0.867
New.ReferenceOTU247	p_Firmicutes; c_Bacilli; o_Turicibacterales; f_Turicibacteraceae; g_Turicibacter; s_	85.29%	76.19%	80.65%	81.82%	87.10%	94.29%	0.732	0.098	0.199
New.ReferenceOTU197	p_Firmicutes; c_Clostridia; o_Clostridiales	61.76%	28.57%	38.71%	38.18%	29.03%	34.29%	-0.582	0.226	0.365
EU794086.1.1446	p_Firmicutes; c_Clostridia; o_Clostridiales	94.12%	92.86%	100.00%	90.91%	96.77%	100.00%	0.502	0.310	0.454
LC028876.1.1506	p_Firmicutes; c_Clostridia; o_Clostridiales	100.00%	97.62%	100.00%	100.00%	96.77%	100.00%	-0.094	0.860	0.897
JX095388.1.1396	p_Firmicutes; c_Clostridia; o_Clostridiales; f_; g_; s_	50.00%	38.10%	32.26%	23.64%	19.35%	14.29%	-0.957	0.003	0.025
FPLO01008568.16.1523	p_Firmicutes; c_Clostridia; o_Clostridiales; f_; g_; s_	26.47%	28.57%	51.61%	45.45%	16.13%	17.14%	-0.430	0.394	0.540
New.CleanUp.ReferenceOTU43342	p_Firmicutes; c_Clostridia; o_Clostridiales; f_; g_; s_	52.94%	40.48%	48.39%	30.91%	32.26%	17.14%	-0.927	0.008	0.042
EU461015.1.1397	p_Firmicutes; c_Clostridia; o_Clostridiales; f_; g_; s_	50.00%	35.71%	35.48%	43.64%	32.26%	17.14%	-0.866	0.026	0.083
FJ825497.1.1371	p_Firmicutes; c_Clostridia; o_Clostridiales; f_; g_; s_	64.71%	35.71%	45.16%	36.36%	22.58%	22.86%	-0.871	0.024	0.078
GQ448612.1.1402	p_Firmicutes; c_Clostridia; o_Clostridiales; f_; g_; s_	50.00%	42.86%	48.39%	29.09%	38.71%	22.86%	-0.836	0.038	0.107
GQ159307.1.1468	p_Firmicutes; c_Clostridia; o_Clostridiales; f_; g_; s_	58.82%	42.86%	67.74%	41.82%	48.39%	22.86%	-0.720	0.106	0.211
EU779286.1.1369	p_Firmicutes; c_Clostridia; o_Clostridiales; f_; g_; s_	55.88%	52.38%	41.94%	34.55%	35.48%	25.71%	-0.945	0.005	0.032
AB626922.1.1486	p_Firmicutes; c_Clostridia; o_Clostridiales; f_; g_; s_	70.59%	50.00%	51.61%	45.45%	38.71%	25.71%	-0.962	0.002	0.023
EU469250.1.1356	p_Firmicutes; c_Clostridia; o_Clostridiales; f_; g_; s_	47.06%	23.81%	51.61%	52.73%	38.71%	25.71%	-0.383	0.453	0.595
GQ451299.1.1535	p_Firmicutes; c_Clostridia; o_Clostridiales; f_; g_; s_	52.94%	42.86%	61.29%	40.00%	41.94%	25.71%	-0.765	0.077	0.170
New.CleanUp.ReferenceOTU39291	p_Firmicutes; c_Clostridia; o_Clostridiales; f_; g_; s_	55.88%	54.76%	58.06%	41.82%	48.39%	25.71%	-0.857	0.029	0.088
JX094971.1.1381	p_Firmicutes; c_Clostridia; o_Clostridiales; f_; g_; s_	64.71%	59.52%	54.84%	49.09%	41.94%	28.57%	-0.996	0.000	0.008
New.CleanUp.ReferenceOTU28437	p_Firmicutes; c_Clostridia; o_Clostridiales; f_; g_; s_	58.82%	57.14%	64.52%	38.18%	41.94%	31.43%	-0.842	0.036	0.102
New.ReferenceOTU312	p_Firmicutes; c_Clostridia; o_Clostridiales; f_; g_; s_	52.94%	47.62%	51.61%	41.82%	45.16%	31.43%	-0.889	0.018	0.068
New.ReferenceOTU6	p_Firmicutes; c_Clostridia; o_Clostridiales; f_; g_; s_	50.00%	42.86%	48.39%	40.00%	51.61%	31.43%	-0.588	0.220	0.358
New.ReferenceOTU7	p_Firmicutes; c_Clostridia; o_Clostridiales; f_; g_; s_	64.71%	66.67%	67.74%	49.09%	61.29%	31.43%	-0.798	0.057	0.137
AB746750.1.1530	p_Firmicutes; c_Clostridia; o_Clostridiales; f_; g_; s_	44.12%	54.76%	41.94%	32.73%	32.26%	34.29%	-0.692	0.128	0.244
AB185594.1.1530	p_Firmicutes; c_Clostridia; o_Clostridiales; f_; g_; s_	58.82%	40.48%	38.71%	41.82%	38.71%	34.29%	-0.769	0.074	0.166
EU844699.1.1555	p_Firmicutes; c_Clostridia; o_Clostridiales; f_; g_; s_	47.06%	40.48%	41.94%	41.82%	51.61%	34.29%	-0.346	0.501	0.637
JQ084476.1.1482	p_Firmicutes; c_Clostridia; o_Clostridiales; f_; g_; s_	38.24%	33.33%	41.94%	52.73%	51.61%	34.29%	0.141	0.790	0.851
EU381553.1.1508	p_Firmicutes; c_Clostridia; o_Clostridiales; f_; g_; s_	64.71%	52.38%	74.19%	45.45%	61.29%	34.29%	-0.628	0.182	0.314
JX095608.1.1380	p_Firmicutes; c_Clostridia; o_Clostridiales; f_; g_; s_	58.82%	59.52%	51.61%	36.36%	35.48%	37.14%	-0.846	0.034	0.098
EU771517.1.1377	p_Firmicutes; c_Clostridia; o_Clostridiales; f_; g_; s_	41.18%	54.76%	41.94%	36.36%	38.71%	37.14%	-0.519	0.292	0.431
EU779161.1.1394	p_Firmicutes; c_Clostridia; o_Clostridiales; f_; g_; s_	76.47%	66.67%	54.84%	41.82%	45.16%	37.14%	-0.906	0.013	0.055
LC028566.1.1509	p_Firmicutes; c_Clostridia; o_Clostridiales; f_; g_; s_	61.76%	59.52%	54.84%	50.91%	45.16%	40.00%	-0.991	0.000	0.008
EU382028.1.1510	p_Firmicutes; c_Clostridia; o_Clostridiales; f_; g_; s_	67.65%	59.52%	61.29%	47.27%	54.84%	40.00%	-0.886	0.019	0.071
KR514451.1.1443	p_Firmicutes; c_Clostridia; o_Clostridiales; f_; g_; s_	61.76%	45.24%	41.94%	30.91%	35.48%	42.86%	-0.576	0.231	0.371
FJ848448.1.1487	p_Firmicutes; c_Clostridia; o_Clostridiales; f_; g_; s_	67.65%	57.14%	64.52%	54.55%	54.84%	42.86%	-0.904	0.013	0.057
FJ680604.1.1375	p_Firmicutes; c_Clostridia; o_Clostridiales; f_; g_; s_	58.82%	45.24%	58.06%	43.64%	38.71%	45.71%	-0.624	0.186	0.319
EU845654.1.1551	p_Firmicutes; c_Clostridia; o_Clostridiales; f_; g_; s_	64.71%	45.24%	64.52%	52.73%	45.16%	45.71%	-0.643	0.169	0.297
AB185631.1.1534	p_Firmicutes; c_Clostridia; o_Clostridiales; f_; g_; s_	35.29%	50.00%	45.16%	43.64%	48.39%	45.71%	0.470	0.346	0.488
New.CleanUp.ReferenceOTU984	p_Firmicutes; c_Clostridia; o_Clostridiales; f_; g_; s_	58.82%	42.86%	51.61%	38.18%	51.61%	45.71%	-0.343	0.505	0.639
AB185646.1.1535	p_Firmicutes; c_Clostridia; o_Clostridiales; f_; g_; s_	58.82%	35.71%	48.39%	32.73%	54.84%	45.71%	-0.089	0.867	0.903
New.ReferenceOTU363	p_Firmicutes; c_Clostridia; o_Clostridiales; f_; g_; s_	79.41%	45.24%	70.97%	56.36%	54.84%	45.71%	-0.638	0.173	0.303
KC991122.1.1441	p_Firmicutes; c_Clostridia; o_Clostridiales; f_; g_; s_	76.47%	57.14%	64.52%	56.36%	61.29%	45.71%	-0.813	0.049	0.127
New.CleanUp.ReferenceOTU16067	p_Firmicutes; c_Clostridia; o_Clostridiales; f_; g_; s_	38.24%	40.48%	58.06%	40.00%	54.84%	48.57%	0.451	0.369	0.513
KR068191.1.1488	p_Firmicutes; c_Clostridia; o_Clostridiales; f_; g_; s_	58.82%	35.71%	48.39%	50.91%	58.06%	48.57%	0.067	0.900	0.928
New.ReferenceOTU201	p_Firmicutes; c_Clostridia; o_Clostridiales; f_; g_; s_	50.00%	35.71%	58.06%	43.64%	41.94%	51.43%	0.073	0.890	0.921
EF031001.1.1547	p_Firmicutes; c_Clostridia; o_Clostridiales; f_; g_; s_	50.00%	30.95%	48.39%	41.82%	45.16%	51.43%	0.315	0.544	0.670
EU381730.1.1481	p_Firmicutes; c_Clostridia; o_Clostridiales; f_; g_; s_	44.12%	50.00%	51.61%	45.45%	54.84%	51.43%	0.601	0.207	0.343
New.CleanUp.ReferenceOTU30648	p_Firmicutes; c_Clostridia; o_Clostridiales; f_; g_; s_	44.12%	50.00%	38.71%	52.73%	54.84%	51.43%	0.567	0.241	0.380
AY854296.1.1528	p_Firmicutes; c_Clostridia; o_Clostridiales; f_; g_; s_	79.41%	64.29%	77.42%	52.73%	54.84%	51.43%	-0.807	0.052	0.132
AY854369.1.1517	p_Firmicutes; c_Clostridia; o_Clostridiales; f_; g_; s_	64.71%	50.00%	54.84%	56.36%	77.42%	51.43%	0.036	0.946	0.963
EU382057.1.1511	p_Firmicutes; c_Clostridia; o_Clostridiales; f_; g_; s_	79.41%	66.67%	74.19%	65.45%	77.42%	51.43%	-0.666	0.148	0.274
EU509838.1.1378	p_Firmicutes; c_Clostridia; o_Clostridiales; f_; g_; s_	67.65%	57.14%	80.65%	58.18%	54.84%	54.29%	-0.538	0.271	0.413
FJ825557.1.1415	p_Firmicutes; c_Clostridia; o_Clostridiales; f_; g_; s_	67.65%	38.10%	70.97%	41.82%	58.06%	54.29%	-0.144	0.785	0.849
LC028750.1.1504	p_Firmicutes; c_Clostridia; o_Clostridiales; f_; g_; s_	67.65%	54.76%	58.06%	49.09%	61.29%	54.29%	-0.422	0.404	0.548
New.ReferenceOTU75	p_Firmicutes; c_Clostridia; o_Clostridiales; f_; g_; s_	70.59%	47.62%	70.97%	40.00%	64.52%	54.29%	-0.238	0.650	0.755
KC991134.1.1450	p_Firmicutes; c_Clostridia; o_Clostridiales; f_; g_; s_	79.41%	59.52%	77.42%	54.55%	64.52%	54.29%	-0.648	0.164	0.293
JX095424.1.1392	p_Firmicutes; c_Clostridia; o_Clostridiales; f_; g_; s_	85.29%	59.52%	70.97%	60.00%	67.74%	54.29%	-0.693	0.127	0.243

DQ394604.1.1560	p_Firmicutes; c_Clostridia; o_Clostridiales; f_; g_; s_	55.88%	35.71%	45.16%	50.91%	54.84%	57.14%	0.451	0.370	0.513
AB821810.1.1528	p_Firmicutes; c_Clostridia; o_Clostridiales; f_; g_; s_	70.59%	57.14%	54.84%	58.18%	54.84%	57.14%	-0.590	0.218	0.356
New.CleanUp.ReferenceOTU38208	p_Firmicutes; c_Clostridia; o_Clostridiales; f_; g_; s_	41.18%	42.86%	54.84%	27.27%	58.06%	57.14%	0.495	0.318	0.463
DQ394666.1.1561	p_Firmicutes; c_Clostridia; o_Clostridiales; f_; g_; s_	32.35%	33.33%	48.39%	40.00%	61.29%	57.14%	0.859	0.028	0.087
New.CleanUp.ReferenceOTU25162	p_Firmicutes; c_Clostridia; o_Clostridiales; f_; g_; s_	50.00%	40.48%	41.94%	54.55%	54.84%	60.00%	0.732	0.098	0.199
DQ394667.1.1517	p_Firmicutes; c_Clostridia; o_Clostridiales; f_; g_; s_	58.82%	57.14%	67.74%	67.27%	54.84%	60.00%	-0.105	0.842	0.884
AB606277.1.1532	p_Firmicutes; c_Clostridia; o_Clostridiales; f_; g_; s_	64.71%	47.62%	67.74%	30.91%	61.29%	60.00%	-0.008	0.988	0.993
New.CleanUp.ReferenceOTU38487	p_Firmicutes; c_Clostridia; o_Clostridiales; f_; g_; s_	41.18%	38.10%	64.52%	45.45%	61.29%	60.00%	0.643	0.169	0.297
DQ116004.1.1477	p_Firmicutes; c_Clostridia; o_Clostridiales; f_; g_; s_	79.41%	64.29%	74.19%	60.00%	61.29%	60.00%	-0.757	0.081	0.175
AB185619.1.1533	p_Firmicutes; c_Clostridia; o_Clostridiales; f_; g_; s_	61.76%	42.86%	61.29%	67.27%	64.52%	60.00%	0.287	0.581	0.698
New.ReferenceOTU313	p_Firmicutes; c_Clostridia; o_Clostridiales; f_; g_; s_	79.41%	80.95%	77.42%	67.27%	64.52%	60.00%	-0.937	0.006	0.036
CEAH01023145.29.1558	p_Firmicutes; c_Clostridia; o_Clostridiales; f_; g_; s_	32.35%	35.71%	35.48%	50.91%	67.74%	60.00%	0.879	0.021	0.077
LC028715.1.1513	p_Firmicutes; c_Clostridia; o_Clostridiales; f_; g_; s_	88.24%	78.57%	87.10%	74.55%	70.97%	60.00%	-0.929	0.007	0.042
DQ394597.1.1566	p_Firmicutes; c_Clostridia; o_Clostridiales; f_; g_; s_	70.59%	50.00%	67.74%	65.45%	77.42%	60.00%	0.071	0.894	0.923
FN667249.1.1454	p_Firmicutes; c_Clostridia; o_Clostridiales; f_; g_; s_	20.59%	21.43%	35.48%	27.27%	45.16%	62.86%	0.939	0.005	0.035
EF436353.1.1501	p_Firmicutes; c_Clostridia; o_Clostridiales; f_; g_; s_	61.76%	52.38%	54.84%	58.18%	61.29%	62.86%	0.478	0.337	0.481
AB185553.1.1555	p_Firmicutes; c_Clostridia; o_Clostridiales; f_; g_; s_	58.82%	54.76%	41.94%	60.00%	48.39%	65.71%	0.282	0.589	0.704
New.ReferenceOTU226	p_Firmicutes; c_Clostridia; o_Clostridiales; f_; g_; s_	47.06%	52.38%	64.52%	52.73%	54.84%	65.71%	0.663	0.152	0.279
EF406864.1.1535	p_Firmicutes; c_Clostridia; o_Clostridiales; f_; g_; s_	58.82%	35.71%	48.39%	49.09%	67.74%	65.71%	0.591	0.217	0.356
New.ReferenceOTU355	p_Firmicutes; c_Clostridia; o_Clostridiales; f_; g_; s_	64.71%	64.29%	77.42%	63.64%	51.61%	68.57%	-0.186	0.724	0.807
GQ358479.1.1399	p_Firmicutes; c_Clostridia; o_Clostridiales; f_; g_; s_	52.94%	59.52%	77.42%	65.45%	58.06%	71.43%	0.420	0.407	0.550
DQ394601.1.1564	p_Firmicutes; c_Clostridia; o_Clostridiales; f_; g_; s_	64.71%	59.52%	58.06%	58.18%	64.52%	71.43%	0.609	0.200	0.334
AB185804.1.1535	p_Firmicutes; c_Clostridia; o_Clostridiales; f_; g_; s_	85.29%	76.19%	83.87%	78.18%	74.19%	71.43%	-0.836	0.038	0.106
KC162944.1.1530	p_Firmicutes; c_Clostridia; o_Clostridiales; f_; g_; s_	85.29%	66.67%	83.87%	70.91%	83.87%	71.43%	-0.237	0.650	0.755
DQ288680.1.1547	p_Firmicutes; c_Clostridia; o_Clostridiales; f_; g_; s_	58.82%	76.19%	70.97%	65.45%	74.19%	74.29%	0.565	0.243	0.382
FJ848396.1.1484	p_Firmicutes; c_Clostridia; o_Clostridiales; f_; g_; s_	73.53%	66.67%	80.65%	74.55%	80.65%	74.29%	0.318	0.539	0.668
DQ394600.1.1565	p_Firmicutes; c_Clostridia; o_Clostridiales; f_; g_; s_	76.47%	64.29%	70.97%	74.55%	90.32%	74.29%	0.388	0.448	0.592
AB627477.1.1526	p_Firmicutes; c_Clostridia; o_Clostridiales; f_; g_; s_	94.12%	73.81%	87.10%	74.55%	74.19%	77.14%	-0.583	0.225	0.364
LC028569.1.1518	p_Firmicutes; c_Clostridia; o_Clostridiales; f_; g_; s_	82.35%	71.43%	90.32%	83.64%	87.10%	77.14%	-0.003	0.995	0.997
DQ394661.1.1545	p_Firmicutes; c_Clostridia; o_Clostridiales; f_; g_; s_	82.35%	73.81%	70.97%	72.73%	90.32%	80.00%	0.316	0.541	0.669
FJ684114.1.1428	p_Firmicutes; c_Clostridia; o_Clostridiales; f_; g_; s_	64.71%	54.76%	61.29%	52.73%	64.52%	82.86%	0.657	0.156	0.286
EU779246.1.1387	p_Firmicutes; c_Clostridia; o_Clostridiales; f_; g_; s_	85.29%	66.67%	83.87%	76.36%	70.97%	82.86%	-0.031	0.953	0.969
New.CleanUp.ReferenceOTU43470	p_Firmicutes; c_Clostridia; o_Clostridiales; f_; g_; s_	76.47%	73.81%	67.74%	80.00%	70.97%	82.86%	0.391	0.444	0.589
New.ReferenceOTU70	p_Firmicutes; c_Clostridia; o_Clostridiales; f_; g_; s_	82.35%	83.33%	100.00%	78.18%	80.65%	82.86%	-0.198	0.708	0.798
DQ394665.1.1563	p_Firmicutes; c_Clostridia; o_Clostridiales; f_; g_; s_	85.29%	78.57%	90.32%	70.91%	83.87%	85.71%	0.052	0.921	0.945
New.ReferenceOTU142	p_Firmicutes; c_Clostridia; o_Clostridiales; f_; g_; s_	79.41%	76.19%	80.65%	80.00%	87.10%	85.71%	0.821	0.045	0.121
AB185711.1.1545	p_Firmicutes; c_Clostridia; o_Clostridiales; f_; g_; s_	88.24%	90.48%	93.55%	90.91%	87.10%	85.71%	-0.561	0.246	0.384
EF686530.1.1533	p_Firmicutes; c_Clostridia; o_Clostridiales; f_; g_; s_	82.35%	80.95%	96.77%	74.55%	90.32%	85.71%	0.160	0.762	0.836
LC028883.1.1510	p_Firmicutes; c_Clostridia; o_Clostridiales; f_; g_; s_	91.18%	85.71%	96.77%	80.00%	90.32%	85.71%	-0.270	0.605	0.716
KR514383.1.1445	p_Firmicutes; c_Clostridia; o_Clostridiales; f_; g_; s_	91.18%	85.71%	93.55%	81.82%	90.32%	85.71%	-0.300	0.564	0.686
JX096108.1.1389	p_Firmicutes; c_Clostridia; o_Clostridiales; f_; g_; s_	73.53%	73.81%	87.10%	70.91%	77.42%	88.57%	0.550	0.258	0.399
LC028802.1.1535	p_Firmicutes; c_Clostridia; o_Clostridiales; f_; g_; s_	91.18%	90.48%	100.00%	85.45%	87.10%	88.57%	-0.385	0.451	0.595
LC028720.1.1523	p_Firmicutes; c_Clostridia; o_Clostridiales; f_; g_; s_	94.12%	92.86%	93.55%	89.09%	93.55%	88.57%	-0.655	0.158	0.288
FJ374207.1.1547	p_Firmicutes; c_Clostridia; o_Clostridiales; f_; g_; s_	97.06%	92.86%	100.00%	92.73%	93.55%	91.43%	-0.587	0.221	0.359
AB506415.1.1518	p_Firmicutes; c_Clostridia; o_Clostridiales; f_; g_; s_	91.18%	95.24%	100.00%	96.36%	93.55%	91.43%	-0.221	0.674	0.770
FJ879989.1.1495	p_Firmicutes; c_Clostridia; o_Clostridiales; f_; g_; s_	94.12%	73.81%	87.10%	89.09%	96.77%	91.43%	0.342	0.507	0.639
AY854349.1.1558	p_Firmicutes; c_Clostridia; o_Clostridiales; f_; g_; s_	94.12%	88.10%	80.65%	92.73%	96.77%	91.43%	0.235	0.653	0.757
LC028709.1.1507	p_Firmicutes; c_Clostridia; o_Clostridiales; f_; g_; s_	94.12%	95.24%	100.00%	89.09%	96.77%	97.14%	0.187	0.723	0.807
AB185689.1.1554	p_Firmicutes; c_Clostridia; o_Clostridiales; f_; g_; s_	97.06%	97.62%	100.00%	94.55%	96.77%	97.14%	-0.185	0.725	0.807
AY858424.1.1561	p_Firmicutes; c_Clostridia; o_Clostridiales; f_; g_; s_	73.53%	83.33%	100.00%	94.55%	93.55%	100.00%	0.755	0.083	0.178
AB506408.1.1519	p_Firmicutes; c_Clostridia; o_Clostridiales; f_; g_; s_	97.06%	97.62%	100.00%	96.36%	96.77%	100.00%	0.349	0.498	0.634
AB746647.1.1559	p_Firmicutes; c_Clostridia; o_Clostridiales; f_; g_; s_	94.12%	90.48%	90.32%	90.91%	100.00%	100.00%	0.732	0.098	0.199
KP150938.1.1495	p_Firmicutes; c_Clostridia; o_Clostridiales; f_; g_; s_	100.00%	100.00%	100.00%	96.36%	100.00%	100.00%	-0.019	0.972	0.982
AB185667.1.1557	p_Firmicutes; c_Clostridia; o_Clostridiales; f_; g_; s_	97.06%	100.00%	100.00%	98.18%	100.00%	100.00%	0.562	0.246	0.384
EF405418.1.1497	p_Firmicutes; c_Clostridia; o_Clostridiales; f_; g_; s_	100.00%	100.00%	100.00%	100.00%	100.00%	100.00%	NA	NA	NA
New.CleanUp.ReferenceOTU23840	p_Firmicutes; c_Clostridia; o_Clostridiales; f_[Mogibacteriaceae]; g_; s_	58.82%	50.00%	41.94%	41.82%	29.03%	8.57%	-0.985	0.000	0.011
EU472609.1.1386	p_Firmicutes; c_Clostridia; o_Clostridiales; f_[Mogibacteriaceae]; g_; s_	55.88%	52.38%	58.06%	30.91%	41.94%	31.43%	-0.772	0.072	0.164
JX096259.1.1387	p_Firmicutes; c_Clostridia; o_Clostridiales; f_[Mogibacteriaceae]; g_; s_	64.71%	59.52%	67.74%	56.36%	67.74%	51.43%	-0.487	0.327	0.471
JX095236.1.1385	p_Firmicutes; c_Clostridia; o_Clostridiales; f_[Mogibacteriaceae]; g_; s_	8.82%	23.81%	35.48%	32.73%	29.03%	54.29%	0.875	0.023	0.078
AB506296.1.1510	p_Firmicutes; c_Clostridia; o_Clostridiales; f_[Mogibacteriaceae]; g_; s_	61.76%	54.76%	77.42%	67.27%	58.06%	74.29%	0.350	0.496	0.634
JX095091.1.1392	p_Firmicutes; c_Clostridia; o_Clostridiales; f_[Mogibacteriaceae]; g_; s_	94.12%	83.33%	96.77%	90.91%	90.32%	82.86%	-0.490	0.324	0.468
LC028890.1.1510	p_Firmicutes; c_Clostridia; o_Clostridiales; f_[Mogibacteriaceae]; g_; s_	94.12%	90.48%	100.00%	89.09%	77.42%	85.71%	-0.644	0.167	0.297
New.ReferenceOTU285	p_Firmicutes; c_Clostridia; o_Clostridiales; f_[Mogibacteriaceae]; g_Anaerovorax; s_	47.06%	50.00%	41.94%	29.09%	48.39%	42.86%	-0.166	0.753	0.830
AB034014.1.1518	p_Firmicutes; c_Clostridia; o_Clostridiales; f_[Mogibacteriaceae]; g_Mogibacterium; s_	79.41%	61.90%	51.61%	67.27%	67.74%	62.86%	-0.269	0.606	0.716
AB270089.1.1512	p_Firmicutes; c_Clostridia; o_Clostridiales; f_[Mogibacteriaceae]; g_Mogibacterium; s_	94.12%	95.24%	96.77%	96.36%	93.55%	100.00%	0.603	0.205	0.342
New.ReferenceOTU112	p_Firmicutes; c_Clostridia; o_Clostridiales; f_[Tissierellaceae]; g_Anaerococcus; s_	47.06%	42.86%	35.48%	50.91%	48.39%	48.57%	0.374	0.465	0.605
HQ780809.1.1449	p_Firmicutes; c_Clostridia; o_Clostridiales; f_Christensenellaceae; g_; s_	52.94%	33.33%	48.39%	36.36%	25.81%	20.00%	-0.875	0.022	0.078
New.CleanUp.ReferenceOTU19990	p_Firmicutes; c_Clostridia; o_Clostridiales; f_Christensenellaceae; g_; s_	52.94%	47.62%	51.61%	43.64%	35.48%	37.14%	-0.901	0.014	0.059
AB746648.1.1512	p_Firmicutes; c_Clostridia; o_Clostridiales; f_Christensenellaceae; g_; s_	35.29%	47.62%	25.81%	32.73%	48.39%	51.43%	0.563	0.245	0.383
FJ880924.1.1487	p_Firmicutes; c_Clostridia; o_Clostridiales; f_Christensenellaceae; g_; s_	85.29%	73.81%	77.42%	72.73%	83.87%	65.71%	-0.569	0.238	0.379
AY337519.1.1512	p_Firmicutes; c_Clostridia; o_Clostridiales; f_Clostridiaceae; g_; s_	41.18%	59.52%	35.48%	27.27%	41.94%	40.00%	-0.228	0.664	0.761
New.CleanUp.ReferenceOTU15637	p_Firmicutes; c_Clostridia; o_Clostridiales; f_Clostridiaceae; g_; s_	55.88%	52.38%	54.84%	45.45%	41.94%	48.57%	-0.683	0.135	0.254
AB824582.1.1510	p_Firmicutes; c_Clostridia; o_Clostridiales; f_Clostridiaceae; g_; s_	38.24%	35.71%	41.94%	40.00%	35.48%	51.43%	0.633	0.178	0.308
EU779105.1.1385	p_Firmicutes; c_Clostridia; o_Clostridiales; f_Clostridiaceae; g_; s_	91.18%	76.19%	83.87%	63.64%	67.74%	62.86%	-0.838	0.037	0.105
FJ685180.1.1412	p_Firmicutes; c_Clostridia; o_Clostridiales; f_Clostridiaceae; g_; s_	76.47%	80.95%	67.74%	76.36%	64.52%	77.14%	-0.232	0.659	0.759
AB506357.1.1508	p_Firmicutes; c_Clostridia; o_Clostridiales; f_Clostridiaceae; g_; s_	100.00%	97.62%	96.77%	89.09%	93.55%	97.14%	-0.344	0.504	0.639
LC028615.1.1508	p_Firmicutes; c_Clostridia; o_Clostridiales; f_Clostridiaceae; g_; s_	97.06%	97.62%	100.00%	98.18%	96.77%	100.00%	0.408	0.422	0.565
JX841326.1.1525	p_Firmicutes; c_Clostridia; o_Clostridiales; f_Clostridiaceae; g_Clostridium; s_	50.00%	45.24%	38.71%	41.82%	45.16%	34.29%	-0.742	0.091	0.191
LC028700.1.1527	p_Firmicutes; c_Clostridia; o_Clostridiales; f_Clostridiaceae; g_Clostridium; s_	50.00%	59.52%	64.52%	56.36%	41.94%	48.57%	-0.473	0.343	0.486
LC028798.1.1527	p_Firmicutes; c_Clostridia; o_Clostridiales; f_Clostridiaceae; g_Clostridium; s_	64.71%	59.52%	64.52%	47.27%	48.39%	48.57%	-0.802	0.055	0.134
LC028811.1.1525	p_Firmicutes; c_Clostridia; o_Clostridiales; f_Clostridiaceae; g_Clostridium; s_	82.35%	71.43%	74.19%	63.64%	70.97%	57.14%	-0.854	0.031	0.090
AB627668.1.1507	p_Firmicutes; c_Clostridia; o_Clostridiales; f_Clostridiaceae; g_Clostridium; s_	70.59%	47.62%	51.61%	54.55%	51.61%	62.86%	-0.083	0.876	0.908
LC028742.1.1523	p_Firmicutes; c_Clostridia; o_Clostridiales; f_Clostridiaceae; g_Clostridium; s_	97.06%	97.62%	100.00%	98.18%	100.00%	100.00%	0.769	0.074	0.166
EU774653.1.1430	p_Firmicutes; c_Clostridia; o_Clostridiales; f_Dehalobacteriaceae; g_; s_	50.00%	50.00%	38.71%	49.09%	48.39%	45.71%	-0.167	0.751	0.828
AB506523.1.1525	p_Firmicutes; c_Clostridia; o_Clostridiales; f_Lachnospiraceae	76.47%	69.05%	61.29%	56.36%	54.84%	40.00%	-0.977	0.001	0.016
LNAM01000152.36752.38349	p_Firmicutes; c_Clostridia; o_Clostridiales; f_Lachnospiraceae	50.00%	21.43%	35.48%	32.73%	58.06%	51.43%	0.459	0.360	0.503
GQ448658.1.1404	p_Firmicutes; c_Clostridia; o_Clostridiales; f_Lachnospiraceae	47.06%	52.38%	58.06%	52.73%	45.16%	60.00%	0.381	0.456	0.598
AB506127.1.1523	p_Firmicutes; c_Clostridia; o_Clostridiales; f_Lachnospiraceae	97.06%	90.48%	70.97%	83.64%	77.42%	88.57%	-0.288	0.580	0.698
New.CleanUp.ReferenceOTU31195	p_Firmicutes; c_Clostridia; o_Clostridiales; f_Lachnospiraceae; g_; s_	50.00%	23.81%	25.81%	18.18%	25.81%	17.14%	-0.708	0.116	0.226
New.CleanUp.ReferenceOTU47135	p_Firmicutes; c_Clostridia; o_Clostridiales; f_Lachnospiraceae; g_; s_	67.65%	47.62%	58.06%	32.73%	32.26%	17.14%	-0.932	0.007	0.039
New.ReferenceOTU173	p_Firmicutes; c_Clostridia; o_Clostridiales; f_Lachnospiraceae; g_; s_	50.00%	26.19%	35.48%	23.64%	16.13%	20.00%	-0.805	0.053	0.132
AB627660.1.1525	p_Firmicutes; c_Clostridia; o_Clostridiales; f_Lachnospiraceae; g_; s_	55.88%	35.71%	35.48%	16.36%	22.58%	22.86%	-0.771	0.073	0.165
EU772005.1.1306	p_Firmicutes; c_Clostridia; o_Clostridiales; f_Lachnospiraceae; g_; s_	50.00%	40.48%	35.48%	30.91%	32.26%	22.86%	-0.938	0.006	0.036
DQ115991.1.1485	p_Firmicutes; c_Clostridia; o_Clostridiales; f_Lachnospiraceae; g_; s_	64.71%	50.00%	35.48%	34.55%	41.94%	28.57%	-0.798	0.057	0.137
New.ReferenceOTU10	p_Firmicutes; c_Clostridia; o_Clostridiales; f_Lachnospiraceae; g_; s_	58.82%	38.10%	32.26%	43.64%	45.16%	28.57%	-0.613	0.195	0.330
AB746664.1.1536	p_Firmicutes; c_Clostridia; o_Clostridiales; f_Lachnospiraceae; g_; s_	32.35%	40.48%	51.61%	34.55%	45.16%	34.29%	-0.009	0.986	0.992
EU794106.1.1444	p_Firmicutes; c_Clostridia; o_Clostridiales; f_Lachnospiraceae; g_; s_	44.12%	35.71%	45.16%	30.91%	54.84%	34.29%	-0.061	0.909	0.936
LC028659.1.1516	p_Firmicutes; c_Clostridia; o_Clostridiales; f_Lachnospiraceae; g_; s_	35.29%	30.95%	58.06%	29.09%	29.03%	37.14%	-0.131	0.805	0.866
JX096140.1.1395	p_Firmicutes; c_Clostridia; o_Clostridiales; f_Lachnospiraceae; g_; s_	67.65%	57.14%	51.61%	58.18%	54.84%	37.14%	-0.864	0.026	0.084
New.CleanUp.ReferenceOTU7806	p_Firmicutes; c_Clostridia; o_Clostridiales; f_Lachnospiraceae; g_; s_	47.06%	28.57%	51.61%	40.00%	22.58%	40.00%	-0.323	0.533	0.662
New.CleanUp.ReferenceOTU3620	p_Firmicutes; c_Clostridia; o_Clostridiales; f_Lachnospiraceae; g_; s_	55.88%	42.86%	45.16%	41.82%	35.48%	40.00%	-0.778	0.068	0.159
New.ReferenceOTU28	p_Firmicutes; c_Clostridia; o_Clostridiales; f_Lachnospiraceae; g_; s_	32.35%	40.48%	41.94%	47.27%	58.06%	40.00%	0.500	0.313	0.457
JNLE01000003.1151759.1153290	p_Firmicutes; c_Clostridia; o_Clostridiales; f_Lachnospiraceae; g_; s_	52.94%	45.24%	51.61%	45.45%	61.29%	42.86%	-0.122	0.819	0.871
HQ815183.1.1447	p_Firmicutes; c_Clostridia; o_Clostridiales; f_Lachnospiraceae; g_; s_	38.24%	40.48%	54.84%	50.91%	29.03%	48.57%	0.047	0.930	0.950
DQ905930.1.1794	p_Firmicutes; c_Clostridia; o_Clostridiales; f_Lachnospiraceae; g_; s_	17.65%	30.95%	12.90%	36.36%	19.35%	51.43%	0.646	0.166	0.295

FJ679794.1.1456	p_Firmicutes; c_Clostridia; o_Clostridiales; f_Lachnospiraceae; g_; s_	52.94%	42.86%	51.61%	41.82%	45.16%	51.43%	-0.040	0.939	0.959
GQ897987.1.1492	p_Firmicutes; c_Clostridia; o_Clostridiales; f_Lachnospiraceae; g_; s_	58.82%	50.00%	51.61%	60.00%	58.06%	51.43%	-0.147	0.781	0.847
EU466182.1.1382	p_Firmicutes; c_Clostridia; o_Clostridiales; f_Lachnospiraceae; g_; s_	67.65%	66.67%	64.52%	60.00%	74.19%	51.43%	-0.485	0.330	0.473
AB034064.1.1528	p_Firmicutes; c_Clostridia; o_Clostridiales; f_Lachnospiraceae; g_; s_	52.94%	35.71%	54.84%	41.82%	35.48%	54.29%	0.003	0.995	0.997
EU772076.1.1400	p_Firmicutes; c_Clostridia; o_Clostridiales; f_Lachnospiraceae; g_; s_	64.71%	64.29%	51.61%	69.09%	48.39%	54.29%	-0.534	0.275	0.416
EU773474.1.1403	p_Firmicutes; c_Clostridia; o_Clostridiales; f_Lachnospiraceae; g_; s_	73.53%	54.76%	67.74%	74.55%	54.84%	54.29%	-0.574	0.234	0.374
AB507484.1.1519	p_Firmicutes; c_Clostridia; o_Clostridiales; f_Lachnospiraceae; g_; s_	55.88%	52.38%	54.84%	61.82%	58.06%	54.29%	0.124	0.814	0.870
FMEB01000008.156586.158100	p_Firmicutes; c_Clostridia; o_Clostridiales; f_Lachnospiraceae; g_; s_	20.59%	11.90%	12.90%	27.27%	35.48%	57.14%	0.889	0.018	0.068
FPLO01000585.119.1630	p_Firmicutes; c_Clostridia; o_Clostridiales; f_Lachnospiraceae; g_; s_	17.65%	40.48%	29.03%	30.91%	41.94%	60.00%	0.877	0.022	0.077
New.ReferenceOTU256	p_Firmicutes; c_Clostridia; o_Clostridiales; f_Lachnospiraceae; g_; s_	64.71%	50.00%	64.52%	56.36%	45.16%	60.00%	-0.289	0.579	0.698
FPLO01000921.10.1524	p_Firmicutes; c_Clostridia; o_Clostridiales; f_Lachnospiraceae; g_; s_	41.18%	38.10%	41.94%	38.18%	54.84%	60.00%	0.862	0.027	0.085
New.ReferenceOTU245	p_Firmicutes; c_Clostridia; o_Clostridiales; f_Lachnospiraceae; g_; s_	35.29%	42.86%	41.94%	58.18%	54.84%	60.00%	0.890	0.017	0.068
EU461086.1.1389	p_Firmicutes; c_Clostridia; o_Clostridiales; f_Lachnospiraceae; g_; s_	23.53%	19.05%	19.35%	23.64%	41.94%	62.86%	0.895	0.016	0.063
FJ172888.1.1497	p_Firmicutes; c_Clostridia; o_Clostridiales; f_Lachnospiraceae; g_; s_	76.47%	66.67%	67.74%	60.00%	58.06%	62.86%	-0.750	0.086	0.183
New.ReferenceOTU242	p_Firmicutes; c_Clostridia; o_Clostridiales; f_Lachnospiraceae; g_; s_	88.24%	69.05%	90.32%	61.82%	67.74%	65.71%	-0.620	0.189	0.323
New.ReferenceOTU281	p_Firmicutes; c_Clostridia; o_Clostridiales; f_Lachnospiraceae; g_; s_	64.71%	69.05%	74.19%	74.55%	80.65%	65.71%	0.225	0.668	0.764
New.ReferenceOTU210	p_Firmicutes; c_Clostridia; o_Clostridiales; f_Lachnospiraceae; g_; s_	85.29%	85.71%	87.10%	70.91%	87.10%	65.71%	-0.640	0.171	0.301
FJ683372.1.1467	p_Firmicutes; c_Clostridia; o_Clostridiales; f_Lachnospiraceae; g_; s_	11.76%	30.95%	29.03%	52.73%	48.39%	68.57%	0.944	0.005	0.032
LC028646.1.1523	p_Firmicutes; c_Clostridia; o_Clostridiales; f_Lachnospiraceae; g_; s_	85.29%	61.90%	70.97%	56.36%	64.52%	68.57%	-0.414	0.414	0.557
New.ReferenceOTU303	p_Firmicutes; c_Clostridia; o_Clostridiales; f_Lachnospiraceae; g_; s_	17.65%	21.43%	25.81%	41.82%	51.61%	71.43%	0.984	0.000	0.011
HQ784683.1.1458	p_Firmicutes; c_Clostridia; o_Clostridiales; f_Lachnospiraceae; g_; s_	58.82%	50.00%	48.39%	56.36%	54.84%	71.43%	0.636	0.175	0.305
AB506430.1.1524	p_Firmicutes; c_Clostridia; o_Clostridiales; f_Lachnospiraceae; g_; s_	26.47%	33.33%	32.26%	45.45%	58.06%	71.43%	0.984	0.000	0.011
New.ReferenceOTU145	p_Firmicutes; c_Clostridia; o_Clostridiales; f_Lachnospiraceae; g_; s_	58.82%	45.24%	70.97%	70.91%	58.06%	71.43%	0.456	0.363	0.507
BABA01013952.1.1506	p_Firmicutes; c_Clostridia; o_Clostridiales; f_Lachnospiraceae; g_; s_	76.47%	61.90%	77.42%	67.27%	67.74%	71.43%	-0.162	0.758	0.834
New.CleanUp.ReferenceOTU25865	p_Firmicutes; c_Clostridia; o_Clostridiales; f_Lachnospiraceae; g_; s_	41.18%	45.24%	45.16%	58.18%	70.97%	71.43%	0.946	0.004	0.032
New.ReferenceOTU207	p_Firmicutes; c_Clostridia; o_Clostridiales; f_Lachnospiraceae; g_; s_	73.53%	57.14%	58.06%	67.27%	70.97%	74.29%	0.416	0.412	0.555
New.ReferenceOTU343	p_Firmicutes; c_Clostridia; o_Clostridiales; f_Lachnospiraceae; g_; s_	85.29%	69.05%	74.19%	70.91%	77.42%	74.29%	-0.280	0.591	0.704
EU773262.1.1395	p_Firmicutes; c_Clostridia; o_Clostridiales; f_Lachnospiraceae; g_; s_	76.47%	69.05%	77.42%	78.18%	77.42%	74.29%	0.119	0.822	0.873
FJ684605.1.1416	p_Firmicutes; c_Clostridia; o_Clostridiales; f_Lachnospiraceae; g_; s_	94.12%	83.33%	80.65%	81.82%	64.52%	77.14%	-0.737	0.095	0.196
New.ReferenceOTU134	p_Firmicutes; c_Clostridia; o_Clostridiales; f_Lachnospiraceae; g_; s_	70.59%	69.05%	74.19%	61.82%	70.97%	77.14%	0.374	0.466	0.605
LC028598.1.1524	p_Firmicutes; c_Clostridia; o_Clostridiales; f_Lachnospiraceae; g_; s_	85.29%	83.33%	80.65%	80.00%	77.42%	77.14%	-0.949	0.004	0.029
AGYU01000014.10038.11557	p_Firmicutes; c_Clostridia; o_Clostridiales; f_Lachnospiraceae; g_; s_	94.12%	83.33%	90.32%	83.64%	80.65%	80.00%	-0.815	0.048	0.126
FJ681558.1.1405	p_Firmicutes; c_Clostridia; o_Clostridiales; f_Lachnospiraceae; g_; s_	32.35%	35.71%	54.84%	54.55%	64.52%	82.86%	0.971	0.001	0.018
JX193319.1.1513	p_Firmicutes; c_Clostridia; o_Clostridiales; f_Lachnospiraceae; g_; s_	91.18%	83.33%	90.32%	80.00%	74.19%	82.86%	-0.615	0.194	0.328
EU774489.1.1384	p_Firmicutes; c_Clostridia; o_Clostridiales; f_Lachnospiraceae; g_; s_	91.18%	78.57%	96.77%	81.82%	77.42%	82.86%	-0.432	0.392	0.538
FPLO01004200.11.1520	p_Firmicutes; c_Clostridia; o_Clostridiales; f_Lachnospiraceae; g_; s_	20.59%	26.19%	22.58%	49.09%	58.06%	85.71%	0.961	0.002	0.023
LC028836.1.1521	p_Firmicutes; c_Clostridia; o_Clostridiales; f_Lachnospiraceae; g_; s_	94.12%	95.24%	96.77%	90.91%	90.32%	85.71%	-0.866	0.026	0.083
AF371632.1.1474	p_Firmicutes; c_Clostridia; o_Clostridiales; f_Lachnospiraceae; g_; s_	85.29%	69.05%	87.10%	78.18%	83.87%	88.57%	0.385	0.451	0.595
AB606272.1.1525	p_Firmicutes; c_Clostridia; o_Clostridiales; f_Lachnospiraceae; g_; s_	88.24%	80.95%	87.10%	87.27%	87.10%	88.57%	0.356	0.489	0.629
LC028789.1.1534	p_Firmicutes; c_Clostridia; o_Clostridiales; f_Lachnospiraceae; g_; s_	97.06%	95.24%	96.77%	96.36%	93.55%	88.57%	-0.884	0.019	0.073
CZAC01000092.963.2475	p_Firmicutes; c_Clostridia; o_Clostridiales; f_Lachnospiraceae; g_; s_	94.12%	80.95%	93.55%	87.27%	96.77%	91.43%	0.230	0.661	0.760
FLKC01000014.123770.125280	p_Firmicutes; c_Clostridia; o_Clostridiales; f_Lachnospiraceae; g_; s_	58.82%	61.90%	67.74%	74.55%	87.10%	94.29%	0.988	0.000	0.010
New.ReferenceOTU138	p_Firmicutes; c_Clostridia; o_Clostridiales; f_Lachnospiraceae; g_; s_	91.18%	90.48%	96.77%	87.27%	93.55%	94.29%	0.285	0.584	0.700
AB034123.1.1528	p_Firmicutes; c_Clostridia; o_Clostridiales; f_Lachnospiraceae; g_; s_	97.06%	97.62%	100.00%	96.36%	96.77%	94.29%	-0.640	0.171	0.301
LC028630.1.1516	p_Firmicutes; c_Clostridia; o_Clostridiales; f_Lachnospiraceae; g_; s_	97.06%	97.62%	100.00%	98.18%	100.00%	94.29%	-0.335	0.516	0.649
AB746681.1.1519	p_Firmicutes; c_Clostridia; o_Clostridiales; f_Lachnospiraceae; g_; s_	94.12%	90.48%	87.10%	89.09%	90.32%	100.00%	0.484	0.330	0.473
AY239462.1.1500	p_Firmicutes; c_Clostridia; o_Clostridiales; f_Lachnospiraceae; g_; s_	91.18%	90.48%	93.55%	90.91%	90.32%	100.00%	0.669	0.146	0.271
BABG01001469.752.2264	p_Firmicutes; c_Clostridia; o_Clostridiales; f_Lachnospiraceae; g_; s_	82.35%	88.10%	90.32%	100.00%	93.55%	100.00%	0.834	0.039	0.108
JX096119.1.1400	p_Firmicutes; c_Clostridia; o_Clostridiales; f_Lachnospiraceae; g_; s_	97.06%	90.48%	93.55%	92.73%	100.00%	100.00%	0.610	0.199	0.334
LC028722.1.1511	p_Firmicutes; c_Clostridia; o_Clostridiales; f_Lachnospiraceae; g_; s_	97.06%	92.86%	87.10%	96.36%	100.00%	100.00%	0.530	0.280	0.419
AATC01000018.2.1513	p_Firmicutes; c_Clostridia; o_Clostridiales; f_Lachnospiraceae; g_; s_	97.06%	95.24%	96.77%	100.00%	100.00%	100.00%	0.770	0.073	0.165
AB494794.1.1538	p_Firmicutes; c_Clostridia; o_Clostridiales; f_Lachnospiraceae; g_; s_	100.00%	100.00%	100.00%	100.00%	100.00%	100.00%	NA	NA	NA
AAVP02000025.9720.11231	p_Firmicutes; c_Clostridia; o_Clostridiales; f_Lachnospiraceae; g_[Ruminococcus]	26.47%	33.33%	48.39%	52.73%	64.52%	80.00%	0.989	0.000	0.009
BBDW01000030.73.1604	p_Firmicutes; c_Clostridia; o_Clostridiales; f_Lachnospiraceae; g_[Ruminococcus]; s_	17.65%	23.81%	22.58%	38.18%	45.16%	51.43%	0.965	0.002	0.021
New.ReferenceOTU301	p_Firmicutes; c_Clostridia; o_Clostridiales; f_Lachnospiraceae; g_[Ruminococcus]; s_gnavus	44.12%	35.71%	51.61%	45.45%	41.94%	45.71%	0.149	0.778	0.845
FPLM01007606.1.1499	p_Firmicutes; c_Clostridia; o_Clostridiales; f_Lachnospiraceae; g_Anaerostipes; s_	41.18%	52.38%	58.06%	34.55%	32.26%	37.14%	-0.511	0.301	0.443
JX096298.1.1400	p_Firmicutes; c_Clostridia; o_Clostridiales; f_Lachnospiraceae; g_Blautia; s_	52.94%	33.33%	61.29%	45.45%	25.81%	17.14%	-0.765	0.077	0.170
FJ880389.1.1491	p_Firmicutes; c_Clostridia; o_Clostridiales; f_Lachnospiraceae; g_Blautia; s_	41.18%	57.14%	64.52%	72.73%	80.65%	94.29%	0.986	0.000	0.011
JN559613.1.1501	p_Firmicutes; c_Clostridia; o_Clostridiales; f_Lachnospiraceae; g_Blautia; s_producta	55.88%	80.95%	74.19%	89.09%	80.65%	97.14%	0.832	0.040	0.109
EU843609.1.1521	p_Firmicutes; c_Clostridia; o_Clostridiales; f_Lachnospiraceae; g_Butyrivibrio; s_	67.65%	80.95%	70.97%	80.00%	70.97%	80.00%	0.396	0.437	0.582
AB507542.1.1526	p_Firmicutes; c_Clostridia; o_Clostridiales; f_Lachnospiraceae; g_Butyrivibrio; s_	94.12%	97.62%	93.55%	96.36%	96.77%	97.14%	0.530	0.279	0.419
GU302762.1.1499	p_Firmicutes; c_Clostridia; o_Clostridiales; f_Lachnospiraceae; g_Butyrivibrio; s_	85.29%	95.24%	90.32%	89.09%	93.55%	100.00%	0.778	0.068	0.159
AB185562.1.1541	p_Firmicutes; c_Clostridia; o_Clostridiales; f_Lachnospiraceae; g_Butyrivibrio; s_	91.18%	95.24%	90.32%	100.00%	100.00%	100.00%	0.777	0.069	0.159
EU777186.1.1321	p_Firmicutes; c_Clostridia; o_Clostridiales; f_Lachnospiraceae; g_Coprococcus; s_	26.47%	26.19%	22.58%	29.09%	29.03%	51.43%	0.806	0.053	0.132
EU844298.1.1506	p_Firmicutes; c_Clostridia; o_Clostridiales; f_Lachnospiraceae; g_Coprococcus; s_	61.76%	50.00%	58.06%	74.55%	67.74%	65.71%	0.475	0.342	0.484
EU466237.1.1383	p_Firmicutes; c_Clostridia; o_Clostridiales; f_Lachnospiraceae; g_Coprococcus; s_	38.24%	42.86%	54.84%	58.18%	70.97%	68.57%	0.928	0.008	0.042
MJIG01000003.216170.217710	p_Firmicutes; c_Clostridia; o_Clostridiales; f_Lachnospiraceae; g_Coprococcus; s_	26.47%	40.48%	45.16%	52.73%	58.06%	82.86%	0.984	0.000	0.011
DQ115988.1.1485	p_Firmicutes; c_Clostridia; o_Clostridiales; f_Lachnospiraceae; g_Coprococcus; s_	82.35%	76.19%	100.00%	74.55%	87.10%	85.71%	0.130	0.807	0.867
DQ105656.1.1515	p_Firmicutes; c_Clostridia; o_Clostridiales; f_Lachnospiraceae; g_Coprococcus; s_	100.00%	97.62%	96.77%	98.18%	100.00%	100.00%	0.380	0.458	0.599
New.ReferenceOTU262	p_Firmicutes; c_Clostridia; o_Clostridiales; f_Lachnospiraceae; g_Dorea; s_	44.12%	42.86%	54.84%	41.82%	29.03%	14.29%	-0.843	0.035	0.100
LC028755.1.1515	p_Firmicutes; c_Clostridia; o_Clostridiales; f_Lachnospiraceae; g_Dorea; s_	55.88%	47.62%	45.16%	47.27%	41.94%	22.86%	-0.927	0.008	0.042
JX096354.1.1408	p_Firmicutes; c_Clostridia; o_Clostridiales; f_Lachnospiraceae; g_Dorea; s_	52.94%	64.29%	41.94%	36.36%	35.48%	31.43%	-0.794	0.059	0.142
EU778759.1.1343	p_Firmicutes; c_Clostridia; o_Clostridiales; f_Lachnospiraceae; g_Dorea; s_	67.65%	59.52%	64.52%	43.64%	54.84%	48.57%	-0.706	0.117	0.228
New.ReferenceOTU159	p_Firmicutes; c_Clostridia; o_Clostridiales; f_Lachnospiraceae; g_Dorea; s_	67.65%	40.48%	61.29%	56.36%	61.29%	54.29%	-0.104	0.845	0.885
LC028833.1.1523	p_Firmicutes; c_Clostridia; o_Clostridiales; f_Lachnospiraceae; g_Dorea; s_	55.88%	61.90%	41.94%	47.27%	38.71%	60.00%	-0.107	0.840	0.883
JX096367.1.1408	p_Firmicutes; c_Clostridia; o_Clostridiales; f_Lachnospiraceae; g_Dorea; s_	73.53%	64.29%	67.74%	49.09%	51.61%	62.86%	-0.521	0.290	0.430
HQ716221.1.1478	p_Firmicutes; c_Clostridia; o_Clostridiales; f_Lachnospiraceae; g_Dorea; s_	79.41%	66.67%	77.42%	74.55%	70.97%	62.86%	-0.698	0.123	0.239
AAXA02000014.354465.355983	p_Firmicutes; c_Clostridia; o_Clostridiales; f_Lachnospiraceae; g_Dorea; s_	44.12%	54.76%	45.16%	60.00%	48.39%	68.57%	0.705	0.118	0.229
LC028575.1.1524	p_Firmicutes; c_Clostridia; o_Clostridiales; f_Lachnospiraceae; g_Dorea; s_	82.35%	73.81%	87.10%	76.36%	93.55%	71.43%	-0.118	0.824	0.873
LC028629.1.1515	p_Firmicutes; c_Clostridia; o_Clostridiales; f_Lachnospiraceae; g_Dorea; s_	94.12%	92.86%	96.77%	85.45%	87.10%	82.86%	-0.827	0.042	0.114
FLKG01000008.748.2260	p_Firmicutes; c_Clostridia; o_Clostridiales; f_Lachnospiraceae; g_Dorea; s_	82.35%	71.43%	58.06%	83.64%	77.42%	85.71%	0.346	0.502	0.637
LC028878.1.1515	p_Firmicutes; c_Clostridia; o_Clostridiales; f_Lachnospiraceae; g_Dorea; s_	94.12%	83.33%	90.32%	85.45%	77.42%	88.57%	-0.413	0.416	0.558
LC028572.1.1524	p_Firmicutes; c_Clostridia; o_Clostridiales; f_Lachnospiraceae; g_Dorea; s_	97.06%	97.62%	100.00%	98.18%	100.00%	91.43%	-0.503	0.309	0.452
FPLO01002600.8.1520	p_Firmicutes; c_Clostridia; o_Clostridiales; f_Lachnospiraceae; g_Roseburia; s_	29.41%	28.57%	41.94%	52.73%	64.52%	65.71%	0.939	0.005	0.035
FPLO01004147.8.1521	p_Firmicutes; c_Clostridia; o_Clostridiales; f_Lachnospiraceae; g_Roseburia; s_	88.24%	76.19%	83.87%	74.55%	93.55%	77.14%	-0.107	0.840	0.883
New.ReferenceOTU148	p_Firmicutes; c_Clostridia; o_Clostridiales; f_Peptococcaceae; g_rc4-4; s_	14.71%	16.67%	32.26%	29.09%	48.39%	60.00%	0.970	0.001	0.018
EU622770.1.1565	p_Firmicutes; c_Clostridia; o_Clostridiales; f_Peptococcaceae; g_rc4-4; s_	91.18%	92.86%	93.55%	94.55%	100.00%	100.00%	0.954	0.003	0.027
GQ468579.1.1488	p_Firmicutes; c_Clostridia; o_Clostridiales; f_Peptostreptococcaceae; g_; s_	79.41%	85.71%	67.74%	72.73%	74.19%	91.43%	0.329	0.524	0.657
JFOF01000052.9721.11268	p_Firmicutes; c_Clostridia; o_Clostridiales; f_Peptostreptococcaceae; g_; s_	88.24%	85.71%	93.55%	81.82%	90.32%	91.43%	0.278	0.593	0.704
FMGS01000005.587.2069	p_Firmicutes; c_Clostridia; o_Clostridiales; f_Peptostreptococcaceae; g_; s_	94.12%	97.62%	93.55%	94.55%	93.55%	91.43%	-0.662	0.152	0.280
JMMB01000008.9370.10857	p_Firmicutes; c_Clostridia; o_Clostridiales; f_Peptostreptococcaceae; g_; s_	100.00%	92.86%	93.55%	89.09%	96.77%	91.43%	-0.446	0.375	0.518
LC028825.1.1520	p_Firmicutes; c_Clostridia; o_Clostridiales; f_Ruminococcaceae; g_; s_	55.88%	40.48%	45.16%	16.36%	9.68%	8.57%	-0.912	0.011	0.052
FJ951904.1.1471	p_Firmicutes; c_Clostridia; o_Clostridiales; f_Ruminococcaceae; g_; s_	50.00%	28.57%	25.81%	36.36%	16.13%	8.57%	-0.885	0.019	0.072
New.ReferenceOTU41	p_Firmicutes; c_Clostridia; o_Clostridiales; f_Ruminococcaceae; g_; s_	52.94%	30.95%	45.16%	32.73%	32.26%	8.57%	-0.872	0.024	0.078
AB185817.1.1510	p_Firmicutes; c_Clostridia; o_Clostridiales; f_Ruminococcaceae; g_; s_	52.94%	23.81%	25.81%	25.45%	12.90%	11.43%	-0.854	0.030	0.090
New.ReferenceOTU26	p_Firmicutes; c_Clostridia; o_Clostridiales; f_Ruminococcaceae; g_; s_	55.88%	40.48%	41.94%	21.82%	25.81%	11.43%	-0.935	0.006	0.036
EU461120.1.1370	p_Firmicutes; c_Clostridia; o_Clostridiales; f_Ruminococcaceae; g_; s_	70.59%	42.86%	45.16%	34.55%	29.03%	11.43%	-0.954	0.003	0.026
New.ReferenceOTU205	p_Firmicutes; c_Clostridia; o_Clostridiales; f_Ruminococcaceae; g_; s_	50.00%	42.86%	41.94%	20.00%	32.26%	11.43%	-0.879	0.021	0.077
FJ848435.1.1474	p_Firmicutes; c_Clostridia; o_Clostridiales; f_Ruminococcaceae; g_; s_	52.94%	30.95%	41.94%	18.18%	19.35%	14.29%	-0.859	0.028	0.087
New.ReferenceOTU147	p_Firmicutes; c_Clostridia; o_Clostridiales; f_Ruminococcaceae; g_; s_	55.88%	33.33%	35.48%	18.18%	22.58%	14.29%	-0.874	0.023	0.078
KR514379.1.1435	p_Firmicutes; c_Clostridia; o_Clostridiales; f_Ruminococcaceae; g_; s_	50.00%	47.62%	35.48%	30.91%	22.58%	14.29%	-0.978	0.001	0.016
HQ716104.1.1471	p_Firmicutes; c_Clostridia; o_Clostridiales; f_Ruminococcaceae; g_; s_	55.88%	38.10%	51.61%	29.09%	41.94%	14.29%	-0.804	0.054	0.132
LC028564.1.1523	p_Firmicutes; c_Clostridia; o_Clostridiales; f_Ruminococcaceae; g_; s_	67.65%	42.86%	38.71%	29.09%	29.03%	17.14%	-0.916	0.010	0.050
EU777225.1.1256	p_Firmicutes; c_Clostridia; o_Clostridiales; f_Ruminococcaceae; g_; s_	70.59%	45.24%	51.61%	25.45%	32.26%	17.14%	-0.895	0.016	0.063
EU464176.1.1371	p_Firmicutes; c_Clostridia; o_Clostridiales; f_Ruminococcaceae; g_; s_	55.88%	42.86%	48.39%	41.82%	41.94%	17.14%	-0.896	0.016	0.063

EU474471.1.1402	p_Firmicutes; c_Clostridia; o_Clostridiales; f_Ruminococcaceae; g_; s_	47.06%	40.48%	51.61%	40.00%	32.26%	20.00%	-0.881	0.020	0.076
EU466447.1.1388	p_Firmicutes; c_Clostridia; o_Clostridiales; f_Ruminococcaceae; g_; s_	61.76%	35.71%	41.94%	38.18%	41.94%	20.00%	-0.808	0.052	0.131
LC028740.1.1495	p_Firmicutes; c_Clostridia; o_Clostridiales; f_Ruminococcaceae; g_; s_	79.41%	57.14%	54.84%	49.09%	41.94%	20.00%	-0.974	0.001	0.018
FJ881007.1.1472	p_Firmicutes; c_Clostridia; o_Clostridiales; f_Ruminococcaceae; g_; s_	52.94%	33.33%	41.94%	30.91%	54.84%	20.00%	-0.486	0.329	0.473
EU794180.1.1436	p_Firmicutes; c_Clostridia; o_Clostridiales; f_Ruminococcaceae; g_; s_	50.00%	54.76%	41.94%	34.55%	16.13%	22.86%	-0.878	0.021	0.077
JX095338.1.1399	p_Firmicutes; c_Clostridia; o_Clostridiales; f_Ruminococcaceae; g_; s_	52.94%	50.00%	38.71%	40.00%	35.48%	22.86%	-0.961	0.002	0.023
EU778682.1.1371	p_Firmicutes; c_Clostridia; o_Clostridiales; f_Ruminococcaceae; g_; s_	73.53%	50.00%	58.06%	36.36%	45.16%	22.86%	-0.890	0.018	0.068
JX096366.1.1401	p_Firmicutes; c_Clostridia; o_Clostridiales; f_Ruminococcaceae; g_; s_	50.00%	52.38%	51.61%	36.36%	32.26%	25.71%	-0.920	0.009	0.046
EU460956.1.1373	p_Firmicutes; c_Clostridia; o_Clostridiales; f_Ruminococcaceae; g_; s_	55.88%	38.10%	41.94%	38.18%	35.48%	25.71%	-0.903	0.014	0.057
LC028617.1.1502	p_Firmicutes; c_Clostridia; o_Clostridiales; f_Ruminococcaceae; g_; s_	61.76%	40.48%	35.48%	45.45%	38.71%	25.71%	-0.807	0.052	0.132
AB874529.1.1431	p_Firmicutes; c_Clostridia; o_Clostridiales; f_Ruminococcaceae; g_; s_	50.00%	40.48%	54.84%	47.27%	38.71%	25.71%	-0.791	0.061	0.144
LC028658.1.1502	p_Firmicutes; c_Clostridia; o_Clostridiales; f_Ruminococcaceae; g_; s_	50.00%	42.86%	48.39%	41.82%	41.94%	25.71%	-0.881	0.020	0.076
EU465772.1.1371	p_Firmicutes; c_Clostridia; o_Clostridiales; f_Ruminococcaceae; g_; s_	52.94%	45.24%	38.71%	43.64%	41.94%	25.71%	-0.878	0.021	0.077
GQ448446.1.1382	p_Firmicutes; c_Clostridia; o_Clostridiales; f_Ruminococcaceae; g_; s_	50.00%	38.10%	38.71%	36.36%	48.39%	25.71%	-0.606	0.203	0.339
EU794122.1.1443	p_Firmicutes; c_Clostridia; o_Clostridiales; f_Ruminococcaceae; g_; s_	70.59%	50.00%	54.84%	43.64%	48.39%	25.71%	-0.902	0.014	0.058
New.ReferenceOTU40	p_Firmicutes; c_Clostridia; o_Clostridiales; f_Ruminococcaceae; g_; s_	52.94%	42.86%	61.29%	47.27%	48.39%	25.71%	-0.696	0.124	0.241
New.ReferenceOTU95	p_Firmicutes; c_Clostridia; o_Clostridiales; f_Ruminococcaceae; g_; s_	76.47%	47.62%	54.84%	47.27%	54.84%	25.71%	-0.818	0.047	0.123
JX095157.1.1377	p_Firmicutes; c_Clostridia; o_Clostridiales; f_Ruminococcaceae; g_; s_	55.88%	35.71%	41.94%	34.55%	38.71%	28.57%	-0.779	0.068	0.158
New.ReferenceOTU81	p_Firmicutes; c_Clostridia; o_Clostridiales; f_Ruminococcaceae; g_; s_	70.59%	47.62%	48.39%	40.00%	41.94%	28.57%	-0.897	0.015	0.062
JQ084465.1.1489	p_Firmicutes; c_Clostridia; o_Clostridiales; f_Ruminococcaceae; g_; s_	70.59%	40.48%	48.39%	45.45%	41.94%	28.57%	-0.828	0.042	0.113
JX096094.1.1374	p_Firmicutes; c_Clostridia; o_Clostridiales; f_Ruminococcaceae; g_; s_	50.00%	40.48%	38.71%	41.82%	48.39%	28.57%	-0.619	0.190	0.323
FJ951892.1.1480	p_Firmicutes; c_Clostridia; o_Clostridiales; f_Ruminococcaceae; g_; s_	67.65%	30.95%	58.06%	45.45%	48.39%	28.57%	-0.611	0.198	0.333
New.CleanUp.ReferenceOTU4661	p_Firmicutes; c_Clostridia; o_Clostridiales; f_Ruminococcaceae; g_; s_	55.88%	42.86%	51.61%	38.18%	51.61%	28.57%	-0.688	0.131	0.250
New.ReferenceOTU46	p_Firmicutes; c_Clostridia; o_Clostridiales; f_Ruminococcaceae; g_; s_	64.71%	47.62%	61.29%	41.82%	51.61%	28.57%	-0.799	0.056	0.137
EU794231.1.1426	p_Firmicutes; c_Clostridia; o_Clostridiales; f_Ruminococcaceae; g_; s_	70.59%	52.38%	54.84%	47.27%	67.74%	28.57%	-0.649	0.163	0.293
EU777305.1.1341	p_Firmicutes; c_Clostridia; o_Clostridiales; f_Ruminococcaceae; g_; s_	52.94%	45.24%	51.61%	27.27%	29.03%	31.43%	-0.775	0.070	0.162
EU772004.1.1368	p_Firmicutes; c_Clostridia; o_Clostridiales; f_Ruminococcaceae; g_; s_	50.00%	33.33%	51.61%	27.27%	38.71%	31.43%	-0.531	0.278	0.418
EU465792.1.1395	p_Firmicutes; c_Clostridia; o_Clostridiales; f_Ruminococcaceae; g_; s_	58.82%	47.62%	61.29%	30.91%	41.94%	31.43%	-0.741	0.092	0.191
KC162990.1.1523	p_Firmicutes; c_Clostridia; o_Clostridiales; f_Ruminococcaceae; g_; s_	52.94%	52.38%	35.48%	32.73%	41.94%	31.43%	-0.724	0.103	0.207
New.CleanUp.ReferenceOTU24585	p_Firmicutes; c_Clostridia; o_Clostridiales; f_Ruminococcaceae; g_; s_	52.94%	42.86%	41.94%	34.55%	41.94%	31.43%	-0.820	0.046	0.121
KR068079.1.1489	p_Firmicutes; c_Clostridia; o_Clostridiales; f_Ruminococcaceae; g_; s_	61.76%	38.10%	51.61%	41.82%	45.16%	31.43%	-0.741	0.092	0.191
HM124210.1.1491	p_Firmicutes; c_Clostridia; o_Clostridiales; f_Ruminococcaceae; g_; s_	61.76%	54.76%	64.52%	49.09%	45.16%	31.43%	-0.910	0.012	0.053
JX095159.1.1382	p_Firmicutes; c_Clostridia; o_Clostridiales; f_Ruminococcaceae; g_; s_	44.12%	45.24%	29.03%	30.91%	51.61%	31.43%	-0.210	0.690	0.782
JX095618.1.1403	p_Firmicutes; c_Clostridia; o_Clostridiales; f_Ruminococcaceae; g_; s_	44.12%	42.86%	41.94%	49.09%	51.61%	31.43%	-0.351	0.495	0.634
EU794230.1.1432	p_Firmicutes; c_Clostridia; o_Clostridiales; f_Ruminococcaceae; g_; s_	73.53%	57.14%	58.06%	49.09%	51.61%	31.43%	-0.937	0.006	0.036
JX218683.1.1490	p_Firmicutes; c_Clostridia; o_Clostridiales; f_Ruminococcaceae; g_; s_	29.41%	33.33%	41.94%	40.00%	54.84%	31.43%	0.306	0.555	0.681
KR514492.1.1433	p_Firmicutes; c_Clostridia; o_Clostridiales; f_Ruminococcaceae; g_; s_	70.59%	54.76%	61.29%	47.27%	58.06%	31.43%	-0.832	0.040	0.109
EU794147.1.1449	p_Firmicutes; c_Clostridia; o_Clostridiales; f_Ruminococcaceae; g_; s_	35.29%	45.24%	41.94%	49.09%	35.48%	34.29%	-0.326	0.528	0.659
LC028614.1.1499	p_Firmicutes; c_Clostridia; o_Clostridiales; f_Ruminococcaceae; g_; s_	55.88%	42.86%	64.52%	45.45%	45.16%	34.29%	-0.669	0.146	0.271
EU463264.1.1365	p_Firmicutes; c_Clostridia; o_Clostridiales; f_Ruminococcaceae; g_; s_	76.47%	71.43%	74.19%	49.09%	58.06%	34.29%	-0.897	0.015	0.062
CDYI01023850.213.1721	p_Firmicutes; c_Clostridia; o_Clostridiales; f_Ruminococcaceae; g_; s_	64.71%	35.71%	48.39%	52.73%	58.06%	34.29%	-0.435	0.389	0.535
EU794292.1.1444	p_Firmicutes; c_Clostridia; o_Clostridiales; f_Ruminococcaceae; g_; s_	82.35%	73.81%	70.97%	61.82%	58.06%	34.29%	-0.978	0.001	0.016
LC028667.1.1507	p_Firmicutes; c_Clostridia; o_Clostridiales; f_Ruminococcaceae; g_; s_	23.53%	35.71%	51.61%	27.27%	32.26%	37.14%	0.180	0.734	0.814
EU772986.1.1288	p_Firmicutes; c_Clostridia; o_Clostridiales; f_Ruminococcaceae; g_; s_	50.00%	45.24%	45.16%	45.45%	35.48%	37.14%	-0.906	0.013	0.055
New.CleanUp.ReferenceOTU39673	p_Firmicutes; c_Clostridia; o_Clostridiales; f_Ruminococcaceae; g_; s_	50.00%	40.48%	58.06%	54.55%	38.71%	37.14%	-0.542	0.266	0.406
AY916205.1.1351	p_Firmicutes; c_Clostridia; o_Clostridiales; f_Ruminococcaceae; g_; s_	50.00%	47.62%	58.06%	36.36%	45.16%	37.14%	-0.611	0.198	0.333
New.ReferenceOTU121	p_Firmicutes; c_Clostridia; o_Clostridiales; f_Ruminococcaceae; g_; s_	79.41%	47.62%	54.84%	54.55%	48.39%	37.14%	-0.812	0.050	0.127
LC028609.1.1518	p_Firmicutes; c_Clostridia; o_Clostridiales; f_Ruminococcaceae; g_; s_	70.59%	61.90%	58.06%	50.91%	51.61%	37.14%	-0.970	0.001	0.018
New.CleanUp.ReferenceOTU16711	p_Firmicutes; c_Clostridia; o_Clostridiales; f_Ruminococcaceae; g_; s_	52.94%	21.43%	45.16%	36.36%	25.81%	40.00%	-0.266	0.611	0.718
LC028666.1.1498	p_Firmicutes; c_Clostridia; o_Clostridiales; f_Ruminococcaceae; g_; s_	70.59%	47.62%	54.84%	36.36%	35.48%	40.00%	-0.770	0.073	0.166
New.CleanUp.ReferenceOTU16541	p_Firmicutes; c_Clostridia; o_Clostridiales; f_Ruminococcaceae; g_; s_	17.65%	28.57%	51.61%	32.73%	38.71%	40.00%	0.523	0.287	0.426
LC028796.1.1523	p_Firmicutes; c_Clostridia; o_Clostridiales; f_Ruminococcaceae; g_; s_	50.00%	45.24%	29.03%	38.18%	41.94%	40.00%	-0.301	0.562	0.685
LC028558.1.1520	p_Firmicutes; c_Clostridia; o_Clostridiales; f_Ruminococcaceae; g_; s_	55.88%	54.76%	48.39%	38.18%	45.16%	40.00%	-0.793	0.060	0.143
LC028803.1.1521	p_Firmicutes; c_Clostridia; o_Clostridiales; f_Ruminococcaceae; g_; s_	64.71%	45.24%	51.61%	38.18%	48.39%	40.00%	-0.680	0.137	0.258
EU465539.1.1372	p_Firmicutes; c_Clostridia; o_Clostridiales; f_Ruminococcaceae; g_; s_	79.41%	59.52%	58.06%	50.91%	48.39%	40.00%	-0.928	0.008	0.042
New.ReferenceOTU93	p_Firmicutes; c_Clostridia; o_Clostridiales; f_Ruminococcaceae; g_; s_	55.88%	38.10%	51.61%	29.09%	58.06%	40.00%	-0.180	0.733	0.814
LC028787.1.1526	p_Firmicutes; c_Clostridia; o_Clostridiales; f_Ruminococcaceae; g_; s_	76.47%	71.43%	67.74%	54.55%	61.29%	40.00%	-0.925	0.008	0.043
JX096196.1.1388	p_Firmicutes; c_Clostridia; o_Clostridiales; f_Ruminococcaceae; g_; s_	88.24%	59.52%	70.97%	47.27%	64.52%	40.00%	-0.776	0.070	0.160
EU773203.1.1395	p_Firmicutes; c_Clostridia; o_Clostridiales; f_Ruminococcaceae; g_; s_	61.76%	54.76%	61.29%	52.73%	64.52%	40.00%	-0.600	0.208	0.344
FJ848384.1.1503	p_Firmicutes; c_Clostridia; o_Clostridiales; f_Ruminococcaceae; g_; s_	50.00%	33.33%	48.39%	40.00%	29.03%	42.86%	-0.349	0.497	0.634
EU461007.1.1373	p_Firmicutes; c_Clostridia; o_Clostridiales; f_Ruminococcaceae; g_; s_	52.94%	59.52%	74.19%	47.27%	38.71%	42.86%	-0.590	0.218	0.356
AB185613.1.1514	p_Firmicutes; c_Clostridia; o_Clostridiales; f_Ruminococcaceae; g_; s_	55.88%	57.14%	35.48%	45.45%	41.94%	42.86%	-0.561	0.247	0.385
New.CleanUp.ReferenceOTU44031	p_Firmicutes; c_Clostridia; o_Clostridiales; f_Ruminococcaceae; g_; s_	44.12%	57.14%	51.61%	41.82%	48.39%	42.86%	-0.354	0.491	0.631
LC028571.1.1523	p_Firmicutes; c_Clostridia; o_Clostridiales; f_Ruminococcaceae; g_; s_	67.65%	57.14%	61.29%	58.18%	51.61%	42.86%	-0.952	0.003	0.027
New.ReferenceOTU139	p_Firmicutes; c_Clostridia; o_Clostridiales; f_Ruminococcaceae; g_; s_	41.18%	35.71%	51.61%	41.82%	58.06%	42.86%	0.357	0.487	0.629
New.CleanUp.ReferenceOTU17903	p_Firmicutes; c_Clostridia; o_Clostridiales; f_Ruminococcaceae; g_; s_	41.18%	47.62%	58.06%	47.27%	35.48%	45.71%	-0.195	0.711	0.800
New.ReferenceOTU50	p_Firmicutes; c_Clostridia; o_Clostridiales; f_Ruminococcaceae; g_; s_	67.65%	50.00%	58.06%	54.55%	35.48%	45.71%	-0.757	0.081	0.175
New.ReferenceOTU56	p_Firmicutes; c_Clostridia; o_Clostridiales; f_Ruminococcaceae; g_; s_	55.88%	45.24%	45.16%	40.00%	41.94%	45.71%	-0.548	0.261	0.401
GQ448447.1.1390	p_Firmicutes; c_Clostridia; o_Clostridiales; f_Ruminococcaceae; g_; s_	50.00%	28.57%	48.39%	32.73%	48.39%	45.71%	0.144	0.785	0.849
EU772225.1.1386	p_Firmicutes; c_Clostridia; o_Clostridiales; f_Ruminococcaceae; g_; s_	67.65%	64.29%	61.29%	56.36%	48.39%	45.71%	-0.983	0.000	0.011
New.ReferenceOTU127	p_Firmicutes; c_Clostridia; o_Clostridiales; f_Ruminococcaceae; g_; s_	70.59%	59.52%	70.97%	49.09%	54.84%	45.71%	-0.804	0.054	0.132
EU794275.1.1454	p_Firmicutes; c_Clostridia; o_Clostridiales; f_Ruminococcaceae; g_; s_	70.59%	66.67%	61.29%	58.18%	54.84%	45.71%	-0.991	0.000	0.008
EU472358.1.1395	p_Firmicutes; c_Clostridia; o_Clostridiales; f_Ruminococcaceae; g_; s_	73.53%	52.38%	61.29%	43.64%	58.06%	45.71%	-0.653	0.159	0.290
LC028828.1.1506	p_Firmicutes; c_Clostridia; o_Clostridiales; f_Ruminococcaceae; g_; s_	70.59%	54.76%	61.29%	49.09%	58.06%	45.71%	-0.759	0.080	0.174
FJ951856.1.1489	p_Firmicutes; c_Clostridia; o_Clostridiales; f_Ruminococcaceae; g_; s_	76.47%	61.90%	74.19%	52.73%	61.29%	45.71%	-0.810	0.051	0.129
New.CleanUp.ReferenceOTU8049	p_Firmicutes; c_Clostridia; o_Clostridiales; f_Ruminococcaceae; g_; s_	67.65%	50.00%	48.39%	50.91%	64.52%	45.71%	-0.384	0.453	0.595
EU794165.1.1436	p_Firmicutes; c_Clostridia; o_Clostridiales; f_Ruminococcaceae; g_; s_	50.00%	52.38%	61.29%	47.27%	41.94%	48.57%	-0.427	0.398	0.542
AB746687.1.1524	p_Firmicutes; c_Clostridia; o_Clostridiales; f_Ruminococcaceae; g_; s_	52.94%	35.71%	35.48%	40.00%	48.39%	48.57%	0.203	0.700	0.791
DQ800164.1.1390	p_Firmicutes; c_Clostridia; o_Clostridiales; f_Ruminococcaceae; g_; s_	50.00%	26.19%	35.48%	45.45%	48.39%	48.57%	0.375	0.464	0.604
LC028779.1.1510	p_Firmicutes; c_Clostridia; o_Clostridiales; f_Ruminococcaceae; g_; s_	88.24%	78.57%	77.42%	72.73%	48.39%	48.57%	-0.952	0.003	0.027
EU464147.1.1366	p_Firmicutes; c_Clostridia; o_Clostridiales; f_Ruminococcaceae; g_; s_	52.94%	47.62%	48.39%	49.09%	51.61%	48.57%	-0.244	0.641	0.747
EU794079.1.1431	p_Firmicutes; c_Clostridia; o_Clostridiales; f_Ruminococcaceae; g_; s_	73.53%	61.90%	61.29%	52.73%	51.61%	48.57%	-0.920	0.009	0.046
EU468981.1.1335	p_Firmicutes; c_Clostridia; o_Clostridiales; f_Ruminococcaceae; g_; s_	82.35%	54.76%	67.74%	61.82%	54.84%	48.57%	-0.799	0.057	0.137
New.ReferenceOTU315	p_Firmicutes; c_Clostridia; o_Clostridiales; f_Ruminococcaceae; g_; s_	73.53%	52.38%	64.52%	38.18%	58.06%	48.57%	-0.534	0.276	0.416
LC028785.1.1511	p_Firmicutes; c_Clostridia; o_Clostridiales; f_Ruminococcaceae; g_; s_	82.35%	59.52%	58.06%	43.64%	58.06%	48.57%	-0.695	0.125	0.242
New.CleanUp.ReferenceOTU42678	p_Firmicutes; c_Clostridia; o_Clostridiales; f_Ruminococcaceae; g_; s_	67.65%	50.00%	54.84%	47.27%	61.29%	48.57%	-0.446	0.375	0.518
LC028754.1.1521	p_Firmicutes; c_Clostridia; o_Clostridiales; f_Ruminococcaceae; g_; s_	73.53%	52.38%	74.19%	45.45%	67.74%	48.57%	-0.454	0.366	0.510
LC028725.1.1497	p_Firmicutes; c_Clostridia; o_Clostridiales; f_Ruminococcaceae; g_; s_	82.35%	73.81%	70.97%	60.00%	74.19%	48.57%	-0.805	0.053	0.132
EF404722.1.1476	p_Firmicutes; c_Clostridia; o_Clostridiales; f_Ruminococcaceae; g_; s_	5.88%	19.05%	6.45%	18.18%	19.35%	51.43%	0.855	0.030	0.090
EU259405.1.1499	p_Firmicutes; c_Clostridia; o_Clostridiales; f_Ruminococcaceae; g_; s_	70.59%	54.76%	58.06%	52.73%	58.06%	51.43%	-0.687	0.131	0.250
GQ451266.1.1497	p_Firmicutes; c_Clostridia; o_Clostridiales; f_Ruminococcaceae; g_; s_	64.71%	57.14%	70.97%	54.55%	58.06%	51.43%	-0.634	0.177	0.307
LC028851.1.1522	p_Firmicutes; c_Clostridia; o_Clostridiales; f_Ruminococcaceae; g_; s_	73.53%	64.29%	58.06%	56.36%	58.06%	51.43%	-0.877	0.022	0.077
LC028585.1.1498	p_Firmicutes; c_Clostridia; o_Clostridiales; f_Ruminococcaceae; g_; s_	67.65%	69.05%	74.19%	56.36%	58.06%	51.43%	-0.808	0.052	0.131
EU460994.1.1388	p_Firmicutes; c_Clostridia; o_Clostridiales; f_Ruminococcaceae; g_; s_	70.59%	61.90%	51.61%	52.73%	61.29%	51.43%	-0.622	0.187	0.321
FJ680365.1.1442	p_Firmicutes; c_Clostridia; o_Clostridiales; f_Ruminococcaceae; g_; s_	76.47%	71.43%	74.19%	60.00%	67.74%	51.43%	-0.862	0.027	0.085
AB821809.1.1523	p_Firmicutes; c_Clostridia; o_Clostridiales; f_Ruminococcaceae; g_; s_	88.24%	76.19%	80.65%	72.73%	77.42%	51.43%	-0.867	0.026	0.083
GQ897144.1.1479	p_Firmicutes; c_Clostridia; o_Clostridiales; f_Ruminococcaceae; g_; s_	17.65%	19.05%	22.58%	32.73%	29.03%	54.29%	0.915	0.010	0.050
EU460147.1.1386	p_Firmicutes; c_Clostridia; o_Clostridiales; f_Ruminococcaceae; g_; s_	11.76%	9.52%	16.13%	29.09%	32.26%	54.29%	0.955	0.003	0.026
FAUK01000014.218606.220097	p_Firmicutes; c_Clostridia; o_Clostridiales; f_Ruminococcaceae; g_; s_	20.59%	33.33%	29.03%	32.73%	32.26%	54.29%	0.876	0.022	0.077
EU466081.1.1399	p_Firmicutes; c_Clostridia; o_Clostridiales; f_Ruminococcaceae; g_; s_	44.12%	33.33%	38.71%	45.45%	48.39%	54.29%	0.762	0.078	0.172
HQ780963.1.1453	p_Firmicutes; c_Clostridia; o_Clostridiales; f_Ruminococcaceae; g_; s_	64.71%	52.38%	51.61%	50.91%	48.39%	54.29%	-0.526	0.284	0.424
FJ681917.1.1396	p_Firmicutes; c_Clostridia; o_Clostridiales; f_Ruminococcaceae; g_; s_	55.88%	54.76%	61.29%	43.64%	51.61%	54.29%	-0.246	0.638	0.744
JX095350.1.1407	p_Firmicutes; c_Clostridia; o_Clostridiales; f_Ruminococcaceae; g_; s_	64.71%	69.05%	67.74%	52.73%	51.61%	54.29%	-0.745	0.089	0.188
FJ951877.1.1490	p_Firmicutes; c_Clostridia; o_Clostridiales; f_Ruminococcaceae; g_; s_	70.59%	64.29%	74.19%	60.00%	58.06%	54.29%	-0.822	0.045	0.120
AB821806.1.1501	p_Firmicutes; c_Clostridia; o_Clostridiales; f_Ruminococcaceae; g_; s_	64.71%	52.38%	67.74%	47.27%	61.29%	54.29%	-0.301	0.562	0.685

LC028599.1.1502	p_Firmicutes; c_Clostridia; o_Clostridiales; f_Ruminococcaceae; g_; s_	76.47%	69.05%	67.74%	49.09%	61.29%	54.29%	-0.744	0.090	0.188
JX218358.1.1492	p_Firmicutes; c_Clostridia; o_Clostridiales; f_Ruminococcaceae; g_; s_	67.65%	64.29%	67.74%	50.91%	61.29%	54.29%	-0.666	0.148	0.274
LC028772.1.1502	p_Firmicutes; c_Clostridia; o_Clostridiales; f_Ruminococcaceae; g_; s_	76.47%	57.14%	70.97%	50.91%	64.52%	54.29%	-0.569	0.239	0.379
EU779450.1.1407	p_Firmicutes; c_Clostridia; o_Clostridiales; f_Ruminococcaceae; g_; s_	79.41%	80.95%	83.87%	67.27%	67.74%	54.29%	-0.891	0.017	0.068
EU773276.1.1369	p_Firmicutes; c_Clostridia; o_Clostridiales; f_Ruminococcaceae; g_; s_	52.94%	45.24%	41.94%	34.55%	48.39%	57.14%	0.291	0.575	0.698
EU794111.1.1402	p_Firmicutes; c_Clostridia; o_Clostridiales; f_Ruminococcaceae; g_; s_	76.47%	59.52%	61.29%	58.18%	54.84%	57.14%	-0.741	0.092	0.191
GQ448713.1.1385	p_Firmicutes; c_Clostridia; o_Clostridiales; f_Ruminococcaceae; g_; s_	70.59%	64.29%	77.42%	63.64%	54.84%	57.14%	-0.723	0.104	0.208
LC028649.1.1490	p_Firmicutes; c_Clostridia; o_Clostridiales; f_Ruminococcaceae; g_; s_	64.71%	76.19%	70.97%	56.36%	58.06%	57.14%	-0.649	0.163	0.293
EU794263.1.1453	p_Firmicutes; c_Clostridia; o_Clostridiales; f_Ruminococcaceae; g_; s_	91.18%	69.05%	67.74%	58.18%	64.52%	57.14%	-0.792	0.060	0.143
AF371757.1.1435	p_Firmicutes; c_Clostridia; o_Clostridiales; f_Ruminococcaceae; g_; s_	67.65%	57.14%	80.65%	65.45%	64.52%	57.14%	-0.372	0.468	0.607
JX096145.1.1403	p_Firmicutes; c_Clostridia; o_Clostridiales; f_Ruminococcaceae; g_; s_	79.41%	71.43%	74.19%	54.55%	67.74%	57.14%	-0.740	0.093	0.192
FJ881137.1.1492	p_Firmicutes; c_Clostridia; o_Clostridiales; f_Ruminococcaceae; g_; s_	58.82%	66.67%	67.74%	60.00%	67.74%	57.14%	-0.194	0.713	0.800
AY858446.1.1525	p_Firmicutes; c_Clostridia; o_Clostridiales; f_Ruminococcaceae; g_; s_	73.53%	54.76%	80.65%	61.82%	77.42%	57.14%	-0.231	0.659	0.759
HM124327.1.1483	p_Firmicutes; c_Clostridia; o_Clostridiales; f_Ruminococcaceae; g_; s_	8.82%	14.29%	19.35%	23.64%	22.58%	60.00%	0.898	0.015	0.061
AQOB01000012.1002.2519	p_Firmicutes; c_Clostridia; o_Clostridiales; f_Ruminococcaceae; g_; s_	26.47%	54.76%	32.26%	40.00%	51.61%	60.00%	0.729	0.100	0.201
FJ848420.1.1492	p_Firmicutes; c_Clostridia; o_Clostridiales; f_Ruminococcaceae; g_; s_	73.53%	66.67%	64.52%	60.00%	54.84%	60.00%	-0.821	0.045	0.121
LC028830.1.1516	p_Firmicutes; c_Clostridia; o_Clostridiales; f_Ruminococcaceae; g_; s_	79.41%	64.29%	74.19%	65.45%	58.06%	60.00%	-0.805	0.053	0.132
FJ848430.1.1482	p_Firmicutes; c_Clostridia; o_Clostridiales; f_Ruminococcaceae; g_; s_	88.24%	78.57%	70.97%	60.00%	67.74%	60.00%	-0.838	0.037	0.105
FTRU01000003.3359.4854	p_Firmicutes; c_Clostridia; o_Clostridiales; f_Ruminococcaceae; g_; s_	14.71%	23.81%	16.13%	25.45%	41.94%	62.86%	0.943	0.005	0.033
CDZH01000385.10251.11775	p_Firmicutes; c_Clostridia; o_Clostridiales; f_Ruminococcaceae; g_; s_	35.29%	54.76%	58.06%	52.73%	54.84%	62.86%	0.753	0.084	0.180
JX198636.1.1412	p_Firmicutes; c_Clostridia; o_Clostridiales; f_Ruminococcaceae; g_; s_	67.65%	59.52%	74.19%	61.82%	58.06%	62.86%	-0.378	0.460	0.600
KR514445.1.1442	p_Firmicutes; c_Clostridia; o_Clostridiales; f_Ruminococcaceae; g_; s_	73.53%	66.67%	74.19%	70.91%	58.06%	62.86%	-0.723	0.104	0.208
DQ394655.1.1523	p_Firmicutes; c_Clostridia; o_Clostridiales; f_Ruminococcaceae; g_; s_	79.41%	76.19%	80.65%	76.36%	61.29%	62.86%	-0.864	0.027	0.084
FJ951878.1.1494	p_Firmicutes; c_Clostridia; o_Clostridiales; f_Ruminococcaceae; g_; s_	82.35%	71.43%	77.42%	69.09%	64.52%	62.86%	-0.904	0.013	0.056
FJ848422.1.1512	p_Firmicutes; c_Clostridia; o_Clostridiales; f_Ruminococcaceae; g_; s_	70.59%	61.90%	74.19%	50.91%	67.74%	62.86%	-0.247	0.638	0.744
LC028613.1.1495	p_Firmicutes; c_Clostridia; o_Clostridiales; f_Ruminococcaceae; g_; s_	91.18%	71.43%	70.97%	63.64%	70.97%	62.86%	-0.747	0.088	0.187
GU304571.1.1509	p_Firmicutes; c_Clostridia; o_Clostridiales; f_Ruminococcaceae; g_; s_	82.35%	69.05%	74.19%	67.27%	70.97%	62.86%	-0.798	0.057	0.137
EU794278.1.1449	p_Firmicutes; c_Clostridia; o_Clostridiales; f_Ruminococcaceae; g_; s_	82.35%	64.29%	70.97%	60.00%	74.19%	62.86%	-0.484	0.331	0.473
LC028736.1.1505	p_Firmicutes; c_Clostridia; o_Clostridiales; f_Ruminococcaceae; g_; s_	85.29%	78.57%	80.65%	72.73%	74.19%	62.86%	-0.943	0.005	0.033
JX096127.1.1380	p_Firmicutes; c_Clostridia; o_Clostridiales; f_Ruminococcaceae; g_; s_	73.53%	54.76%	61.29%	52.73%	64.52%	65.71%	-0.055	0.918	0.944
New.ReferenceOTU284	p_Firmicutes; c_Clostridia; o_Clostridiales; f_Ruminococcaceae; g_; s_	82.35%	69.05%	83.87%	56.36%	64.52%	65.71%	-0.572	0.236	0.376
JQ820142.1.1478	p_Firmicutes; c_Clostridia; o_Clostridiales; f_Ruminococcaceae; g_; s_	70.59%	73.81%	67.74%	67.27%	64.52%	65.71%	-0.767	0.075	0.168
EU466103.1.1399	p_Firmicutes; c_Clostridia; o_Clostridiales; f_Ruminococcaceae; g_; s_	67.65%	59.52%	77.42%	56.36%	67.74%	65.71%	-0.039	0.942	0.961
New.CleanUp.ReferenceOTU37874	p_Firmicutes; c_Clostridia; o_Clostridiales; f_Ruminococcaceae; g_; s_	70.59%	54.76%	70.97%	58.18%	67.74%	65.71%	0.016	0.975	0.983
LC028612.1.1527	p_Firmicutes; c_Clostridia; o_Clostridiales; f_Ruminococcaceae; g_; s_	79.41%	78.57%	77.42%	69.09%	74.19%	65.71%	-0.857	0.029	0.088
LC028874.1.1502	p_Firmicutes; c_Clostridia; o_Clostridiales; f_Ruminococcaceae; g_; s_	88.24%	73.81%	77.42%	65.45%	77.42%	65.71%	-0.689	0.130	0.248
KR068088.1.1476	p_Firmicutes; c_Clostridia; o_Clostridiales; f_Ruminococcaceae; g_; s_	70.59%	61.90%	70.97%	58.18%	80.65%	65.71%	0.124	0.815	0.870
JX095377.1.1386	p_Firmicutes; c_Clostridia; o_Clostridiales; f_Ruminococcaceae; g_; s_	64.71%	71.43%	61.29%	65.45%	54.84%	68.57%	-0.149	0.778	0.845
New.ReferenceOTU196	p_Firmicutes; c_Clostridia; o_Clostridiales; f_Ruminococcaceae; g_; s_	79.41%	66.67%	83.87%	65.45%	61.29%	68.57%	-0.526	0.284	0.424
LC028888.1.1512	p_Firmicutes; c_Clostridia; o_Clostridiales; f_Ruminococcaceae; g_; s_	88.24%	57.14%	77.42%	61.82%	70.97%	68.57%	-0.333	0.518	0.650
AB506356.1.1529	p_Firmicutes; c_Clostridia; o_Clostridiales; f_Ruminococcaceae; g_; s_	64.71%	71.43%	80.65%	63.64%	70.97%	68.57%	0.000	1.000	1.000
LC028845.1.1524	p_Firmicutes; c_Clostridia; o_Clostridiales; f_Ruminococcaceae; g_; s_	76.47%	66.67%	74.19%	70.91%	70.97%	68.57%	-0.491	0.322	0.468
LC028865.1.1499	p_Firmicutes; c_Clostridia; o_Clostridiales; f_Ruminococcaceae; g_; s_	82.35%	80.95%	74.19%	67.27%	74.19%	68.57%	-0.764	0.077	0.170
LC028747.1.1522	p_Firmicutes; c_Clostridia; o_Clostridiales; f_Ruminococcaceae; g_; s_	85.29%	69.05%	70.97%	67.27%	67.74%	71.43%	-0.542	0.267	0.406
LC028863.1.1529	p_Firmicutes; c_Clostridia; o_Clostridiales; f_Ruminococcaceae; g_; s_	82.35%	69.05%	80.65%	65.45%	70.97%	71.43%	-0.475	0.341	0.484
AB746761.1.1525	p_Firmicutes; c_Clostridia; o_Clostridiales; f_Ruminococcaceae; g_; s_	64.71%	73.81%	67.74%	69.09%	70.97%	71.43%	0.484	0.331	0.473
AY676490.1.1415	p_Firmicutes; c_Clostridia; o_Clostridiales; f_Ruminococcaceae; g_; s_	79.41%	64.29%	77.42%	52.73%	74.19%	71.43%	-0.122	0.818	0.871
EU794145.1.1433	p_Firmicutes; c_Clostridia; o_Clostridiales; f_Ruminococcaceae; g_; s_	67.65%	52.38%	80.65%	60.00%	61.29%	74.29%	0.212	0.686	0.780
KJ571149.1.1493	p_Firmicutes; c_Clostridia; o_Clostridiales; f_Ruminococcaceae; g_; s_	79.41%	71.43%	77.42%	60.00%	67.74%	74.29%	-0.305	0.557	0.682
EU461012.1.1378	p_Firmicutes; c_Clostridia; o_Clostridiales; f_Ruminococcaceae; g_; s_	85.29%	71.43%	83.87%	65.45%	67.74%	74.29%	-0.491	0.323	0.468
LC028638.1.1501	p_Firmicutes; c_Clostridia; o_Clostridiales; f_Ruminococcaceae; g_; s_	91.18%	71.43%	74.19%	56.36%	74.19%	74.29%	-0.358	0.485	0.627
LC028579.1.1502	p_Firmicutes; c_Clostridia; o_Clostridiales; f_Ruminococcaceae; g_; s_	94.12%	88.10%	80.65%	69.09%	77.42%	74.29%	-0.747	0.088	0.186
JX095623.1.1387	p_Firmicutes; c_Clostridia; o_Clostridiales; f_Ruminococcaceae; g_; s_	76.47%	66.67%	80.65%	63.64%	80.65%	74.29%	0.118	0.824	0.873
LC028594.1.1523	p_Firmicutes; c_Clostridia; o_Clostridiales; f_Ruminococcaceae; g_; s_	91.18%	90.48%	87.10%	81.82%	80.65%	74.29%	-0.975	0.001	0.018
EU474545.1.1386	p_Firmicutes; c_Clostridia; o_Clostridiales; f_Ruminococcaceae; g_; s_	85.29%	76.19%	70.97%	76.36%	87.10%	74.29%	-0.144	0.786	0.849
LC028557.1.1507	p_Firmicutes; c_Clostridia; o_Clostridiales; f_Ruminococcaceae; g_; s_	79.41%	83.33%	80.65%	83.64%	90.32%	74.29%	-0.114	0.829	0.875
EF409956.1.1509	p_Firmicutes; c_Clostridia; o_Clostridiales; f_Ruminococcaceae; g_; s_	17.65%	38.10%	38.71%	50.91%	54.84%	77.14%	0.978	0.001	0.016
FJ848385.1.1467	p_Firmicutes; c_Clostridia; o_Clostridiales; f_Ruminococcaceae; g_; s_	94.12%	78.57%	96.77%	67.27%	74.19%	77.14%	-0.546	0.262	0.403
LC028593.1.1521	p_Firmicutes; c_Clostridia; o_Clostridiales; f_Ruminococcaceae; g_; s_	94.12%	73.81%	83.87%	70.91%	74.19%	77.14%	-0.550	0.259	0.399
LC028684.1.1507	p_Firmicutes; c_Clostridia; o_Clostridiales; f_Ruminococcaceae; g_; s_	91.18%	78.57%	83.87%	76.36%	74.19%	77.14%	-0.734	0.097	0.199
JX096218.1.1415	p_Firmicutes; c_Clostridia; o_Clostridiales; f_Ruminococcaceae; g_; s_	76.47%	80.95%	83.87%	69.09%	77.42%	77.14%	-0.172	0.744	0.824
FJ951884.1.1490	p_Firmicutes; c_Clostridia; o_Clostridiales; f_Ruminococcaceae; g_; s_	97.06%	83.33%	90.32%	72.73%	77.42%	77.14%	-0.734	0.096	0.198
EU772313.1.1392	p_Firmicutes; c_Clostridia; o_Clostridiales; f_Ruminococcaceae; g_; s_	79.41%	78.57%	87.10%	67.27%	67.74%	80.00%	-0.261	0.617	0.722
JX096169.1.1382	p_Firmicutes; c_Clostridia; o_Clostridiales; f_Ruminococcaceae; g_; s_	82.35%	80.95%	77.42%	69.09%	74.19%	80.00%	-0.289	0.578	0.698
LT598592.1.1493	p_Firmicutes; c_Clostridia; o_Clostridiales; f_Ruminococcaceae; g_; s_	91.18%	83.33%	90.32%	70.91%	77.42%	80.00%	-0.568	0.240	0.379
LC028604.1.1535	p_Firmicutes; c_Clostridia; o_Clostridiales; f_Ruminococcaceae; g_; s_	91.18%	80.95%	87.10%	74.55%	77.42%	80.00%	-0.620	0.189	0.323
LC028814.1.1512	p_Firmicutes; c_Clostridia; o_Clostridiales; f_Ruminococcaceae; g_; s_	94.12%	92.86%	100.00%	85.45%	80.65%	80.00%	-0.815	0.048	0.126
GQ451226.1.1497	p_Firmicutes; c_Clostridia; o_Clostridiales; f_Ruminococcaceae; g_; s_	76.47%	64.29%	80.65%	70.91%	83.87%	82.86%	0.563	0.244	0.383
JX096073.1.1385	p_Firmicutes; c_Clostridia; o_Clostridiales; f_Ruminococcaceae; g_; s_	91.18%	78.57%	87.10%	74.55%	83.87%	82.86%	-0.291	0.576	0.698
FJ848437.1.1481	p_Firmicutes; c_Clostridia; o_Clostridiales; f_Ruminococcaceae; g_; s_	91.18%	76.19%	96.77%	81.82%	83.87%	82.86%	-0.280	0.591	0.704
AY854350.1.1536	p_Firmicutes; c_Clostridia; o_Clostridiales; f_Ruminococcaceae; g_; s_	94.12%	85.71%	90.32%	70.91%	77.42%	85.71%	-0.437	0.386	0.533
JX095332.1.1403	p_Firmicutes; c_Clostridia; o_Clostridiales; f_Ruminococcaceae; g_; s_	85.29%	90.48%	96.77%	83.64%	83.87%	85.71%	-0.317	0.540	0.669
DQ105658.1.1522	p_Firmicutes; c_Clostridia; o_Clostridiales; f_Ruminococcaceae; g_; s_	94.12%	88.10%	96.77%	83.64%	87.10%	85.71%	-0.595	0.212	0.350
JX096292.1.1380	p_Firmicutes; c_Clostridia; o_Clostridiales; f_Ruminococcaceae; g_; s_	91.18%	90.48%	96.77%	81.82%	90.32%	85.71%	-0.427	0.398	0.542
JQ248104.1.1509	p_Firmicutes; c_Clostridia; o_Clostridiales; f_Ruminococcaceae; g_; s_	85.29%	80.95%	80.65%	74.55%	64.52%	88.57%	-0.129	0.807	0.867
LC028582.1.1504	p_Firmicutes; c_Clostridia; o_Clostridiales; f_Ruminococcaceae; g_; s_	94.12%	88.10%	96.77%	92.73%	83.87%	88.57%	-0.542	0.266	0.406
LC028652.1.1523	p_Firmicutes; c_Clostridia; o_Clostridiales; f_Ruminococcaceae; g_; s_	94.12%	95.24%	100.00%	90.91%	87.10%	88.57%	-0.694	0.126	0.243
KM978278.1.1535	p_Firmicutes; c_Clostridia; o_Clostridiales; f_Ruminococcaceae; g_; s_	91.18%	80.95%	90.32%	87.27%	93.55%	88.57%	0.218	0.678	0.773
HM124252.1.1494	p_Firmicutes; c_Clostridia; o_Clostridiales; f_Ruminococcaceae; g_; s_	94.12%	88.10%	100.00%	92.73%	96.77%	88.57%	-0.205	0.696	0.788
FJ672837.1.1356	p_Firmicutes; c_Clostridia; o_Clostridiales; f_Ruminococcaceae; g_; s_	76.47%	71.43%	77.42%	60.00%	70.97%	91.43%	0.417	0.411	0.555
LC028618.1.1521	p_Firmicutes; c_Clostridia; o_Clostridiales; f_Ruminococcaceae; g_; s_	91.18%	83.33%	83.87%	78.18%	87.10%	91.43%	0.194	0.713	0.800
LC028745.1.1522	p_Firmicutes; c_Clostridia; o_Clostridiales; f_Ruminococcaceae; g_; s_	94.12%	88.10%	96.77%	83.64%	87.10%	91.43%	-0.266	0.611	0.718
LC028801.1.1523	p_Firmicutes; c_Clostridia; o_Clostridiales; f_Ruminococcaceae; g_; s_	88.24%	83.33%	90.32%	81.82%	90.32%	91.43%	0.430	0.395	0.540
AY854363.1.1528	p_Firmicutes; c_Clostridia; o_Clostridiales; f_Ruminococcaceae; g_; s_	97.06%	92.86%	90.32%	90.91%	90.32%	91.43%	-0.649	0.163	0.293
LC028621.1.1521	p_Firmicutes; c_Clostridia; o_Clostridiales; f_Ruminococcaceae; g_; s_	94.12%	92.86%	96.77%	87.27%	93.55%	91.43%	-0.309	0.551	0.678
LC028771.1.1511	p_Firmicutes; c_Clostridia; o_Clostridiales; f_Ruminococcaceae; g_; s_	94.12%	97.62%	96.77%	96.36%	93.55%	94.29%	-0.405	0.426	0.570
LC028624.1.1525	p_Firmicutes; c_Clostridia; o_Clostridiales; f_Ruminococcaceae; g_; s_	94.12%	97.62%	96.77%	94.55%	96.77%	94.29%	-0.152	0.774	0.844
LC028664.1.1523	p_Firmicutes; c_Clostridia; o_Clostridiales; f_Ruminococcaceae; g_; s_	94.12%	97.62%	96.77%	94.55%	96.77%	94.29%	-0.152	0.774	0.844
AB506345.1.1524	p_Firmicutes; c_Clostridia; o_Clostridiales; f_Ruminococcaceae; g_; s_	91.18%	92.86%	96.77%	85.45%	100.00%	100.00%	0.579	0.229	0.369
LC028650.1.1523	p_Firmicutes; c_Clostridia; o_Clostridiales; f_Ruminococcaceae; g_; s_	100.00%	100.00%	100.00%	96.36%	100.00%	100.00%	-0.019	0.972	0.982
LC028668.1.1525	p_Firmicutes; c_Clostridia; o_Clostridiales; f_Ruminococcaceae; g_; s_	97.06%	97.62%	100.00%	100.00%	100.00%	100.00%	0.760	0.079	0.173
GQ867365.1.1473	p_Firmicutes; c_Clostridia; o_Clostridiales; f_Ruminococcaceae; g_Faecalibacterium; s_prausnitzii	70.59%	50.00%	67.74%	56.36%	58.06%	40.00%	-0.733	0.097	0.199
DQ804563.1.1391	p_Firmicutes; c_Clostridia; o_Clostridiales; f_Ruminococcaceae; g_Faecalibacterium; s_prausnitzii	11.76%	21.43%	25.81%	32.73%	41.94%	62.86%	0.993	0.000	0.008
CYGL01005916.57.1552	p_Firmicutes; c_Clostridia; o_Clostridiales; f_Ruminococcaceae; g_Faecalibacterium; s_prausnitzii	29.41%	52.38%	48.39%	54.55%	58.06%	80.00%	0.938	0.006	0.036
CBXK010002535.1192.2697	p_Firmicutes; c_Clostridia; o_Clostridiales; f_Ruminococcaceae; g_Faecalibacterium; s_prausnitzii	41.18%	57.14%	54.84%	67.27%	70.97%	88.57%	0.977	0.001	0.016
AB606386.1.1498	p_Firmicutes; c_Clostridia; o_Clostridiales; f_Ruminococcaceae; g_Oscillospira; s_	55.88%	42.86%	41.94%	34.55%	29.03%	8.57%	-0.984	0.000	0.011
FJ848441.1.1500	p_Firmicutes; c_Clostridia; o_Clostridiales; f_Ruminococcaceae; g_Oscillospira; s_	55.88%	26.19%	32.26%	27.27%	12.90%	14.29%	-0.858	0.029	0.088
New.ReferenceOTU290	p_Firmicutes; c_Clostridia; o_Clostridiales; f_Ruminococcaceae; g_Oscillospira; s_	61.76%	47.62%	51.61%	36.36%	38.71%	25.71%	-0.936	0.006	0.036
New.ReferenceOTU1	p_Firmicutes; c_Clostridia; o_Clostridiales; f_Ruminococcaceae; g_Oscillospira; s_	79.41%	50.00%	51.61%	50.91%	41.94%	34.29%	-0.877	0.022	0.077
FJ880076.1.1490	p_Firmicutes; c_Clostridia; o_Clostridiales; f_Ruminococcaceae; g_Oscillospira; s_	35.29%	40.48%	41.94%	38.18%	45.16%	40.00%	0.504	0.307	0.452
New.CleanUp.ReferenceOTU37760	p_Firmicutes; c_Clostridia; o_Clostridiales; f_Ruminococcaceae; g_Oscillospira; s_	44.12%	42.86%	51.61%	50.91%	38.71%	48.57%	0.054	0.919	0.944
JX095160.1.1399	p_Firmicutes; c_Clostridia; o_Clostridiales; f_Ruminococcaceae; g_Oscillospira; s_	85.29%	78.57%	77.42%	65.45%	64.52%	48.57%	-0.976	0.001	0.017
EF404039.1.1494	p_Firmicutes; c_Clostridia; o_Clostridiales; f_Ruminococcaceae; g_Oscillospira; s_	14.71%	19.05%	25.81%	23.64%	29.03%	54.29%	0.922	0.009	0.045
New.CleanUp.ReferenceOTU26561	p_Firmicutes; c_Clostridia; o_Clostridiales; f_Ruminococcaceae; g_Oscillospira; s_	14.71%	14.29%	16.13%	29.09%	35.48%	54.29%	0.959	0.003	0.024
FPLO01006472.6.1515	p_Firmicutes; c_Clostridia; o_Clostridiales; f_Ruminococcaceae; g_Oscillospira; s_	23.53%	14.29%	16.13%	30.91%	35.48%	57.14%	0.877	0.022	0.077

EU464123.1.1390	p_Firmicutes; c_Clostridia; o_Clostridiales; f_Ruminococcaceae; g_Oscillospira; s_	73.53%	52.38%	70.97%	60.00%	61.29%	57.14%	-0.471	0.346	0.488
New.ReferenceOTU77	p_Firmicutes; c_Clostridia; o_Clostridiales; f_Ruminococcaceae; g_Oscillospira; s_	79.41%	61.90%	74.19%	56.36%	74.19%	57.14%	-0.505	0.307	0.452
CDTY01028331.429.1939	p_Firmicutes; c_Clostridia; o_Clostridiales; f_Ruminococcaceae; g_Oscillospira; s_	11.76%	16.67%	32.26%	29.09%	25.81%	60.00%	0.874	0.023	0.078
HG970987.1.1486	p_Firmicutes; c_Clostridia; o_Clostridiales; f_Ruminococcaceae; g_Oscillospira; s_	35.29%	26.19%	38.71%	38.18%	38.71%	60.00%	0.816	0.048	0.126
FJ880741.1.1488	p_Firmicutes; c_Clostridia; o_Clostridiales; f_Ruminococcaceae; g_Oscillospira; s_	64.71%	57.14%	45.16%	56.36%	61.29%	62.86%	0.189	0.720	0.805
HK693831.1.1471	p_Firmicutes; c_Clostridia; o_Clostridiales; f_Ruminococcaceae; g_Oscillospira; s_	61.76%	40.48%	64.52%	60.00%	64.52%	62.86%	0.366	0.476	0.616
LC028655.1.1520	p_Firmicutes; c_Clostridia; o_Clostridiales; f_Ruminococcaceae; g_Oscillospira; s_	94.12%	85.71%	90.32%	76.36%	74.19%	62.86%	-0.958	0.003	0.024
BAGW01000037.287.1796	p_Firmicutes; c_Clostridia; o_Clostridiales; f_Ruminococcaceae; g_Oscillospira; s_	88.24%	50.00%	70.97%	61.82%	67.74%	65.71%	-0.289	0.579	0.698
HQ716472.1.1476	p_Firmicutes; c_Clostridia; o_Clostridiales; f_Ruminococcaceae; g_Oscillospira; s_	82.35%	61.90%	61.29%	69.09%	83.87%	68.57%	0.018	0.973	0.982
FMGI01000008.23183.24694	p_Firmicutes; c_Clostridia; o_Clostridiales; f_Ruminococcaceae; g_Oscillospira; s_	70.59%	61.90%	54.84%	54.55%	74.19%	71.43%	0.341	0.508	0.639
JX198644.1.1488	p_Firmicutes; c_Clostridia; o_Clostridiales; f_Ruminococcaceae; g_Oscillospira; s_	85.29%	71.43%	83.87%	67.27%	83.87%	74.29%	-0.228	0.664	0.761
LC028773.1.1520	p_Firmicutes; c_Clostridia; o_Clostridiales; f_Ruminococcaceae; g_Oscillospira; s_	82.35%	80.95%	74.19%	83.64%	83.87%	77.14%	-0.151	0.775	0.844
CWJP01000005.3412.4922	p_Firmicutes; c_Clostridia; o_Clostridiales; f_Ruminococcaceae; g_Oscillospira; s_	97.06%	85.71%	90.32%	81.82%	90.32%	80.00%	-0.680	0.137	0.258
FJ681256.1.1398	p_Firmicutes; c_Clostridia; o_Clostridiales; f_Ruminococcaceae; g_Oscillospira; s_	32.35%	45.24%	64.52%	61.82%	77.42%	88.57%	0.964	0.002	0.021
EU772321.1.1393	p_Firmicutes; c_Clostridia; o_Clostridiales; f_Ruminococcaceae; g_Oscillospira; s_	58.82%	61.90%	61.29%	72.73%	80.65%	88.57%	0.972	0.001	0.018
GQ448391.1.1388	p_Firmicutes; c_Clostridia; o_Clostridiales; f_Ruminococcaceae; g_Oscillospira; s_	91.18%	85.71%	96.77%	90.91%	90.32%	91.43%	0.080	0.880	0.911
EU794236.1.1450	p_Firmicutes; c_Clostridia; o_Clostridiales; f_Ruminococcaceae; g_Oscillospira; s_	94.12%	92.86%	100.00%	89.09%	96.77%	91.43%	-0.191	0.716	0.802
LC028804.1.1521	p_Firmicutes; c_Clostridia; o_Clostridiales; f_Ruminococcaceae; g_Oscillospira; s_	94.12%	85.71%	100.00%	89.09%	100.00%	91.43%	0.116	0.826	0.873
AY858452.1.1523	p_Firmicutes; c_Clostridia; o_Clostridiales; f_Ruminococcaceae; g_Oscillospira; s_	52.94%	71.43%	70.97%	80.00%	80.65%	94.29%	0.948	0.004	0.030
New.ReferenceOTU345	p_Firmicutes; c_Clostridia; o_Clostridiales; f_Ruminococcaceae; g_Oscillospira; s_	91.18%	85.71%	100.00%	85.45%	96.77%	94.29%	0.304	0.557	0.682
LC028793.1.1523	p_Firmicutes; c_Clostridia; o_Clostridiales; f_Ruminococcaceae; g_Oscillospira; s_	100.00%	85.71%	100.00%	89.09%	96.77%	97.14%	0.085	0.873	0.907
EU794273.1.1451	p_Firmicutes; c_Clostridia; o_Clostridiales; f_Ruminococcaceae; g_Oscillospira; s_	97.06%	97.62%	96.77%	98.18%	93.55%	100.00%	0.168	0.750	0.828
KR514395.1.1452	p_Firmicutes; c_Clostridia; o_Clostridiales; f_Ruminococcaceae; g_Oscillospira; s_	100.00%	95.24%	100.00%	98.18%	100.00%	100.00%	0.324	0.531	0.661
AB627509.1.1525	p_Firmicutes; c_Clostridia; o_Clostridiales; f_Ruminococcaceae; g_Oscillospira; s_	97.06%	97.62%	100.00%	100.00%	100.00%	100.00%	0.760	0.079	0.173
AB746831.1.1523	p_Firmicutes; c_Clostridia; o_Clostridiales; f_Ruminococcaceae; g_Ruminococcus; s_	58.82%	38.10%	29.03%	30.91%	35.48%	31.43%	-0.631	0.179	0.310
EF404262.1.1485	p_Firmicutes; c_Clostridia; o_Clostridiales; f_Ruminococcaceae; g_Ruminococcus; s_	44.12%	45.24%	48.39%	50.91%	48.39%	37.14%	-0.401	0.431	0.575
New.ReferenceOTU161	p_Firmicutes; c_Clostridia; o_Clostridiales; f_Ruminococcaceae; g_Ruminococcus; s_	70.59%	47.62%	64.52%	52.73%	51.61%	48.57%	-0.645	0.167	0.296
FJ503849.1.1375	p_Firmicutes; c_Clostridia; o_Clostridiales; f_Ruminococcaceae; g_Ruminococcus; s_	8.82%	11.90%	9.68%	14.55%	19.35%	51.43%	0.873	0.023	0.078
LAQZ01000049.1994.3490	p_Firmicutes; c_Clostridia; o_Clostridiales; f_Ruminococcaceae; g_Ruminococcus; s_	64.71%	57.14%	64.52%	56.36%	54.84%	57.14%	-0.635	0.175	0.306
AB506550.1.1526	p_Firmicutes; c_Clostridia; o_Clostridiales; f_Ruminococcaceae; g_Ruminococcus; s_	47.06%	64.29%	67.74%	63.64%	64.52%	62.86%	0.493	0.320	0.466
New.ReferenceOTU265	p_Firmicutes; c_Clostridia; o_Clostridiales; f_Ruminococcaceae; g_Ruminococcus; s_	76.47%	66.67%	67.74%	61.82%	67.74%	65.71%	-0.559	0.249	0.387
AB746644.1.1524	p_Firmicutes; c_Clostridia; o_Clostridiales; f_Ruminococcaceae; g_Ruminococcus; s_	58.82%	64.29%	41.94%	56.36%	67.74%	68.57%	0.478	0.338	0.481
AB746539.1.1522	p_Firmicutes; c_Clostridia; o_Clostridiales; f_Ruminococcaceae; g_Ruminococcus; s_	88.24%	88.10%	87.10%	89.09%	96.77%	94.29%	0.804	0.054	0.132
ADMB01000074.90.1632	p_Firmicutes; c_Clostridia; o_Clostridiales; f_Veillonellaceae; g_Megamonas; s_	2.94%	14.29%	16.13%	23.64%	25.81%	57.14%	0.952	0.003	0.027
LC028606.1.1547	p_Firmicutes; c_Clostridia; o_Clostridiales; f_Veillonellaceae; g_Phascolarctobacterium; s_	85.29%	61.90%	64.52%	60.00%	51.61%	25.71%	-0.957	0.003	0.025
EU776905.1.1410	p_Firmicutes; c_Clostridia; o_Clostridiales; f_Veillonellaceae; g_Phascolarctobacterium; s_	50.00%	28.57%	32.26%	40.00%	45.16%	28.57%	-0.350	0.497	0.634
LC028605.1.1547	p_Firmicutes; c_Clostridia; o_Clostridiales; f_Veillonellaceae; g_Phascolarctobacterium; s_	52.94%	47.62%	54.84%	56.36%	45.16%	42.86%	-0.651	0.162	0.293
KC245244.1.1485	p_Firmicutes; c_Clostridia; o_Clostridiales; f_Veillonellaceae; g_Phascolarctobacterium; s_	58.82%	40.48%	48.39%	50.91%	67.74%	51.43%	0.233	0.657	0.759
New.ReferenceOTU321	p_Firmicutes; c_Clostridia; o_Clostridiales; f_Veillonellaceae; g_Phascolarctobacterium; s_	17.65%	28.57%	38.71%	41.82%	41.94%	54.29%	0.942	0.005	0.033
EU774211.1.1284	p_Firmicutes; c_Clostridia; o_Clostridiales; f_Veillonellaceae; g_Phascolarctobacterium; s_	8.82%	19.05%	25.81%	29.09%	48.39%	54.29%	0.985	0.000	0.011
EU531937.1.1451	p_Firmicutes; c_Clostridia; o_Clostridiales; f_Veillonellaceae; g_Phascolarctobacterium; s_	52.94%	54.76%	51.61%	60.00%	61.29%	57.14%	0.615	0.193	0.328
New.ReferenceOTU253	p_Firmicutes; c_Clostridia; o_Clostridiales; f_Veillonellaceae; g_Phascolarctobacterium; s_	88.24%	69.05%	61.29%	54.55%	64.52%	57.14%	-0.710	0.114	0.224
New.ReferenceOTU12	p_Firmicutes; c_Clostridia; o_Clostridiales; f_Veillonellaceae; g_Phascolarctobacterium; s_	29.41%	26.19%	41.94%	45.45%	51.61%	60.00%	0.943	0.005	0.033
AB490812.1.1611	p_Firmicutes; c_Clostridia; o_Clostridiales; f_Veillonellaceae; g_Phascolarctobacterium; s_	91.18%	80.95%	77.42%	78.18%	74.19%	65.71%	-0.953	0.003	0.027
New.CleanUp.ReferenceOTU36677	p_Firmicutes; c_Clostridia; o_Clostridiales; f_Veillonellaceae; g_Phascolarctobacterium; s_	23.53%	30.95%	41.94%	52.73%	67.74%	68.57%	0.959	0.003	0.024
ADGC01007328.3448.5099	p_Firmicutes; c_Clostridia; o_Clostridiales; f_Veillonellaceae; g_Phascolarctobacterium; s_	100.00%	100.00%	100.00%	100.00%	100.00%	100.00%	NA	NA	NA
AB824377.1.1549	p_Firmicutes; c_Clostridia; o_Clostridiales; f_Veillonellaceae; g_Succiniclasticum; s_	32.35%	40.48%	41.94%	47.27%	51.61%	45.71%	0.777	0.069	0.159
New.ReferenceOTU317	p_Firmicutes; c_Clostridia; o_Clostridiales; f_Veillonellaceae; g_Succiniclasticum; s_	38.24%	52.38%	32.26%	50.91%	45.16%	54.29%	0.519	0.291	0.431
AB185593.1.1557	p_Firmicutes; c_Clostridia; o_Clostridiales; f_Veillonellaceae; g_Succiniclasticum; s_	47.06%	54.76%	64.52%	61.82%	61.29%	60.00%	0.598	0.210	0.347
EU096495.1.1449	p_Firmicutes; c_Clostridia; o_Clostridiales; f_Veillonellaceae; g_Veillonella; s_	26.47%	45.24%	29.03%	43.64%	51.61%	48.57%	0.742	0.091	0.191
EU772340.1.1327	p_Firmicutes; c_Erysipelotrichi; o_Erysipelotrichales; f_Erysipelotrichaceae; g_; s_	61.76%	52.38%	45.16%	36.36%	29.03%	20.00%	-0.987	0.000	0.010
New.ReferenceOTU362	p_Firmicutes; c_Erysipelotrichi; o_Erysipelotrichales; f_Erysipelotrichaceae; g_; s_	52.94%	28.57%	32.26%	38.18%	35.48%	22.86%	-0.684	0.134	0.253
EU475451.1.1382	p_Firmicutes; c_Erysipelotrichi; o_Erysipelotrichales; f_Erysipelotrichaceae; g_; s_	67.65%	52.38%	64.52%	47.27%	58.06%	31.43%	-0.785	0.065	0.151
EU474466.1.1374	p_Firmicutes; c_Erysipelotrichi; o_Erysipelotrichales; f_Erysipelotrichaceae; g_; s_	64.71%	54.76%	61.29%	47.27%	29.03%	34.29%	-0.895	0.016	0.063
EU466211.1.1382	p_Firmicutes; c_Erysipelotrichi; o_Erysipelotrichales; f_Erysipelotrichaceae; g_; s_	70.59%	47.62%	58.06%	58.18%	54.84%	37.14%	-0.763	0.078	0.171
FJ680618.1.1439	p_Firmicutes; c_Erysipelotrichi; o_Erysipelotrichales; f_Erysipelotrichaceae; g_; s_	38.24%	28.57%	48.39%	40.00%	38.71%	54.29%	0.624	0.186	0.319
FJ681221.1.1463	p_Firmicutes; c_Erysipelotrichi; o_Erysipelotrichales; f_Erysipelotrichaceae; g_; s_	52.94%	47.62%	70.97%	50.91%	48.39%	54.29%	-0.098	0.853	0.892
LK021123.1.1724	p_Firmicutes; c_Erysipelotrichi; o_Erysipelotrichales; f_Erysipelotrichaceae; g_; s_	20.59%	33.33%	22.58%	43.64%	35.48%	65.71%	0.865	0.026	0.083
KF698526.1.1421	p_Firmicutes; c_Erysipelotrichi; o_Erysipelotrichales; f_Erysipelotrichaceae; g_; s_	47.06%	45.24%	48.39%	49.09%	54.84%	65.71%	0.923	0.009	0.045
EU794282.1.1445	p_Firmicutes; c_Erysipelotrichi; o_Erysipelotrichales; f_Erysipelotrichaceae; g_; s_	35.29%	47.62%	38.71%	49.09%	64.52%	65.71%	0.920	0.009	0.046
JX096253.1.1392	p_Firmicutes; c_Erysipelotrichi; o_Erysipelotrichales; f_Erysipelotrichaceae; g_; s_	61.76%	59.52%	80.65%	56.36%	70.97%	74.29%	0.425	0.401	0.544
GQ449198.1.1372	p_Firmicutes; c_Erysipelotrichi; o_Erysipelotrichales; f_Erysipelotrichaceae; g_[Eubacterium]; s_dolichum	52.94%	69.05%	61.29%	69.09%	61.29%	62.86%	0.262	0.616	0.722
FJ680600.1.1472	p_Firmicutes; c_Erysipelotrichi; o_Erysipelotrichales; f_Erysipelotrichaceae; g_Coprobacillus; s_	17.65%	30.95%	25.81%	54.55%	41.94%	37.14%	0.567	0.241	0.380
JX096221.1.1411	p_Planctomycetes; c_Planctomycetia; o_Pirellulales; f_Pirellulaceae; g_; s_	58.82%	47.62%	29.03%	38.18%	29.03%	17.14%	-0.906	0.013	0.055
AB270056.1.1531	p_Planctomycetes; c_Planctomycetia; o_Pirellulales; f_Pirellulaceae; g_; s_	52.94%	59.52%	70.97%	58.18%	61.29%	42.86%	-0.436	0.387	0.533
EU460205.1.1360	p_Proteobacteria; c_Alphaproteobacteria; o_RF32; f_; g_; s_	29.41%	30.95%	41.94%	38.18%	58.06%	45.71%	0.760	0.079	0.173
GQ898126.1.1466	p_Proteobacteria; c_Alphaproteobacteria; o_RF32; f_; g_; s_	20.59%	33.33%	51.61%	49.09%	64.52%	57.14%	0.845	0.034	0.098
EU794199.1.1457	p_Proteobacteria; c_Betaproteobacteria; o_Burkholderiales; f_Alcaligenaceae	79.41%	59.52%	51.61%	40.00%	45.16%	22.86%	-0.935	0.006	0.037
New.ReferenceOTU69	p_Proteobacteria; c_Betaproteobacteria; o_Burkholderiales; f_Alcaligenaceae; g_Sutterella; s_	70.59%	57.14%	48.39%	41.82%	19.35%	17.14%	-0.972	0.001	0.018
New.ReferenceOTU250	p_Proteobacteria; c_Betaproteobacteria; o_Burkholderiales; f_Alcaligenaceae; g_Sutterella; s_	20.59%	30.95%	32.26%	21.82%	51.61%	45.71%	0.787	0.063	0.148
NFZD01016365.1.1230	p_Proteobacteria; c_Betaproteobacteria; o_Burkholderiales; f_Alcaligenaceae; g_Sutterella; s_	26.47%	57.14%	48.39%	43.64%	35.48%	60.00%	0.466	0.351	0.493
CEAC01010956.3592.5108	p_Proteobacteria; c_Betaproteobacteria; o_Burkholderiales; f_Alcaligenaceae; g_Sutterella; s_	11.76%	30.95%	35.48%	47.27%	64.52%	74.29%	0.987	0.000	0.010
FPLO01007446.12.1527	p_Proteobacteria; c_Betaproteobacteria; o_Burkholderiales; f_Alcaligenaceae; g_Sutterella; s_	44.12%	66.67%	70.97%	87.27%	90.32%	94.29%	0.913	0.011	0.052
EU470175.1.1394	p_Proteobacteria; c_Deltaproteobacteria; o_Desulfovibrionales; f_Desulfovibrionaceae; g_; s_	82.35%	71.43%	70.97%	52.73%	61.29%	40.00%	-0.919	0.010	0.046
FTLM01000021.103.1630	p_Proteobacteria; c_Deltaproteobacteria; o_Desulfovibrionales; f_Desulfovibrionaceae; g_; s_	14.71%	33.33%	45.16%	50.91%	70.97%	71.43%	0.953	0.003	0.027
CDZU01018059.20708.22237	p_Proteobacteria; c_Deltaproteobacteria; o_Desulfovibrionales; f_Desulfovibrionaceae; g_Desulfovibrio; s_	47.06%	47.62%	45.16%	54.55%	54.84%	65.71%	0.910	0.012	0.053
CP018789.273377.275106	p_Proteobacteria; c_Epsilonproteobacteria; o_Campylobacterales; f_Campylobacteraceae; g_Campylobacter; s_	97.06%	97.62%	96.77%	94.55%	93.55%	85.71%	-0.911	0.011	0.052
FPID01000788.5.1506	p_Proteobacteria; c_Gammaproteobacteria; o_Enterobacteriales; f_Enterobacteriaceae; g_; s_	29.41%	50.00%	22.58%	25.45%	16.13%	28.57%	-0.388	0.448	0.592
DQ818972.1.1483	p_Proteobacteria; c_Gammaproteobacteria; o_Enterobacteriales; f_Enterobacteriaceae; g_; s_	26.47%	52.38%	25.81%	36.36%	29.03%	28.57%	-0.232	0.658	0.759
AB855728.1.1528	p_Proteobacteria; c_Gammaproteobacteria; o_Enterobacteriales; f_Enterobacteriaceae; g_; s_	50.00%	57.14%	32.26%	47.27%	35.48%	42.86%	-0.425	0.401	0.544
KF625183.1.1786	p_Proteobacteria; c_Gammaproteobacteria; o_Enterobacteriales; f_Enterobacteriaceae; g_; s_	52.94%	71.43%	64.52%	54.55%	38.71%	48.57%	-0.577	0.230	0.370
FPID01000474.6.1490	p_Proteobacteria; c_Gammaproteobacteria; o_Enterobacteriales; f_Enterobacteriaceae; g_; s_	70.59%	61.90%	67.74%	50.91%	54.84%	65.71%	-0.327	0.528	0.659
CCPS01000022.154.1916	p_Proteobacteria; c_Gammaproteobacteria; o_Enterobacteriales; f_Enterobacteriaceae; g_; s_	100.00%	100.00%	100.00%	100.00%	100.00%	100.00%	NA	NA	NA
KM116008.1.1667	p_Proteobacteria; c_Gammaproteobacteria; o_Enterobacteriales; f_Enterobacteriaceae; g_Trabulsiella	55.88%	78.57%	67.74%	60.00%	61.29%	71.43%	0.197	0.709	0.798
JTJL01000052.415.1962	p_Proteobacteria; c_Gammaproteobacteria; o_Pasteurellales; f_Pasteurellaceae; g_Gallibacterium; s_	88.24%	90.48%	90.32%	81.82%	96.77%	82.86%	-0.170	0.747	0.826
EU776449.1.1402	p_Spirochaetes; c_Spirochaetes; o_Spirochaetales; f_Spirochaetaceae; g_Treponema; s_	52.94%	28.57%	35.48%	16.36%	16.13%	11.43%	-0.877	0.022	0.077
AB185596.1.1545	p_Spirochaetes; c_Spirochaetes; o_Spirochaetales; f_Spirochaetaceae; g_Treponema; s_	52.94%	52.38%	61.29%	56.36%	58.06%	60.00%	0.654	0.159	0.289
EU794279.1.1444	p_Tenericutes; c_Mollicutes; o_Anaeroplasmatales; f_Anaeroplasmataceae; g_; s_	82.35%	83.33%	87.10%	70.91%	67.74%	51.43%	-0.911	0.011	0.052
New.ReferenceOTU160	p_Tenericutes; c_Mollicutes; o_Anaeroplasmatales; f_Anaeroplasmataceae; g_Anaeroplasma; s_	61.76%	54.76%	61.29%	45.45%	38.71%	31.43%	-0.943	0.005	0.033
KF843061.1.1424	p_Tenericutes; c_Mollicutes; o_RF39; f_; g_; s_	38.24%	30.95%	51.61%	45.45%	45.16%	31.43%	-0.125	0.814	0.870
CEAB01016577.1.1534	p_Tenericutes; c_Mollicutes; o_RF39; f_; g_; s_	52.94%	45.24%	41.94%	34.55%	16.13%	37.14%	-0.670	0.145	0.271
CDYY01027434.449.2003	p_Tenericutes; c_Mollicutes; o_RF39; f_; g_; s_	29.41%	35.71%	51.61%	38.18%	32.26%	37.14%	0.049	0.927	0.949
JX218855.1.1553	p_Tenericutes; c_Mollicutes; o_RF39; f_; g_; s_	52.94%	47.62%	54.84%	58.18%	54.84%	45.71%	-0.279	0.592	0.704
EU469995.1.1302	p_Tenericutes; c_Mollicutes; o_RF39; f_; g_; s_	76.47%	52.38%	67.74%	50.91%	54.84%	48.57%	-0.717	0.109	0.215
AY916191.1.1394	p_Tenericutes; c_Mollicutes; o_RF39; f_; g_; s_	5.88%	11.90%	16.13%	18.18%	16.13%	51.43%	0.871	0.024	0.078
New.ReferenceOTU146	p_Tenericutes; c_Mollicutes; o_RF39; f_; g_; s_	52.94%	52.38%	64.52%	58.18%	67.74%	54.29%	0.278	0.594	0.704
JX096311.1.1429	p_Tenericutes; c_Mollicutes; o_RF39; f_; g_; s_	67.65%	52.38%	58.06%	61.82%	64.52%	60.00%	-0.015	0.977	0.984
JX096326.1.1403	p_Tenericutes; c_RF3; o_ML615J-28; f_; g_; s_	61.76%	33.33%	35.48%	25.45%	29.03%	14.29%	-0.868	0.025	0.082
JX096375.1.1403	p_Tenericutes; c_RF3; o_ML615J-28; f_; g_; s_	32.35%	50.00%	51.61%	38.18%	41.94%	48.57%	0.343	0.506	0.639
New.ReferenceOTU131	p_Tenericutes; c_RF3; o_ML615J-28; f_; g_; s_	61.76%	50.00%	64.52%	50.91%	48.39%	48.57%	-0.648	0.164	0.293
New.ReferenceOTU66	p_Tenericutes; c_RF3; o_ML615J-28; f_; g_; s_	70.59%	45.24%	64.52%	41.82%	38.71%	51.43%	-0.526	0.284	0.424
EU474778.1.1412	p_Verrucomicrobia; c_Verruco-5; o_WCHB1-41; f_RFP12; g_; s_	61.76%	50.00%	51.61%	45.45%	41.94%	22.86%	-0.965	0.002	0.021
EU474711.1.1281	p_Verrucomicrobia; c_Verrucomicrobiae; o_Verrucomicrobiales; f_Verrucomicrobiaceae; g_Akkermansia; s_	50.00%	33.33%	41.94%	21.82%	22.58%	5.71%	-0.936	0.006	0.036
FJ951846.1.1467	p_Verrucomicrobia; c_Verrucomicrobiae; o_Verrucomicrobiales; f_Verrucomicrobiaceae; g_Akkermansia; s_	58.82%	47.62%	48.39%	20.00%	16.13%	8.57%	-0.938	0.006	0.036
EU470026.1.1361	p_Verrucomicrobia; c_Verrucomicrobiae; o_Verrucomicrobiales; f_Verrucomicrobiaceae; g_Akkermansia; s_	52.94%	42.86%	35.48%	20.00%	16.13%	11.43%	-0.946	0.004	0.032

EU468820.1.1267	p_Verrucomicrobia; c_Verrucomicrobiae; o_Verrucomicrobiales; f_Verrucomicrobiaceae; g_Akkermansia; s_	55.88%	45.24%	38.71%	30.91%	16.13%	14.29%	-0.971	0.001	0.018
LC028555.1.1493	p_Verrucomicrobia; c_Verrucomicrobiae; o_Verrucomicrobiales; f_Verrucomicrobiaceae; g_Akkermansia; s_	82.35%	66.67%	64.52%	45.45%	41.94%	34.29%	-0.953	0.003	0.027
