# Supplementary material for: Host genetic effects upon the early gut microbiota in a bovine model with graduated spectrum of genetic variation
Source: ISME J. 2019 Oct 17;14(1):302–17. doi: 10.1038/s41396-019-0529-2 (PMC6908690; doi:10.1038/s41396-019-0529-2)
Supplement: Supplementary file 5 — Supplementary Table S4. Influences of age, Brahman proportion and gender on growth, plasma parameters, and gut microbiota of MAB1 preweaning calves reflected from the multiple linear regression model [file 41396_2019_529_MOESM5_ESM.pdf]

Supplementary Table S4. Influences of age, Brahman proportion and gender on growth, plasma parameters, and gut microbiota of MAB[1] preweaning calves reflected from the multiple linear regression model.

Response variables	Explanatory variables					
	Age in days		Brahman proportion		Gender	
	Coefficient	*P* value	Coefficient	*P* value	Coefficient[2]	*P* value
Weight gain	0.980	< 2e-16	-10.720	2.690E-03	-8.553	5.950E-06
Glucose	-0.016	3.330E-06	0.392	5.043E-02	0.341	1.540E-03
Non-esterified fatty acid (NEFA)	0.001	1.150E-01	0.066	2.216E-02	NA[3]	NA
Immunoglobulin G1 (IgG1)	NA	NA	-2.089	1.580E-04	NA	NA
Chao 1	7.513	1.970E-06	NA	NA	NA	NA
Shannon	0.018	4.050E-11	NA	NA	0.123	1.540E-01
p_Actinobacteria	NA	NA	NA	NA	NA	NA
p_Bacteroidetes	-0.003	3.040E-06	NA	NA	0.045	3.224E-02
p_Chloroflexi	0.011	1.430E-07	0.258	4.380E-02	NA	NA
p_Cyanobacteria	-0.004	2.680E-02	NA	NA	NA	NA
p_Firmicutes	0.001	4.450E-03	NA	NA	NA	NA
p_Planctomycetes	0.014	3.750E-08	-0.547	2.300E-04	NA	NA
p_Proteobacteria	NA	NA	NA	NA	NA	NA
p_Tenericutes	0.007	2.360E-04	NA	NA	NA	NA
p_Verrucomicrobia	0.014	8.660E-07	-0.518	2.040E-03	NA	NA
p_Actinobacteria;c_Coriobacteriia;o_Coriobacteriales;f_Coriobacteriaceae	NA	NA	0.230	3.130E-02	0.086	1.256E-01
p_Bacteroidetes;c_Bacteroidia;o_Bacteroidales;f_Bacteroidaceae	-0.005	6.120E-08	NA	NA	NA	NA
p_Bacteroidetes;c_Bacteroidia;o_Bacteroidales;f_Porphyromonadaceae	NA	NA	NA	NA	NA	NA
p_Bacteroidetes;c_Bacteroidia;o_Bacteroidales;f_Prevotellaceae	-0.018	7.090E-10	0.537	2.440E-03	NA	NA
p_Bacteroidetes;c_Bacteroidia;o_Bacteroidales;f_Rikenellaceae	0.018	1.550E-09	-0.501	5.590E-03	NA	NA
p_Bacteroidetes;c_Bacteroidia;o_Bacteroidales;f_S24-7	-0.006	2.060E-03	NA	NA	0.151	1.440E-02
p_Bacteroidetes;c_Bacteroidia;o_Bacteroidales;f_[Barnesiellaceae]	0.003	1.523E-01	-0.277	6.478E-02	0.205	9.150E-03
p_Bacteroidetes;c_Bacteroidia;o_Bacteroidales;f_[Odoribacteraceae]	-0.015	3.700E-10	0.214	1.320E-01	NA	NA
p_Bacteroidetes;c_Bacteroidia;o_Bacteroidales;f_[Paraprevotellaceae]	-0.011	1.820E-06	NA	NA	0.243	5.680E-04
p_Firmicutes;c_Bacilli;o_Bacillales;f_Bacillaceae	0.017	<2e-16	NA	NA	NA	NA
p_Firmicutes;c_Bacilli;o_Bacillales;f_Planococcaceae	0.015	6.460E-10	NA	NA	NA	NA
p_Firmicutes;c_Bacilli;o_Lactobacillales;f_Lactobacillaceae	NA	NA	NA	NA	NA	NA
p_Firmicutes;c_Bacilli;o_Lactobacillales;f_Streptococcaceae	0.014	2.090E-05	NA	NA	NA	NA
p_Firmicutes;c_Bacilli;o_Turicibacterales;f_Turicibacteraceae	0.012	5.130E-10	NA	NA	NA	NA
p_Firmicutes;c_Clostridia;o_Clostridiales;f_Christensenellaceae	0.009	1.130E-04	-0.225	1.130E-01	NA	NA
p_Firmicutes;c_Clostridia;o_Clostridiales;f_Clostridiaceae	0.005	1.270E-05	-0.210	2.590E-03	NA	NA
p_Firmicutes;c_Clostridia;o_Clostridiales;f_Lachnospiraceae	NA	NA	0.110	1.510E-02	NA	NA
p_Firmicutes;c_Clostridia;o_Clostridiales;f_Peptococcaceae	-0.009	2.680E-06	0.281	1.630E-02	0.141	2.070E-02
p_Firmicutes;c_Clostridia;o_Clostridiales;f_Peptostreptococcaceae	0.015	5.510E-16	NA	NA	NA	NA
p_Firmicutes;c_Clostridia;o_Clostridiales;f_Ruminococcaceae	NA	NA	NA	NA	NA	NA
p_Firmicutes;c_Clostridia;o_Clostridiales;f_Veillonellaceae	-0.003	1.250E-04	NA	NA	NA	NA
p_Firmicutes;c_Clostridia;o_Clostridiales;f_[Mogibacteriaceae]	0.008	1.090E-09	NA	NA	-0.078	5.190E-02
p_Firmicutes;c_Erysipelotrichi;o_Erysipelotrichales;f_Erysipelotrichaceae	0.003	4.310E-02	0.242	2.170E-03	NA	NA
p_Proteobacteria;c_Betaproteobacteria;o_Burkholderiales;f_Alcaligenaceae	-0.004	1.053E-02	0.248	9.580E-03	-0.077	1.195E-01
p_Proteobacteria;c_Deltaproteobacteria;o_Desulfovibrionales;f_Desulfovibrionaceae	NA	NA	NA	NA	NA	NA
p_Proteobacteria;c_Epsilonproteobacteria;o_Campylobacterales;f_Campylobacteraceae	0.007	3.987E-02	-0.559	4.970E-03	-0.176	8.883E-02
p_Proteobacteria;c_Gammaproteobacteria;o_Enterobacteriales;f_Enterobacteriaceae	NA	NA	-0.375	1.710E-02	NA	NA
p_Proteobacteria;c_Gammaproteobacteria;o_Pasteurellales;f_Pasteurellaceae	NA	NA	NA	NA	NA	NA
p_Tenericutes;c_Mollicutes;o_Anaeroplasmatales;f_Anaeroplasmataceae	0.020	1.000E-14	-0.396	7.670E-03	-0.202	9.000E-03
p_Verrucomicrobia;c_Verruco-5;o_WCHB1-41;f_RFP12	0.009	1.700E-05	NA	NA	NA	NA
p_Bacteroidetes;c_Bacteroidia;o_Bacteroidales;f_Bacteroidaceae;g_Bacteroides	-0.016	1.270E-14	0.723	2.940E-09	NA	NA
p_Bacteroidetes;c_Bacteroidia;o_Bacteroidales;f_Prevotellaceae;g_Prevotella	-0.018	7.350E-10	0.533	2.700E-03	NA	NA
p_Bacteroidetes;c_Bacteroidia;o_Bacteroidales;f_[Odoribacteraceae];g_Odoribacter	-0.018	2.890E-13	NA	NA	NA	NA
p_Bacteroidetes;c_Bacteroidia;o_Bacteroidales;f_[Paraprevotellaceae];g_CF231	0.007	1.910E-02	NA	NA	0.210	2.550E-02
p_Bacteroidetes;c_Bacteroidia;o_Bacteroidales;f_[Paraprevotellaceae];g_[Prevotella]	-0.017	4.530E-07	0.390	5.729E-02	0.329	2.290E-03
p_Firmicutes;c_Bacilli;o_Bacillales;f_Bacillaceae;g_Bacillus	0.017	1.130E-15	NA	NA	NA	NA
p_Firmicutes;c_Bacilli;o_Bacillales;f_Planococcaceae;g_Lysinibacillus	0.014	1.700E-08	NA	NA	NA	NA
p_Firmicutes;c_Bacilli;o_Lactobacillales;f_Lactobacillaceae;g_Lactobacillus	NA	NA	NA	NA	NA	NA
p_Firmicutes;c_Bacilli;o_Lactobacillales;f_Streptococcaceae;g_Streptococcus	0.014	2.100E-05	NA	NA	NA	NA
p_Firmicutes;c_Bacilli;o_Turicibacterales;f_Turicibacteraceae;g_Turicibacter	NA	NA	NA	NA	NA	NA
p_Firmicutes;c_Clostridia;o_Clostridiales;f_Clostridiaceae;g_Clostridium	0.006	4.760E-05	-0.200	2.060E-02	NA	NA
p_Firmicutes;c_Clostridia;o_Clostridiales;f_Lachnospiraceae;g_Blautia	-0.014	6.440E-08	0.854	5.480E-08	NA	NA
p_Firmicutes;c_Clostridia;o_Clostridiales;f_Lachnospiraceae;g_Butyrivibrio	0.012	2.840E-16	0.250	2.180E-03	-0.108	1.108E-02
p_Firmicutes;c_Clostridia;o_Clostridiales;f_Lachnospiraceae;g_Coprococcus	NA	NA	0.535	9.620E-10	NA	NA
p_Firmicutes;c_Clostridia;o_Clostridiales;f_Lachnospiraceae;g_Dorea	0.003	2.290E-02	-0.101	1.529E-01	NA	NA
p_Firmicutes;c_Clostridia;o_Clostridiales;f_Lachnospiraceae;g_Roseburia	-0.004	2.030E-02	0.327	2.000E-03	NA	NA
p_Firmicutes;c_Clostridia;o_Clostridiales;f_Peptococcaceae;g_rc4-4	-0.010	3.310E-07	0.322	9.000E-03	0.162	1.160E-02
p_Firmicutes;c_Clostridia;o_Clostridiales;f_Ruminococcaceae;g_Faecalibacterium	-0.024	1.210E-11	1.140	8.770E-08	NA	NA
p_Firmicutes;c_Clostridia;o_Clostridiales;f_Ruminococcaceae;g_Oscillospira	-0.002	6.032E-02	0.232	1.030E-03	0.072	5.112E-02
p_Firmicutes;c_Clostridia;o_Clostridiales;f_Ruminococcaceae;g_Ruminococcus	0.010	8.750E-12	NA	NA	-0.064	1.450E-01
p_Firmicutes;c_Clostridia;o_Clostridiales;f_Veillonellaceae;g_Phascolarctobacterium	-0.004	3.430E-03	NA	NA	0.079	6.456E-02
p_Firmicutes;c_Clostridia;o_Clostridiales;f_[Mogibacteriaceae];g_Mogibacterium	0.009	1.590E-13	0.137	5.800E-02	-0.058	1.220E-01
p_Proteobacteria;c_Betaproteobacteria;o_Burkholderiales;f_Alcaligenaceae;g_Sutterella	-0.014	1.090E-08	0.740	1.450E-06	NA	NA
p_Proteobacteria;c_Epsilonproteobacteria;o_Campylobacterales;f_Campylobacteraceae;g_Campylobacter	0.007	3.683E-02	-0.555	5.290E-03	-0.175	9.027E-02
p_Proteobacteria;c_Gammaproteobacteria;o_Pasteurellales;f_Pasteurellaceae;g_Gallibacterium	NA	NA	NA	NA	NA	NA
p_Bacteroidetes;c_Bacteroidia;o_Bacteroidales;f_Bacteroidaceae;g_Bacteroides;s_fragilis	-0.012	2.570E-05	0.718	4.650E-05	NA	NA
p_Bacteroidetes;c_Bacteroidia;o_Bacteroidales;f_Prevotellaceae;g_Prevotella;s_stercorea	-0.018	2.130E-05	0.889	7.630E-04	NA	NA
p_Firmicutes;c_Bacilli;o_Lactobacillales;f_Lactobacillaceae;g_Lactobacillus;s_reuteri	NA	NA	NA	NA	NA	NA
p_Firmicutes;c_Clostridia;o_Clostridiales;f_Ruminococcaceae;g_Faecalibacterium;s_prausnitzii	-0.024	1.210E-11	1.140	8.770E-08	NA	NA

Notes:

[1]MAB: Multibreed Angus-Brahman

[2]Positive values reflect postive associations with heifers, and negative values reflect positive associations with bulls

[3]NA reflects that this corresponding variable is not included in the model. It is decided based on its large *P* value when this variable is included in the model as well as a larger Akaike information criterion (AIC) value compared to that of the model exluding this variable.
