# Supplementary material for: Host genetic effects upon the early gut microbiota in a bovine model with graduated spectrum of genetic variation
Source: ISME J. 2019 Oct 17;14(1):302–17. doi: 10.1038/s41396-019-0529-2 (PMC6908690; doi:10.1038/s41396-019-0529-2)
Supplement: Supplementary file 6 — Supplementary Table S5. Influences of age, sires' and dams' Brahman proportion, and gender on growth, plasma parameters, and gut microbiota of regrouped MAB1 preweaning calves based on sires' breed co [file 41396_2019_529_MOESM6_ESM.pdf]

Supplementary Table S5. Influences of age, sires' and dams' Brahman proportion, and gender on growth, plasma parameters, and gut microbiota of regrouped MAB[1] preweaning calves based on sires' breed compostion reflected from the multiple linear regression model.

Response variables	Explanatory variables							
	Age in days		Sire Brahman proportion		Dam Brahman proportion		Gender	
	Coefficient	*P* value	Coefficient	*P* value	Coefficient	*P* value	Coefficient[2]	*P* value
Weight gain	1.045	< 2e-16	NA[3]	NA	-17.568	1.864E-02	-7.340	1.230E-03
Glucose	-0.016	1.300E-05	NA	NA	-0.900	2.210E-02	0.217	6.840E-02
Non-esterified fatty acid (NEFA)	NA	NA	NA	NA	NA	NA	NA	NA
Immunoglobulin G1 (IgG1)	NA	NA	-0.833	8.100E-02	NA	NA	NA	NA
Chao 1	8.651	3.090E-06	NA	NA	NA	NA	NA	NA
Shannon	0.018	4.610E-07	NA	NA	NA	NA	NA	NA
p_Actinobacteria	NA	NA	NA	NA	NA	NA	NA	NA
p_Bacteroidetes	-0.003	2.700E-04	NA	NA	NA	NA	0.067	2.697E-02
p_Chloroflexi	0.010	8.870E-05	NA	NA	0.444	1.150E-01	NA	NA
p_Cyanobacteria	-0.004	6.350E-02	NA	NA	-0.384	9.980E-02	NA	NA
p_Firmicutes	0.001	9.220E-02	NA	NA	NA	NA	NA	NA
p_Planctomycetes	0.014	4.700E-06	-0.283	4.560E-02	NA	NA	NA	NA
p_Proteobacteria	NA	NA	NA	NA	NA	NA	NA	NA
p_Tenericutes	0.006	9.040E-03	NA	NA	NA	NA	NA	NA
p_Verrucomicrobia	0.016	1.290E-06	-0.446	4.580E-03	NA	NA	0.208	5.399E-02
p_Actinobacteria;c_Coriobacteriia;o_Coriobacteriales;f_Coriobacteriaceae	-0.003	1.255E-01	0.328	6.860E-04	NA	NA	0.097	1.389E-01
p_Bacteroidetes;c_Bacteroidia;o_Bacteroidales;f_Bacteroidaceae	-0.005	1.310E-06	NA	NA	0.213	7.050E-02	NA	NA
p_Bacteroidetes;c_Bacteroidia;o_Bacteroidales;f_Porphyromonadaceae	NA	NA	NA	NA	NA	NA	NA	NA
p_Bacteroidetes;c_Bacteroidia;o_Bacteroidales;f_Prevotellaceae	-0.020	4.100E-08	0.345	3.790E-02	NA	NA	NA	NA
p_Bacteroidetes;c_Bacteroidia;o_Bacteroidales;f_Rikenellaceae	0.018	3.370E-06	-0.321	7.970E-02	NA	NA	NA	NA
p_Bacteroidetes;c_Bacteroidia;o_Bacteroidales;f_S24-7	-0.006	2.580E-02	0.194	1.061E-01	NA	NA	0.147	7.700E-02
p_Bacteroidetes;c_Bacteroidia;o_Bacteroidales;f_[Barnesiellaceae]	0.006	4.790E-02	NA	NA	NA	NA	0.142	1.556E-01
p_Bacteroidetes;c_Bacteroidia;o_Bacteroidales;f_[Odoribacteraceae]	-0.014	1.110E-05	NA	NA	NA	NA	NA	NA
p_Bacteroidetes;c_Bacteroidia;o_Bacteroidales;f_[Paraprevotellaceae]	-0.009	3.310E-04	0.281	2.400E-02	NA	NA	0.273	1.653E-03
p_Chloroflexi;c_Anaerolineae;o_Anaerolineales;f_Anaerolinaceae	0.010	4.520E-05	NA	NA	0.378	1.550E-01	NA	NA
p_Firmicutes;c_Bacilli;o_Bacillales;f_Bacillaceae	0.016	6.280E-11	NA	NA	NA	NA	NA	NA
p_Firmicutes;c_Bacilli;o_Bacillales;f_Planococcaceae	0.013	3.130E-06	NA	NA	NA	NA	NA	NA
p_Firmicutes;c_Bacilli;o_Lactobacillales;f_Lactobacillaceae	NA	NA	NA	NA	NA	NA	NA	NA
p_Firmicutes;c_Bacilli;o_Lactobacillales;f_Streptococcaceae	0.017	3.310E-05	NA	NA	NA	NA	NA	NA
p_Firmicutes;c_Bacilli;o_Turicibacterales;f_Turicibacteraceae	0.012	4.740E-07	NA	NA	NA	NA	NA	NA
p_Firmicutes;c_Clostridia;o_Clostridiales;f_Christensenellaceae	0.010	2.410E-04	NA	NA	NA	NA	NA	NA
p_Firmicutes;c_Clostridia;o_Clostridiales;f_Clostridiaceae	0.004	3.480E-03	-0.203	5.070E-03	NA	NA	NA	NA
p_Firmicutes;c_Clostridia;o_Clostridiales;f_Lachnospiraceae	NA	NA	NA	NA	NA	NA	NA	NA
p_Firmicutes;c_Clostridia;o_Clostridiales;f_Peptococcaceae	-0.012	2.970E-06	0.233	4.620E-02	NA	NA	0.165	4.100E-02
p_Firmicutes;c_Clostridia;o_Clostridiales;f_Peptostreptococcaceae	0.014	4.530E-10	NA	NA	NA	NA	NA	NA
p_Firmicutes;c_Clostridia;o_Clostridiales;f_Ruminococcaceae	NA	NA	NA	NA	NA	NA	NA	NA
p_Firmicutes;c_Clostridia;o_Clostridiales;f_Veillonellaceae	-0.004	1.020E-03	0.080	1.197E-01	NA	NA	NA	NA
p_Firmicutes;c_Clostridia;o_Clostridiales;f_[Mogibacteriaceae]	0.007	1.120E-04	NA	NA	NA	NA	-0.096	8.514E-02
p_Firmicutes;c_Erysipelotrichi;o_Erysipelotrichales;f_Erysipelotrichaceae	NA	NA	0.170	2.630E-02	NA	NA	NA	NA
p_Planctomycetes;c_Planctomycetia;o_Pirellulales;f_Pirellulaceae	0.015	2.330E-06	-0.265	7.770E-02	NA	NA	NA	NA
p_Proteobacteria;c_Betaproteobacteria;o_Burkholderiales;f_Alcaligenaceae	-0.006	3.870E-03	NA	NA	NA	NA	NA	NA
p_Proteobacteria;c_Deltaproteobacteria;o_Desulfovibrionales;f_Desulfovibrionaceae	NA	NA	NA	NA	NA	NA	NA	NA
p_Proteobacteria;c_Epsilonproteobacteria;o_Campylobacterales;f_Campylobacteraceae	0.010	1.740E-02	-0.351	8.130E-02	NA	NA	NA	NA
p_Proteobacteria;c_Gammaproteobacteria;o_Enterobacteriales;f_Enterobacteriaceae	NA	NA	-0.471	1.870E-03	NA	NA	NA	NA
p_Proteobacteria;c_Gammaproteobacteria;o_Pasteurellales;f_Pasteurellaceae	NA	NA	-0.366	8.630E-02	NA	NA	NA	NA
p_Tenericutes;c_Mollicutes;o_Anaeroplasmatales;f_Anaeroplasmataceae	0.018	1.640E-09	-0.212	1.275E-01	-0.504	1.135E-01	-0.195	4.270E-02
p_Verrucomicrobia;c_Verruco-5;o_WCHB1-41;f_RFP12	0.009	2.680E-04	NA	NA	NA	NA	0.131	1.223E-01
p_Bacteroidetes;c_Bacteroidia;o_Bacteroidales;f_Bacteroidaceae;g_Bacteroides	-0.017	5.740E-11	0.320	5.100E-03	0.701	7.460E-03	NA	NA
p_Bacteroidetes;c_Bacteroidia;o_Bacteroidales;f_Porphyromonadaceae;g_Parabacteroides	-0.024	1.210E-09	0.533	3.890E-03	0.667	1.112E-01	NA	NA
p_Bacteroidetes;c_Bacteroidia;o_Bacteroidales;f_Prevotellaceae;g_Prevotella	-0.020	4.570E-08	0.335	4.550E-02	NA	NA	NA	NA
p_Bacteroidetes;c_Bacteroidia;o_Bacteroidales;f_[Odoribacteraceae];g_Odoribacter	-0.017	2.210E-07	NA	NA	NA	NA	NA	NA
p_Bacteroidetes;c_Bacteroidia;o_Bacteroidales;f_[Paraprevotellaceae];g_CF231	0.008	2.240E-02	NA	NA	NA	NA	0.214	6.700E-02
p_Bacteroidetes;c_Bacteroidia;o_Bacteroidales;f_[Paraprevotellaceae];g_[Prevotella]	-0.016	1.580E-04	0.471	2.212E-02	NA	NA	0.356	1.252E-02
p_Chloroflexi;c_Anaerolineae;o_Anaerolineales;f_Anaerolinaceae;g_SHD-231	0.009	1.320E-04	NA	NA	NA	NA	NA	NA
p_Firmicutes;c_Bacilli;o_Bacillales;f_Bacillaceae;g_Bacillus	0.016	1.070E-09	NA	NA	NA	NA	NA	NA
p_Firmicutes;c_Bacilli;o_Bacillales;f_Planococcaceae;g_Lysinibacillus	0.012	4.100E-05	NA	NA	NA	NA	NA	NA
p_Firmicutes;c_Bacilli;o_Lactobacillales;f_Lactobacillaceae;g_Lactobacillus	NA	NA	NA	NA	NA	NA	NA	NA
p_Firmicutes;c_Bacilli;o_Lactobacillales;f_Streptococcaceae;g_Streptococcus	0.017	3.340E-05	NA	NA	NA	NA	NA	NA
p_Firmicutes;c_Bacilli;o_Turicibacterales;f_Turicibacteraceae;g_Turicibacter	0.012	4.740E-07	NA	NA	NA	NA	NA	NA
p_Firmicutes;c_Clostridia;o_Clostridiales;f_Clostridiaceae;g_Clostridium	0.006	3.010E-03	-0.233	1.080E-02	NA	NA	NA	NA
p_Firmicutes;c_Clostridia;o_Clostridiales;f_Lachnospiraceae;g_Blautia	-0.013	2.530E-05	0.652	1.990E-05	NA	NA	NA	NA
p_Firmicutes;c_Clostridia;o_Clostridiales;f_Lachnospiraceae;g_Butyrivibrio	0.011	4.510E-11	0.200	7.810E-03	NA	NA	-0.151	3.760E-03
p_Firmicutes;c_Clostridia;o_Clostridiales;f_Lachnospiraceae;g_Coprococcus	NA	NA	0.420	6.010E-07	NA	NA	NA	NA
p_Firmicutes;c_Clostridia;o_Clostridiales;f_Lachnospiraceae;g_Dorea	NA	NA	NA	NA	NA	NA	NA	NA
p_Firmicutes;c_Clostridia;o_Clostridiales;f_Lachnospiraceae;g_Roseburia	-0.004	8.448E-02	0.290	4.640E-03	NA	NA	NA	NA
p_Firmicutes;c_Clostridia;o_Clostridiales;f_Lachnospiraceae;g_[Ruminococcus]	-0.017	3.740E-06	0.587	7.340E-04	NA	NA	NA	NA
p_Firmicutes;c_Clostridia;o_Clostridiales;f_Peptococcaceae;g_rc4-4	-0.013	9.010E-07	0.250	4.320E-02	NA	NA	0.194	2.350E-02
p_Firmicutes;c_Clostridia;o_Clostridiales;f_Ruminococcaceae;g_Faecalibacterium	-0.024	4.500E-08	0.753	1.910E-04	NA	NA	NA	NA
p_Firmicutes;c_Clostridia;o_Clostridiales;f_Ruminococcaceae;g_Oscillospira	-0.003	5.490E-02	0.127	8.650E-02	NA	NA	0.079	1.224E-01
p_Firmicutes;c_Clostridia;o_Clostridiales;f_Ruminococcaceae;g_Ruminococcus	0.009	2.940E-07	NA	NA	-0.361	5.830E-02	NA	NA
p_Firmicutes;c_Clostridia;o_Clostridiales;f_Veillonellaceae;g_Phascolarctobacterium	-0.005	4.390E-03	NA	NA	0.356	7.896E-02	0.087	1.522E-01
p_Firmicutes;c_Clostridia;o_Clostridiales;f_Veillonellaceae;g_Succiniclasticum	NA	NA	NA	NA	NA	NA	NA	NA
p_Firmicutes;c_Clostridia;o_Clostridiales;f_[Mogibacteriaceae];g_Mogibacterium	0.008	2.030E-07	NA	NA	NA	NA	-0.080	8.360E-02
p_Firmicutes;c_Erysipelotrichi;o_Erysipelotrichales;f_Erysipelotrichaceae;g_[Eubacterium]	NA	NA	NA	NA	NA	NA	NA	NA
p_Proteobacteria;c_Betaproteobacteria;o_Burkholderiales;f_Alcaligenaceae;g_Sutterella	-0.015	2.490E-06	0.460	1.600E-03	NA	NA	NA	NA
p_Proteobacteria;c_Epsilonproteobacteria;o_Campylobacterales;f_Campylobacteraceae;g_Campylobacter	0.010	1.700E-02	-0.351	8.160E-02	NA	NA	NA	NA
p_Proteobacteria;c_Gammaproteobacteria;o_Pasteurellales;f_Pasteurellaceae;g_Gallibacterium	NA	NA	NA	NA	NA	NA	NA	NA
p_Bacteroidetes;c_Bacteroidia;o_Bacteroidales;f_Bacteroidaceae;g_Bacteroides;s_coprophilus	-0.033	6.240E-10	0.724	2.700E-03	0.895	1.018E-01	NA	NA
p_Bacteroidetes;c_Bacteroidia;o_Bacteroidales;f_Bacteroidaceae;g_Bacteroides;s_fragilis	-0.011	2.560E-03	0.299	9.468E-02	0.590	1.485E-01	-0.189	1.259E-01
p_Bacteroidetes;c_Bacteroidia;o_Bacteroidales;f_Prevotellaceae;g_Prevotella;s_stercorea	-0.019	2.120E-04	0.872	4.350E-04	NA	NA	NA	NA
p_Firmicutes;c_Bacilli;o_Lactobacillales;f_Lactobacillaceae;g_Lactobacillus;s_reuteri	NA	NA	NA	NA	NA	NA	NA	NA
p_Firmicutes;c_Clostridia;o_Clostridiales;f_Lachnospiraceae;g_Blautia;s_producta	-0.018	5.940E-06	0.845	1.020E-05	NA	NA	NA	NA
p_Firmicutes;c_Clostridia;o_Clostridiales;f_Ruminococcaceae;g_Faecalibacterium;s_prausnitzii	-0.024	4.500E-08	0.753	1.910E-04	NA	NA	NA	NA

Notes:

[1]MAB: Multibreed Angus-Brahman

[2]Positive values reflect postive associations with heifers, and negative values reflect positive associations with bulls

[3]NA reflects that this corresponding variable is not included in the model. It is decided based on its large *P* value when this variable is included in the model as well as a larger Akaike information criterion (AIC) value compared to that of the model exluding this variable.
