# Supplementary material for: Host genetic effects upon the early gut microbiota in a bovine model with graduated spectrum of genetic variation
Source: ISME J. 2019 Oct 17;14(1):302–17. doi: 10.1038/s41396-019-0529-2 (PMC6908690; doi:10.1038/s41396-019-0529-2)
Supplement: Supplementary file 7 — Supplementary Table S6. Influences of age, sires' and dams' Brahman proportion, and gender on growth, plasma parameters, and gut microbiota of regrouped MAB1 preweaning calves based on dams' breed com [file 41396_2019_529_MOESM7_ESM.pdf]

Supplementary Table S6. Influences of age, sires' and dams' Brahman proportion, and gender on growth, plasma parameters, and gut microbiota of regrouped MAB[1] preweaning calves based on dams' breed compostion reflected from the multiple linear regression model.

Response variables	Explanatory variables							
	Age in days		Sire Brahman proportion		Dam Brahman proportion		Gender	
	Coefficient	*P* value	Coefficient	*P* value	Coefficient	*P* value	Coefficient[2]	*P* value
Weight gain	0.953	< 2e-16	NA[3]	NA	-22.803	2.000E-04	-9.743	1.780E-04
Glucose	-0.011	1.570E-02	NA	NA	NA	NA	0.343	2.160E-02
Non-esterified fatty acid (NEFA)	NA	NA	-0.122	1.010E-01	NA	NA	0.055	7.240E-03
Immunoglobulin G1 (IgG1)	NA	NA	NA	NA	NA	NA	NA	NA
Chao 1	6.004	5.950E-03	NA	NA	NA	NA	NA	NA
Shannon	0.019	5.130E-06	NA	NA	NA	NA	NA	NA
p_Actinobacteria	NA	NA	NA	NA	NA	NA	NA	NA
p_Bacteroidetes	-0.003	1.030E-02	NA	NA	NA	NA	0.083	3.620E-02
p_Chloroflexi	0.008	4.990E-03	NA	NA	NA	NA	NA	NA
p_Cyanobacteria	-0.005	1.720E-02	NA	NA	NA	NA	0.130	6.880E-02
p_Firmicutes	0.002	8.350E-03	NA	NA	NA	NA	NA	NA
p_Planctomycetes	0.012	7.880E-04	NA	NA	NA	NA	NA	NA
p_Proteobacteria	NA	NA	NA	NA	NA	NA	NA	NA
p_Tenericutes	0.005	3.180E-02	NA	NA	NA	NA	NA	NA
p_Verrucomicrobia	0.015	1.270E-04	NA	NA	NA	NA	NA	NA
p_Actinobacteria;c_Actinobacteria;o_Actinomycetales;f_Actinomycetaceae	NA	NA	NA	NA	NA	NA	NA	NA
p_Actinobacteria;c_Coriobacteriia;o_Coriobacteriales;f_Coriobacteriaceae	NA	NA	NA	NA	NA	NA	0.133	9.730E-02
p_Bacteroidetes;c_Bacteroidia;o_Bacteroidales;f_Bacteroidaceae	-0.004	3.280E-03	NA	NA	0.183	8.725E-02	NA	NA
p_Bacteroidetes;c_Bacteroidia;o_Bacteroidales;f_Porphyromonadaceae	NA	NA	-0.636	1.250E-01	NA	NA	0.180	1.280E-01
p_Bacteroidetes;c_Bacteroidia;o_Bacteroidales;f_Prevotellaceae	-0.019	8.440E-06	0.941	5.070E-02	-1.432	1.552E-01	NA	NA
p_Bacteroidetes;c_Bacteroidia;o_Bacteroidales;f_Rikenellaceae	0.015	2.100E-04	NA	NA	NA	NA	NA	NA
p_Bacteroidetes;c_Bacteroidia;o_Bacteroidales;f_S24-7	NA	NA	0.640	6.130E-02	NA	NA	0.205	3.530E-02
p_Bacteroidetes;c_Bacteroidia;o_Bacteroidales;f_[Barnesiellaceae]	0.006	8.067E-02	NA	NA	NA	NA	0.334	5.590E-03
p_Bacteroidetes;c_Bacteroidia;o_Bacteroidales;f_[Odoribacteraceae]	-0.011	2.730E-03	1.310	1.740E-03	0.402	1.294E-01	0.190	9.172E-02
p_Bacteroidetes;c_Bacteroidia;o_Bacteroidales;f_[Paraprevotellaceae]	-0.008	1.615E-02	0.759	4.638E-02	-0.545	2.711E-02	0.362	6.710E-04
p_Chloroflexi;c_Anaerolineae;o_Anaerolineales;f_Anaerolinaceae	0.012	5.570E-05	NA	NA	0.339	1.240E-01	NA	NA
p_Firmicutes;c_Bacilli;o_Bacillales;f_Bacillaceae	0.018	4.390E-10	NA	NA	NA	NA	NA	NA
p_Firmicutes;c_Bacilli;o_Bacillales;f_Planococcaceae	0.018	4.350E-07	0.651	9.750E-02	NA	NA	NA	NA
p_Firmicutes;c_Bacilli;o_Lactobacillales;f_Lactobacillaceae	NA	NA	NA	NA	NA	NA	NA	NA
p_Firmicutes;c_Bacilli;o_Lactobacillales;f_Streptococcaceae	0.013	6.800E-03	-1.047	6.390E-02	-0.538	1.402E-01	NA	NA
p_Firmicutes;c_Bacilli;o_Turicibacterales;f_Turicibacteraceae	0.014	2.740E-07	NA	NA	NA	NA	NA	NA
p_Firmicutes;c_Clostridia;o_Clostridiales;f_Christensenellaceae	0.008	2.450E-02	NA	NA	NA	NA	NA	NA
p_Firmicutes;c_Clostridia;o_Clostridiales;f_Clostridiaceae	NA	NA	-0.557	2.030E-03	NA	NA	-0.130	1.073E-02
p_Firmicutes;c_Clostridia;o_Clostridiales;f_Lachnospiraceae	0.002	1.366E-01	NA	NA	0.195	4.220E-02	NA	NA
p_Firmicutes;c_Clostridia;o_Clostridiales;f_Peptococcaceae	-0.008	4.490E-03	NA	NA	0.302	1.581E-01	0.251	6.260E-03
p_Firmicutes;c_Clostridia;o_Clostridiales;f_Peptostreptococcaceae	0.017	1.480E-09	NA	NA	NA	NA	NA	NA
p_Firmicutes;c_Clostridia;o_Clostridiales;f_Ruminococcaceae	0.002	7.560E-02	NA	NA	NA	NA	NA	NA
p_Firmicutes;c_Clostridia;o_Clostridiales;f_Veillonellaceae	-0.004	1.310E-03	NA	NA	NA	NA	NA	NA
p_Firmicutes;c_Clostridia;o_Clostridiales;f_[Mogibacteriaceae]	0.006	1.910E-03	-0.407	7.484E-02	NA	NA	-0.110	7.945E-02
p_Firmicutes;c_Erysipelotrichi;o_Erysipelotrichales;f_Erysipelotrichaceae	0.003	8.730E-02	NA	NA	NA	NA	NA	NA
p_Planctomycetes;c_Planctomycetia;o_Pirellulales;f_Pirellulaceae	0.013	5.660E-04	NA	NA	NA	NA	NA	NA
p_Proteobacteria;c_Betaproteobacteria;o_Burkholderiales;f_Alcaligenaceae	NA	NA	NA	NA	NA	NA	NA	NA
p_Proteobacteria;c_Deltaproteobacteria;o_Desulfovibrionales;f_Desulfovibrionaceae	NA	NA	NA	NA	NA	NA	NA	NA
p_Proteobacteria;c_Epsilonproteobacteria;o_Campylobacterales;f_Campylobacteraceae	NA	NA	NA	NA	NA	NA	NA	NA
p_Proteobacteria;c_Gammaproteobacteria;o_Enterobacteriales;f_Enterobacteriaceae	NA	NA	NA	NA	NA	NA	NA	NA
p_Proteobacteria;c_Gammaproteobacteria;o_Pasteurellales;f_Pasteurellaceae	NA	NA	NA	NA	NA	NA	NA	NA
p_Tenericutes;c_Mollicutes;o_Anaeroplasmatales;f_Anaeroplasmataceae	0.020	6.750E-09	NA	NA	NA	NA	NA	NA
p_Verrucomicrobia;c_Verruco-5;o_WCHB1-41;f_RFP12	0.011	4.830E-04	NA	NA	NA	NA	NA	NA
p_Actinobacteria;c_Coriobacteriia;o_Coriobacteriales;f_Coriobacteriaceae;g_Slackia	NA	NA	NA	NA	NA	NA	0.191	5.270E-02
p_Bacteroidetes;c_Bacteroidia;o_Bacteroidales;f_Bacteroidaceae;g_5-7N15	0.027	4.690E-05	NA	NA	NA	NA	NA	NA
p_Bacteroidetes;c_Bacteroidia;o_Bacteroidales;f_Bacteroidaceae;g_Bacteroides	-0.015	7.170E-07	NA	NA	NA	NA	NA	NA
p_Bacteroidetes;c_Bacteroidia;o_Bacteroidales;f_Porphyromonadaceae;g_Parabacteroides	-0.022	2.630E-06	NA	NA	NA	NA	NA	NA
p_Bacteroidetes;c_Bacteroidia;o_Bacteroidales;f_Prevotellaceae;g_Prevotella	-0.019	1.250E-05	0.952	4.930E-02	NA	NA	NA	NA
p_Bacteroidetes;c_Bacteroidia;o_Bacteroidales;f_[Odoribacteraceae];g_Odoribacter	-0.014	2.590E-04	0.977	2.331E-02	NA	NA	0.187	1.118E-01
p_Bacteroidetes;c_Bacteroidia;o_Bacteroidales;f_[Paraprevotellaceae];g_CF231	NA	NA	NA	NA	NA	NA	0.267	6.300E-02
p_Bacteroidetes;c_Bacteroidia;o_Bacteroidales;f_[Paraprevotellaceae];g_[Prevotella]	-0.011	1.011E-02	1.445	4.860E-03	NA	NA	0.474	8.310E-04
p_Chloroflexi;c_Anaerolineae;o_Anaerolineales;f_Anaerolinaceae;g_SHD-231	0.012	8.970E-06	NA	NA	0.393	6.360E-02	NA	NA
p_Firmicutes;c_Bacilli;o_Bacillales;f_Bacillaceae;g_Bacillus	0.019	2.290E-09	0.526	1.190E-01	NA	NA	NA	NA
p_Firmicutes;c_Bacilli;o_Bacillales;f_Planococcaceae;g_Lysinibacillus	0.017	5.480E-06	0.690	9.630E-02	NA	NA	NA	NA
p_Firmicutes;c_Bacilli;o_Lactobacillales;f_Lactobacillaceae;g_Lactobacillus	NA	NA	NA	NA	NA	NA	NA	NA
p_Firmicutes;c_Bacilli;o_Lactobacillales;f_Streptococcaceae;g_Streptococcus	0.013	6.790E-03	-1.052	6.241E-02	-0.539	1.393E-01	NA	NA
p_Firmicutes;c_Bacilli;o_Turicibacterales;f_Turicibacteraceae;g_Turicibacter	0.014	2.740E-07	NA	NA	NA	NA	NA	NA
p_Firmicutes;c_Clostridia;o_Clostridiales;f_Clostridiaceae;g_Clostridium	0.003	8.000E-02	-0.386	9.640E-02	NA	NA	-0.106	9.540E-02
p_Firmicutes;c_Clostridia;o_Clostridiales;f_Lachnospiraceae;g_Blautia	-0.009	1.120E-02	0.703	8.970E-02	-0.491	6.740E-02	NA	NA
p_Firmicutes;c_Clostridia;o_Clostridiales;f_Lachnospiraceae;g_Butyrivibrio	0.010	5.370E-07	NA	NA	NA	NA	-0.147	2.300E-02
p_Firmicutes;c_Clostridia;o_Clostridiales;f_Lachnospiraceae;g_Coprococcus	NA	NA	NA	NA	NA	NA	0.107	7.090E-02
p_Firmicutes;c_Clostridia;o_Clostridiales;f_Lachnospiraceae;g_Dorea	0.003	5.980E-02	NA	NA	NA	NA	NA	NA
p_Firmicutes;c_Clostridia;o_Clostridiales;f_Lachnospiraceae;g_Roseburia	-0.005	5.592E-02	NA	NA	0.540	5.930E-03	NA	NA
p_Firmicutes;c_Clostridia;o_Clostridiales;f_Lachnospiraceae;g_[Ruminococcus]	-0.016	5.320E-05	NA	NA	NA	NA	NA	NA
p_Firmicutes;c_Clostridia;o_Clostridiales;f_Peptococcaceae;g_rc4-4	-0.009	1.660E-03	NA	NA	NA	NA	0.267	7.340E-03
p_Firmicutes;c_Clostridia;o_Clostridiales;f_Ruminococcaceae;g_Faecalibacterium	-0.022	2.750E-06	NA	NA	NA	NA	NA	NA
p_Firmicutes;c_Clostridia;o_Clostridiales;f_Ruminococcaceae;g_Oscillospira	NA	NA	NA	NA	NA	NA	0.124	5.020E-02
p_Firmicutes;c_Clostridia;o_Clostridiales;f_Ruminococcaceae;g_Ruminococcus	0.010	3.810E-06	NA	NA	-0.252	1.120E-01	-0.097	1.490E-01
p_Firmicutes;c_Clostridia;o_Clostridiales;f_Veillonellaceae;g_Phascolarctobacterium	-0.006	1.420E-02	NA	NA	0.301	1.088E-01	0.138	8.270E-02
p_Firmicutes;c_Clostridia;o_Clostridiales;f_Veillonellaceae;g_Succiniclasticum	NA	NA	NA	NA	NA	NA	NA	NA
p_Firmicutes;c_Clostridia;o_Clostridiales;f_[Mogibacteriaceae];g_Mogibacterium	0.010	1.090E-07	NA	NA	0.306	3.090E-02	NA	NA
p_Firmicutes;c_Erysipelotrichi;o_Erysipelotrichales;f_Erysipelotrichaceae;g_Coprobacillus	0.006	3.420E-02	NA	NA	NA	NA	NA	NA
p_Firmicutes;c_Erysipelotrichi;o_Erysipelotrichales;f_Erysipelotrichaceae;g_[Eubacterium]	NA	NA	NA	NA	NA	NA	NA	NA
p_Proteobacteria;c_Betaproteobacteria;o_Burkholderiales;f_Alcaligenaceae;g_Sutterella	-0.010	6.957E-03	0.649	1.478E-01	NA	NA	NA	NA
p_Proteobacteria;c_Epsilonproteobacteria;o_Campylobacterales;f_Campylobacteraceae;g_Campylobacter	NA	NA	NA	NA	NA	NA	NA	NA
p_Proteobacteria;c_Gammaproteobacteria;o_Pasteurellales;f_Pasteurellaceae;g_Gallibacterium	NA	NA	NA	NA	NA	NA	NA	NA
p_Bacteroidetes;c_Bacteroidia;o_Bacteroidales;f_Bacteroidaceae;g_Bacteroides;s_coprophilus	-0.024	2.700E-04	1.856	1.403E-02	NA	NA	0.397	5.417E-02
p_Bacteroidetes;c_Bacteroidia;o_Bacteroidales;f_Bacteroidaceae;g_Bacteroides;s_fragilis	-0.016	2.440E-04	NA	NA	NA	NA	NA	NA
p_Bacteroidetes;c_Bacteroidia;o_Bacteroidales;f_Prevotellaceae;g_Prevotella;s_stercorea	-0.017	5.950E-03	1.518	3.597E-02	NA	NA	NA	NA
p_Firmicutes;c_Bacilli;o_Lactobacillales;f_Lactobacillaceae;g_Lactobacillus;s_reuteri	NA	NA	NA	NA	NA	NA	NA	NA
p_Firmicutes;c_Clostridia;o_Clostridiales;f_Lachnospiraceae;g_Blautia;s_producta	-0.014	4.210E-03	1.206	3.131E-02	NA	NA	NA	NA
p_Firmicutes;c_Clostridia;o_Clostridiales;f_Ruminococcaceae;g_Faecalibacterium;s_prausnitzii	-0.022	2.750E-06	NA	NA	NA	NA	NA	NA

Notes:

[1]MAB: Multibreed Angus-Brahman

[2]Positive values reflect postive associations with heifers, and negative values reflect positive associations with bulls

[3]NA reflects that this corresponding variable is not included in the model. It is decided based on its large *P* value when this variable is included in the model as well as a larger Akaike information criterion (AIC) value compared to that of the model exluding this variable.
