# Supplementary material for: Host genetic effects upon the early gut microbiota in a bovine model with graduated spectrum of genetic variation
Source: ISME J. 2019 Oct 17;14(1):302–17. doi: 10.1038/s41396-019-0529-2 (PMC6908690; doi:10.1038/s41396-019-0529-2)
Supplement: Supplementary file 8 — Supplementary Table S7. Correlation between Brahman proportion and genotypes of SNPs that are located in or near mucin-encoding genes [file 41396_2019_529_MOESM8_ESM.pdf]

Supplementary Table S7. Correlation between Brahman proportion and genotypes of SNPs that are located in or near mucin-encoding genes.

SNP name	Chromosome	Position	Gene	Inside the gene?	Spearman correlation coefficient	P_{raw} value	P_{adjust} value
1-69942606-T-C-rs378114794	1	69942606	MUC13	Yes,_Inside_Gene	-0.515	1.204E-16	1.440E-15
1-69942646-C-T-rs381423382	1	69942646	MUC13	Yes,_Inside_Gene	0.347	1.084E-07	3.482E-07
1-69945680-C-T-rs522080259	1	69945680	MUC13	Yes,_Inside_Gene	0.341	1.600E-07	5.082E-07
1-69945697-G-T-rs381772189	1	69945697	MUC13	Yes,_Inside_Gene	-0.118	7.780E-02	1.025E-01
1-69945704-T-C-rs384651654	1	69945704	MUC13	Yes,_Inside_Gene	-0.166	1.254E-02	1.868E-02
1-69945834-G-A-rs379833914	1	69945834	MUC13	Yes,_Inside_Gene	0.662	9.607E-30	6.919E-28
1-69958891-A-G-rs134555951	1	69958891	MUC13	Yes,_Inside_Gene	0.530	1.512E-17	2.330E-16
1-69958904-T-G-rs452169488	1	69958904	MUC13	Yes,_Inside_Gene	0.239	3.145E-04	5.856E-04
1-69958915-G-A-rs135266185	1	69958915	MUC13	Yes,_Inside_Gene	-0.256	1.132E-04	2.204E-04
1-69958919-T-C-rs136441203	1	69958919	MUC13	Yes,_Inside_Gene	0.204	2.823E-03	4.550E-03
1-69959029-C-T-rs456600677	1	69959029	MUC13	Yes,_Inside_Gene	0.108	1.074E-01	1.389E-01
1-69959045-T-C-rs481124020	1	69959045	MUC13	Yes,_Inside_Gene	-0.419	1.263E-10	7.162E-10
1-69959576-C-T-rs385153041	1	69959576	MUC13	Marker_is_<=_2500_bp_After_Feature	0.167	1.227E-02	1.840E-02
1-69961838-T-G-rs209870119	1	69961838	MUC13	Marker_is_>_2500_bp_<=5000_bp_After_Feature	-0.327	6.082E-07	1.706E-06
1-69961865-A-T-rs208482355	1	69961865	MUC13	Marker_is_>_2500_bp_<=5000_bp_After_Feature	0.329	4.768E-07	1.374E-06
1-71078283-A-T-rs520894556	1	71078283	MUC20	Yes,_Inside_Gene	-0.134	4.779E-02	6.575E-02
1-71078456-G-A-rs209863266	1	71078456	MUC20	Yes,_Inside_Gene	-0.190	4.211E-03	6.640E-03
1-71078502-A-G-rs210543483	1	71078502	MUC20	Yes,_Inside_Gene	-0.236	3.549E-04	6.496E-04
1-71080055-C-G-rs210304339	1	71080055	MUC20	Yes,_Inside_Gene	-0.335	6.181E-07	1.711E-06
1-71085391-G-T-rs208323556	1	71085391	MUC20	Yes,_Inside_Gene	0.516	1.797E-16	2.046E-15
1-71096129-C-T-rs452308189	1	71096129	MUC4	Yes,_Inside_Gene	0.293	9.444E-06	2.138E-05
1-71099068-C-T-rs516084023	1	71099068	MUC4	Yes,_Inside_Gene	-0.172	1.183E-02	1.787E-02
1-71099103-T-G-rs458620462	1	71099103	MUC4	Yes,_Inside_Gene	-0.331	3.917E-07	1.144E-06
1-71101197-C-T-rs435334313	1	71101197	MUC4	Yes,_Inside_Gene	0.320	1.457E-06	3.710E-06
1-71102370-G-T-rs133257965	1	71102370	MUC4	Yes,_Inside_Gene	0.148	2.659E-02	3.829E-02
1-71104148-C-T-rs208812969	1	71104148	MUC4	Yes,_Inside_Gene	-0.526	2.436E-17	3.514E-16
1-71105876-T-C-rs109704859	1	71105876	MUC4	Yes,_Inside_Gene	0.019	7.819E-01	8.042E-01
1-71107208-G-A-rs210161567	1	71107208	MUC4	Yes,_Inside_Gene	-0.401	4.846E-10	2.494E-09
1-71107253-G-T-rs133681725	1	71107253	MUC4	Yes,_Inside_Gene	0.256	1.002E-04	2.004E-04
1-71112140-G-A-rs384284796	1	71112140	MUC4	Yes,_Inside_Gene	0.260	7.899E-05	1.595E-04
1-71114449-T-C-rs210156914	1	71114449	MUC4	Yes,_Inside_Gene	-0.229	5.842E-04	1.018E-03
1-71116493-C-T-rs463288132	1	71116493	MUC4	Yes,_Inside_Gene	0.088	1.902E-01	2.334E-01
1-71117959-G-A-rs110703404	1	71117959	MUC4	Yes,_Inside_Gene	0.064	3.417E-01	3.864E-01
1-71117981-T-C-rs379515375	1	71117981	MUC4	Yes,_Inside_Gene	-0.403	4.220E-10	2.223E-09
1-71119822-G-T-rs133490274	1	71119822	MUC4	Yes,_Inside_Gene	-0.310	2.092E-06	5.072E-06
1-71121105-G-T-rs209874806	1	71121105	MUC4	Yes,_Inside_Gene	-0.051	4.535E-01	4.923E-01
3-15484489-C-T-rs432755602	3	15484489	MUC1	Yes,_Inside_Gene	-0.055	4.152E-01	4.623E-01
5-40452937-G-T-rs43049932	5	40452937	MUC19	Nearest_feature_is_>_25,000_bp_after_marker	-0.076	2.591E-01	2.993E-01
5-40563598-A-G-rs475040730	5	40563598	MUC19	Yes,_Inside_Gene	-0.112	9.346E-02	1.224E-01
5-40585825-T-C-rs43435100	5	40585825	MUC19	Yes,_Inside_Gene	0.225	6.996E-04	1.209E-03
5-40598970-G-A-rs109902399	5	40598970	MUC19	Yes,_Inside_Gene	-0.085	2.036E-01	2.429E-01
5-40598974-A-C-rs110889423	5	40598974	MUC19	Yes,_Inside_Gene	0.631	4.667E-26	1.681E-24
5-40599195-G-T-rs110499319	5	40599195	MUC19	Yes,_Inside_Gene	0.086	1.982E-01	2.406E-01
5-40599208-A-G-rs110297500	5	40599208	MUC19	Yes,_Inside_Gene	0.230	5.162E-04	9.066E-04
5-40599220-C-A-rs132946965	5	40599220	MUC19	Yes,_Inside_Gene	-0.085	2.036E-01	2.429E-01
5-40599364-G-A-rs109084320	5	40599364	MUC19	Yes,_Inside_Gene	-0.371	1.207E-08	4.752E-08
5-40599418-G-C-rs109969033	5	40599418	MUC19	Yes,_Inside_Gene	0.032	6.370E-01	6.712E-01
5-40599460-G-C-rs110407313	5	40599460	MUC19	Yes,_Inside_Gene	0.051	4.449E-01	4.878E-01
5-40604197-G-T-rs432355789	5	40604197	MUC19	Yes,_Inside_Gene	-0.224	7.426E-04	1.273E-03
5-40604431-G-A-rs455144379	5	40604431	MUC19	Yes,_Inside_Gene	0.316	1.337E-06	3.446E-06
5-40604539-T-G-rs42999193	5	40604539	MUC19	Yes,_Inside_Gene	0.529	3.029E-17	4.091E-16
5-40604569-G-T-rs42999194	5	40604569	MUC19	Yes,_Inside_Gene	-0.077	2.498E-01	2.933E-01
5-40604737-G-T-rs42999197	5	40604737	MUC19	Yes,_Inside_Gene	-0.361	2.384E-08	8.863E-08
5-40618749-G-A-rs383139383	5	40618749	MUC19	Yes,_Inside_Gene	0.506	4.636E-16	4.773E-15
5-40643873-T-C-rs136517752	5	40643873	MUC19	Yes,_Inside_Gene	0.038	5.697E-01	6.062E-01
5-40643965-C-T-rs446954663	5	40643965	MUC19	Yes,_Inside_Gene	0.318	1.174E-06	3.082E-06
5-40653866-C-T-rs383263575	5	40653866	MUC19	Yes,_Inside_Gene	0.333	1.045E-06	2.800E-06
5-40654105-C-T-rs453934939	5	40654105	MUC19	Yes,_Inside_Gene	0.106	1.141E-01	1.467E-01
7-14320716-C-A-rs209024096	7	14320716	MUC16	Yes,_Inside_Gene	0.296	7.457E-06	1.714E-05
7-14323295-G-C-rs209334744	7	14323295	MUC16	Yes,_Inside_Gene	0.425	5.481E-11	3.199E-10
7-14326280-T-C-rs460291013	7	14326280	MUC16	Yes,_Inside_Gene	-0.405	2.690E-10	1.453E-09
7-14328090-T-A-rs209627122	7	14328090	MUC16	Yes,_Inside_Gene	0.031	6.445E-01	6.758E-01
7-14330874-C-T-rs435626914	7	14330874	MUC16	Yes,_Inside_Gene	0.500	1.177E-15	1.071E-14
7-14335319-G-T-rs110563141	7	14335319	MUC16	Yes,_Inside_Gene	-0.238	3.093E-04	5.809E-04
7-14338508-C-T-rs379918306	7	14338508	MUC16	Yes,_Inside_Gene	-0.661	1.393E-29	7.506E-28
7-14343366-C-T-rs209011074	7	14343366	MUC16	Yes,_Inside_Gene	0.174	9.234E-03	1.415E-02
7-14344266-A-C-rs433704502	7	14344266	MUC16	Yes,_Inside_Gene	0.173	9.806E-03	1.492E-02
7-14354974-G-A-rs109735464	7	14354974	MUC16	Yes,_Inside_Gene	0.284	1.667E-05	3.644E-05
7-14364954-G-A-rs208573389	7	14364954	MUC16	Yes,_Inside_Gene	-0.002	9.784E-01	9.784E-01
7-14365027-G-A-rs211153134	7	14365027	MUC16	Yes,_Inside_Gene	0.272	3.593E-05	7.602E-05
7-14367888-T-A-rs381206569	7	14367888	MUC16	Yes,_Inside_Gene	0.340	1.876E-07	5.885E-07
7-14372099-C-T-rs208778554	7	14372099	MUC16	Yes,_Inside_Gene	0.194	3.686E-03	5.890E-03
7-14373458-T-G-rs110614577	7	14373458	MUC16	Yes,_Inside_Gene	0.360	2.860E-08	1.047E-07
7-14384888-G-C-rs208967240	7	14384888	MUC16	Yes,_Inside_Gene	0.319	1.021E-06	2.754E-06
7-14385123-C-G-rs208623006	7	14385123	MUC16	Yes,_Inside_Gene	0.232	4.837E-04	8.564E-04
7-14387039-C-T-rs447008800	7	14387039	MUC16	Yes,_Inside_Gene	-0.097	1.458E-01	1.831E-01
7-14387078-G-A-rs451081431	7	14387078	MUC16	Yes,_Inside_Gene	0.388	1.721E-09	8.256E-09

7-14387390-A-G-rs385478124	7	14387390	MUC16	Yes,_Inside_Gene	0.014	8.377E-01	8.535E-01
7-14387763-T-G-rs462387951	7	14387763	MUC16	Yes,_Inside_Gene	-0.691	3.478E-33	7.517E-31
7-14388834-T-C-rs136758635	7	14388834	MUC16	Yes,_Inside_Gene	0.386	2.059E-09	9.467E-09
7-14388897-C-T-rs134698651	7	14388897	MUC16	Yes,_Inside_Gene	0.267	5.363E-05	1.113E-04
7-14389020-T-G-rs136055907	7	14389020	MUC16	Yes,_Inside_Gene	0.402	6.130E-10	3.079E-09
7-14389127-A-G-rs110948032	7	14389127	MUC16	Yes,_Inside_Gene	-0.255	2.369E-04	4.489E-04
7-14390301-C-T-rs454517095	7	14390301	MUC16	Yes,_Inside_Gene	-0.439	5.175E-12	3.384E-11
7-14390542-C-A-rs210577627	7	14390542	MUC16	Yes,_Inside_Gene	0.132	4.862E-02	6.647E-02
7-14391024-A-G-rs208869147	7	14391024	MUC16	Yes,_Inside_Gene	-0.687	1.052E-32	1.134E-30
7-14405094-C-A-rs109547736	7	14405094	MUC16	Yes,_Inside_Gene	0.086	1.973E-01	2.406E-01
7-14405096-A-G-rs110228721	7	14405096	MUC16	Yes,_Inside_Gene	0.612	2.310E-24	6.237E-23
7-14410427-C-T-rs440589844	7	14410427	MUC16	Yes,_Inside_Gene	-0.081	2.261E-01	2.669E-01
7-14410434-G-C-rs209094158	7	14410434	MUC16	Yes,_Inside_Gene	0.232	4.430E-04	7.975E-04
7-14415699-A-G-rs109586044	7	14415699	MUC16	Yes,_Inside_Gene	-0.462	3.984E-13	2.964E-12
7-14415792-G-A-rs108987183	7	14415792	MUC16	Yes,_Inside_Gene	-0.391	2.321E-09	1.023E-08
7-14416102-A-G-rs109113961	7	14416102	MUC16	Yes,_Inside_Gene	0.157	1.844E-02	2.674E-02
7-14416363-T-G-rs474361208	7	14416363	MUC16	Yes,_Inside_Gene	0.098	1.432E-01	1.809E-01
7-14416701-C-T-rs209163760	7	14416701	MUC16	Yes,_Inside_Gene	0.180	6.910E-03	1.082E-02
7-14417314-T-C-rs525901464	7	14417314	MUC16	Yes,_Inside_Gene	0.658	2.540E-29	1.097E-27
7-14417638-G-A-rs435681981	7	14417638	MUC16	Yes,_Inside_Gene	0.051	4.489E-01	4.897E-01
15-58050659-T-C-rs210576274	15	58050659	MUC15	Yes,_Inside_Gene	0.516	1.932E-16	2.084E-15
15-58050979-A-G-rs451108017	15	58050979	MUC15	Yes,_Inside_Gene	0.505	5.944E-16	5.832E-15
15-58052642-T-C-rs137805979	15	58052642	MUC15	Yes,_Inside_Gene	-0.434	9.813E-12	6.054E-11
15-58057449-C-T-rs133888462	15	58057449	MUC15	Yes,_Inside_Gene	-0.213	1.289E-03	2.175E-03
23-27908924-G-A-rs379911053	23	27908924	MUC21	Yes,_Inside_Gene	-0.554	1.835E-19	3.593E-18
23-27909368-A-C-rs382612899	23	27909368	MUC21	Yes,_Inside_Gene	0.364	1.862E-08	7.048E-08
25-36118604-G-A-rs43725180	25	36118604	MUC12	Yes,_Inside_Gene	0.335	2.933E-07	8.790E-07
25-36119961-A-C-rs43725203	25	36119961	MUC12	Yes,_Inside_Gene	-0.371	9.864E-09	4.096E-08
25-36124376-A-T-rs378007912	25	36124376	MUC12	Yes,_Inside_Gene	0.317	1.206E-06	3.149E-06
25-36124542-G-A-rs43763103	25	36124542	MUC12	Yes,_Inside_Gene	-0.290	1.008E-05	2.249E-05
25-36124568-C-G-rs384256310	25	36124568	MUC12	Yes,_Inside_Gene	-0.030	6.530E-01	6.814E-01
25-36124581-C-A-rs43763105	25	36124581	MUC12	Yes,_Inside_Gene	0.179	6.966E-03	1.083E-02
25-36124674-C-G-rs43764631	25	36124674	MUC12	Yes,_Inside_Gene	0.274	3.159E-05	6.758E-05
25-36124688-G-A-rs43764632	25	36124688	MUC12	Yes,_Inside_Gene	-0.166	1.289E-02	1.906E-02
25-36124806-T-C-rs43764633	25	36124806	MUC12	Yes,_Inside_Gene	-0.386	2.148E-09	9.675E-09
25-36124860-T-C-rs208639614	25	36124860	MUC12	Yes,_Inside_Gene	-0.493	3.553E-15	3.067E-14
25-36124878-C-T-rs207552374	25	36124878	MUC12	Yes,_Inside_Gene	-0.140	3.697E-02	5.153E-02
25-36124923-C-T-rs209938014	25	36124923	MUC12	Yes,_Inside_Gene	-0.046	4.882E-01	5.246E-01
25-36124929-C-T-rs455010069	25	36124929	MUC12	Yes,_Inside_Gene	-0.350	7.213E-08	2.396E-07
25-36125015-A-C-rs447475023	25	36125015	MUC12	Yes,_Inside_Gene	0.019	7.717E-01	7.975E-01
25-36125052-G-A-rs210588156	25	36125052	MUC12	Yes,_Inside_Gene	-0.336	2.639E-07	8.032E-07
25-36125139-C-T-rs379661977	25	36125139	MUC12	Yes,_Inside_Gene	-0.352	6.038E-08	2.039E-07
25-36125142-T-C-rs208394055	25	36125142	MUC12	Yes,_Inside_Gene	-0.357	3.751E-08	1.306E-07
25-36125163-T-C-rs469487306	25	36125163	MUC12	Yes,_Inside_Gene	0.358	3.347E-08	1.186E-07
25-36125218-T-A-rs380152318	25	36125218	MUC12	Yes,_Inside_Gene	0.129	5.271E-02	7.160E-02
25-36125228-G-A-rs436614100	25	36125228	MUC12	Yes,_Inside_Gene	0.043	5.251E-01	5.615E-01
25-36125252-C-G-rs475552619	25	36125252	MUC12	Yes,_Inside_Gene	0.232	4.590E-04	8.193E-04
25-36125292-A-G-rs460471693	25	36125292	MUC12	Yes,_Inside_Gene	0.215	1.177E-03	2.002E-03
25-36125337-C-T-rs380050173	25	36125337	MUC12	Yes,_Inside_Gene	0.387	1.859E-09	8.734E-09
25-36125348-C-A-rs451289443	25	36125348	MUC12	Yes,_Inside_Gene	0.444	3.122E-12	2.106E-11
25-36125426-G-A-rs382796747	25	36125426	MUC12	Yes,_Inside_Gene	-0.369	1.200E-08	4.752E-08
25-36125789-A-G-rs432580800	25	36125789	MUC12	Yes,_Inside_Gene	0.291	9.495E-06	2.138E-05
25-36125813-T-A-rs208442207	25	36125813	MUC12	Yes,_Inside_Gene	-0.347	9.491E-08	3.106E-07
25-36125825-C-T-rs210308856	25	36125825	MUC12	Yes,_Inside_Gene	0.060	3.668E-01	4.106E-01
25-36126068-G-A-rs208831969	25	36126068	MUC12	Yes,_Inside_Gene	-0.013	8.452E-01	8.553E-01
25-36127075-G-A-rs432239059	25	36127075	MUC12	Yes,_Inside_Gene	-0.459	4.168E-13	3.002E-12
25-36127125-T-C-rs440961305	25	36127125	MUC12	Yes,_Inside_Gene	-0.261	7.488E-05	1.526E-04
25-36127133-G-A-rs461491946	25	36127133	MUC12	Yes,_Inside_Gene	-0.296	6.310E-06	1.481E-05
25-36127179-G-A-rs380618318	25	36127179	MUC12	Yes,_Inside_Gene	0.212	1.829E-03	3.016E-03
25-36127963-T-G-rs43763093	25	36127963	MUC12	Yes,_Inside_Gene	0.062	3.627E-01	4.080E-01
25-36127984-G-A-rs210595462	25	36127984	MUC12	Yes,_Inside_Gene	-0.101	1.325E-01	1.683E-01
25-36128054-A-G-rs208401341	25	36128054	MUC12	Yes,_Inside_Gene	0.017	8.039E-01	8.230E-01
25-36128929-C-T-rs210014138	25	36128929	MUC12	Yes,_Inside_Gene	-0.145	3.025E-02	4.298E-02
25-36129062-A-G-rs382384702	25	36129062	MUC12	Yes,_Inside_Gene	0.147	2.940E-02	4.206E-02
25-36129440-T-G-rs471303474	25	36129440	MUC12	Yes,_Inside_Gene	0.209	1.969E-03	3.222E-03
25-36129970-A-C-rs210809242	25	36129970	MUC12	Yes,_Inside_Gene	-0.379	4.429E-09	1.914E-08
25-36130313-T-C-rs450453591	25	36130313	MUC12	Yes,_Inside_Gene	-0.485	1.184E-14	9.803E-14
25-36130596-G-A-rs450822779	25	36130596	MUC12	Yes,_Inside_Gene	0.073	2.760E-01	3.154E-01
25-36130632-A-G-rs210987447	25	36130632	MUC12	Yes,_Inside_Gene	-0.288	1.137E-05	2.513E-05
25-36130650-T-C-rs207765355	25	36130650	MUC12	Yes,_Inside_Gene	-0.256	1.037E-04	2.036E-04
25-36146175-A-C-rs524371728	25	36146175	MUC12	Yes,_Inside_Gene	-0.109	1.018E-01	1.325E-01
25-36146473-G-A-rs522033207	25	36146473	MUC12	Yes,_Inside_Gene	-0.201	2.469E-03	4.010E-03
25-36146649-A-C-rs381166539	25	36146649	MUC12	Yes,_Inside_Gene	0.419	1.684E-10	9.305E-10
25-36146683-T-C-rs207666727	25	36146683	MUC12	Yes,_Inside_Gene	-0.053	4.321E-01	4.786E-01
25-36147807-C-T-rs211277321	25	36147807	MUC12	Yes,_Inside_Gene	-0.428	2.039E-11	1.224E-10
25-36148129-T-C-rs384275373	25	36148129	MUC12	Yes,_Inside_Gene	-0.052	4.426E-01	4.878E-01
25-36166928-G-C-rs433140174	25	36166928	MUC12	Yes,_Inside_Gene	0.264	6.534E-05	1.343E-04
25-36167786-C-T-rs207678154	25	36167786	MUC12	Yes,_Inside_Gene	0.400	6.470E-10	3.176E-09
25-36167802-G-A-rs382870075	25	36167802	MUC12	Yes,_Inside_Gene	-0.212	1.395E-03	2.335E-03
25-36168706-A-G-rs455849525	25	36168706	MUC12	Yes,_Inside_Gene	-0.371	1.036E-08	4.238E-08

25-36168827-A-G-rs469425205	25	36168827	MUC12	Yes,_Inside_Gene	-0.071	2.902E-01	3.299E-01
25-36168911-A-G-rs209852756	25	36168911	MUC12	Yes,_Inside_Gene	-0.126	6.143E-02	8.294E-02
25-36169022-C-A-rs470670444	25	36169022	MUC12	Yes,_Inside_Gene	-0.091	1.721E-01	2.136E-01
25-36174076-T-A-rs471608849	25	36174076	MUC12	Yes,_Inside_Gene	0.256	1.018E-04	2.016E-04
25-36177035-G-T-rs43726625	25	36177035	MUC12	Yes,_Inside_Gene	0.542	1.632E-18	2.934E-17
25-36177161-A-G-rs208101045	25	36177161	MUC12	Yes,_Inside_Gene	0.478	3.984E-14	3.070E-13
25-36264390-A-C-rs43725892	25	36264390	MUC12	Yes,_Inside_Gene	-0.101	1.293E-01	1.653E-01
25-36264843-T-C-rs524961186	25	36264843	MUC12	Yes,_Inside_Gene	0.321	8.399E-07	2.297E-06
25-36266585-C-T-rs211227324	25	36266585	MUC12	Yes,_Inside_Gene	-0.255	1.215E-04	2.343E-04
25-36266591-G-A-rs208665498	25	36266591	MUC12	Yes,_Inside_Gene	-0.123	6.447E-02	8.649E-02
25-36267719-T-G-rs210476511	25	36267719	MUC12	Yes,_Inside_Gene	0.174	9.026E-03	1.393E-02
25-36267882-C-T-rs135614410	25	36267882	MUC12	Yes,_Inside_Gene	-0.047	4.854E-01	5.242E-01
25-36268034-A-G-rs208036323	25	36268034	MUC12	Yes,_Inside_Gene	0.119	7.409E-02	9.879E-02
25-36274534-T-A-rs208879451	25	36274534	MUC12	Yes,_Inside_Gene	0.194	3.708E-03	5.890E-03
25-36274568-C-G-rs210320285	25	36274568	MUC12	Yes,_Inside_Gene	-0.530	1.171E-17	1.944E-16
29-51504286-C-T-rs456327378	29	51504286	MUC2	Marker_is_<=_2500_bp_Before_Feature	0.365	1.773E-08	6.827E-08
