# Supplementary material for: Host genetic effects upon the early gut microbiota in a bovine model with graduated spectrum of genetic variation
Source: ISME J. 2019 Oct 17;14(1):302–17. doi: 10.1038/s41396-019-0529-2 (PMC6908690; doi:10.1038/s41396-019-0529-2)
Supplement: Supplementary file 9 — Supplementary Table S8. Associations between the genotypes of SNPs located in mucin-encoding genes of MAB1 preweaning calves and log10 transformed relative abundance of mucin-degrading bacteria reveal [file 41396_2019_529_MOESM9_ESM.pdf]

Supplementary Table S8. Associations between the genotypes of SNPs located in mucin-encoding genes of MAB[1] preweaning calves and log10 transformed relative abundance of mucin-degrading bacteria revealed from the multiple linear regression model.

Explanatory variables		g_Clostridium							f_Rikenellaceae							g_Akkermansia						
Response variables		Age in days		Brahman proportion		SNP genotype		AIC[2]	Age in days		Brahman proportion		SNP genotype		AIC	Age in days		Brahman proportion		SNP genotype		AIC
SNP name	Gene	Coefficient	*P* value	Coefficient	*P* value	Coefficient	*P* value		Coefficient	*P* value	Coefficient	*P* value	Coefficient	*P* value		Coefficient	*P* value	Coefficient	*P* value	Coefficient	*P* value	
15-58050659-T-C-rs210576274	MUC15	0.006	5.170E-05	-0.197	2.360E-02	NA[3]	NA	155.52	0.019	6.690E-10	-0.497	5.790E-03	NA	NA	480.53	0.016	1.900E-04	-1.217	5.600E-06	NA	NA	652.85
15-58050979-A-G-rs451108017	MUC15	0.006	5.170E-05	-0.197	2.360E-02	NA	NA	155.52	0.019	6.690E-10	-0.497	5.790E-03	NA	NA	480.53	0.016	2.420E-04	-0.981	1.657E-03	-0.167	1.490E-01	652.73
15-58052642-T-C-rs137805979	MUC15	0.006	5.170E-05	-0.197	2.360E-02	NA	NA	155.52	0.019	6.690E-10	-0.497	5.790E-03	NA	NA	480.53	0.015	3.420E-04	-1.632	3.700E-07	-0.271	1.739E-02	649.08
15-58057449-C-T-rs133888462	MUC15	0.006	5.170E-05	-0.197	2.360E-02	NA	NA	155.52	0.019	6.690E-10	-0.497	5.790E-03	NA	NA	480.53	0.016	1.900E-04	-1.217	5.600E-06	NA	NA	652.85
1-69942606-T-C-rs378114794	MUC13	0.006	5.170E-05	-0.197	2.360E-02	NA	NA	155.52	0.019	6.690E-10	-0.497	5.790E-03	NA	NA	480.53	0.016	1.900E-04	-1.217	5.600E-06	NA	NA	652.85
1-69942646-C-T-rs381423382	MUC13	0.006	5.170E-05	-0.197	2.360E-02	NA	NA	155.52	0.019	6.690E-10	-0.497	5.790E-03	NA	NA	480.53	0.016	1.900E-04	-1.217	5.600E-06	NA	NA	652.85
1-69945680-C-T-rs522080259	MUC13	0.006	5.170E-05	-0.197	2.360E-02	NA	NA	155.52	0.019	6.690E-10	-0.497	5.790E-03	NA	NA	480.53	0.016	1.900E-04	-1.217	5.600E-06	NA	NA	652.85
1-69945697-G-T-rs381772189	MUC13	0.006	5.170E-05	-0.197	2.360E-02	NA	NA	155.52	0.018	2.100E-09	-0.551	2.430E-03	-0.181	6.118E-02	478.95	0.015	3.690E-04	-1.282	2.280E-06	-0.218	1.243E-01	652.44
1-69945704-T-C-rs384651654	MUC13	0.006	5.170E-05	-0.197	2.360E-02	NA	NA	155.52	0.019	6.690E-10	-0.497	5.790E-03	NA	NA	480.53	0.016	1.900E-04	-1.217	5.600E-06	NA	NA	652.85
1-69945834-G-A-rs379833914	MUC13	0.006	5.170E-05	-0.197	2.360E-02	NA	NA	155.52	0.019	6.690E-10	-0.497	5.790E-03	NA	NA	480.53	0.016	1.900E-04	-1.217	5.600E-06	NA	NA	652.85
1-69958891-A-G-rs134555951	MUC13	0.006	2.580E-05	NA	NA	-0.088	5.979E-03	150.67	0.019	1.350E-10	NA	NA	-0.228	6.870E-04	473.18	0.016	1.900E-04	-1.217	5.600E-06	NA	NA	652.85
1-69958904-T-G-rs452169488	MUC13	0.006	8.320E-05	-0.248	5.690E-03	0.086	6.655E-02	151.93	0.018	1.140E-09	-0.614	9.350E-04	0.211	2.912E-02	475.02	0.016	1.900E-04	-1.217	5.600E-06	NA	NA	652.85
1-69958915-G-A-rs135266185	MUC13	0.006	5.170E-05	-0.197	2.360E-02	NA	NA	155.52	0.019	6.690E-10	-0.497	5.790E-03	NA	NA	480.53	0.016	1.900E-04	-1.217	5.600E-06	NA	NA	652.85
1-69958919-T-C-rs136441203	MUC13	0.006	5.170E-05	-0.197	2.360E-02	NA	NA	155.52	0.018	2.100E-09	-0.675	4.800E-04	0.190	1.783E-02	446.27	0.016	1.900E-04	-1.217	5.600E-06	NA	NA	652.85
1-69959029-C-T-rs456600677	MUC13	0.006	5.170E-05	-0.197	2.360E-02	NA	NA	155.52	0.019	6.690E-10	-0.497	5.790E-03	NA	NA	480.53	0.016	3.190E-04	-1.183	1.020E-05	-0.137	1.469E-01	652.71
1-69959045-T-C-rs481124020	MUC13	0.006	5.170E-05	-0.197	2.360E-02	NA	NA	155.52	0.019	4.150E-09	-0.658	1.560E-03	-0.135	7.853E-02	467.82	0.016	1.900E-04	-1.217	5.600E-06	NA	NA	652.85
1-69959576-C-T-rs385153041	MUC13	0.006	5.170E-05	-0.197	2.360E-02	NA	NA	155.52	0.019	6.690E-10	-0.497	5.790E-03	NA	NA	480.53	0.016	1.900E-04	-1.217	5.600E-06	NA	NA	652.85
1-69961838-T-G-rs209870119	MUC13	0.006	5.170E-05	-0.197	2.360E-02	NA	NA	155.52	0.019	6.690E-10	-0.497	5.790E-03	NA	NA	480.53	0.016	1.900E-04	-1.217	5.600E-06	NA	NA	652.85
1-69961865-A-T-rs208482355	MUC13	0.006	5.170E-05	-0.197	2.360E-02	NA	NA	155.52	0.019	6.690E-10	-0.497	5.790E-03	NA	NA	480.53	0.016	1.900E-04	-1.217	5.600E-06	NA	NA	652.85
1-71078283-A-T-rs520894556	MUC20	0.005	2.060E-04	-0.242	6.686E-03	-0.083	1.041E-01	149.67	0.017	1.710E-08	-0.614	8.690E-04	-0.259	1.423E-02	467.95	0.016	1.900E-04	-1.217	5.600E-06	NA	NA	652.85
1-71078456-G-A-rs209863266	MUC20	0.006	5.170E-05	-0.197	2.360E-02	NA	NA	155.52	0.019	6.690E-10	-0.497	5.790E-03	NA	NA	480.53	0.016	1.900E-04	-1.217	5.600E-06	NA	NA	652.85
1-71078502-A-G-rs210543483	MUC20	0.006	5.170E-05	-0.197	2.360E-02	NA	NA	155.52	0.019	6.690E-10	-0.497	5.790E-03	NA	NA	480.53	0.016	1.900E-04	-1.217	5.600E-06	NA	NA	652.85
1-71080055-C-G-rs210304339	MUC20	0.006	5.170E-05	-0.197	2.360E-02	NA	NA	155.52	0.018	5.230E-09	-0.373	5.890E-02	0.199	2.530E-02	452.85	0.016	1.900E-04	-1.217	5.600E-06	NA	NA	652.85
1-71085391-G-T-rs208323556	MUC20	0.006	6.450E-05	NA	NA	-0.083	7.160E-03	148.59	0.019	5.220E-10	NA	NA	-0.224	4.620E-04	471.25	0.016	1.900E-04	-1.217	5.600E-06	NA	NA	652.85
1-71096129-C-T-rs452308189	MUC4	0.005	1.630E-04	-0.180	4.697E-02	-0.061	1.547E-01	150.01	0.018	1.660E-09	-0.401	3.160E-02	-0.198	2.500E-02	470.58	0.016	1.900E-04	-1.217	5.600E-06	NA	NA	652.85
1-71099068-C-T-rs516084023	MUC4	0.006	5.170E-05	-0.197	2.360E-02	NA	NA	155.52	0.019	6.690E-10	-0.497	5.790E-03	NA	NA	480.53	0.016	1.900E-04	-1.217	5.600E-06	NA	NA	652.85
1-71099103-T-G-rs458620462	MUC4	0.006	5.170E-05	-0.197	2.360E-02	NA	NA	155.52	0.018	9.100E-10	-0.366	5.180E-02	0.203	2.270E-02	475.95	0.016	1.900E-04	-1.217	5.600E-06	NA	NA	652.85
1-71101197-C-T-rs435334313	MUC4	0.006	5.170E-05	-0.197	2.360E-02	NA	NA	155.52	0.018	6.620E-09	-0.389	4.290E-02	-0.201	2.930E-02	463.56	0.016	1.900E-04	-1.217	5.600E-06	NA	NA	652.85
1-71102370-G-T-rs133257965	MUC4	0.006	5.170E-05	-0.197	2.360E-02	NA	NA	155.52	0.017	7.960E-09	-0.598	1.050E-03	0.255	1.527E-02	473.87	0.016	1.900E-04	-1.217	5.600E-06	NA	NA	652.85
1-71104148-C-T-rs208812969	MUC4	0.006	2.030E-05	NA	NA	0.075	1.380E-02	152.72	0.019	2.840E-10	NA	NA	0.209	9.440E-04	475.86	0.016	1.900E-04	-1.217	5.600E-06	NA	NA	652.85
1-71105876-T-C-rs109704859	MUC4	0.006	3.520E-05	-0.193	2.690E-02	-0.183	1.593E-01	155.5	0.019	6.690E-10	-0.497	5.790E-03	NA	NA	480.53	0.017	1.060E-04	-1.197	6.990E-06	-0.789	4.449E-02	650.73
1-71107208-G-A-rs210161567	MUC4	0.006	5.170E-05	-0.197	2.360E-02	NA	NA	155.52	0.018	1.420E-09	-0.357	6.600E-02	0.165	4.440E-02	475.75	0.016	1.900E-04	-1.217	5.600E-06	NA	NA	652.85
1-71107253-G-T-rs133681725	MUC4	0.006	5.170E-05	-0.197	2.360E-02	NA	NA	155.52	0.019	6.690E-10	-0.497	5.790E-03	NA	NA	480.53	0.016	1.900E-04	-1.217	5.600E-06	NA	NA	652.85
1-71112140-G-A-rs384284796	MUC4	0.006	5.170E-05	-0.197	2.360E-02	NA	NA	155.52	0.019	2.620E-10	-0.415	2.680E-02	-0.108	1.313E-01	480.21	0.016	1.900E-04	-1.217	5.600E-06	NA	NA	652.85
1-71114449-T-C-rs210156914	MUC4	0.006	5.170E-05	-0.197	2.360E-02	NA	NA	155.52	0.019	6.690E-10	-0.497	5.790E-03	NA	NA	480.53	0.016	1.900E-04	-1.217	5.600E-06	NA	NA	652.85
1-71116493-C-T-rs463288132	MUC4	0.006	5.170E-05	-0.197	2.360E-02	NA	NA	155.52	0.019	6.690E-10	-0.497	5.790E-03	NA	NA	480.53	0.016	1.900E-04	-1.217	5.600E-06	NA	NA	652.85
1-71117959-G-A-rs110703404	MUC4	0.006	5.170E-05	-0.197	2.360E-02	NA	NA	155.52	0.019	6.690E-10	-0.497	5.790E-03	NA	NA	480.53	0.016	1.900E-04	-1.217	5.600E-06	NA	NA	652.85
1-71117981-T-C-rs379515375	MUC4	0.006	5.170E-05	-0.197	2.360E-02	NA	NA	155.52	0.018	1.390E-09	-0.403	3.950E-02	0.105	1.335E-01	476.29	0.016	1.900E-04	-1.217	5.600E-06	NA	NA	652.85
1-71119822-G-T-rs133490274	MUC4	0.006	5.170E-05	-0.197	2.360E-02	NA	NA	155.52	0.019	5.350E-10	-0.383	4.100E-02	0.184	4.630E-02	478.48	0.016	1.900E-04	-1.217	5.600E-06	NA	NA	652.85
1-71121105-G-T-rs209874806	MUC4	0.006	5.170E-05	-0.197	2.360E-02	NA	NA	155.52	0.019	6.690E-10	-0.497	5.790E-03	NA	NA	480.53	0.016	1.900E-04	-1.217	5.600E-06	NA	NA	652.85
23-27908924-G-A-rs379911053	MUC21	0.006	5.170E-05	-0.197	2.360E-02	NA	NA	155.52	0.018	1.430E-09	-0.757	4.510E-04	-0.170	2.848E-02	477.63	0.016	1.900E-04	-1.217	5.600E-06	NA	NA	652.85
23-27909368-A-C-rs382612899	MUC21	0.006	5.170E-05	-0.197	2.360E-02	NA	NA	155.52	0.019	6.690E-10	-0.497	5.790E-03	NA	NA	480.53	0.016	1.900E-04	-1.217	5.600E-06	NA	NA	652.85
25-36118604-G-A-rs43725180	MUC12	0.000	5.517E-03	-0.246	8.250E-03	0.088	1.414E-01	154.29	0.019	6.690E-10	-0.497	5.790E-03	NA	NA	480.53	0.016	1.900E-04	-1.217	5.600E-06	NA	NA	652.85
25-36119961-A-C-rs43725203	MUC12	0.006	5.170E-05	-0.197	2.360E-02	NA	NA	155.52	0.019	6.690E-10	-0.497	5.790E-03	NA	NA	480.53	0.017	1.110E-04	-1.054	2.340E-04	0.271	1.296E-01	652.51
25-36124376-A-T-rs378007912	MUC12	0.006	5.170E-05	-0.197	2.360E-02	NA	NA	155.52	0.019	6.690E-10	-0.497	5.790E-03	NA	NA	480.53	0.016	1.370E-04	-1.059	1.690E-04	-0.236	9.145E-02	651.95
25-36124542-G-A-rs43763103	MUC12	0.006	5.170E-05	-0.197	2.360E-02	NA	NA	155.52	0.019	6.690E-10	-0.497	5.790E-03	NA	NA	480.53	0.016	1.900E-04	-1.217	5.600E-06	NA	NA	652.85
25-36124568-C-G-rs384256310	MUC12	0.006	3.420E-05	-0.200	2.240E-02	-0.241	1.176E-01	155.36	0.019	6.690E-10	-0.497	5.790E-03	NA	NA	480.53	0.016	1.900E-04	-1.217	5.600E-06	NA	NA	652.85
25-36124581-C-A-rs43763105	MUC12	0.000	5.971E-03	-0.160	6.780E-02	-0.078	2.400E-02	152.32	0.019	6.690E-10	-0.497	5.790E-03	NA	NA	480.53	0.016	1.900E-04	-1.217	5.600E-06	NA	NA	652.85
25-36124674-C-G-rs43764631	MUC12	0.006	5.170E-05	-0.197	2.360E-02	NA	NA	155.52	0.019	6.690E-10	-0.497	5.790E-03	NA	NA	480.53	0.016	1.900E-04	-1.217	5.600E-06	NA	NA	652.85
25-36124688-G-A-rs43764632	MUC12	0.006	5.170E-05	-0.197	2.360E-02	NA	NA	155.52	0.019	6.690E-10	-0.497	5.790E-03	NA	NA	480.53	0.015	3.440E-04	1.149	1.890E-05	0.235	6.618E-02	651.41
25-36124806-T-C-rs43764633	MUC12	0.006	7.900E-06	NA	NA	0.106	9.340E-04	149.6	0.019	6.690E-10	-0.497	5.790E-03	NA	NA	480.53	0.016	1.900E-04	-1.217	5.600E-06	NA	NA	652.85
25-36124860-T-C-rs208639614	MUC12	0.006	5.170E-05	-0.197	2.360E-02	NA	NA	155.52	0.019	6.690E-10	-0.497	5.790E-03	NA	NA	480.53	0.016	1.900E-04	-1.217	5.600E-06	NA	NA	652.85
25-36124878-C-T-rs207552374	MUC12	0.006	5.170E-05	-0.197	2.360E-02	NA	NA	155.52	0.019	6.690E-10	-0.497	5.790E-03	NA	NA	480.53	0.016	1.900E-04	-1.217	5.600E-06	NA	NA	652.85
25-36124923-C-T-rs209938014	MUC12	0.006	7.830E-05	-0.206	1.770E-02	-0.066	6.640E-02	154.08	0.018	1.110E-09	-0.512	4.450E-03	-0.112	1.324E-01	480.22	0.016	1.900E-04	-1.217	5.600E-06	NA	NA	652.85
25-36124929-C-T-rs455010069	MUC12	0.006	5.170E-05	-0.197	2.360E-02	NA	NA	155.52	0.019	6.690E-10	-0.497	5.790E-03	NA	NA	480.53	0.017	1.030E-04	-1.048	2.280E-04	0.257	9.727E-02	648.78
25-36125015-A-C-rs447475023	MUC12	0.006	5.170E-05	-0.197	2.360E-02	NA	NA	155.52	0.019	6.690E-10	-0.497	5.790E-03	NA	NA	480.53	0.017	9.210E-05	-1.207	5.390E-06	-0.225	1.860E-02	649.21
25-36125052-G-A-rs210588156	MUC12	0.006	5.170E-05	-0.197	2.360E-02	NA	NA	155.52	0.019	6.690E-10	-0.497	5.790E-03	NA	NA	480.53	0.016	1.900E-04	-1.217	5.600E-06	NA	NA	652.85
25-36125139-C-T-rs379661977	MUC12	0.006	5.610E-05	-0.247	7.600E-03	-0.081	1.115E-01	154.94	0.019	6.690E-10	-0.497	5.790E-03	NA	NA	480.53	0.016	1.900E-04	-1.217	5.600E-06	NA	NA	652.85
25-36125142-T-C-rs208394055	MUC12	0.006	5.170E-05	-0.197	2.360E-02	NA	NA	155.52	0.019	6.690E-10	-0.497	5.790E-03	NA	NA	480.53	0.017	1.250E-04	-1.054	1.900E-04	0.260	9.098E-02	651.94
25-36125163-T-C-rs469487306	MUC12	0.006	5.320E-05	-0.253	6.530E-03	0.094	8.690E-02	154.53	0.019	6.690E-10	-0.497	5.790E-03	NA	NA	480.53	0.016	1.900E-04	-1.217	5.600E-06	NA	NA	652.85
25-36125218-T-A-rs380152318	MUC12	0.006	3.150E-05	-0.168	5.270E-02	-0.082	1.590E-02	151.59	0.019	6.690E-10	-0.497	5.790E-03	NA	NA	480.53	0.016	1.900E-04	-1.217	5.600E-06	NA	NA	652.85
25-36125228-G-A-rs436614100	MUC12	0.006	5.170E-05	-0.197	2.360E-02	NA	NA	155.52	0.019	6.690E-10	-0.497	5.790E-03	NA	NA	480.53	0.016	1.900E-04	-1.217	5.600E-06	NA	NA	652.85
25-36125252-C-G-rs475552619	MUC12	0.006	5.170E-05	-0.197	2.360E-02	NA	NA	155.52	0.019	6.690E-10	-0.497	5.790E-03	NA	NA	480.53	0.016	1.680E-04	-1.124	3.650E-05	-0.310	9.982E-02	652.09
25-36125292-A-G-rs460471693	MUC12	0.006	5.170E-05	-0.197	2.360E-02	NA	NA	155.52	0.019	6.690E-10	-0.497	5.790E-03	NA	NA	480.53	0.016	1.900E-04	-1.217	5.600E-06	NA	NA	652.85
25-36125337-C-T-rs380050173	MUC12	0.006	5.170E-05	-0.197	2.360E-02	NA	NA	155.52	0.019	6.690E-10	-0.497	5.790E-03	NA	NA	480.53	0.016	1.900E-04	-1.217	5.600E-06	NA	NA	652.85
25-36125348-C-A-rs451289443	MUC12	0.006	5.170E-05	-0.197	2.360E-02	NA	NA	155.52	0.019	3.960E-10	-0.645	1.170E-03	0.128	8.272E-02	476.11	0.017	1.270E-04	-1.451	9.030E-07	0.202	6.198E-02	646.56
25-36125426-G-A-rs382796747	MUC12	0.006	5.170E-05	-0.197	2.360E-02	NA	NA	155.52	0.019	6.690E-10	-0.497	5.790E-03	NA	NA	480.53	0.016	1.900E-04	-1.217	5.600E-06	NA	NA	652.85
25-36125789-A-G-rs432580800	MUC12	0.006	5.170E-05	-0.197	2.360E-02	NA	NA	155.52	0.018	1.290E-09	-0.605	1.270E-03	0.185	8.501E-02	476.53	0.016	1.900E-04	-1.217	5.600E-06	NA	NA	652.85
25-36125813-T-A-rs208442207	MUC12	0.006	9.180E-05	-0.246	8.060E-03	-0.079	1.250E-01	155.12	0.019	6.690E-10	-0.497	5.790E-03	NA	NA	480.53	0.016	1.900E-04	-1.217	5.600E-06	NA	NA	652.85
25-36125825-C-T-rs210308856	MUC12	0.006	5.170E-05	-0.197	2.360E-02	NA	NA	155.52	0.019	6.690E-10	-0.497	5.790E-03	NA	NA	480.53	0.016	1.900E-04	-1.217	5.600E-06	NA	NA	652.85
25-36126068-G-A-rs208831969	MUC12	0.006	5.170E-05	-0.197	2.360E-02	NA	NA	155.52	0.019	6.690E-10	-0.497	5.790E-03	NA	NA	480.53	0.016	1.900E-04	-1.217	5.600E-06	NA	NA	652.85

25-36127075-G-A-rs432239059	MUC12	0.006	5.170E-05	-0.197	2.360E-02	NA	NA	155.52	0.019	6.690E-10	-0.497	5.790E-03	NA	NA	480.53	0.016	1.900E-04	-1.217	5.600E-06	NA	NA	652.85
25-36127125-T-C-rs440961305	MUC12	0.006	5.170E-05	-0.197	2.360E-02	NA	NA	155.52	0.019	6.690E-10	-0.497	5.790E-03	NA	NA	480.53	0.016	1.900E-04	-1.217	5.600E-06	NA	NA	652.85
25-36127133-G-A-rs461491946	MUC12	0.006	5.170E-05	-0.197	2.360E-02	NA	NA	155.52	0.019	6.690E-10	-0.497	5.790E-03	NA	NA	480.53	0.016	1.900E-04	-1.217	5.600E-06	NA	NA	652.85
25-36127179-G-A-rs380618318	MUC12	0.006	5.170E-05	-0.197	2.360E-02	NA	NA	155.52	0.019	6.690E-10	-0.497	5.790E-03	NA	NA	480.53	0.016	1.900E-04	-1.217	5.600E-06	NA	NA	652.85
25-36127963-T-G-rs43763093	MUC12	0.006	5.170E-05	-0.197	2.360E-02	NA	NA	155.52	0.019	6.690E-10	-0.497	5.790E-03	NA	NA	480.53	0.016	1.900E-04	-1.217	5.600E-06	NA	NA	652.85
25-36127984-G-A-rs210595462	MUC12	0.006	5.170E-05	-0.197	2.360E-02	NA	NA	155.52	0.019	6.690E-10	-0.497	5.790E-03	NA	NA	480.53	0.016	3.030E-04	-1.261	2.790E-06	-0.157	1.157E-01	652.33
25-36128054-A-G-rs208401341	MUC12	0.006	5.170E-05	-0.197	2.360E-02	NA	NA	155.52	0.019	6.690E-10	-0.497	5.790E-03	NA	NA	480.53	0.017	1.310E-04	-1.221	5.080E-06	-0.198	6.616E-02	645.33
25-36128929-C-T-rs210014138	MUC12	0.006	5.170E-05	-0.197	2.360E-02	NA	NA	155.52	0.019	6.690E-10	-0.497	5.790E-03	NA	NA	480.53	0.016	2.510E-04	-1.159	1.430E-05	0.342	3.684E-02	650.41
25-36129062-A-G-rs382384702	MUC12	0.006	5.170E-05	-0.197	2.360E-02	NA	NA	155.52	0.019	6.690E-10	-0.497	5.790E-03	NA	NA	480.53	0.016	3.310E-04	-1.222	5.790E-06	-0.337	3.934E-02	637.91
25-36129440-T-G-rs471303474	MUC12	0.006	5.170E-05	-0.197	2.360E-02	NA	NA	155.52	0.018	1.750E-08	-0.419	2.329E-02	-0.171	9.340E-03	461.82	0.016	1.900E-04	-1.217	5.600E-06	NA	NA	652.85
25-36129970-A-C-rs210809242	MUC12	0.005	1.590E-04	-0.140	1.316E-01	0.096	8.393E-02	154.47	0.018	3.050E-09	-0.395	3.930E-02	0.169	1.383E-01	480.29	0.016	1.900E-04	-1.217	5.600E-06	NA	NA	652.85
25-36130313-T-C-rs450453591	MUC12	0.006	5.170E-05	-0.197	2.360E-02	NA	NA	155.52	0.019	6.690E-10	-0.497	5.790E-03	NA	NA	480.53	0.016	1.900E-04	-1.217	5.600E-06	NA	NA	652.85
25-36130596-G-A-rs450822779	MUC12	0.006	5.170E-05	-0.197	2.360E-02	NA	NA	155.52	0.019	6.690E-10	-0.497	5.790E-03	NA	NA	480.53	0.017	1.020E-04	-1.253	2.920E-06	0.170	8.646E-02	648.59
25-36130632-A-G-rs210987447	MUC12	0.006	1.060E-04	-0.163	7.033E-02	0.083	1.293E-01	155.17	0.019	6.690E-10	-0.497	5.790E-03	NA	NA	480.53	0.016	1.900E-04	-1.217	5.600E-06	NA	NA	652.85
25-36130650-T-C-rs207765355	MUC12	0.006	5.170E-05	-0.197	2.360E-02	NA	NA	155.52	0.019	6.690E-10	-0.497	5.790E-03	NA	NA	480.53	0.016	1.900E-04	-1.217	5.600E-06	NA	NA	652.85
25-36146175-A-C-rs524371728	MUC12	0.006	5.170E-05	-0.197	2.360E-02	NA	NA	155.52	0.019	6.690E-10	-0.497	5.790E-03	NA	NA	480.53	0.016	1.900E-04	-1.217	5.600E-06	NA	NA	652.85
25-36146473-G-A-rs522033207	MUC12	0.006	5.170E-05	-0.197	2.360E-02	NA	NA	155.52	0.019	6.690E-10	-0.497	5.790E-03	NA	NA	480.53	0.016	1.900E-04	-1.217	5.600E-06	NA	NA	652.85
25-36146649-A-C-rs381166539	MUC12	0.006	5.170E-05	-0.197	2.360E-02	NA	NA	155.52	0.019	6.690E-10	-0.497	5.790E-03	NA	NA	480.53	0.016	1.900E-04	-1.217	5.600E-06	NA	NA	652.85
25-36146683-T-C-rs207666727	MUC12	0.006	5.170E-05	-0.197	2.360E-02	NA	NA	155.52	0.019	6.690E-10	-0.497	5.790E-03	NA	NA	480.53	0.016	1.900E-04	-1.217	5.600E-06	NA	NA	652.85
25-36147807-C-T-rs211277321	MUC12	0.006	5.170E-05	-0.197	2.360E-02	NA	NA	155.52	0.019	6.080E-10	-0.672	8.540E-04	-0.213	5.281E-02	478.7	0.016	1.900E-04	-1.217	5.600E-06	NA	NA	652.85
25-36148129-T-C-rs384275373	MUC12	0.006	3.610E-05	-0.191	2.930E-02	0.055	1.152E-01	155.33	0.019	6.690E-10	-0.497	5.790E-03	NA	NA	480.53	0.016	1.900E-04	-1.217	5.600E-06	NA	NA	652.85
25-36166928-G-C-rs433140174	MUC12	0.006	5.170E-05	-0.197	2.360E-02	NA	NA	155.52	0.019	6.690E-10	-0.497	5.790E-03	NA	NA	480.53	0.016	1.900E-04	-1.217	5.600E-06	NA	NA	652.85
25-36167786-C-T-rs207678154	MUC12	0.006	5.170E-05	-0.197	2.360E-02	NA	NA	155.52	0.018	4.540E-09	-0.637	1.270E-03	0.155	1.439E-01	471.7	0.016	1.900E-04	-1.217	5.600E-06	NA	NA	652.85
25-36167802-G-A-rs382870075	MUC12	0.006	5.170E-05	-0.197	2.360E-02	NA	NA	155.52	0.019	6.690E-10	-0.497	5.790E-03	NA	NA	480.53	0.017	8.510E-05	-1.362	7.520E-07	-0.240	2.920E-02	646.74
25-36168706-A-G-rs455849525	MUC12	0.006	5.170E-05	-0.197	2.360E-02	NA	NA	155.52	0.019	6.690E-10	-0.497	5.790E-03	NA	NA	480.53	0.016	1.900E-04	-1.217	5.600E-06	NA	NA	652.85
25-36168827-A-G-rs469425205	MUC12	0.006	5.170E-05	-0.197	2.360E-02	NA	NA	155.52	0.019	6.690E-10	-0.497	5.790E-03	NA	NA	480.53	0.016	1.900E-04	-1.217	5.600E-06	NA	NA	652.85
25-36168911-A-G-rs209852756	MUC12	0.006	5.170E-05	-0.197	2.360E-02	NA	NA	155.52	0.019	6.690E-10	-0.497	5.790E-03	NA	NA	480.53	0.016	1.900E-04	-1.217	5.600E-06	NA	NA	652.85
25-36169022-C-A-rs470670444	MUC12	0.006	5.170E-05	-0.197	2.360E-02	NA	NA	155.52	0.019	6.690E-10	-0.497	5.790E-03	NA	NA	480.53	0.016	1.900E-04	-1.217	5.600E-06	NA	NA	652.85
25-36174076-T-A-rs471608849	MUC12	0.006	5.170E-05	-0.197	2.360E-02	NA	NA	155.52	0.019	6.690E-10	-0.497	5.790E-03	NA	NA	480.53	0.016	1.900E-04	-1.217	5.600E-06	NA	NA	652.85
25-36177035-G-T-rs43726625	MUC12	0.006	5.170E-05	-0.197	2.360E-02	NA	NA	155.52	0.019	6.690E-10	-0.497	5.790E-03	NA	NA	480.53	0.016	1.900E-04	-1.217	5.600E-06	NA	NA	652.85
25-36177161-A-G-rs208101045	MUC12	0.006	5.170E-05	-0.197	2.360E-02	NA	NA	155.52	0.019	6.690E-10	-0.497	5.790E-03	NA	NA	480.53	0.016	1.900E-04	-1.217	5.600E-06	NA	NA	652.85
25-36264390-A-C-rs43725892	MUC12	0.006	5.170E-05	-0.197	2.360E-02	NA	NA	155.52	0.019	6.690E-10	-0.497	5.790E-03	NA	NA	480.53	0.016	1.900E-04	-1.217	5.600E-06	NA	NA	652.85
25-36264843-T-C-rs524961186	MUC12	0.006	5.170E-05	-0.197	2.360E-02	NA	NA	155.52	0.019	6.690E-10	-0.497	5.790E-03	NA	NA	480.53	0.016	1.900E-04	-1.217	5.600E-06	NA	NA	652.85
25-36266585-C-T-rs211227324	MUC12	0.006	6.690E-05	-0.246	6.050E-03	-0.076	1.224E-01	151.64	0.019	6.690E-10	-0.497	5.790E-03	NA	NA	480.53	0.016	1.900E-04	-1.217	5.600E-06	NA	NA	652.85
25-36266591-G-A-rs208665498	MUC12	0.006	5.170E-05	-0.197	2.360E-02	NA	NA	155.52	0.019	6.690E-10	-0.497	5.790E-03	NA	NA	480.53	0.016	1.900E-04	-1.217	5.600E-06	NA	NA	652.85
25-36267719-T-G-rs210476511	MUC12	0.006	5.170E-05	-0.197	2.360E-02	NA	NA	155.52	0.019	6.690E-10	-0.497	5.790E-03	NA	NA	480.53	0.016	1.900E-04	-1.217	5.600E-06	NA	NA	652.85
25-36267882-C-T-rs135614410	MUC12	0.006	5.170E-05	-0.197	2.360E-02	NA	NA	155.52	0.019	6.690E-10	-0.497	5.790E-03	NA	NA	480.53	0.016	1.900E-04	-1.217	5.600E-06	NA	NA	652.85
25-36268034-A-G-rs208036323	MUC12	0.006	5.170E-05	-0.197	2.360E-02	NA	NA	155.52	0.019	6.690E-10	-0.497	5.790E-03	NA	NA	480.53	0.016	1.900E-04	-1.217	5.600E-06	NA	NA	652.85
25-36274534-T-A-rs208879451	MUC12	0.006	5.170E-05	-0.197	2.360E-02	NA	NA	155.52	0.019	6.690E-10	-0.497	5.790E-03	NA	NA	480.53	0.016	1.900E-04	-1.217	5.600E-06	NA	NA	652.85
25-36274568-C-G-rs210320285	MUC12	0.006	5.170E-05	-0.197	2.360E-02	NA	NA	155.52	0.019	6.690E-10	-0.497	5.790E-03	NA	NA	480.53	0.016	1.900E-04	-1.217	5.600E-06	NA	NA	652.85
29-51504286-C-T-rs456327378	MUC2	0.006	5.170E-05	-0.197	2.360E-02	NA	NA	155.52	0.019	6.690E-10	-0.497	5.790E-03	NA	NA	480.53	0.017	1.290E-04	-1.054	2.090E-04	-0.167	1.088E-01	652.23
3-15484489-C-T-rs432755602	MUC1	0.006	5.170E-05	-0.197	2.360E-02	NA	NA	155.52	0.019	6.690E-10	-0.497	5.790E-03	NA	NA	480.53	0.016	1.900E-04	-1.217	5.600E-06	NA	NA	652.85
5-40452937-G-T-rs43049932	MUC19	0.006	5.170E-05	-0.197	2.360E-02	NA	NA	155.52	0.019	6.690E-10	-0.497	5.790E-03	NA	NA	480.53	0.016	1.900E-04	-1.217	5.600E-06	NA	NA	652.85
5-40563598-A-G-rs475040730	MUC19	0.006	5.170E-05	-0.197	2.360E-02	NA	NA	155.52	0.019	6.690E-10	-0.497	5.790E-03	NA	NA	480.53	0.016	1.900E-04	-1.217	5.600E-06	NA	NA	652.85
5-40585825-T-C-rs43435100	MUC19	0.006	4.990E-05	-0.170	5.530E-02	-0.192	1.497E-01	154.38	0.019	4.350E-10	-0.417	2.220E-02	-0.564	3.920E-02	475.56	0.016	1.900E-04	-1.217	5.600E-06	NA	NA	652.85
5-40598970-G-A-rs109902399	MUC19	0.006	5.170E-05	-0.197	2.360E-02	NA	NA	155.52	0.019	6.690E-10	-0.497	5.790E-03	NA	NA	480.53	0.016	1.900E-04	-1.217	5.600E-06	NA	NA	652.85
5-40598974-A-C-rs110889423	MUC19	0.006	5.170E-05	-0.197	2.360E-02	NA	NA	155.52	0.019	6.690E-10	-0.497	5.790E-03	NA	NA	480.53	0.016	1.900E-04	-1.217	5.600E-06	NA	NA	652.85
5-40599195-G-T-rs110499319	MUC19	0.006	5.170E-05	-0.197	2.360E-02	NA	NA	155.52	0.019	6.690E-10	-0.497	5.790E-03	NA	NA	480.53	0.016	1.900E-04	-1.217	5.600E-06	NA	NA	652.85
5-40599208-A-G-rs110297500	MUC19	0.006	5.170E-05	-0.197	2.360E-02	NA	NA	155.52	0.019	6.690E-10	-0.497	5.790E-03	NA	NA	480.53	0.016	1.900E-04	-1.217	5.600E-06	NA	NA	652.85
5-40599220-C-A-rs132946965	MUC19	0.006	5.170E-05	-0.197	2.360E-02	NA	NA	155.52	0.019	6.690E-10	-0.497	5.790E-03	NA	NA	480.53	0.016	1.900E-04	-1.217	5.600E-06	NA	NA	652.85
5-40599364-G-A-rs109084320	MUC19	0.006	5.170E-05	-0.197	2.360E-02	NA	NA	155.52	0.018	4.040E-09	-0.385	4.860E-02	0.134	7.890E-02	473.9	0.016	1.900E-04	-1.217	5.600E-06	NA	NA	652.85
5-40599418-G-C-rs109969033	MUC19	0.006	5.170E-05	-0.197	2.360E-02	NA	NA	155.52	0.019	4.650E-10	-0.490	6.320E-03	-0.507	1.074E-01	479.88	0.016	1.450E-04	-1.206	6.140E-06	-0.843	6.792E-02	651.45
5-40599460-G-C-rs110407313	MUC19	0.006	5.170E-05	-0.197	2.360E-02	NA	NA	155.52	0.019	6.690E-10	-0.497	5.790E-03	NA	NA	480.53	0.016	1.900E-04	-1.217	5.600E-06	NA	NA	652.85
5-40604197-G-T-rs432355789	MUC19	0.006	5.170E-05	-0.197	2.360E-02	NA	NA	155.52	0.019	6.690E-10	-0.497	5.790E-03	NA	NA	480.53	0.016	1.900E-04	-1.217	5.600E-06	NA	NA	652.85
5-40604431-G-A-rs455144379	MUC19	0.005	2.700E-04	-0.253	5.850E-03	0.163	5.942E-02	153.9	0.018	8.470E-09	-0.616	1.170E-03	0.347	5.126E-02	478.65	0.016	1.900E-04	-1.217	5.600E-06	NA	NA	652.85
5-40604539-T-G-rs42999193	MUC19	0.006	5.170E-05	-0.197	2.360E-02	NA	NA	155.52	0.019	6.690E-10	-0.497	5.790E-03	NA	NA	480.53	0.016	1.900E-04	-1.217	5.600E-06	NA	NA	652.85
5-40604569-G-T-rs42999194	MUC19	0.006	5.170E-05	-0.197	2.360E-02	NA	NA	155.52	0.019	6.690E-10	-0.497	5.790E-03	NA	NA	480.53	0.016	1.900E-04	-1.217	5.600E-06	NA	NA	652.85
5-40604737-G-T-rs42999197	MUC19	0.006	5.170E-05	-0.197	2.360E-02	NA	NA	155.52	0.019	6.690E-10	-0.497	5.790E-03	NA	NA	480.53	0.016	1.900E-04	-1.217	5.600E-06	NA	NA	652.85
5-40618749-G-A-rs383139383	MUC19	0.006	5.170E-05	-0.197	2.360E-02	NA	NA	155.52	0.019	6.690E-10	-0.497	5.790E-03	NA	NA	480.53	0.016	1.900E-04	-1.217	5.600E-06	NA	NA	652.85
5-40643873-T-C-rs136517752	MUC19	0.006	5.170E-05	-0.197	2.360E-02	NA	NA	155.52	0.019	6.690E-10	-0.497	5.790E-03	NA	NA	480.53	0.016	1.900E-04	-1.217	5.600E-06	NA	NA	652.85
5-40643965-C-T-rs446954663	MUC19	0.006	5.170E-05	-0.197	2.360E-02	NA	NA	155.52	0.018	1.490E-09	-0.397	3.480E-02	-0.147	1.035E-01	478.19	0.016	1.900E-04	-1.217	5.600E-06	NA	NA	652.85
5-40653866-C-T-rs383263575	MUC19	0.006	5.170E-05	-0.197	2.360E-02	NA	NA	155.52	0.019	6.690E-10	-0.497	5.790E-03	NA	NA	480.53	0.016	1.900E-04	-1.217	5.600E-06	NA	NA	652.85
5-40654105-C-T-rs453934939	MUC19	0.005	1.880E-04	-0.193	2.637E-02	-0.177	1.122E-01	154.95	0.019	6.690E-10	-0.497	5.790E-03	NA	NA	480.53	0.016	1.900E-04	-1.217	5.600E-06	NA	NA	652.85
7-14320716-C-A-rs209024096	MUC16	0.006	5.170E-05	-0.197	2.360E-02	NA	NA	155.52	0.018	4.060E-09	-0.398	3.420E-02	-0.146	5.800E-02	475.45	0.016	1.900E-04	-1.217	5.600E-06	NA	NA	652.85
7-14323295-G-C-rs209334744	MUC16	0.006	5.170E-05	-0.197	2.360E-02	NA	NA	155.52	0.019	6.690E-10	-0.497	5.790E-03	NA	NA	480.53	0.016	1.900E-04	-1.217	5.600E-06	NA	NA	652.85
7-14326280-T-C-rs460291013	MUC16	0.006	5.170E-05	-0.197	2.360E-02	NA	NA	155.52	0.019	6.690E-10	-0.497	5.790E-03	NA	NA	480.53	0.016	1.900E-04	-1.217	5.600E-06	NA	NA	652.85
7-14328090-T-A-rs209627122	MUC16	0.006	5.170E-05	-0.197	2.360E-02	NA	NA	155.52	0.019	6.690E-10	-0.497	5.790E-03	NA	NA	480.53	0.016	1.900E-04	-1.217	5.600E-06	NA	NA	652.85
7-14330874-C-T-rs435626914	MUC16	0.006	5.170E-05	-0.197	2.360E-02	NA	NA	155.52	0.019	6.690E-10	-0.497	5.790E-03	NA	NA	480.53	0.016	1.900E-04	-1.217	5.600E-06	NA	NA	652.85
7-14335319-G-T-rs110563141	MUC16	0.006	5.170E-05	-0.197	2.360E-02	NA	NA	155.52	0.019	6.690E-10	-0.497	5.790E-03	NA	NA	480.53	0.016	1.900E-04	-1.217	5.600E-06	NA	NA	652.85
7-14338508-C-T-rs379918306	MUC16	0.006	5.170E-05	-0.197	2.360E-02	NA	NA	155.52	0.019	2.830E-10	NA	NA	0.212	1.370E-03	477.85	0.016	1.900E-04	-1.217	5.600E-06	NA	NA	652.85
7-14343366-C-T-rs209011074	MUC16	0.006	5.170E-05	-0.197	2.360E-02	NA	NA	155.52	0.019	1.170E-09	-0.447	1.460E-02	-0.122	1.081E-01	478.79	0.016	2.540E-04	-1.145	2.590E-05	-0.176	1.139E-01	650.43
7-14344266-A-C-rs433704502	MUC16	0.006	5.170E-05	-0.197	2.360E-02	NA	NA	155.52	0.019	6.690E-10	-0.497	5.790E-03	NA	NA	480.53	0.016	1.900E-04	-1.217	5.600E-06	NA	NA	652.85
7-14354974-G-A-rs109735464	MUC16	0.005	2.390E-04	-0.256	5.236E-03	0.080	1.427E-01	151.89	0.018	6.980E-09	-0.623	1.060E-03	0.202	7.237E-02	475.29	0.015	6.930E-04	-1.381	1.130E-06	0.312	5.874E-02	647.17
7-14364954-G-A-rs208573389	MUC16	0.006	5.170E-05	-0.197	2.360E-02	NA	NA	155.52	0.019	6.690E-10	-0.497	5.790E-03	NA	NA	480.53	0.016	2.920E-04	-1.226	4.430E-06	0.916	7.610E-02	651.64
7-14365027-G-A-rs211153134	MUC16	0.006	5.170E-05	-0.197	2.360E-02	NA	NA	155.52	0.019	6.690E-10	-0.497	5.790E-03	NA	NA	480.53	0.016	1.900E-04	-1.217	5.600E-06	NA	NA	652.85
7-14367888-T-A-rs381206569	MUC16	0.006	5.170E-05	-0.197	2.360E-02	NA	NA	155.52	0.019	6.690E-10	-0.497	5.790E-03	NA	NA	480.53	0.016	1.900E-04	-1.217	5.600E-06	NA	NA	652.85

7-14372099-C-T-rs208778554	MUC16	0.006	5.170E-05	-0.197	2.360E-02	NA	NA	155.52	0.019	6.690E-10	-0.497	5.790E-03	NA	NA	480.53	0.016	1.900E-04	-1.217	5.600E-06	NA	NA	652.85
7-14373458-T-G-rs110614577	MUC16	0.006	5.170E-05	-0.197	2.360E-02	NA	NA	155.52	0.019	6.690E-10	-0.497	5.790E-03	NA	NA	480.53	0.016	1.900E-04	-1.217	5.600E-06	NA	NA	652.85
7-14384888-G-C-rs208967240	MUC16	0.006	5.170E-05	-0.197	2.360E-02	NA	NA	155.52	0.019	6.690E-10	-0.497	5.790E-03	NA	NA	480.53	0.016	1.900E-04	-1.217	5.600E-06	NA	NA	652.85
7-14385123-C-G-rs208623006	MUC16	0.006	5.170E-05	-0.197	2.360E-02	NA	NA	155.52	0.019	6.690E-10	-0.497	5.790E-03	NA	NA	480.53	0.016	1.900E-04	-1.217	5.600E-06	NA	NA	652.85
7-14387039-C-T-rs447008800	MUC16	0.006	5.170E-05	-0.197	2.360E-02	NA	NA	155.52	0.019	6.690E-10	-0.497	5.790E-03	NA	NA	480.53	0.016	1.900E-04	-1.217	5.600E-06	NA	NA	652.85
7-14387078-G-A-rs451081431	MUC16	0.006	5.170E-05	-0.197	2.360E-02	NA	NA	155.52	0.019	6.690E-10	-0.497	5.790E-03	NA	NA	480.53	0.016	1.900E-04	-1.217	5.600E-06	NA	NA	652.85
7-14387390-A-G-rs385478124	MUC16	0.006	5.170E-05	-0.197	2.360E-02	NA	NA	155.52	0.019	6.690E-10	-0.497	5.790E-03	NA	NA	480.53	0.016	1.900E-04	-1.217	5.600E-06	NA	NA	652.85
7-14387763-T-G-rs462387951	MUC16	0.006	5.170E-05	-0.197	2.360E-02	NA	NA	155.52	0.019	5.200E-10	NA	NA	0.218	1.070E-03	476.21	0.016	1.900E-04	-1.217	5.600E-06	NA	NA	652.85
7-14388834-T-C-rs136758635	MUC16	0.006	5.170E-05	-0.197	2.360E-02	NA	NA	155.52	0.019	6.690E-10	-0.497	5.790E-03	NA	NA	480.53	0.016	1.900E-04	-1.217	5.600E-06	NA	NA	652.85
7-14388897-C-T-rs134698651	MUC16	0.006	5.170E-05	-0.197	2.360E-02	NA	NA	155.52	0.019	6.690E-10	-0.497	5.790E-03	NA	NA	480.53	0.016	1.900E-04	-1.217	5.600E-06	NA	NA	652.85
7-14389020-T-G-rs136055907	MUC16	0.006	4.000E-05	-0.303	1.520E-03	0.085	1.721E-02	149.02	0.019	5.810E-10	-0.650	1.080E-03	0.138	6.094E-02	470.96	0.016	1.900E-04	-1.217	5.600E-06	NA	NA	652.85
7-14389127-A-G-rs110948032	MUC16	0.006	5.170E-05	-0.197	2.360E-02	NA	NA	155.52	0.019	3.520E-09	-0.534	9.700E-03	0.133	1.134E-01	440.12	0.016	1.900E-04	-1.217	5.600E-06	NA	NA	652.85
7-14390301-C-T-rs454517095	MUC16	0.006	5.170E-05	-0.197	2.360E-02	NA	NA	155.52	0.019	6.690E-10	-0.497	5.790E-03	NA	NA	480.53	0.016	1.900E-04	-1.217	5.600E-06	NA	NA	652.85
7-14390542-C-A-rs210577627	MUC16	0.006	5.230E-05	-0.176	4.420E-02	-0.083	6.540E-02	154.06	0.019	6.690E-10	-0.497	5.790E-03	NA	NA	480.53	0.016	1.940E-04	-1.154	1.720E-05	-0.244	7.303E-02	651.58
7-14391024-A-G-rs208869147	MUC16	0.006	4.710E-05	-0.328	8.280E-03	-0.066	1.381E-01	155.28	0.019	5.350E-10	-0.805	1.670E-03	-0.156	8.881E-02	479.57	0.016	1.900E-04	-1.217	5.600E-06	NA	NA	652.85
7-14405094-C-A-rs109547736	MUC16	0.006	5.170E-05	-0.197	2.360E-02	NA	NA	155.52	0.019	6.690E-10	-0.497	5.790E-03			480.53	0.016	1.900E-04	-1.217	5.600E-06	NA	NA	652.85
7-14405096-A-G-rs110228721	MUC16	0.006	5.170E-05	-0.197	2.360E-02	NA	NA	155.52	0.019	2.800E-10	-0.753	1.030E-03	0.187	7.223E-02	475.88	0.016	1.900E-04	-1.217	5.600E-06	NA	NA	652.85
7-14410427-C-T-rs440589844	MUC16	0.006	5.170E-05	-0.197	2.360E-02	NA	NA	155.52	0.019	6.690E-10	-0.497	5.790E-03	NA	NA	480.53	0.016	1.900E-04	-1.217	5.600E-06	NA	NA	652.85
7-14410434-G-C-rs209094158	MUC16	0.006	5.170E-05	-0.197	2.360E-02	NA	NA	155.52	0.019	6.690E-10	-0.497	5.790E-03	NA	NA	480.53	0.016	1.900E-04	-1.217	5.600E-06	NA	NA	652.85
7-14415699-A-G-rs109586044	MUC16	0.006	5.170E-05	-0.197	2.360E-02	NA	NA	155.52	0.019	8.110E-10	-0.327	1.080E-01	0.144	5.070E-02	471.38	0.016	1.900E-04	-1.217	5.600E-06	NA	NA	652.85
7-14415792-G-A-rs108987183	MUC16	0.006	5.170E-05	-0.197	2.360E-02	NA	NA	155.52	0.018	2.160E-09	-0.309	1.147E-01	0.195	1.050E-02	462.42	0.016	1.900E-04	-1.217	5.600E-06	NA	NA	652.85
7-14416102-A-G-rs109113961	MUC16	0.006	5.170E-05	-0.197	2.360E-02	NA	NA	155.52	0.019	6.690E-10	-0.497	5.790E-03	NA	NA	480.53	0.016	1.900E-04	-1.217	5.600E-06	NA	NA	652.85
7-14416363-T-G-rs474361208	MUC16	0.006	5.170E-05	-0.197	2.360E-02	NA	NA	155.52	0.019	3.450E-10	-0.523	3.730E-03	0.151	1.014E-01	479.79	0.016	1.900E-04	-1.217	5.600E-06	NA	NA	652.85
7-14416701-C-T-rs209163760	MUC16	0.006	5.170E-05	-0.197	2.360E-02	NA	NA	155.52	0.019	6.690E-10	-0.497	5.790E-03	NA	NA	480.53	0.015	3.880E-04	-1.309	1.590E-06	0.309	7.734E-02	651.67
7-14417314-T-C-rs525901464	MUC16	0.006	2.990E-05	-0.373	1.590E-03	0.093	2.729E-02	152.55	0.019	2.860E-10	-0.858	4.290E-04	0.193	2.708E-02	477.54	0.016	1.900E-04	-1.217	5.600E-06	NA	NA	652.85
7-14417638-G-A-rs435681981	MUC16	0.006	5.170E-05	-0.197	2.360E-02	NA	NA	155.52	0.019	6.690E-10	-0.497	5.790E-03	NA	NA	480.53	0.016	1.900E-04	-1.217	5.600E-06	NA	NA	652.85

Notes:

[1]MAB: Multibreed Angus-Brahman

[2]AIC: Akaike information criterion

[3]NA reflects that this corresponding variable is not included in the model. It is decided based on its large P value when this variable is included in the model as well as a larger AIC value compared to that of the model exluding this variable.
